# A novel HVEM-Fc recombinant protein for lung cancer immunotherapy

**DOI:** 10.1186/s13046-025-03324-8

**Published:** 2025-02-20

**Authors:** Yuanshan Yao, Bin Li, Jing Wang, Chunji Chen, Wen Gao, Chunguang Li

**Affiliations:** 1 Department of Thoracic Surgery, Huadong Hospital Affiliated to Fudan University, Shanghai, 200041 China; 2 Department of Thoracic Surgery, Shanghai Chest Hospital, Shanghai Jiao Tong University School of Medicine, Shanghai, 200030 China; 3 Shanghai Institute of Thoracic Oncology, Shanghai, 200030 China

## Abstract

**Background:**

The ubiquitously expressed transmembrane protein, Herpesvirus Entry Mediator (HVEM), functions as a molecular switch, capable of both activating and inhibiting the immune response depending on its interacting ligands. HVEM-Fc is a novel recombinant fusion protein with the potential to eradicate tumor cells.

**Methods:**

The anti-tumor efficacy of HVEM-Fc was evaluated in *C57BL/6* mice-bearing lung cancer models: a syngeneic model and an orthotopic model of mouse lung cancer. Additionally, patient-derived organoids were employed in conjunction with T cell co-culture systems. To investigate the underlying mechanisms, a comprehensive array of techniques was utilized, including single-cell RNA sequencing, spatial transcriptomics, bulk RNA sequencing, and flow cytometry. Furthermore, the anti-tumor effects of HVEM-Fc in combination with Programmed Death-1 (PD-1) inhibitors were assessed. Finally, mouse immune cell depletion antibodies were used to elucidate the underlying mechanisms of action.

**Results:**

In vivo, 1 mg/kg HVEM-Fc demonstrated effective inhibition of tumor growth and metastasis in *C57BL/6* mice bearing lung cancer model and a KP orthotopic model of mouse lung cancer. Multi-omics analysis showed that HVEM-Fc induced an immune-stimulatory microenvironment. Notably, the combination of HVEM-Fc with a PD-1 inhibitor demonstrated the most potent inhibition of tumor cell growth. In vitro, HVEM-Fc was validated to eradicate tumor cells through the activation of T cells in both non-small cell lung cancer (NSCLC) organoids and T cell co-culture models.

**Conclusions:**

Our data demonstrate that HVEM-Fc exerts a strong signal that augments and prolongs T-cell activity in both murine models and human NSCLC organoid models. Moreover, the combination of HVEM-Fc with a PD-1 inhibitor yields the most effective anti-tumor outcomes.

**Supplementary Information:**

The online version contains supplementary material available at 10.1186/s13046-025-03324-8.

## Background

In recent years, immune checkpoint inhibitors have revolutionized the treatment landscape of late-stage lung cancer. While Programmed Death-1 (PD-1)/Programmed Death-Ligand 1 (PD-L1) antibody therapy has achieved success in treating advanced non-small cell lung cancer (NSCLC), only a minority of patients benefit from immunotherapy, and the high rate of immune-related adverse events remains unsatisfactory [1–3]. Hence, there is a need to explore other novel immune checkpoint targets.

The second signal, known as costimulatory, encompasses the B7-CD28 family genes (*n* = 14) and tumor necrosis factor (TNF) family genes (*n* = 48). Several TNF family genes provide essential co-stimulatory signals for immune cell proliferation, differentiation, and survival. Notably, antibodies targeting corresponding immune checkpoints have already entered Phase I clinical trials [4]. Preclinical results have demonstrated that agonistic antibodies targeting 4-1BB (TNFRSF9) and OX40 (TNFRSF4) can elicit long-term anti-tumor immunity in humanized mouse models [5, 6]. However, the high rate of kidney or liver toxicity has limited further clinical translation of these antibodies [7].

The TNF receptor superfamily member herpesvirus entry mediator (HVEM) (TNFRSF14) was initially identified as a receptor for viral infection [8]. HVEM exhibits ubiquitous expression across various cell types, encompassing both typical immune cells and several non-hematopoietic cells [9]. HVEM functions as a molecular switch, capable of either activating or inhibiting the immune response depending on the interacting ligand that binds to its receptor. For example, binding to its ligand, B- and T-lymphocyte attenuator (BTLA), initiates an inhibitory signal to effector T cells [10]. Conversely, interaction with TNFSF14 (LIGHT) and LTα transmits signals that subsequently lead to T cell activation [11]. A significant portion of current research centers on novel antibodies targeting the HVEM-BTLA pathway [12]. Olive et al. reported that an anti-HVEM antibody targeting the HVEM-BTLA complex could restrict tumor growth in a humanized mouse model expressing both HVEM and BTLA [13]. However, monoclonal antibody generation is a costly and time-consuming endeavor. Recently, Hwang et al. demonstrated that injection of recombinant PD-1 protein exhibited an anti-tumor effect comparable to that of the anti-PD-1 antibody [14]. In addition, TTI-621 (recombinant SIRPα-IgG1 Fc) displayed a potent tumor suppressive effect and well-tolerated immunotherapy-associated side effects in a Phase I study treating advanced lymphomas, potentially paving the way for a new era in solid tumor treatment. Recombinant proteins offer advantages such as low cost, small molecular weight, and rapid preparation [15]. Building upon this foundation of research, we constructed a recombinant soluble form of the HVEM protein, designated HVEM-Fc.

Our findings demonstrated that 1 mg/kg HVEM-Fc could trigger potent immune activation, resulting in the elimination of tumor cells. Moreover, the combination of HVEM-Fc with a PD-1 inhibitor exhibited the most potent anti-tumor efficacy. In addition, through the integration of multi-omics technologies and patient-derived organoid models coupled with T cell co-culture systems, we observed that HVEM-Fc primarily triggered a 'hot' immune microenvironment through T cell-mediated responses.

## Methods and materials

### HVEM-Fc recombinant protein

Mouse HVEM-Fc protein comprised the N-terminal domain of mouse HVEM (amino acids Gln39-Val207; GenBank: NP849262) fused to a mouse IgG1 Fc tag at the C-terminus. Similarly, human HVEM-Fc protein consisted of the N-terminal domain of human HVEM (GenBank: AAQ89238) fused to a human IgG1 Fc tag. Recombinant HVEM-Fc proteins (mouse HVEM-Fc: Cat No. CJ90; human HVEM-Fc: Cat No. CW43) were generated by Novoprotein Company (China Suzhou). Control recombinant mouse IgG1 Fc (Cat No. CX85) was also procured from the same source. The purity of all proteins was determined to be greater than 95% by high-performance liquid chromatography (HPLC) analysis.

### Cell lines

Mouse Lewis lung carcinoma cells expressing luciferase (LLC-Luc) and CMT-64 cells were maintained in Dulbecco's Modified Eagle Medium (DMEM) supplemented with 10% fetal bovine serum (X–Y Biotechnology, Austria). These cell lines were obtained from Procell Life Technology Company (Wuhan, China) and authenticated by short tandem repeat (STR) profiling.

### Mouse

Kras^LSL−G12D^/ + Tp53^fl/fl^ (KP) mice were obtained from Saiye Company in Suzhou. The presence of lung tumors in these mice was confirmed at 8 weeks of age by micro-CT scanning. Six-week-old wild-type immunocompetent *C57BL/6* mice and immunodeficient NSG mice were also procured from the same vendor. All mice were maintained under specific pathogen-free conditions within the animal facility at Fudan University.

### Mouse experiment protocol and tumor treatment

Tumorigenicity assays were conducted using subcutaneous tumor formation and tail vein injection models. For both models, 1 × 10^6^ Lewis or CMT-64 cells were injected subcutaneously into the left flank or intravenously into the tail vein, respectively. Following engraftment on Day 1, mice received intraperitoneal injections of HVEM-Fc, a PD-1 antibody, or a control anti-human IgG Fc antibody every 3 days. Each mouse received six doses of treatment and was subsequently sacrificed on Day 18. In the tail vein injection model, a separate cohort of mice was also sacrificed on Day 23, and their lungs were harvested for analysis following in vivo imaging. For the dual-immunotherapy group, the PD-1 inhibitor was administered at a dose of 10 mg/kg [16]. Once KP mice were diagnosed with lung cancer at 8 weeks of age, treatment commenced immediately. The treatment regimen followed the previously described protocol. In addition, a cohort of tumor-bearing mice in both the treatment and control groups was monitored for survival and subjected to comparative analysis.

### In vivo immune cell depletion assay

To investigate the potential anti-tumor mechanism of HVEM-Fc, a Lewis subcutaneous tumor formation model and immune cell depletion experiments were employed. On the day prior to tumor cell injection, mice received intraperitoneal injections of the following antibodies: 250 µg anti-CD3 (BE0002, BioXCell), 250 µg anti-CD4 (BE0003-1, BioXCell), 250 µg anti-CD8 (BE0061, BioXCell), 200 µg anti-NK1.1 (BE0036, BioXCell), and 250 µg anti-CD25 (BE0012, BioXCell). This regimen of immune cell depletions was repeated six times throughout the tumor growth period [17]. To deplete macrophages, 0.2 mL clodronate was administered intraperitoneally weekly during the course of the experiment [18].

### Flow cytometry analysis of immune cells in spleen, blood and lung tumor tissue

Flow cytometry was employed to investigate the immune cell composition within the spleen, blood, and lung tumor tissues of mice. Briefly, single-cell suspensions were prepared from fresh spleen and lung tumor tissues. These suspensions, along with blood samples, were stained with antibodies according to two distinct panels: Panel 1: CD3 APC-Cy7, CD8 Percp-Cy5.5, CD4 PE-Cy7, Foxp3 APC, CD25 PE, NK1.1 FITC, CD45 AF700; Panel 2: CD45 APC-Cy7, CD19 APC, CD11b FITC, CD11c PE-Cy7, CD86 BV421, CD163 PE-Cy7, F4/80 Percp-Cy5.5 (all antibodies sourced from Elabscience, China). The subsequent analysis followed the procedures outlined above.

### Establishment of human NSCLC organoids

This research was approved by the Ethics Committee of Huadong Hospital (2023K096). Two patients, one with lung carcinoma and one with lung squamous cell carcinoma, were randomly selected. After obtaining written informed consent from both patients, small fragments of cancer tissue (approximately 10 × 10 × 8 mm) were excised from their respective lung cancer specimens during surgery. To minimize tumor tissue contamination and protein degradation, the cancer tissues were washed three times with sterile saline. In addition, peripheral blood was collected from each patient, and T cells were isolated using density gradient centrifugation. The tumor tissues were placed in primary tissue storage solution (Daxiang, Beijing, China) and transported to the laboratory at 4 °C within 2–3 h for subsequent primary cell isolations. The digested tissue cell suspension was filtered through a 100 µm cell strainer (Falcon), and trypan blue staining was employed to assess cell viability. If cell viability exceeded 80%, the cell suspension was centrifuged at 200 × g for 3 min at low temperature. The cells were then resuspended in Matrigel on ice and inoculated into a 24-well plate. Once the Matrigel solidified, lung cancer organoid culture medium (Precedo, Hefei, China) was added to the 24-well plate. Organoid growth was monitored after 3 days. Successful organoid construction was confirmed in cultures that maintained viability beyond passage 4.

### Co-culture of three-dimensional (3D) NSCLC organoid and human T cells

To assess the anti-tumor efficacy of HVEM-Fc, an integrated Biomimetic Array Chip (iBAC-O2, TC10030A, Daxiang Biotech) was employed. This platform enabled the co-cultivation of 3D NSCLC organoids with human peripheral blood mononuclear cells (PBMCs) [19], effectively recapitulating the complex tumor immune microenvironment of human NSCLC and facilitating personalized precision medicine based on the chip data. Briefly, NSCLC organoids were digested and seeded into the iBAC-O2 chip. Following a 4-day incubation with lung cancer organoid culture medium, cell tracker deep red-labeled human T cells were co-cultured with the organoids at a 3:1 ratio (T cells: 4500; organoids: 1500). Three different concentrations of HVEM-Fc (0.08 μg/mL, 0.8 μg/mL, 8 μg/mL) and Caspase3 Green Detection Reagent were added to the co-culture system. Fluorescence imaging was acquired at 0 h, 72 h, 96 h, and 120 h. The experimental groups included HVEM-Fc, positive control (anti-CD3/28), negative control (IgG1), and an organoid-only blank group. Granzyme levels in the cell supernatant were subsequently quantified using an enzyme-linked immunosorbent assay (ELISA). Following the co-culture experiment, T cells were collected for further downstream analyses.

### Hematoxylin–eosin (HE) staining and Immunohistochemistry (IHC)

Organoids and primary lung cancer tissues were fixed in 4% paraformaldehyde for 24 h, followed by immersion in 75% ethanol and subsequent paraffin embedding. IHC and HE staining were performed to assess and compare the morphological and immunohistochemical characteristics between organoids and primary lung cancer tissues. The detailed methodologies for H&E and IHC staining have been previously described [20]. For IHC, antibodies targeting TP63 (D225020-0025; BBI), TTF1 (34,742; Novus), Napsin A (54,383; Novus), and Ki67 (89,717; Novus) were employed.

### Bulk RNA sequencing

To detect transcriptomic changes during HVEM-Fc treatment, RNA sequencing was performed on both the HVEM-Fc treatment group and the control group. Total RNA was extracted from six samples, comprising three lung cancer tissues from the control group and three from the treatment group, using the RNeasy Micro Kit (74,004; QIAGEN). Following quality control, mRNA sequencing libraries were constructed using the VAHTS Stranded mRNA-seq Library Prep Kit (NR602, Vazyme) and subsequently subjected to sequencing on the Illumina platform.

### Single-cell RNA sequencing (scRNA sequencing)

Fourteen samples, comprising twelve lung cancer tissues (divided between treatment and control groups) and two peripheral blood specimens from the treatment group, underwent 10 × Genomics scRNA-seq analysis. Tissues were immediately transferred on ice to culture dishes containing 10 ml of Dulbecco's Phosphate-Buffered Saline (14,200,075, Gibco) and subsequently minced. Following 45 min of shaking at 60 rpm, lung cancer tissues were dissociated at 35 °C. For blood samples, a minimum of 1 ml was collected in anticoagulant tubes and promptly transported to the laboratory on ice. PBMCs were isolated using density gradient centrifugation (LTS1077, TBD science). Post-dissociation, the viability of all cells was assessed microscopically. As per the manufacturer's instructions, scRNA-seq libraries were constructed using the 10 × Genomics Single Cell Gene Expression Solution V3.1 and subsequently sequenced on an Illumina NovaSeq 6000 platform at a read depth of 80 bases.

### Flow cytometry analysis of markers of T cell activation

Following the co-culture of patient-derived organoids with T cells, the T cells were carefully collected. A washing step using phosphate-buffered saline (PBS) was performed to remove any residual media or non-adherent cells. To assess CD69 and CD25 expression, the cells were incubated with antibodies specific for CD69 and CD25 (BioLegend, San Diego, CA, USA).

### Spatial transcriptomics (ST)

Six samples, comprising three lung cancer tissues from the treatment group and three from the control group, were subjected to ST analysis using the 10 × Genomics Visium Platform. Fresh lung tissues from mice were dissected into pieces with an area less than 6.5 mm × 6.5 mm, following the tissue preparation instructions. After meticulous removal of blood and contaminants, each tissue specimen was embedded in OCT (SAKURA, 4583) and stored at −80 °C. Subsequently, tissue sections with a thickness of 8 µm were prepared to capture the designated areas for both spatial tissue optimization and gene expression analysis on the 10 × Genomics Visium Spatial Gene Expression slides (PN-1000184). Library construction was performed adhering to the 10 × Genomics user guide (CG000239). Finally, the pooled libraries were sequenced on an Illumina NovaSeq 6000 instrument using 150-bp paired-end reads.

### Cytokine detection in mouse serum and tumor

We conducted the RayBio C-Series Mouse Cytokine Antibody Array C1 to detect the expression level of cytokines. Following overnight incubation of mouse serum and lysed tumor tissues with the array at 4 °C, membrane scanning was performed using an ImageQuant LAS4000 Scanner. Subsequent analysis of relative intensities against a control group was conducted utilizing Excel-based Analysis Software. Finally, Gene Ontology (GO) and Kyoto Encyclopedia of Genes and Genomes (KEGG) pathway analyses were performed using the R programming language based on the differentially expressed proteins identified.

## Results

### Structural characterization of the HVEM-Fc protein

**Fig. 1:**
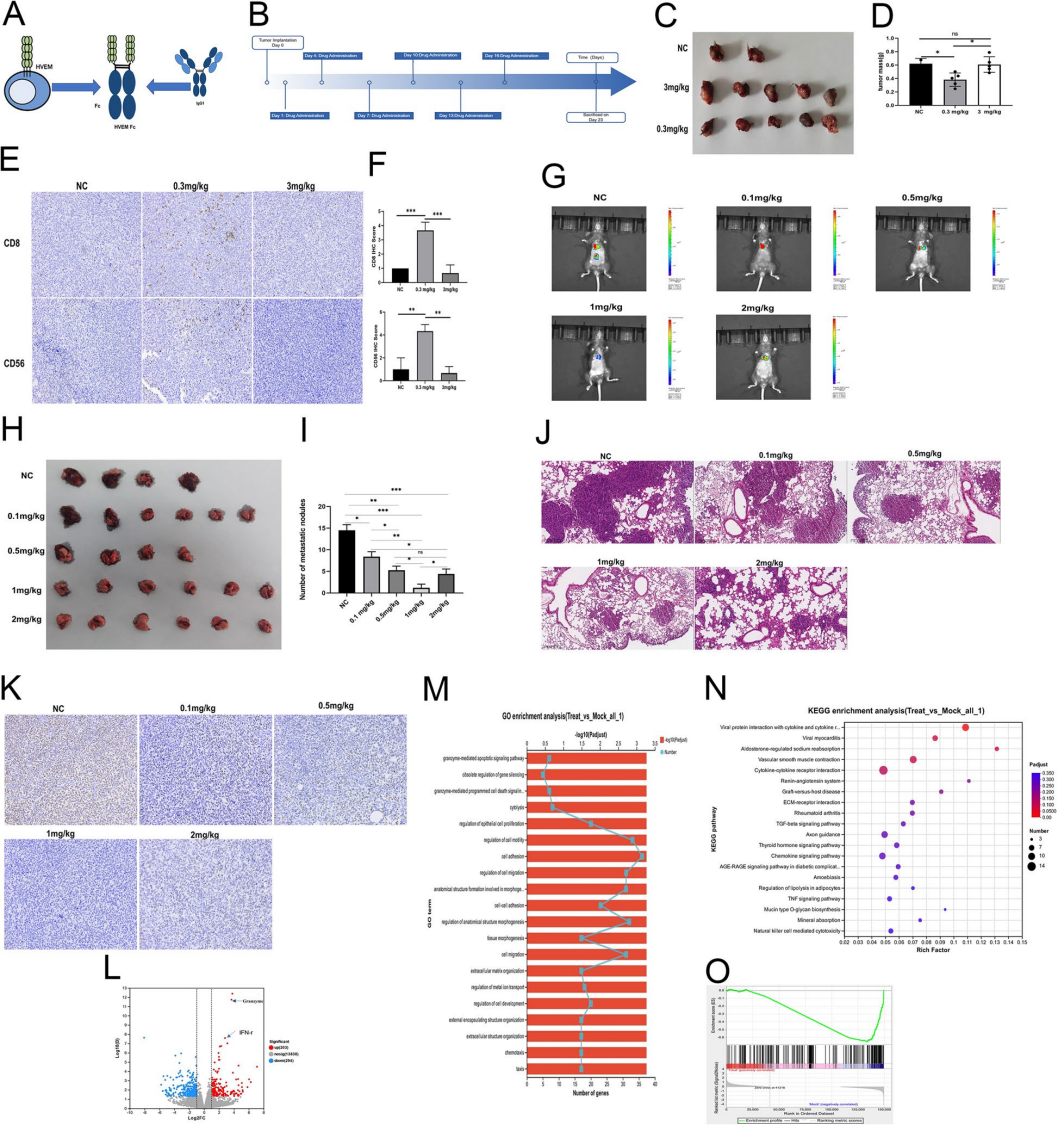
1 mg/kg HVEM-Fc yielded the strongest tumor-inhibitory effect. Notes: **A** The detailed structure of HVEM-Fc; **B** The treatment plans for mouse lung cancer models; **C-D** Low dose of HVEM-Fc had the potential of anti-tumor function; **E–F** IHC of CD8 and CD56 in mouse tumor; **G-K** the optimal dose of HVEM-Fc was explored; **L-O** The results of RNA sequencing. The image captured at 200 × magnification

Recombinant HVEM-Fc fusion proteins, encompassing the extracellular domain of HVEM (Gln39-Val207; mouse: GenBank NP_849262, human: GenBank AAQ89238) fused to the C-terminus of human IgG1 Fc, were generated for both mouse and human sequences. The structural organization of these constructs is depicted in Fig. 1A. It was hypothesized that the HVEM domain could mediate interactions with its cognate receptor(s), while the Fc domain could facilitate binding to Fc receptors. Expressed in HEK-293 cells, the resulting HVEM-Fc proteins exhibited a molecular weight of approximately 55 kDa.

### In vivo anti*-*neoplastic potential of low-dose HVEM-Fc

Figure 1B illustrates the experimental design for the mouse lung cancer model, including tumor implantation and treatment schedules. To investigate the potential anti-tumor effect of HVEM-Fc, immunocompetent *C57BL/6* mice bearing Lewis lung carcinoma cells implanted subcutaneously were used. Since there is no existing literature regarding the HVEM-Fc protein, the high dose (3 mg/kg) and the low dose (0.3 mg/kg) were chosen based on existing studies investigating novel anti-4-1BB antibodies [7]. In addition, a mouse IgG1 Fc antibody served as the control. Notably, we observed that a low dose of HVEM-Fc effectively killed tumor cells, whereas no such effect was evident in the high-dose HVEM-Fc group. Indeed, tumors in the high-dose group were slightly larger than those in the control group (Fig. 1C-D). We hypothesized that the anti-tumor effect of HVEM-Fc is primarily mediated by the immune system, with NK and CD8^+^ T cells acting as the principal effector cells. IHC staining was performed to assess immune cell infiltration within the subcutaneous tumors. The results indicated that the low-dose group exhibited higher levels of CD8^+^ T and CD56^+^ NK cells compared to the control group. In contrast, no significant difference in CD8^+^ T or NK cell levels was observed between the high-dose and control groups (Fig. 1E-F). These findings demonstrate that low-dose HVEM-Fc possesses anti-tumor potential.

### 1 mg/kg HVEM-Fc yielded the most potent tumor-inhibitory effect

To determine the optimal dose of HVEM-Fc for the LLC-Luc lung cancer model, we performed a dose escalation study. LLC-LUC cells were implanted into the tail vein of mice, and then mice were injected with escalating doses of HVEM-Fc (0.1 mg/kg, 0.5 mg/kg, 1 mg/kg and 2 mg/kg). All mice received six doses. Our data from live animal imaging, gross lung specimens, HE staining, and Ki67 scores indicated that 1 mg/kg HVEM-Fc yielded the strongest inhibitory effect on tumor growth. 0.5 mg/kg and 2 mg/kg HVEM-Fc resulted in similar tumor inhibition effects, and even the 0.1 mg/kg dose showed significant tumor suppression compared to the control group (Fig. 1G-K). It is well-established that both peripheral systemic immunity and the tumor immune microenvironment are critical for durable immunotherapy responses [21–23]. Therefore, we used flow cytometry to analyze immune cell subsets in the peripheral blood and lung tumors of these mice. The results revealed a significant expansion of CD3^+^ and CD4^+^ T cells in the blood after treatment with 1 mg/kg HVEM-Fc. Additionally, the ratio of pro-inflammatory M1 macrophages to anti-inflammatory M2 macrophages was altered following treatment with the 1 mg/kg dose. Furthermore, we observed increased infiltration of CD3^+^, CD4^+^, CD8^+^ T cells, and NK cells into the tumor microenvironment, along with a decrease in overall macrophage density. Notably, tumor PD-L1 expression levels remained unchanged after HVEM-Fc treatment (Figure S1-2). The gating strategy for immune cell identification is provided in Supplementary Files 1 and 2. We next investigated cytokine expression levels in the blood of HVEM-Fc-treated mice compared to controls. This analysis revealed changes in several immune-related cytokines (Supplementary File 3). Functional enrichment analysis of these differentially expressed proteins indicated significant enrichment of various immune cell-specific signaling pathways and pro-inflammatory pathways (Figure S3A-B). These findings collectively suggest that HVEM-Fc treatment can induce an immune-stimulatory tumor microenvironment, with the 1 mg/kg dose demonstrating the most potent anti-tumor efficacy. To further validate these observations, we performed bulk RNA sequencing on lung tumors treated with 1 mg/kg HVEM-Fc and their corresponding untreated controls. Differential gene expression was defined as a *p*-value < 0.05 and a log2 fold-change (FC) > 0.5. Based on these criteria, we identified a total of 497 differentially expressed genes, including 203 up-regulated genes and 294 down-regulated genes (Fig. 1L, Supplementary File 4). The top five up-regulated genes belonged to the Perforin and Granzyme family, which are primarily involved in the cytolytic activity of immune cells against cancer cells. GO and KEGG pathway analysis of these differentially expressed genes revealed enrichment of terms such as granzyme-mediated cell death signaling, regulation of cell migration, and immune processes (Fig. 1M-N). Gene Set Enrichment Analysis (GSEA) identified the positive regulation of the cytotoxic T cell differentiation pathway (GO:0045065) as a significantly enriched term, suggesting activation of CD8^+^ cytotoxic T cells in the HVEM-Fc-treated group (Fig. 1O). Analysis of gene interactions revealed significant enrichment of pathways related to granzyme-mediated apoptosis, granzyme-mediated programmed cell death, and regulation of epithelial cell proliferation, further supporting the role of immune cells in mediating tumor cell attack(Figure S3C). However, since bulk RNA sequencing cannot distinguish between cancer cells and other immune cell populations, we subsequently employed single-cell RNA sequencing for more detailed analysis.

### HVEM-Fc suppresses CMT64-induced lung tumorigenesis

**Fig. 2:**
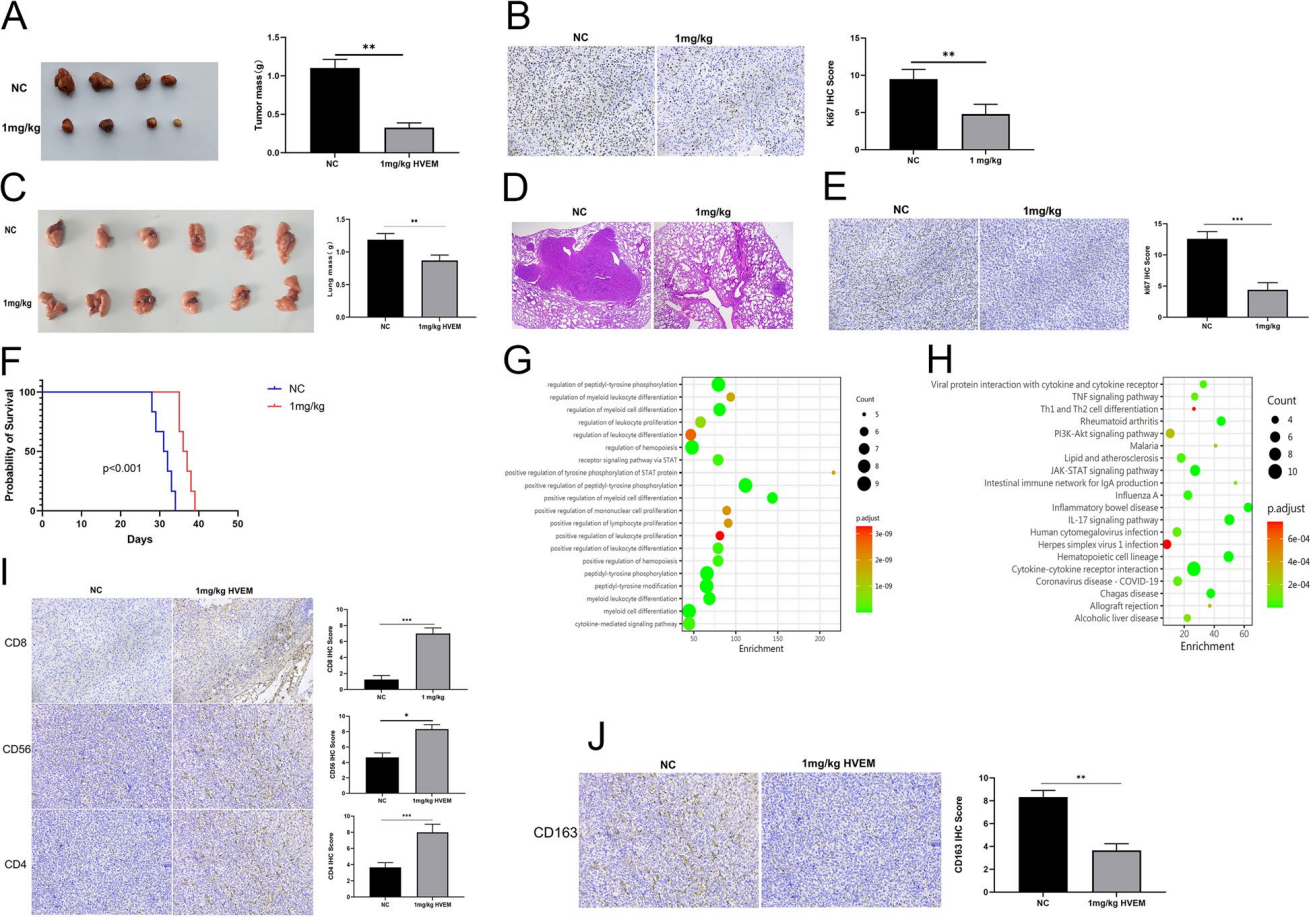
1 mg/kg HVEM-Fc could inhibit tumor formation in CMT64 cells induced mouse lung cancer. Notes: **A-B** 1 mg/kg HVEM-Fc could effectively inhibit the tumor growth in mouse subcutaneous tumor model; **C-F** 1 mg/kg HVEM-Fc suppressed the lung metastasis and prolonged the survival in the tail vein metastasis model; **G-H** GO and KEGG analysis found that immune cell activation and immune associated pathways were highly enriched; **I** The scores of NK cells, CD4 T and CD8 T cells; **J** The scores of CD163 cells. The image captured at 200 × magnification

To further validate our findings, we investigated the efficacy of HVEM-Fc in a mouse model of lung cancer using the CMT-64 cell line. CMT-64 is characterized by low antigenicity and resistance to PD-1 inhibitors [24, 25], making it a challenging model for immunotherapy. Consistent with our previous observations in the LLC-Luc model, 1 mg/kg HVEM-Fc effectively inhibited tumor growth in a subcutaneous mouse model (Fig. 2A-B). In addition, HVEM-Fc significantly suppressed lung metastasis and prolonged survival in a tail vein metastasis model (Fig. 2C-F). To understand the underlying mechanisms, we analyzed cytokine expression levels in lung tumors from mice treated with 1 mg/kg HVEM-Fc compared to untreated controls. The results revealed significant upregulation of IFN-γ and TNF-α, pro-inflammatory cytokines known to play crucial roles in anti-tumor immunity. In contrast, VEGF and IL-6, which are associated with poor prognosis, were downregulated in the tumors from HVEM-Fc-treated mice (Supplementary File 5). Functional analysis indicated significant enrichment of immune cell activation and immune-associated pathways, suggesting robust anti-tumor immune responses in the treated mice (Fig. 2G-H). To further investigate the immune cell infiltration within the tumors, we performed IHC staining for T cells, NK cells, and M2 macrophages. The results showed significantly higher scores for NK cells, CD4^+^ T cells, and CD8^+^ T cells in the tumors from HVEM-Fc-treated mice compared to the control group (Fig. 2I). Conversely, the scores for CD163^+^ M2 macrophages, which are associated with immunosuppression, were significantly lower in the treated group (Fig. 2J). These findings collectively demonstrate that HVEM-Fc treatment enhances T-cell and NK cell-mediated tumor cell killing in the CMT-64 mouse model of lung cancer.

### The combination of HVEM-Fc and PD-1 inhibitor decreased tumor growth and prolonged the survival of C57BL6 and KP mice

**Fig. 3:**
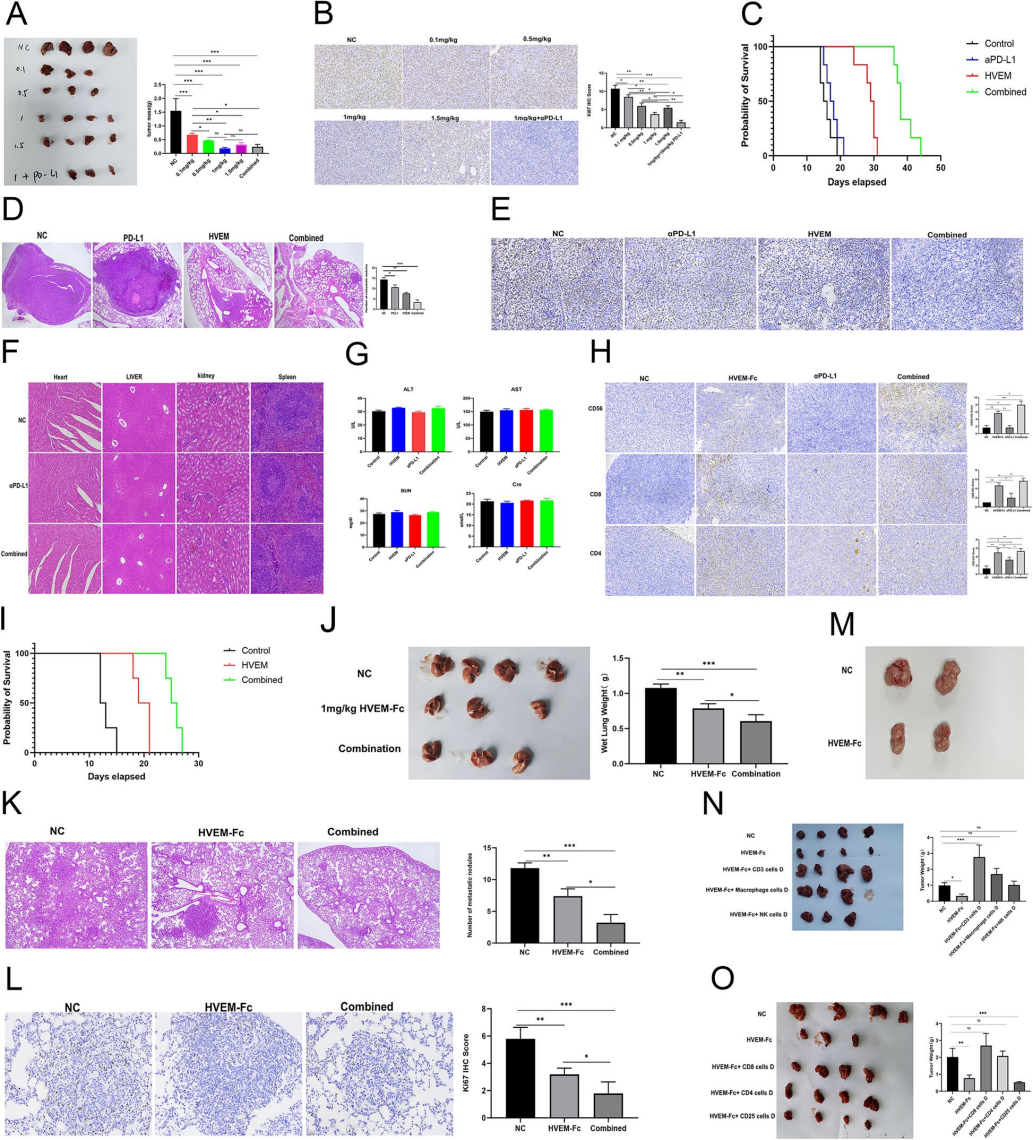
The combination of HVEM-Fc and PD-1 inhibitor decreased the tumor growth and extended the survival of C57BL6 and KP mice. Notes: **A-B** The treatment effects were more pronounced in the drug combination group in subcutaneous tumor-bearing mouse model; **C-E** The treatment effects were more pronounced in the drug combination group in tail vein tumor-bearing mouse model; **F**-**G** No obvious drug associated damage was observed in the drug combination group; **H** The scores of NK cells, CD4 T and CD8 T cells;** I** The drug combination effectively prolonged the survival of genetically engineered mouse models; **J-L** Ki67 index, HE of pulmonary tissues and dry lung weight confirmed our results; **M** Subsequent tumor challenge was effectively controlled by a robust and rapid immunological memory T cell response elicited by prior HVEM-Fc treatment; **N–O **The immune cells depletion antibodies were used. The image captured at 200 × magnification

Currently, PD-1/PD-L1 blockade offers promising outcomes in many solid cancers. However, only a proportion of patients benefit from this treatment [26]. Given that previous studies demonstrated that HVEM-Fc does not alter PD-L1 protein expression, we investigated the potential synergistic effects of combining HVEM-Fc with a PD-1 inhibitor in two distinct mouse lung cancer models. In the first model, subcutaneous Lewis lung carcinoma was established in mice. We evaluated different doses of HVEM-Fc (0.1 mg/kg, 0.5 mg/kg, 1 mg/kg, 1.5 mg/kg) and a combination of 1 mg/kg HVEM-Fc with 10 mg/kg of a PD-1 inhibitor. The results revealed that both the 1 mg/kg HVEM-Fc group and the combination group exhibited the lowest tumor weights (Fig. 3A). While no significant difference in tumor mass was observed between these two groups, the Ki67 index, a marker of cell proliferation, was significantly lower in the combination group compared to all other groups (Fig. 3B). These findings suggest that the combination therapy may exert a more pronounced anti-tumor effect.

Moreover, the efficacy of 1 mg/kg HVEM-Fc, 10 mg/kg PD-1 inhibitor, the combination of 1 mg/kg HVEM-Fc with 10 mg/kg PD-1 inhibitor, and a control group were evaluated in a mouse tail vein metastasis model. Survival analysis demonstrated that the combination treatment group exhibited the most favorable prognosis, followed by the 1 mg/kg HVEM-Fc group. The PD-1 inhibitor-treated group and the control group showed comparable survival outcomes **(**Fig. 3C**)**. These findings were further supported by the Ki67 proliferation index and histological examination of pulmonary tissues (Fig. 3D-E**)**. Histopathological examination of heart, liver, spleen, and kidney tissues from all four groups revealed no apparent drug-related toxicity (Fig. 3F). Similarly, blood biochemistry analyses, including alanine aminotransferase (ALT), aspartate aminotransferase (AST), blood urea nitrogen (BUN), and creatinine levels, showed no significant abnormalities in any group (Fig. 3G). Immunohistochemical analysis of tumor tissues revealed significantly higher scores for NK cells, CD4^+^ T cells, and CD8^+^ T cells in the combination treatment group compared to the other groups (Fig. 3H). These results collectively indicate that the combined treatment of HVEM-Fc and the PD-1 inhibitor exhibits the most potent anti-tumor effect with relatively lower toxicity compared to either single agent alone.

Mouse lung cancer cell lines were utilized in the aforementioned in vivo experiments. While genetically engineered mouse models have significantly advanced our understanding of cancer pathogenesis and facilitated the development of novel immune checkpoint inhibitors, we sought to further validate the therapeutic efficacy of HVEM-Fc monotherapy and the combination of HVEM-Fc with a PD-1 inhibitor in a more physiologically relevant setting. To this end, we employed the spontaneous KP-GEMM (LSL-Kras^G12D/+^; LSL-p53R172H/ +) mouse model of lung adenocarcinoma, which accurately recapitulates the complexity of human lung adenocarcinoma in vivo. Upon confirmation of lung cancer development in the KP mice, treatment with the designated therapies was initiated (Figure S3D). As expected, combination therapy significantly prolonged survival compared to the control group and HVEM-Fc monotherapy **(**Fig. 3I, p < *0.05)*. Furthermore, assessment of the Ki67 proliferation index, histological examination of pulmonary tissues, and measurement of dry lung weight demonstrated that the combination therapy effectively elicited robust anti-tumor effects **(**Fig. 3J-L**)**.

### HVEM-Fc-based treatment elicits a robust anti-tumor immune memory, facilitating secondary tumor prevention

In our previous experiments, 1 mg/kg HVEM-Fc, both alone or in combination with anti-PD-1 therapy, demonstrated the ability to extend the survival of mice bearing lung cancer tumors. Based on these findings, we hypothesized that 1 mg/kg HVEM-Fc treatment, with or without anti-PD-1, could induce long-term anti-tumor immune memory. To validate this hypothesis, we utilized two Lewis lung cancer cell-induced tail vein injection models that achieved complete remission following treatment with HVEM-Fc alone (without anti-PD-1). We first confirmed the complete disappearance of tumors in these mice using IVIS imaging 21 days after the initial tumor implantation (Figure S3E). Subsequently, these mice were re-challenged with a subcutaneous implantation of Lewis lung cancer cells. No further treatment was administered to these mice. The tumor growth in these re-challenged mice was then compared to that observed in age-matched C57BL/6 control mice that had received only control antibody treatment. Our results demonstrated that tumor growth in the re-challenged mice was significantly suppressed, with smaller and lighter tumors compared to the control group. These findings suggest that the re-challenge tumors were effectively controlled by a rapid and robust immunological memory T-cell response elicited by the prior HVEM-Fc treatment (Fig. 3M).

### Antitumor Activity of HVEM-Fc is Mediated by CD8 + and CD4 + T Cells, with Partial Contributions from NK Cells and Macrophages

To comprehensively explore the immune cell populations correlated with HVEM-Fc therapy, we first depleted CD3^+^ T cells, macrophages, or NK cells after receiving HVEM-Fc treatment in a Lewis lung carcinoma subcutaneous tumor-forming mouse model. Depletion of CD3^+^ T cells, NK cells, and macrophages was confirmed by flow cytometry in the peripheral blood and tumor tissue of tumor-bearing mice. No immune cells were depleted in the control group. Representative flow cytometry plots are presented in Supplementary Files 6 and 7. The depletion of CD3^+^ T cells completely reversed the antitumor response, indicating that T cells mediate the therapeutic effect of the antitumor immune response. Furthermore, NK1.1 antibody depletion and macrophage depletion resulted in increased tumor growth compared to the HVEM-Fc treatment group, suggesting that NK cells and macrophages also contribute to the antitumor immune response (Fig. 3N). Considering that NK cell-mediated antibody-dependent cell-mediated cytotoxicity (ADCC) and macrophage-mediated antibody-dependent cell-phagocytosis (ADCP) are triggered by interactions between the Fc domain and Fcγ receptors, we hypothesized that T cell-mediated immune responses might be the most crucial [27]. Cell surface markers primarily divide T cells into CD4^+^ T helper cells, CD8^+^ cytotoxic T lymphocytes (CTLs), and CD4^+^CD25^+^ regulatory T cell (Treg) populations. To assess the specific T cell subpopulations involved in the mechanism of HVEM-Fc treatment, we further depleted CD4^+^ T cells, CD8^+^ T cells, or CD25^+^ Tregs in tumor-bearing mice receiving HVEM-Fc treatment. Compared to the control group, the depletion of CD8^+^ T cells and CD4^+^ T cells both significantly influenced the therapeutic effect of HVEM-Fc, suggesting that both CD8^+^ T cells and CD4^+^ T cells play crucial roles. Notably, we observed that CD25^+^ Tregs depletion further enhanced the antitumor effect, indicating that eradication of CD25^+^ Tregs in combination with HVEM-Fc treatment could improve therapeutic efficacy (Fig. 3O). Our analysis demonstrated that both CD4^+^ and CD8^+^ T cell subpopulations are indispensable for mediating tumor elimination in tumor-bearing mice receiving HVEM-Fc treatment.

Finally, to investigate whether the antitumor effects of HVEM-Fc depended on immune system mobilization, we utilized immune-compromised NOD-scid IL2Rγ-knockout (NSG) mice to establish tail vein tumor-forming models. The results demonstrated that 1 mg/kg HVEM-Fc had no significant impact on tumor growth in tumor-bearing mice, suggesting that HVEM-Fc relies on the immune system to exert its antitumor effects (Figure S4A-C). Next, to determine whether HVEM-Fc exhibits anticancer effects independent of T cells in vitro, lung adenocarcinoma cell line A549 and lung squamous cell line H520 cells were treated with 0.08 μg/mL, 0.8 μg/mL, and 8 μg/mL HVEM-Fc for 72 h, followed by assessment of cell viability using the CCK8 assay. As expected, HVEM-Fc had no significant effect on the proliferation of NSCLC cells (Figure S4D).

### HVEM-Fc could eradicate tumor cells by activating T cells in NSCLC organoids and T cell co-culture models

**Fig. 4:**
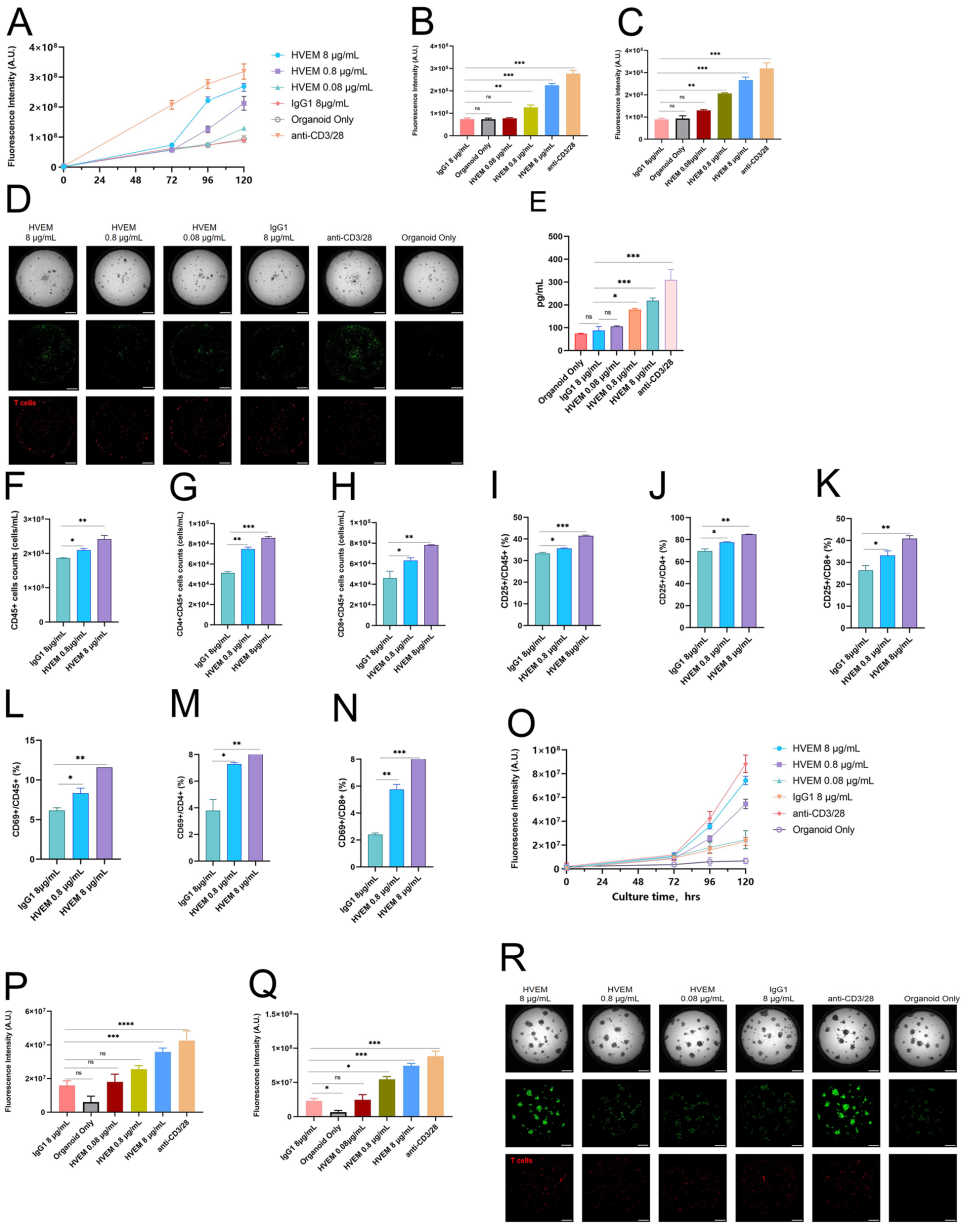
HVEM-Fc could eradicate the tumor cells by activating T cells in NSCLC organoids and T cell co-culture models. Notes: **A-C** The 8 μg/mL HVEM-Fc group yielded the strongest anti-tumor effect and the anti-tumor effect was more pronounced at 5 days; **D** At 120 h, bright field and fluorescent images were taken; **E** ELISA detection of perforin in the supernatants of cell culture medium; **F-N** T cell proliferation and activation were analyzed through flow cytometry; **O-R** The 8 μg/mL HVEM-Fc group had the strongest anti-tumor effect in lung adenocarcinoma organoids and T cell co-culture model

Patient-derived organoids (PDOs) are models for predicting the efficacy of treatment for personalized precision medicine [28]. Recently, the development of NSCLC patient-derived tumor organoid-immune co-culture models holds promise for exploring specific therapeutic medicines and enhancing the understanding of tumor-immune cell interactions [29]. Based on these findings, two NSCLC patient-derived organoid models co-cultured with T cells were constructed to assess the underlying anti-tumor effect of HVEM-Fc. Two patients who underwent curative surgeries were randomly selected: one diagnosed with lung squamous cell carcinoma and the other with lung adenocarcinoma. Baseline parameters and preoperative CT scans are presented in Figure S5A-B and Table S1. Tumor tissues were obtained from freshly resected lung lobes, and T cells were derived from the PBMCs of the corresponding patients. First, we investigated whether the PDOs accurately reflected the histological features of the NSCLC tissues. HE staining and IHC staining demonstrated a high degree of concordance between the two PDOs and the primary lung cancer tissues (Figure S5C-H: Case 1: C-E, Case 2: F–H). Next, flow cytometric analysis was used to characterize the subpopulations of PBMC cells. The detailed proportions of T cells are listed in Table S2. In the PDO1 and T cell co-culture model, the anti-CD3/CD28 positive control group exhibited the strongest ability to kill tumor cells. Among the drug-treated groups, the 8 µg/mL HVEM-Fc group demonstrated the most potent anti-tumor effect compared to the IgG1 control and the organoid-only group (Fig. 4A). We observed that the anti-tumor effect of HVEM-Fc became apparent at 4 days post-co-cultivation (Fig. 4B) and was more pronounced at 5 days (Fig. 4C). Bright-field and fluorescent images were acquired at 120 h (Fig. 4D) and at 0 and 72 h (Figure S5I-J). Due to the short half-life of the dying material, it was challenging to assess the anti-tumor effect beyond 5 days. Considering the potential for long-term anti-tumor memory, we hypothesized that HVEM-Fc could exert its anti-tumor effect over an extended period. ELISA was performed to measure perforin levels in the supernatants of the cell culture medium. The level of perforin in the 8 µg/mL and 0.8 µg/mL HVEM-Fc groups was significantly higher than in the negative control group, suggesting the stimulation and activation of T cells (Fig. 4E). CD69 and CD25 are recognized as early and late markers of T cell activation, respectively [30]. T cell proliferation and activation were analyzed by flow cytometry. An increase in the number of CD45^+^ cells, CD4^+^CD45^+^ cells, and CD8^+^CD45^+^ cells was observed in the HVEM 8 µg/mL group compared to the other groups. Furthermore, the ratios of CD25^+^/CD45^+^, CD25^+^/CD4^+^, CD25^+^/CD8^+^, CD69^+^/CD45^+^, CD69^+^/CD4^+^, and CD69^+^/CD8^+^ in the HVEM 8 µg/mL group were significantly increased compared to the other groups (Fig. 4F-N). In the PDO2 model, we observed similar phenomena (Fig. 4O-Q). As with PDO1, the anti-tumor effect of HVEM-Fc became apparent at 4 days post-co-cultivation. Bright-field and fluorescent images were acquired at 120 h (Fig. 4R)and at 0 and 72 h (Figure S5K-L).

Based on the above results, HVEM-Fc may exert its anti-tumor function in treating NSCLC patients by activating T cells. However, a major limitation of this study is the need to analyze more patient-derived organoids to validate our findings.

### HVEM-Fc altered the immune landscape of the TME

**Fig. 5:**
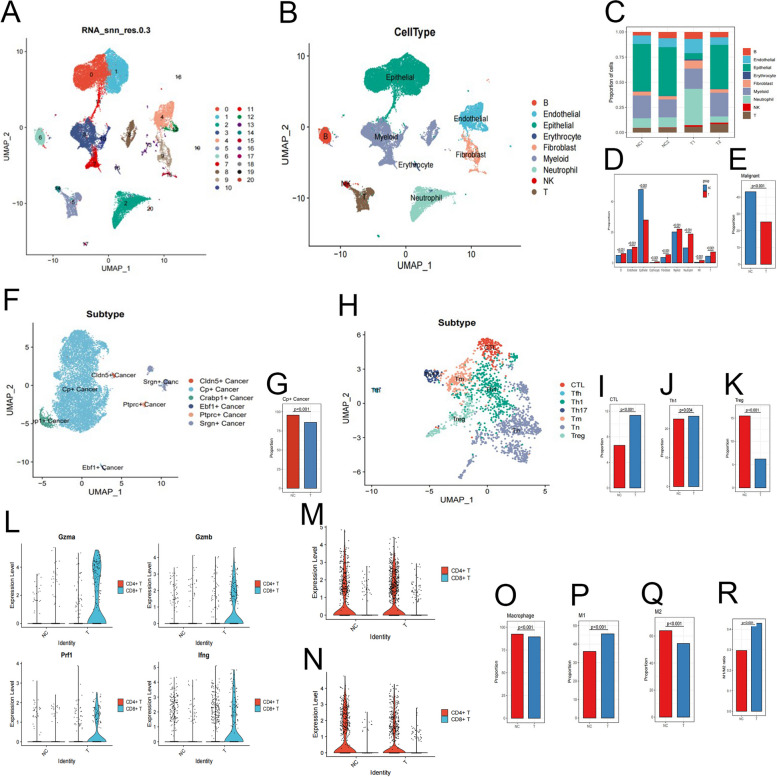
The results of single-cell RNA-sequencing. Notes:** A** UMAP plot showing cells from samples; **B** UMAP plot showing cells grouped into nine major cell types; **C** The proportion of major cell types; **D** Comparison of the proportion for each major cell type between two groups; **E** The epithelial cells harboring higher CNV levels were prominently reduced compared with the NC group; **F** The subtypes of cancer cells;** G** Cp^+^ cancer cells were significantly decreased after treatment; **H** The subtypes of T cells; **I-K** The proportional of CTL and CD4 Th1 cells significantly increased after HVEM-Fc treatment compared with the control group, While the immunosuppressive population of Tregs decreased due to the treatment; **L** Cytolytic enzymes secreted by CTLs and CD4 cells increased after treatment; **M** The subtypes of T cells; **I-K** The proportion of CTLs and CD4 Th1 cells significantly increased after HVEM-Fc treatment compared with the control group, While the immunosuppressive population of Treg cells decreased due to the treatment; **L** Cytolytic enzymes secreted by CTLs and CD4 cells increased after treatment; **M–N** The terminally exhausted phenotype indictor LAG3 and Havcr2 in CD4T decreased after treatment; **O-R** The abundance of macrophages decreased, while the treatment group showed an increase in the M1 macrophage phenotype, while the M2 macrophage phenotype decreased

To further elucidate the influence and potential mechanisms of HVEM-Fc on the tumor immune landscape, lung tumors resected from Lewis lung carcinoma tail vein injection mouse models were subjected to single-cell RNA sequencing (scRNA-seq). In addition, peripheral blood was isolated from HVEM-Fc-treated mice for scRNA-seq analysis. Following quality control (200 < nFeatures < 9000, 300 < nCount < 50,000; Figure S6A), a total of 34,818 cells were retained for further analysis, derived from the IgG-treated group (negative control) and the HVEM-Fc-treated group (treatment group). After re-clustering and dimensionality reduction, nine distinct cell clusters were identified, comprising epithelial, B, endothelial, erythrocyte, fibroblast, T, B, NK, and myeloid cells, based on their representative gene signatures (Fig. 5A-B). Chi-square tests were performed to analyze the differences in cell lineage proportions between the negative control and treatment groups. The results revealed significant shifts in the proportions of various cell lineages in the two groups (Fig. 5C), indicating that HVEM-Fc treatment altered the cellular composition of the tumor microenvironment. Notably, the proportion of epithelial cells was significantly decreased after treatment, while the fractions of other cell types were relatively increased (Fig. 5D). Next, copy number variation (CNV) analysis was performed using CopyKAT to distinguish tumor cells from normal lung epithelial cells [31]. Consistent with previous studies, epithelial cells exhibiting higher CNV levels, considered to be malignant cells, were also significantly reduced compared to the negative control group (Fig. 5E). Clustering analysis of tumor cells revealed six subclusters exhibiting transcriptional heterogeneity, including Cp^+^, Cldn5^+^, Crabp1^+^, Ebf1^+^, Ptprc^+^, and Srgn^+^ tumor cells (Fig. 5F). Cp^+^ cancer cells, which constituted the majority of tumor cells, were significantly decreased after treatment, suggesting that Cp^+^ cell populations may benefit from HVEM-Fc treatment (Fig. 5G).

**Fig. 6:**
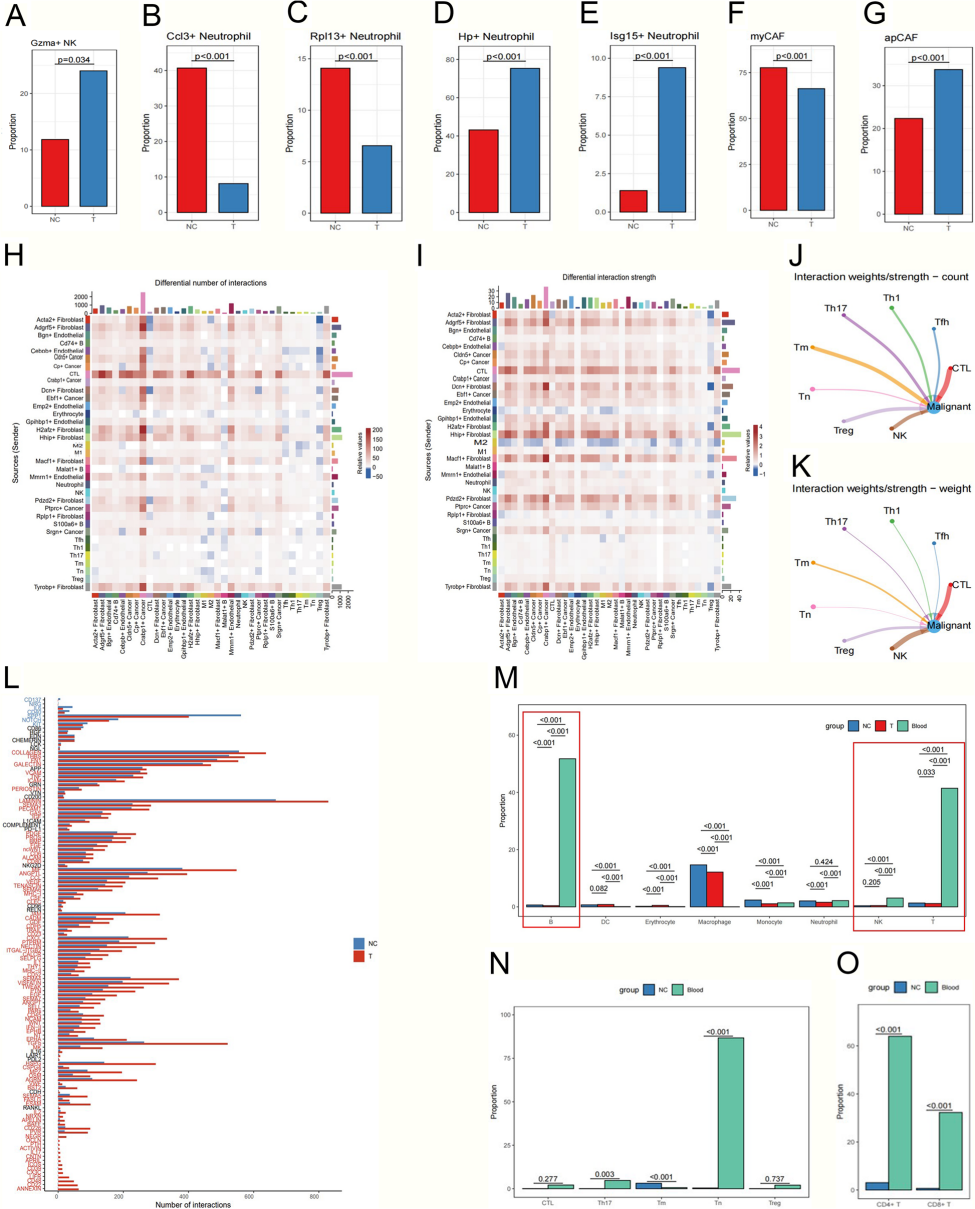
HVEM-Fc altered the immune landscape in the TME. **Notes: A** Gzma^+^ NK cell abundance was more pronounced in the treatment group; **B-E** The proportion of Hp^+^ and Isg15^+^ cells significantly increased after HVEM-Fc treatment compared with the control group, While the ccl3^+^ and Rpl13^+^ decreased after treatment; **F-G** The apCAF subpopulations increased significantly while myCAF decreased after treatment; **H-I** Heatmap demonstrating the Pearson correlation between the cell-type abundance for various immune cell types; **J-K** The HVEM-Fc treatment group showed significant interactions between CTLs, NK cells and tumor cells in the number and strength of communications; **L** The alterations in signaling pathways after HVEM-Fc treatment; **M–O** The comparison of the subsets of immune cells in the peripheral blood and lung tumors of treatment group

T cells, NK cells, and macrophages are all potential tumor-killing cells within the tumor microenvironment. Next, we investigated the dynamic changes in the subsets of these immune cells following HVEM-Fc treatment. A total of 4,718 T cells were screened and classified into CTLs, Tfh, Th1, Th17, memory T cells, naïve T cells, and Tregs. The proportions of CTLs and CD4^+^ Th1 cells significantly increased after HVEM-Fc treatment compared to the control group, while the immunosuppressive population of Tregs decreased due to the treatment (Fig. 5I-K). These results indicated that HVEM-Fc treatment could enhance durable tumor-killing effects and augment anti-tumor immune responses [32]. The identification of cytolytic enzyme expression levels (perforin, granzyme A, granzyme B, granzyme K, and IFN-γ) provided further insights into the anti-tumor functions of these T cells. The results showed that the secretion of most of these cytolytic enzymes by CTLs and CD4^+^ T cells increased after treatment (Fig. 5L). Furthermore, the expression of LAG3 and HAVCR2, markers of terminal T cell exhaustion, in CD4^+^ T cells decreased after treatment (Fig. 5M-N). Collectively, the analysis of the T cell-mediated immune landscape demonstrated that HVEM-Fc treatment shifted the immune response towards an anti-tumor phenotype characterized by CTL dominance and decreased T cell exhaustion. Myeloid cells are a heterogeneous population comprising dendritic cells (DCs), macrophages, and myeloid-derived suppressor cells (MDSCs) (FigureS6B). Myeloid-derived macrophages can be polarized into anti-tumor M1 or pro-tumor M2 phenotypes, which play crucial roles in regulating T cell responses within the tumor microenvironment [33, 34](FigureS6C). The percentage of macrophages decreased, while the treatment group showed an increase in the M1 phenotype and a decrease in the M2 phenotype, resulting in an increased M1/M2 ratio. These findings suggest that HVEM-Fc suppressed tumor invasiveness by reprogramming tumor-associated macrophages (TAMs) towards the M1 phenotype and enhancing their anti-tumor functions, thus highlighting HVEM-Fc as a promising new cancer immunotherapy agent (Fig. 5O-R).

In addition, NK cells were divided into Eomesodermin (Eomes)^+^ NK cells and Granzyme A (Gzma)^+^ NK cells (Figure S6D). Previous studies have shown that Gzma^+^ NK cells exhibit enriched granzyme and perforin signature scores, indicating that they exert the most potent anti-tumor functions [35, 36]. The proportion of Gzma + NK cells was significantly higher in the treatment group, suggesting a better prognosis and the potential for mediating tumor regression [37](Fig. 6A).

Neutrophils play a pivotal role in innate immunity within the tumor microenvironment [38]. However, due to their short half-life, their detailed functions remain controversial [39]. Next, we classified neutrophils into ccl3^+^, Hp^+^, Isg15^+^, Rpl13^+^, and Cd79a^+^ subpopulations (Figure S6E). The proportions of Hp^+^ and Isg15^+^ neutrophils significantly increased after HVEM-Fc treatment compared to the control group, while the proportions of ccl3^+^ and Rpl13^+^ neutrophils decreased after treatment (Fig. 6B-E). Isg15 belongs to the interferon-stimulated gene family, which exerts anti-tumor functions in tumor immunity [40, 41]. Our experiments revealed that Isg15^+^ neutrophils recruited to tumor sites suppressed cancer initiation and metastasis, indicating that not all tumor-associated neutrophils promote cancer growth and reflecting the plasticity of neutrophil function.

Finally, cancer-associated fibroblasts (CAFs) play a critical role in shaping the tumor immune microenvironment. CAF subtypes can secrete soluble factors, such as interleukin-6 (IL-6), TNF-α, and granulocyte-colony stimulating factor (G-SCF), that modulate the immune response, leading to either anti-tumor or pro-tumor effects [42]. Our scRNA-seq analysis identified two CAF subclusters: myofibroblastic CAFs (myCAFs) and antigen-presenting CAFs (apCAFs) **(Figure S6F)**. MyCAFs are characterized by their ability to modulate the extracellular matrix to promote immune evasion, while apCAFs are associated with increased infiltration of CD8^+^ T cells [43]. As expected, the apCAF subpopulation significantly increased, while the myCAF subpopulation decreased after treatment, suggesting an enhanced antigen-presenting capacity during treatment (Fig. 6F-G).

Notably, cell–cell communication within the negative control (NC) and treatment groups was investigated using CellChat analysis. The results demonstrated an increased number and strength of communication pathways between CTLs and tumor cells (Fig. 6H-I). Concurrently, a decrease in M2 macrophages and a slight increase in M1 macrophages were observed, accompanied by enhanced interactions between stromal cells and cancer cells. Notably, the HVEM-Fc treatment group exhibited significantly increased numbers and strengths of communication pathways between CTLs, NK cells, and tumor cells (Fig. 6J-K).

To explore alterations in several signaling pathways following HVEM-Fc treatment, we identified pathways previously reported to be associated with immunity modulation and tumor growth [44]. Compared to the negative control group, most pathways containing MHC-I, MHC-II, and IFN-γ were upregulated, while a few pathways, including IL6, Notch, and SPP1, were downregulated (Fig. 6L).

Peripheral blood was also collected from the HVEM-Fc treatment group for single-cell sequencing. We compared the subsets of immune cells in the peripheral blood and lung tumors of the negative control group. We observed a significant expansion of T cells, B cells, and NK cells in the peripheral blood of mice receiving HVEM-Fc treatment, suggesting the mobilization of immune cells into the peripheral circulation [45](Fig. 6M). Furthermore, the proportions of naïve T cells, CD4^+^ T cells, and CD8^+^ T cells in the peripheral blood were significantly higher in the treatment group compared to the negative control group, indicating that local immune cells in the tumor microenvironment primarily derive from the peripheral blood (Fig. 6N-O).

### Spatial transcriptomic analysis

**Fig. 7:**
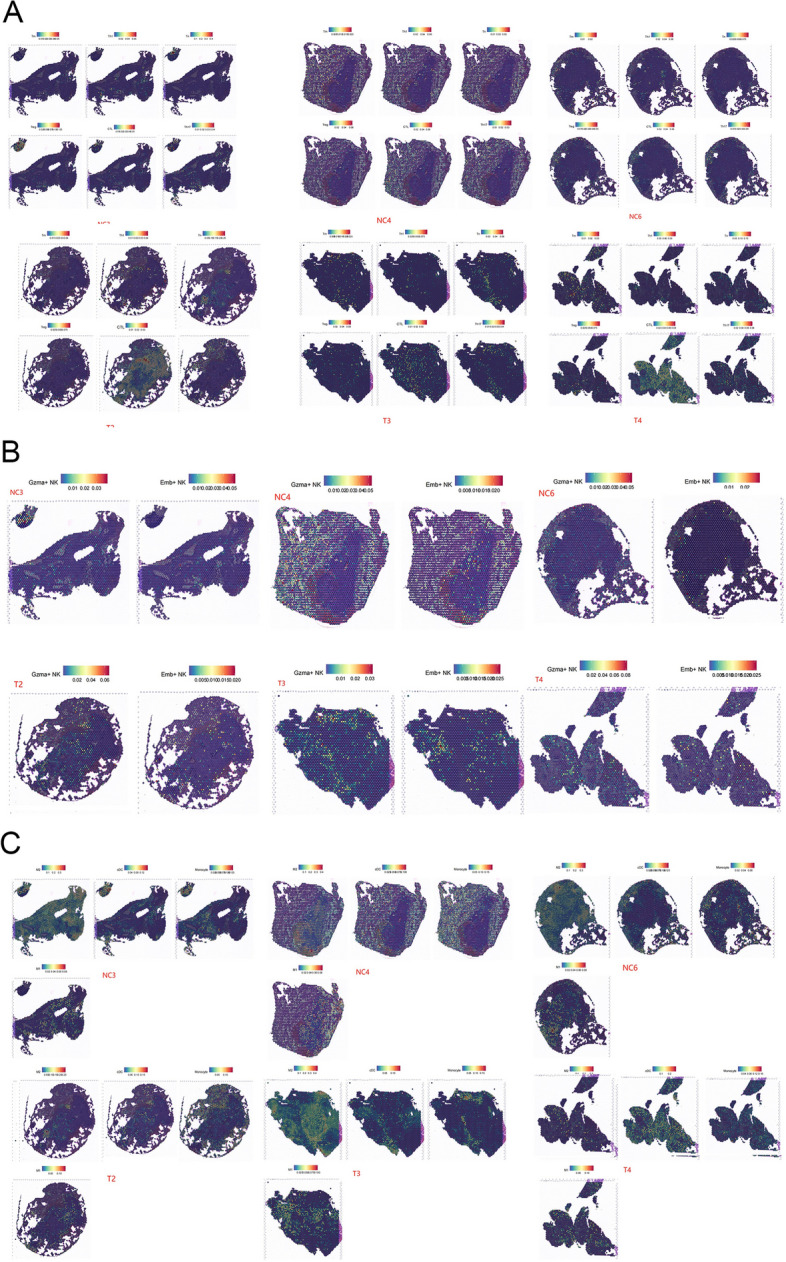
T cells, NK cells and myeloid cells in the tumor microenvironment. Notes:** A** The T cells in the tumor microenvironment; **B** The NK cells in the tumor microenvironment; **C** The myeloid cells in the tumor microenvironment

Spatial transcriptomic sequencing was employed to analyze lung tumors from the HVEM-Fc treatment group and the negative control group. Of the three lung tumors analyzed, two exhibited therapeutic responses (T2, T4), while the remaining tumor revealed no response (T3; FigureS7A-B). This variability in response could be attributed to the heterogeneity of tumor cells and their surrounding tumor microenvironment. Therefore, a deeper understanding is required to elucidate the underlying mechanisms driving this observed spatial structure.

Firstly, we investigated the correlations between T cells and tumor cells. Results demonstrated a significantly higher number of CTLs and naïve T cells surrounding tumor tissue compared to the NC group (Fig. 7A). Secondly, an increased abundance of Gzma-expressing and Eomes-expressing NK cells was observed within the vicinity of cancer cells (Fig. 7B). To investigate the underlying reasons for the non-responsiveness observed in T3, we considered the concept of an "immunity barrier" within NSCLC patients, as described by Lan et al. This barrier encompasses factors such as M2-like macrophages and CAFs, which can hinder T cell function and diminish the efficacy of immunotherapeutic interventions [46]. Consistently, Zhou et al. demonstrated that interactions between CAF cells and M2 macrophages in NSCLC patients confer increased resistance to immunotherapy [47]. Furthermore, a higher density of matrix CAFs is associated with "immunologically cold" tumors and predicts a poor prognosis in NSCLC patients [48]. Analysis of myeloid cells revealed a greater distribution of dendritic cells within the tumor nests (Fig. 7C). Moreover, utilizing SPP1 and RPLP1 as markers for M2 macrophages and CAFs, respectively, we confirmed the presence of an immune barrier in T3 tissues. Notably, this characteristic immune barrier phenotype was not observed in the other two lung cancer tissues examined (Fig. 7C, S8).

Collectively, the responsive mouse model exhibited a higher abundance of CTLs with a reduced presence of M2-like macrophages and CAFs, suggesting that the immunotherapeutic efficacy of HVEM-Fc is influenced by multiple factors, including spatial structure and immune cell infiltration.

## Discussion

Cancer immunotherapy, which assists immune cells, including T cells, NK cells, and macrophages, in identifying and eliminating malignant cells, has garnered significant recent attention. The tumor microenvironment constitutes a complex ecosystem that fosters tumor growth and metastasis, encompassing cancer cells and numerous immunosuppressive components [49]. Targeting the constituents involved within the tumor microenvironment has been proposed as a novel strategy to enhance anti-tumor responses within the realm of cancer immunology research. Immune checkpoint inhibitors have substantially extended the survival of NSCLC patients, ushering in a new era of immunotherapy. The identification of novel immune checkpoint proteins expressed on the membranes of T cells constitutes a research focal point. The inhibition of the PD1/PD-L1 axis amplifies the anti-tumor efficacy of T cells, thereby promoting anti-tumor immunity [50]. Immune checkpoint proteins can function through receptor-ligand interactions between T cells and cancer cells, consequently inhibiting or activating the anti-tumor function of T cells, and thus promoting or suppressing tumor growth and metastasis.

Although the HVEM-BTLA-CD160 or HVEM-LIGHT axis has been extensively studied for decades, limited research has focused on compounds, antibodies, or peptides targeting this axis for cancer immunotherapy [51]. While Olive et al. demonstrated that anti-HVEM antibodies can suppress tumor metastasis by activating T-cell mediated immunity in a mouse lung cancer model [13], HVEM presents a challenging target due to its context-dependent function as both a stimulatory and inhibitory molecule. This complexity arises from receptor-ligand interactions within the tumor microenvironment, including both intercellular and cis interactions [10]. Consequently, HVEM is considered a "molecular switch" capable of either activating or inhibiting T-cell functions. Monoclonal antibodies have emerged as a prominent therapeutic modality in cancer immunotherapy [52], albeit with limitations such as high production costs and complex manufacturing processes. Soluble immune checkpoint proteins have shown potential to enhance anti-tumor effects [53]. For instance, Jin et al. demonstrated that recombinant PD-1 protein can exert anti-tumor immunity, suggesting a novel therapeutic approach [14]. Soluble checkpoints can interact with the same ligands or receptors as their membrane-bound counterparts, thereby regulating T-cell cytokine secretion and cytotoxicity [54]. Given that HVEM has four known ligands, with HVEM-BTLA mediating immunosuppression and HVEM-LIGHT promoting immunostimulation [55], we hypothesized that recombinant HVEM protein could exert anti-tumor effects by preferentially interacting with BTLA and thus interfering with the HVEM-BTLA pathway. The synthesis of recombinant HVEM protein was entrusted to Novoprotein Company. Our findings revealed that low-dose HVEM-Fc protein effectively enhanced T-cell mediated anti-tumor function in both animal models and human NSCLC organoids. Interestingly, high-dose HVEM-Fc did not exhibit anti-tumor activity, suggesting that simultaneous engagement of both BTLA and LIGHT at high doses may diminish or even reverse the anti-tumor effect.

The soluble form of HVEM plays a crucial role in modulating tumor immunity and is associated with the responsiveness to PD-1 inhibitors, although the precise mechanisms remain elusive [56]. While some studies have reported elevated soluble HVEM levels in patients with liver, gastric, and nasopharyngeal cancers, others have observed decreased levels in patients with early-stage breast and nasopharyngeal cancer [57–61]. Furthermore, several studies have found a correlation between increasing soluble HVEM levels and advanced stages of NSCLC [62]. The functional effects of soluble HVEM are currently conflicting and inconsistent. For instance, our previous findings demonstrated the anti-tumor activity of HVEM-Fc, potentially due to discrepancies between cellular and in vivo studies versus analyses of soluble HVEM levels in patient sera. Moreover, as a member of the TNF superfamily, HVEM can regulate the production of multifunctional pro-inflammatory cytokines. Our experiments suggest that HVEM may function similarly to TNF-α, which has been reported to suppress tumors in early stages but promote metastasis in advanced stages [63, 64]. Taken together, these findings suggest that HVEM may represent a novel therapeutic target for lung cancer.

In our study, the tumoricidal activity of HVEM-Fc was significantly superior to that of the control group and the PD-1 inhibitor group. Furthermore, the combination of PD-1 inhibitor and HVEM-Fc demonstrated enhanced efficacy in the LLC-induced mouse lung model and the GEMM model of lung cancer, eradicating the majority of lung tumors. This combination therapy induced long-term survival and resulted in the generation of immune memory, enabling the effective rejection of subsequent challenges with LLC lung cells.

Through immune cell depletion experiments, we determined that the anti-tumor effects of HVEM-Fc primarily require CD4^+^ and CD8^+^ T cells. Integrated transcriptomic analysis, serum cytokine profiling, and lung tumor analysis revealed significant enrichment of immune activation and immune cell-mediated cytotoxicity pathways, indicating that HVEM-Fc can activate anti-tumor immunity involving macrophages, natural killer cells, and CD8^+^ T cells. Notably, Emms et al. reported that breast cancer patients with enriched cell proliferation pathways exhibited sensitivity to immunotherapy and favorable prognoses, aligning with our observation that HVEM-Fc-treated mice responded well to PD-1 inhibitor therapy [65]. We further validated the anti-tumor effects of HVEM-Fc in human lung cancer cell line-derived organoids. These experiments demonstrated that HVEM-Fc had no effect on NSCLC cells in the absence of T cells, confirming its reliance on the immune system for anti-tumor activity. Interestingly, 0.8 μg/mL and 0.08 μg/mL HVEM-Fc exhibited minimal anti-tumor effects, while 8 μg/mL demonstrated robust anti-tumor capacity through T-cell activation compared to the control group. This observation may be attributed to the single-dose administration of HVEM-Fc in NSCLC organoids and T-cell co-culture models. However, multiple dosing regimens have been shown to effectively inhibit tumor growth and metastasis in mouse models. Collectively, these findings demonstrate that HVEM-Fc promotes the formation of a pro-inflammatory immune microenvironment, ultimately leading to tumor growth inhibition.

To account for the heterogeneity of the tumor microenvironment, we employed single-cell RNA sequencing and spatial transcriptomics to investigate the complex cellular ecosystems within the treatment and control groups [66, 67]. This approach enabled us to profile approximately 35,000 cells across both groups. Our analysis revealed a significant interaction between CTLs, NK cells, and tumor cells in the treatment group, suggesting these cell populations may be key targets. Additionally, we observed an increase in antigen-presenting CAFs following treatment. These CAFs can present MHC-peptide complexes, thereby stimulating the activation of CD4^+^ and CD8^+^ T cells in the early stages of the immune response [68]. Collectively, these findings demonstrate that HVEM-Fc treatment promotes the formation of an activated immune microenvironment.

Next, we observed that a subset of mice exhibited unresponsiveness to HVEM-Fc treatment. To investigate the underlying mechanisms, we employed spatial transcriptomics profiling on fresh lung cancer samples from control mice, treatment-responsive mice, and treatment-unresponsive mice. Compared to the control group, the treatment group demonstrated a higher density of NK cells, CTLs, and DCs infiltrating the tumor core and invasive margins, indicating the induction of a "hot" immune microenvironment. However, when comparing treatment-responsive and -unresponsive mice, we observed a higher abundance of M2-like TAMs and CAFs surrounding T cells in the unresponsive group, suggesting the formation of an immunosuppressive microenvironment that may inhibit anti-tumor responses. In the future, combining HVEM-Fc treatment with therapies selectively targeting M2-like TAMs or CAF cells may represent a promising therapeutic strategy.

Several limitations of this study should be acknowledged. Firstly, HVEM is a costimulatory molecule whose receptor-ligand interactions are influenced by factors such as antigen presentation through major histocompatibility complex, cytokines, and metabolites that fuel T cell metabolism [69]. Therefore, a more comprehensive understanding of its mechanisms in activating the immune microenvironment, its role in mediating immunological surveillance and memory responses upon tumor rechallenge, and its potential direct or indirect effects on T-cell subgroups requires further investigation. Secondly, the limited number of patient-derived organoids reduced the statistical power of our study. Thirdly, a larger patient cohort would be necessary to further validate these findings. Finally, the detailed reaction between HVEM-Fc and BTLA in immune cells should be further validated.

Overall, our experiments demonstrate that HVEM-Fc can activate the immune system against lung cancer in vivo and in organoid models. Through multi-omics data analysis, we revealed that HVEM-Fc reinvigorates T cells, enhances immune memory, and promotes the eradication of Tregs. Furthermore, the combination of PD-1 inhibitor and HVEM-Fc exhibited a synergistic anti-tumor effect, suggesting a promising therapeutic strategy for NSCLC patients that warrants further investigation.

## Supplementary Information

Supplementary file 1. Flow cytometry gating strategies for the identification of immune cell subsets.

Supplementary file 2. The alterations in the expression of multiple immune-related cytokines.

Supplementary file 3. The gene expression changes in the negative control (NC) group and treatment group.

Supplementary file 4. The alterations in the expression of multiple immune-related cytokines.

Supplementary file 5. Flow cytometry gating strategies for the identification of immune cell subsets.

Supplementary file 6. Table S1 Baseline characteristics of the two patients.

Supplementary file 7. Table S2 Proportion of CD4^+^ and CD8^+^ T Cells derived from PBMCs.

## Data Availability

No datasets were generated or analysed during the current study.

## Abbreviations

IHC: Immunohistochemical staining
TAM: Tumor-associated macrophage
PDOs: Patient-derived organoids
