# Supplementary material for: A novel HVEM-Fc recombinant protein for lung cancer immunotherapy
Source: J Exp Clin Cancer Res. 2025 Feb 20;44:62. doi: 10.1186/s13046-025-03324-8 (PMC11841141; doi:10.1186/s13046-025-03324-8)
Supplement: Supplementary file 1 — Flow cytometry gating strategies for the identification of immune cell subsets. [file 13046_2025_3324_MOESM1_ESM.zip › Supplement File1.pdf]

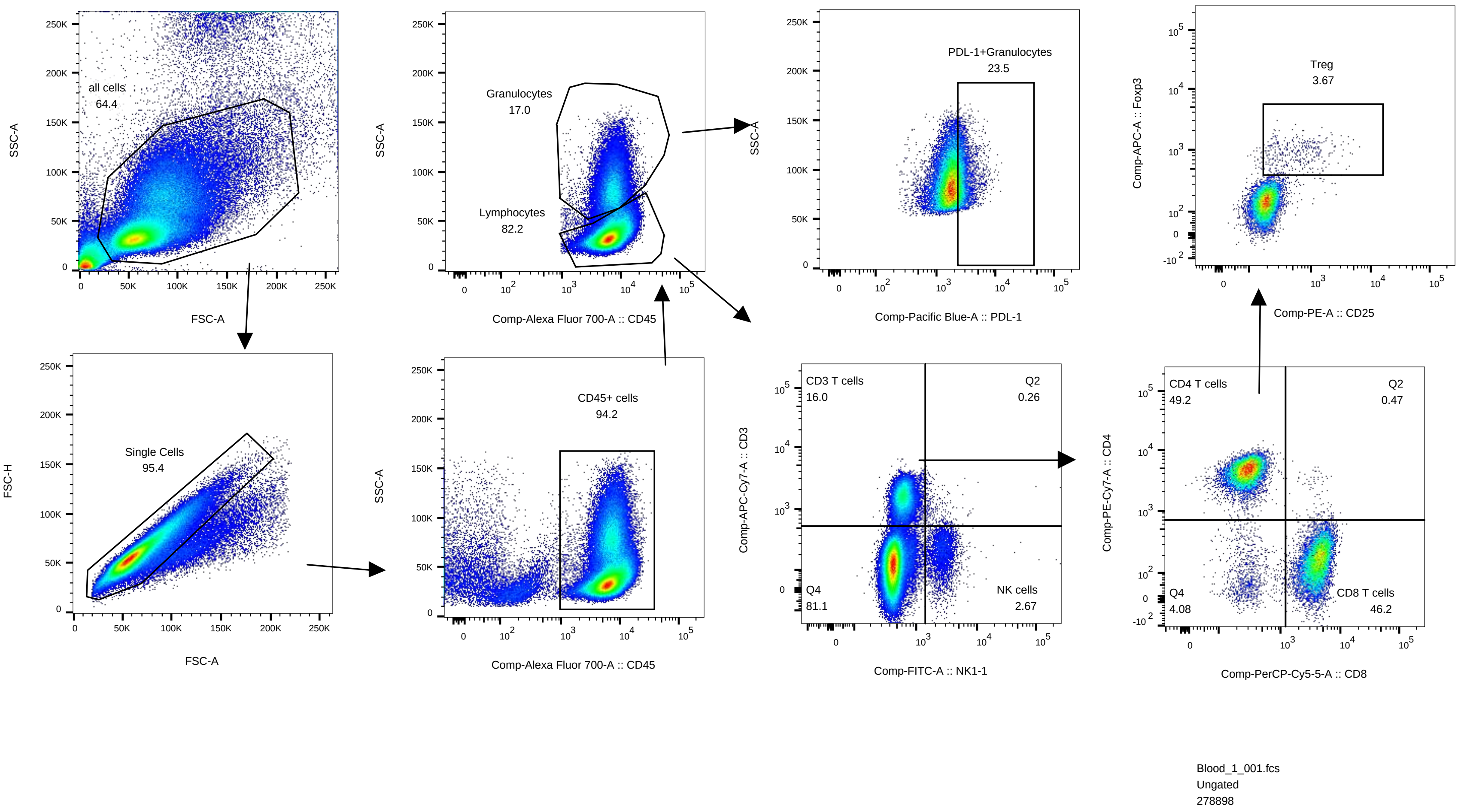

all cells
64.4
SSC-A
FSC-A
Single Cells
95.4
FSC-H
FSC-A
Granulocytes
17.0
Lymphocytes
82.2
SSC-A
Comp-Alexa Fluor 700-A :: CD45
CD45+ cells
94.2
SSC-A
Comp-Alexa Fluor 700-A :: CD45
PDL-1+Granulocytes
23.5
SSC-A
Comp-Pacific Blue-A :: PDL-1
CD3 T cells
16.0
Q2
0.26
Q4
81.1
NK cells
2.67
Comp-APC-Cy7-A :: CD3
Comp-FITC-A :: NK1-1
Treg
3.67
Comp-APC-A :: Foxp3
Comp-PE-A :: CD25
CD4 T cells
49.2
Q2
0.47
Q4
4.08
CD8 T cells
46.2
Comp-PE-Cy7-A :: CD4
Comp-PerCP-Cy5-5-A :: CD8
Blood_1_001.fcs
Ungated
278898

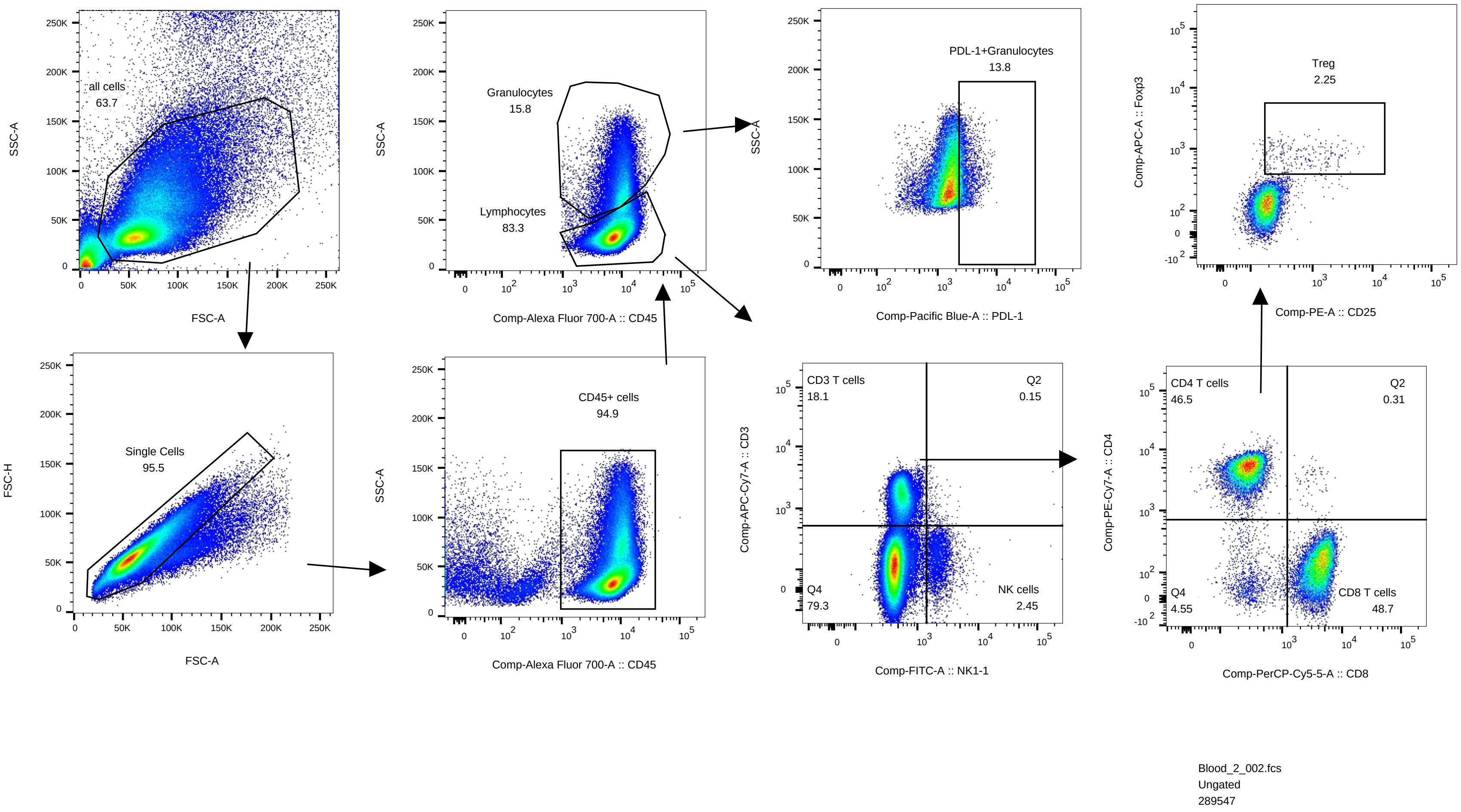
all cells
63.7
SSC-A
FSC-A
Single Cells
95.5
FSC-H
FSC-A
Granulocytes
15.8
Lymphocytes
83.3
SSC-A
Comp-Alexa Fluor 700-A :: CD45
CD45+ cells
94.9
SSC-A
Comp-Alexa Fluor 700-A :: CD45
PDL-1+Granulocytes
13.8
SSC-A
Comp-Pacific Blue-A :: PDL-1
CD3 T cells
18.1
Q2
0.15
Q4
79.3
NK cells
2.45
Comp-APC-Cy7-A :: CD3
Comp-FITC-A :: NK1-1
Treg
2.25
Comp-APC-A :: Foxp3
Comp-PE-A :: CD25
CD4 T cells
46.5
Q2
0.31
Q4
4.55
CD8 T cells
48.7
Comp-PE-Cy7-A :: CD4
Comp-PerCP-Cy5-5-A :: CD8
Blood_2_002.fcs
Ungated
289547

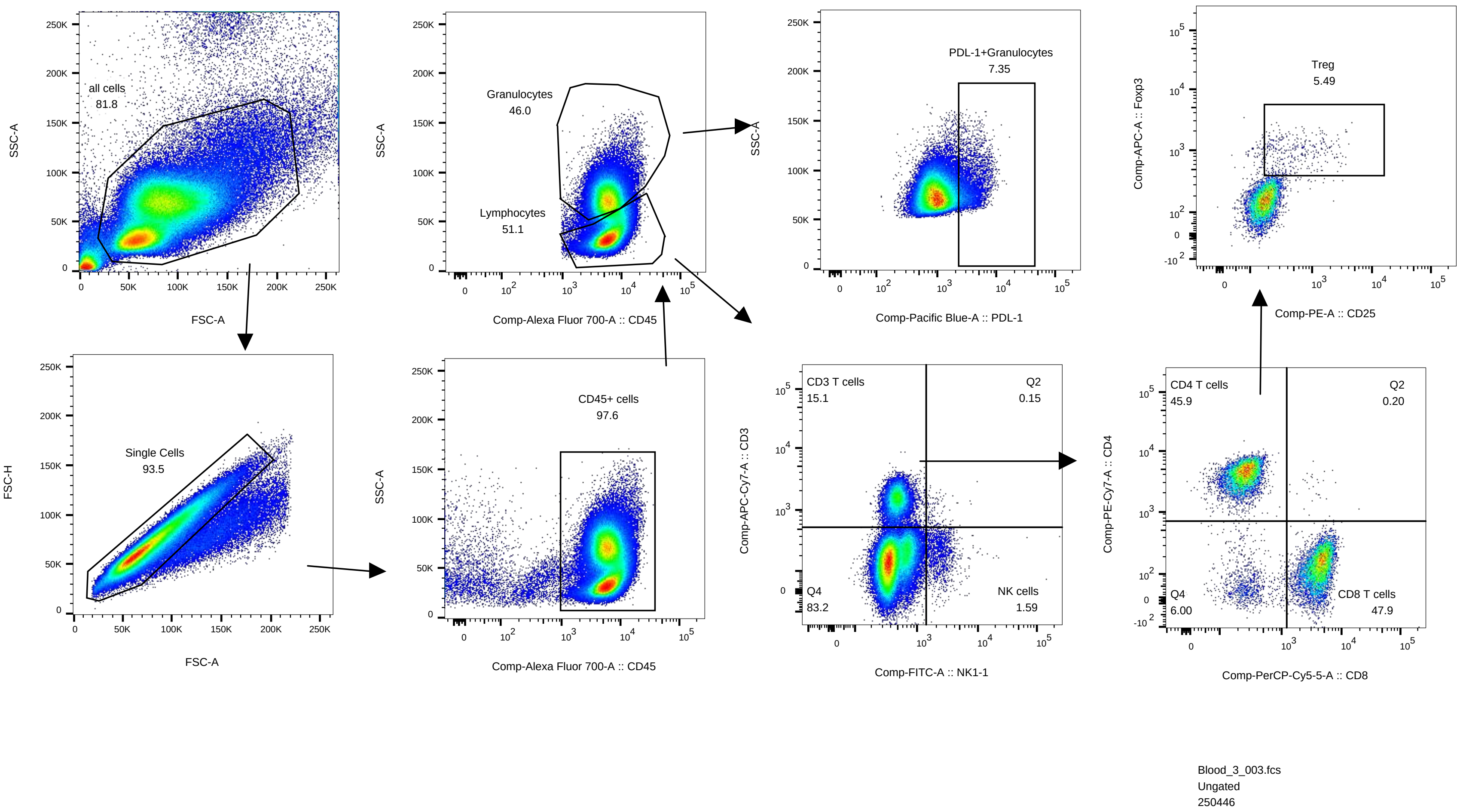

all cells
81.8
SSC-A
FSC-A
Single Cells
93.5
FSC-H
FSC-A
Granulocytes
46.0
Lymphocytes
51.1
SSC-A
Comp-Alexa Fluor 700-A :: CD45
CD45+ cells
97.6
SSC-A
Comp-Alexa Fluor 700-A :: CD45
PDL-1+Granulocytes
7.35
SSC-A
Comp-Pacific Blue-A :: PDL-1
CD3 T cells
15.1
Q2
0.15
Q4
83.2
NK cells
1.59
Comp-APC-Cy7-A :: CD3
Comp-FITC-A :: NK1-1
Treg
5.49
Comp-APC-A :: Foxp3
Comp-PE-A :: CD25
CD4 T cells
45.9
Q2
0.20
Q4
6.00
CD8 T cells
47.9
Comp-PE-Cy7-A :: CD4
Comp-PerCP-Cy5-5-A :: CD8
Blood_3_003.fcs
Ungated
250446

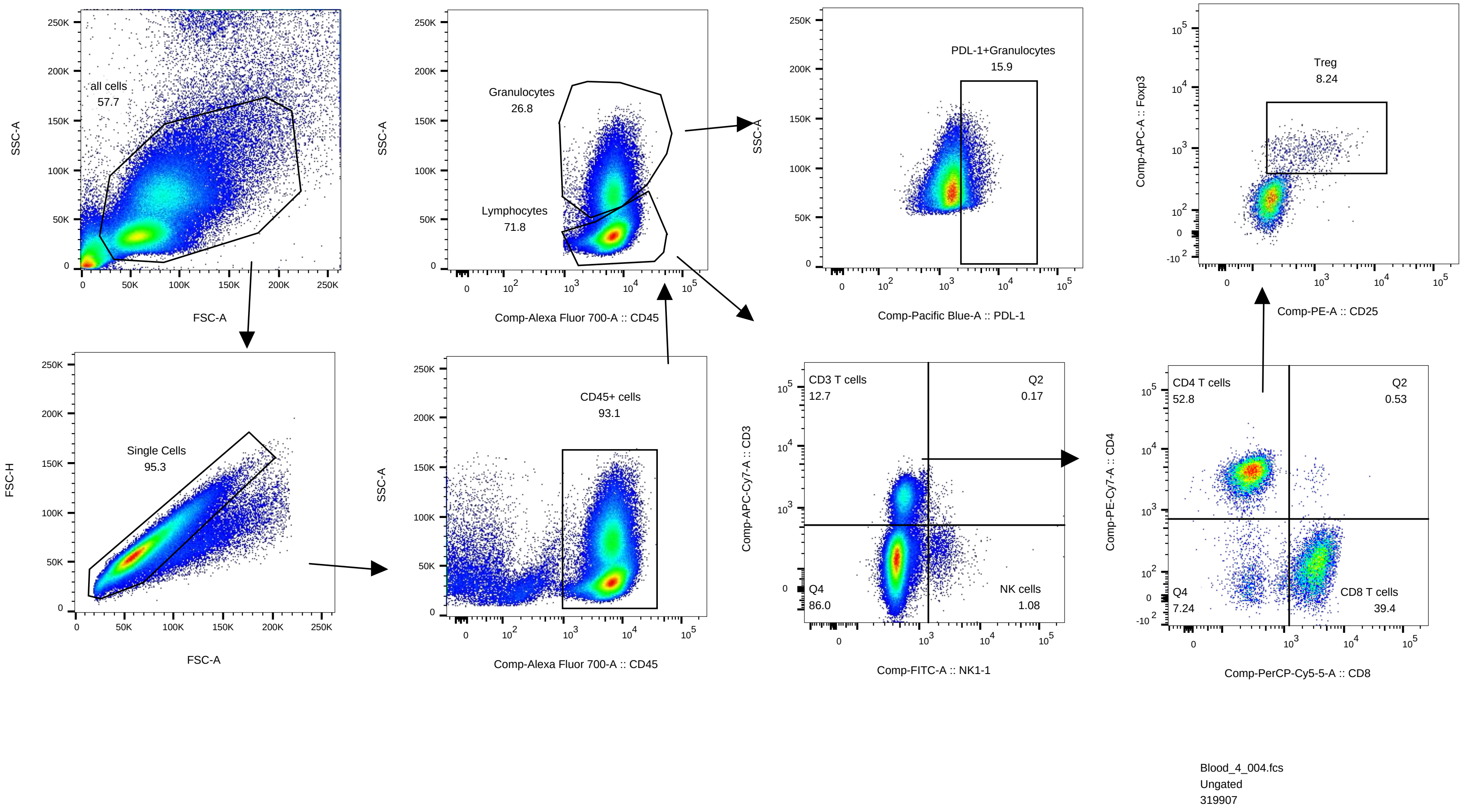

all cells
57.7
SSC-A
FSC-A
Single Cells
95.3
FSC-H
FSC-A
Granulocytes
26.8
Lymphocytes
71.8
SSC-A
Comp-Alexa Fluor 700-A :: CD45
CD45+ cells
93.1
SSC-A
Comp-Alexa Fluor 700-A :: CD45
PDL-1+Granulocytes
15.9
SSC-A
Comp-Pacific Blue-A :: PDL-1
CD3 T cells
12.7
Q2
0.17
Q4
86.0
NK cells
1.08
Comp-APC-Cy7-A :: CD3
Comp-FITC-A :: NK1-1
Treg
8.24
Comp-APC-A :: Foxp3
Comp-PE-A :: CD25
CD4 T cells
52.8
Q2
0.53
Q4
7.24
CD8 T cells
39.4
Comp-PE-Cy7-A :: CD4
Comp-PerCP-Cy5-5-A :: CD8
Blood_4_004.fcs
Ungated
319907

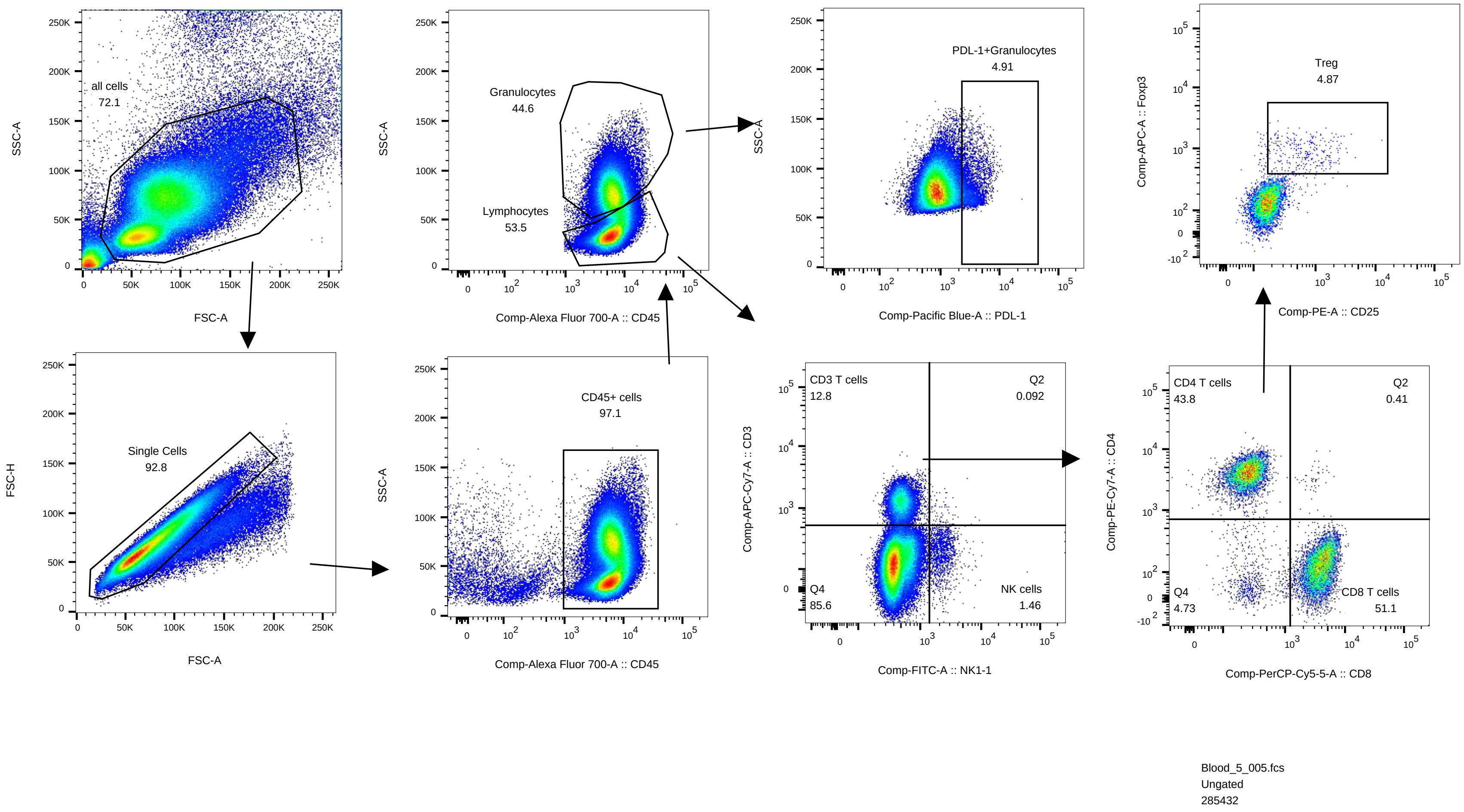
all cells
72.1
SSC-A
FSC-A
Single Cells
92.8
FSC-H
FSC-A
Granulocytes
44.6
Lymphocytes
53.5
SSC-A
Comp-Alexa Fluor 700-A :: CD45
CD45+ cells
97.1
SSC-A
Comp-Alexa Fluor 700-A :: CD45
PDL-1+Granulocytes
4.91
SSC-A
Comp-Pacific Blue-A :: PDL-1
CD3 T cells
12.8
Q2
0.092
Q4
85.6
NK cells
1.46
Comp-APC-Cy7-A :: CD3
Comp-FITC-A :: NK1-1
Treg
4.87
Comp-APC-A :: Foxp3
Comp-PE-A :: CD25
CD4 T cells
43.8
Q2
0.41
Q4
4.73
CD8 T cells
51.1
Comp-PE-Cy7-A :: CD4
Comp-PerCP-Cy5-5-A :: CD8
Blood_5_005.fcs
Ungated
285432

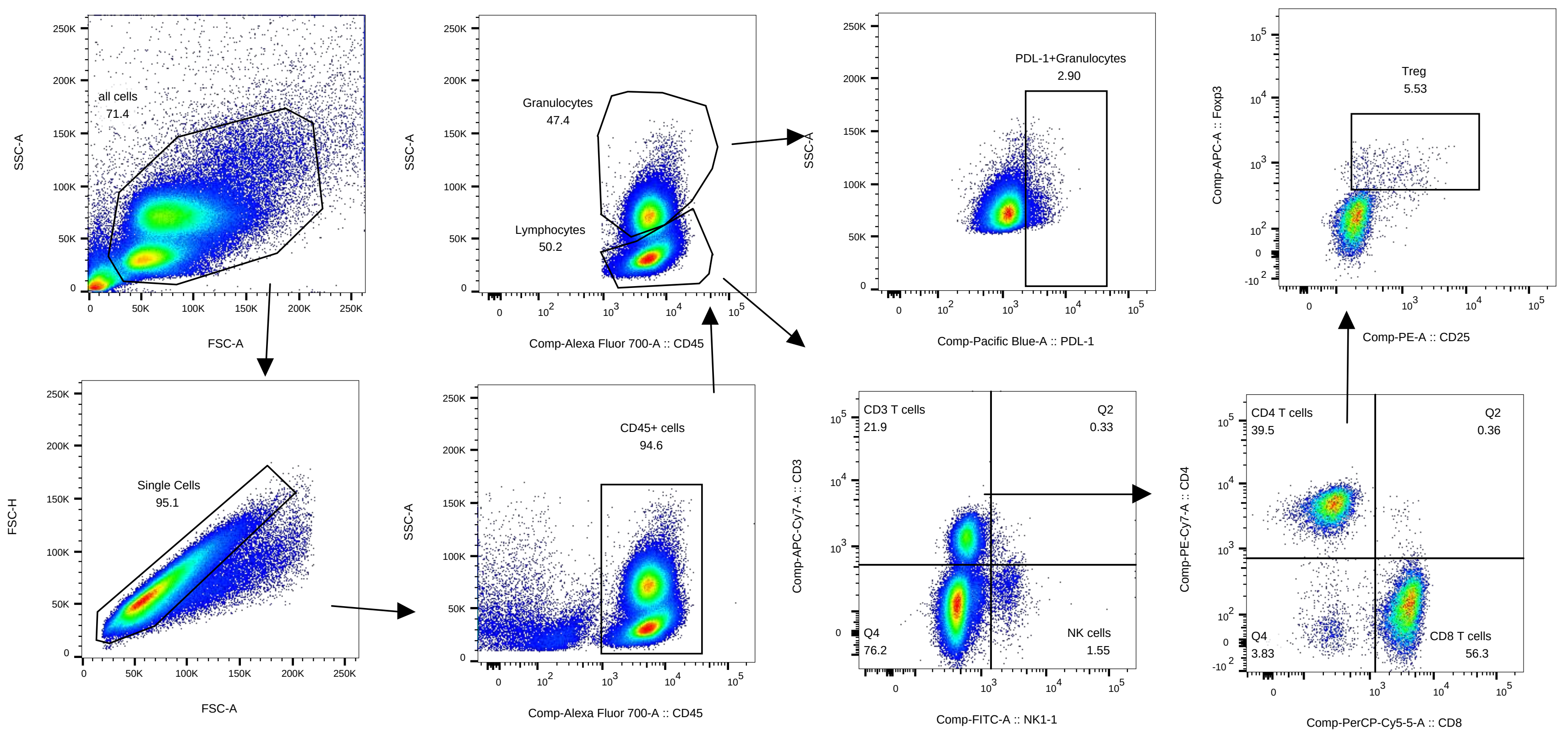

Blood_6_006.fcs
Ungated
283153

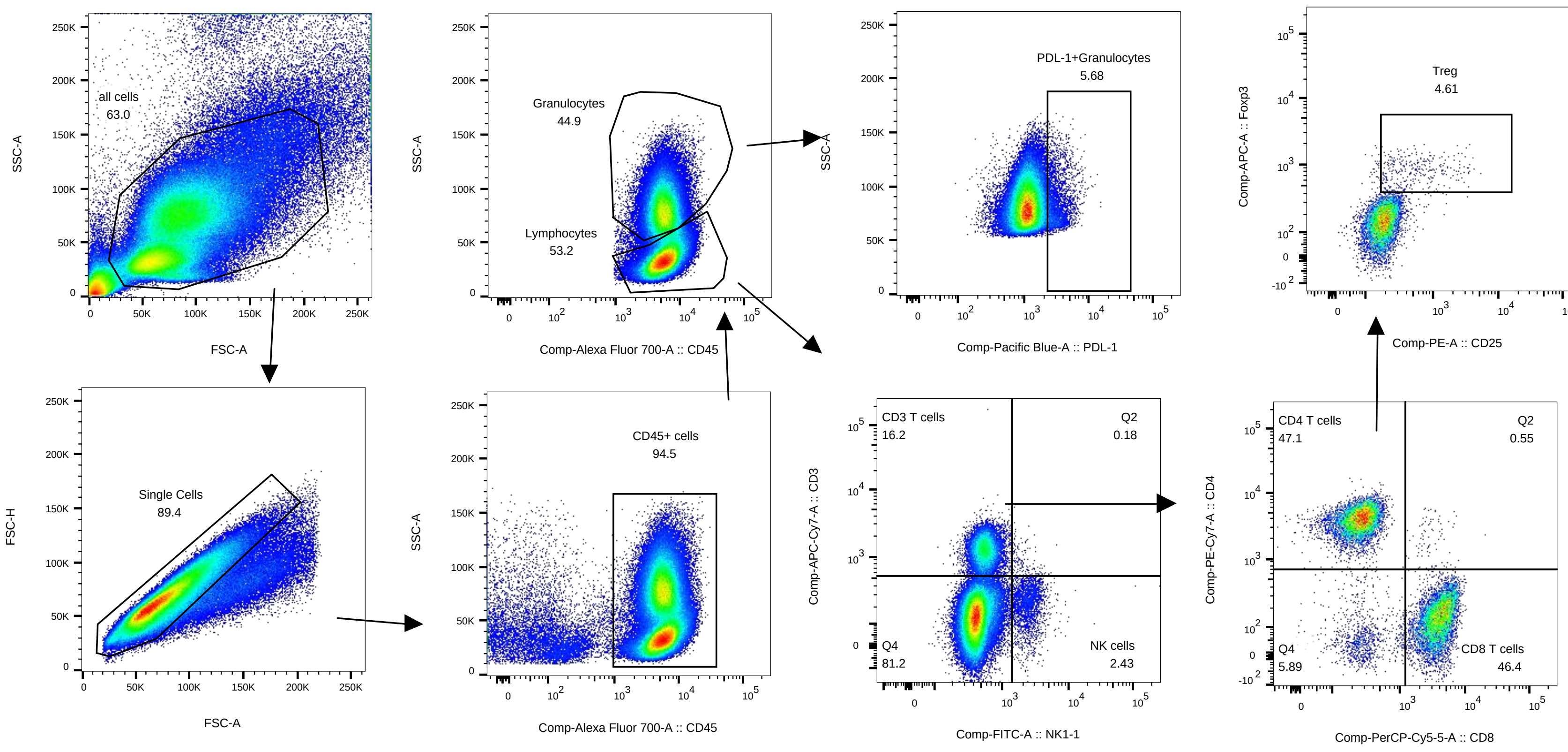

Blood_7_007.fcs
Ungated
331016

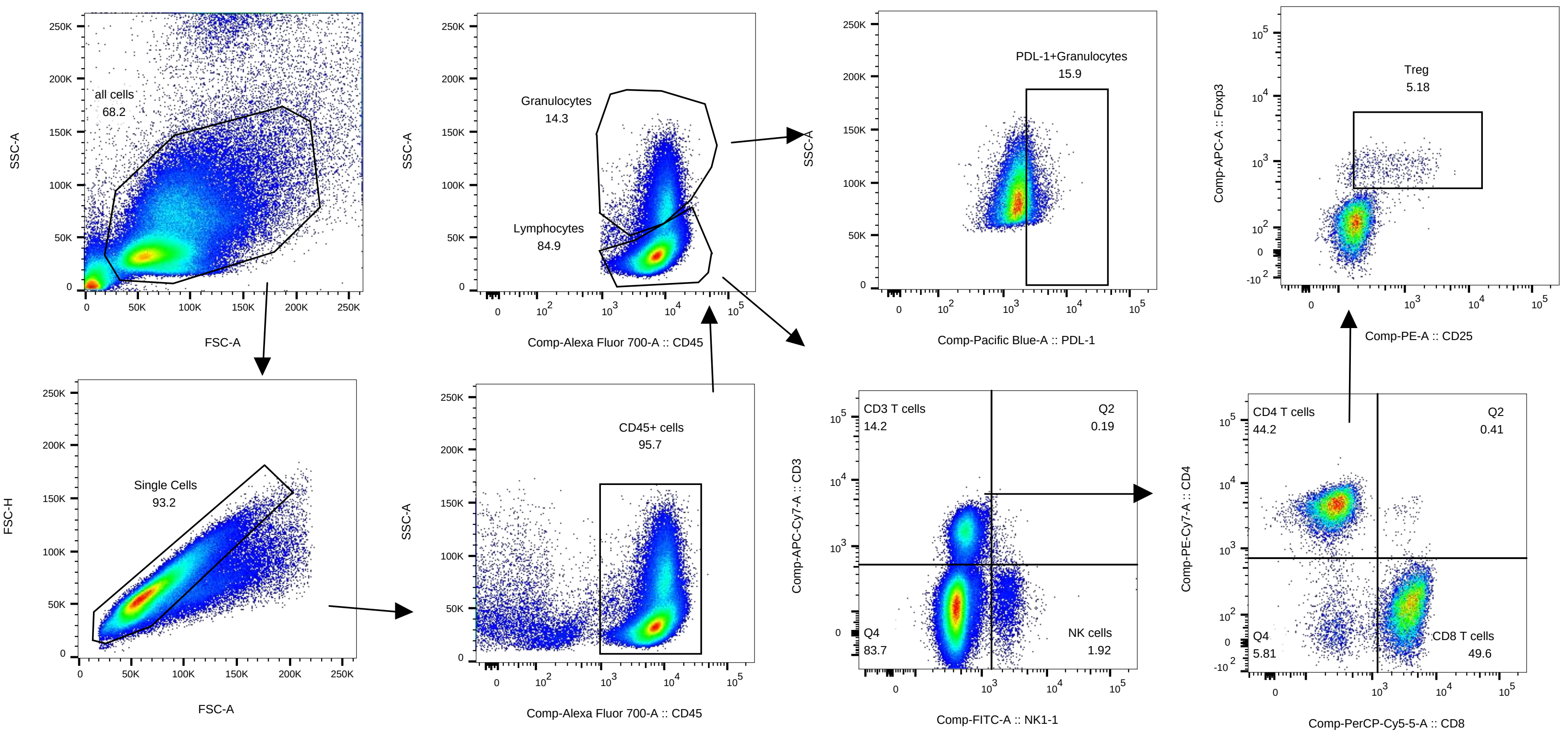

Blood_8_008.fcs
Ungated
295969

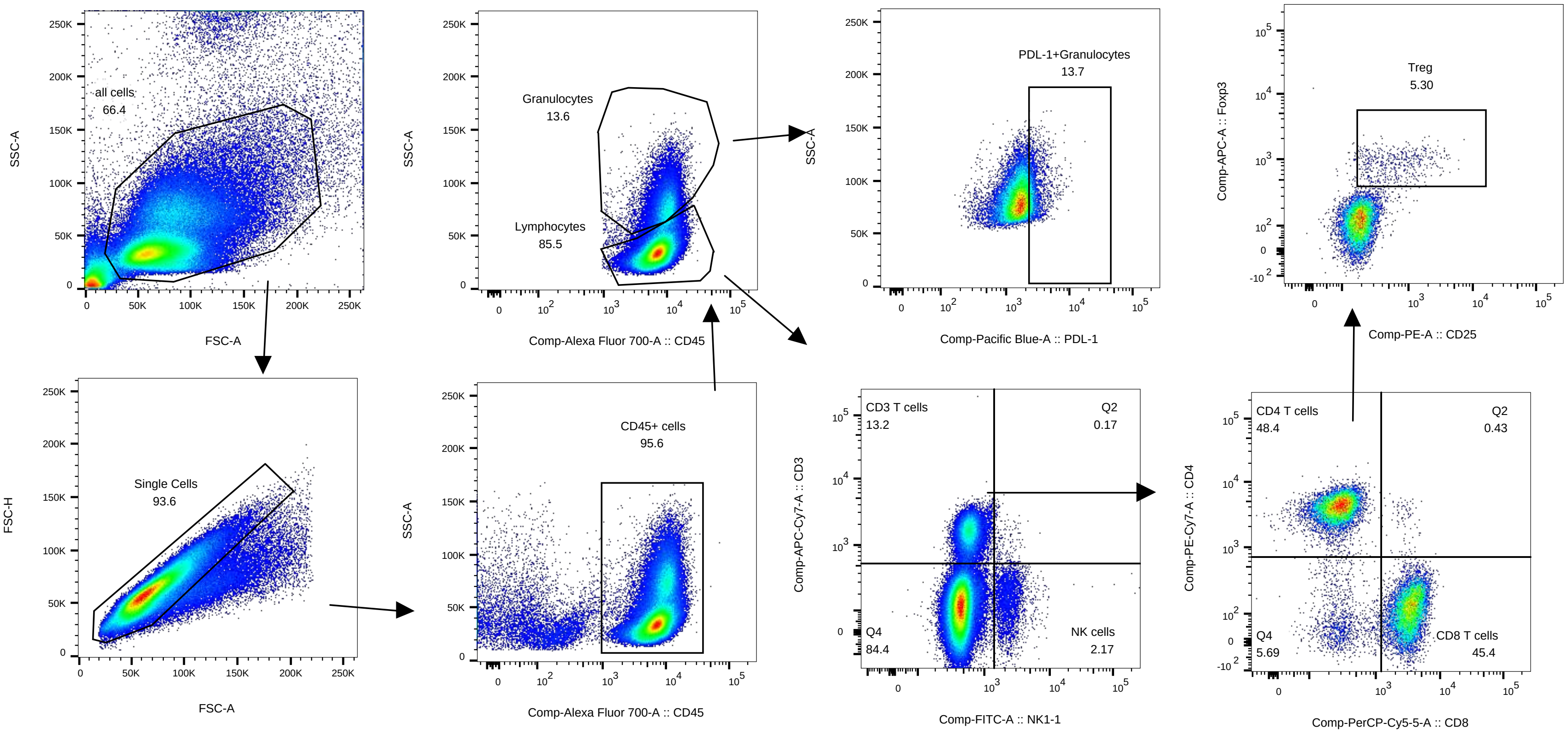

Blood_9_009.fcs
Ungated
303116

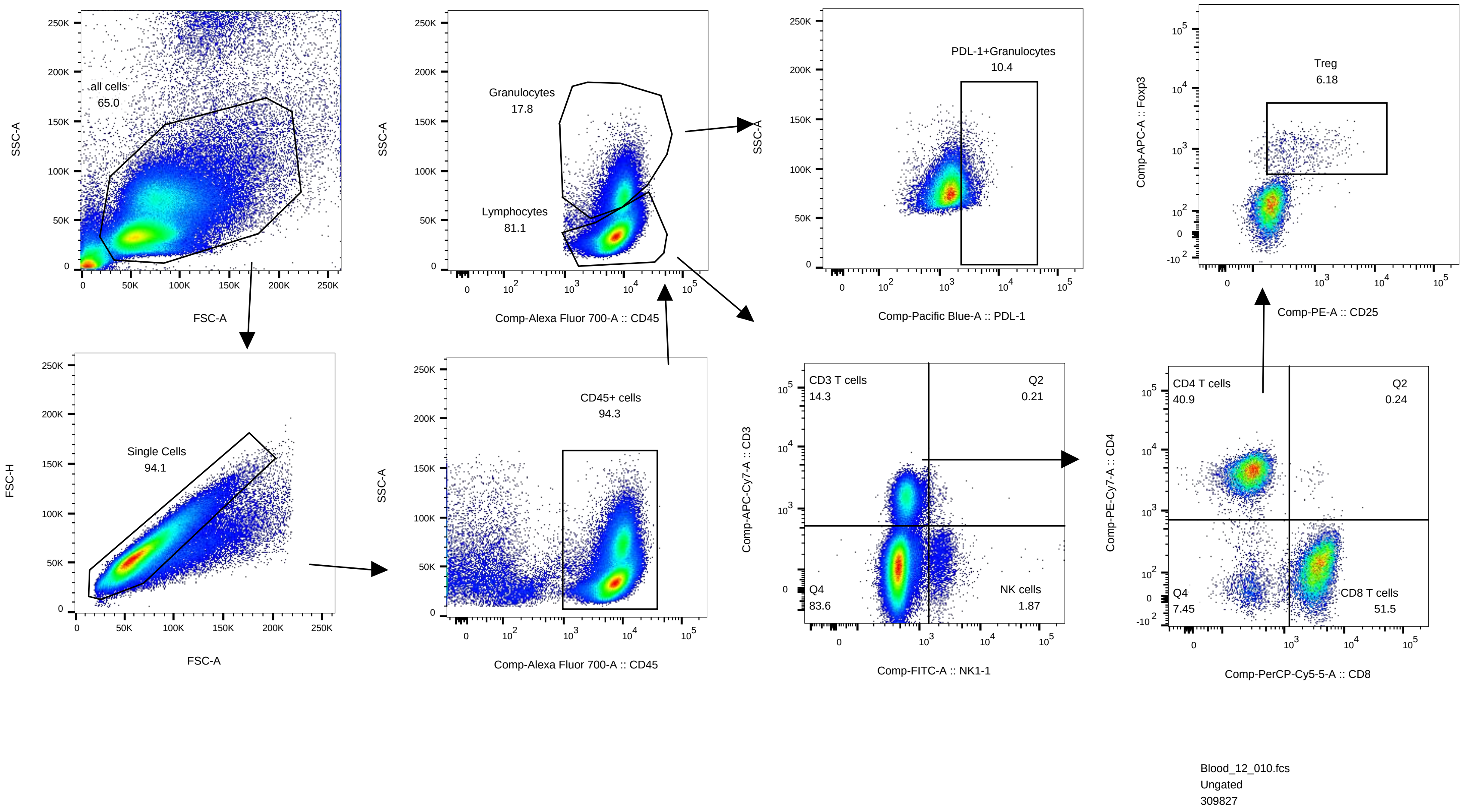
all cells
65.0
SSC-A
FSC-A
Single Cells
94.1
FSC-H
Granulocytes
17.8
Lymphocytes
81.1
Comp-Alexa Fluor 700-A :: CD45
CD45+ cells
94.3
PDL-1+Granulocytes
10.4
Comp-Pacific Blue-A :: PDL-1
CD3 T cells
14.3
Q2
0.21
Q4
83.6
NK cells
1.87
Comp-APC-Cy7-A :: CD3
Comp-FITC-A :: NK1-1
Treg
6.18
Comp-APC-A :: Foxp3
Comp-PE-A :: CD25
CD4 T cells
40.9
Q2
0.24
Q4
7.45
CD8 T cells
51.5
Comp-PE-Cy7-A :: CD4
Comp-PerCP-Cy5-5-A :: CD8
Blood_12_010.fcs
Ungated
309827

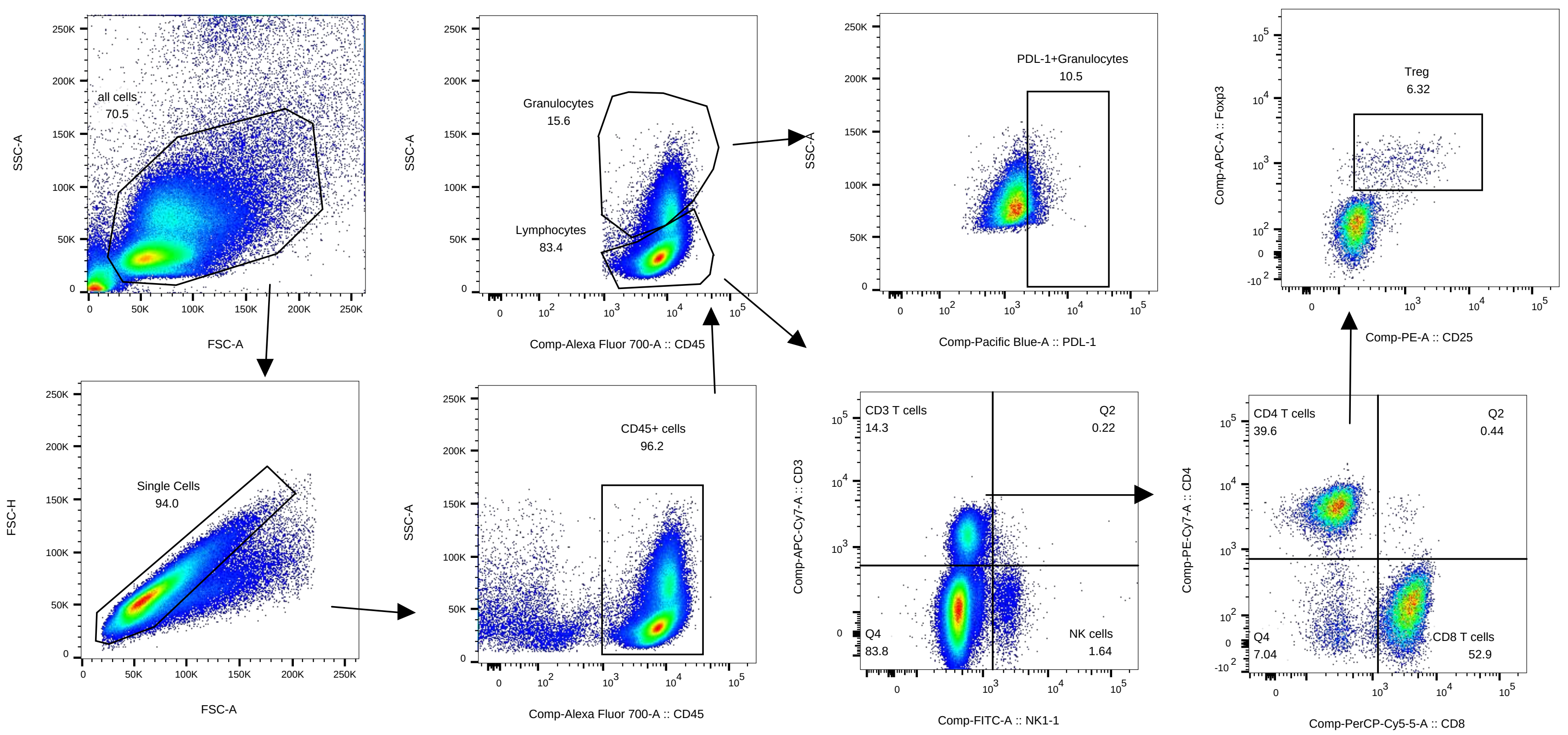

Blood_13_011.fcs
Ungated
285826

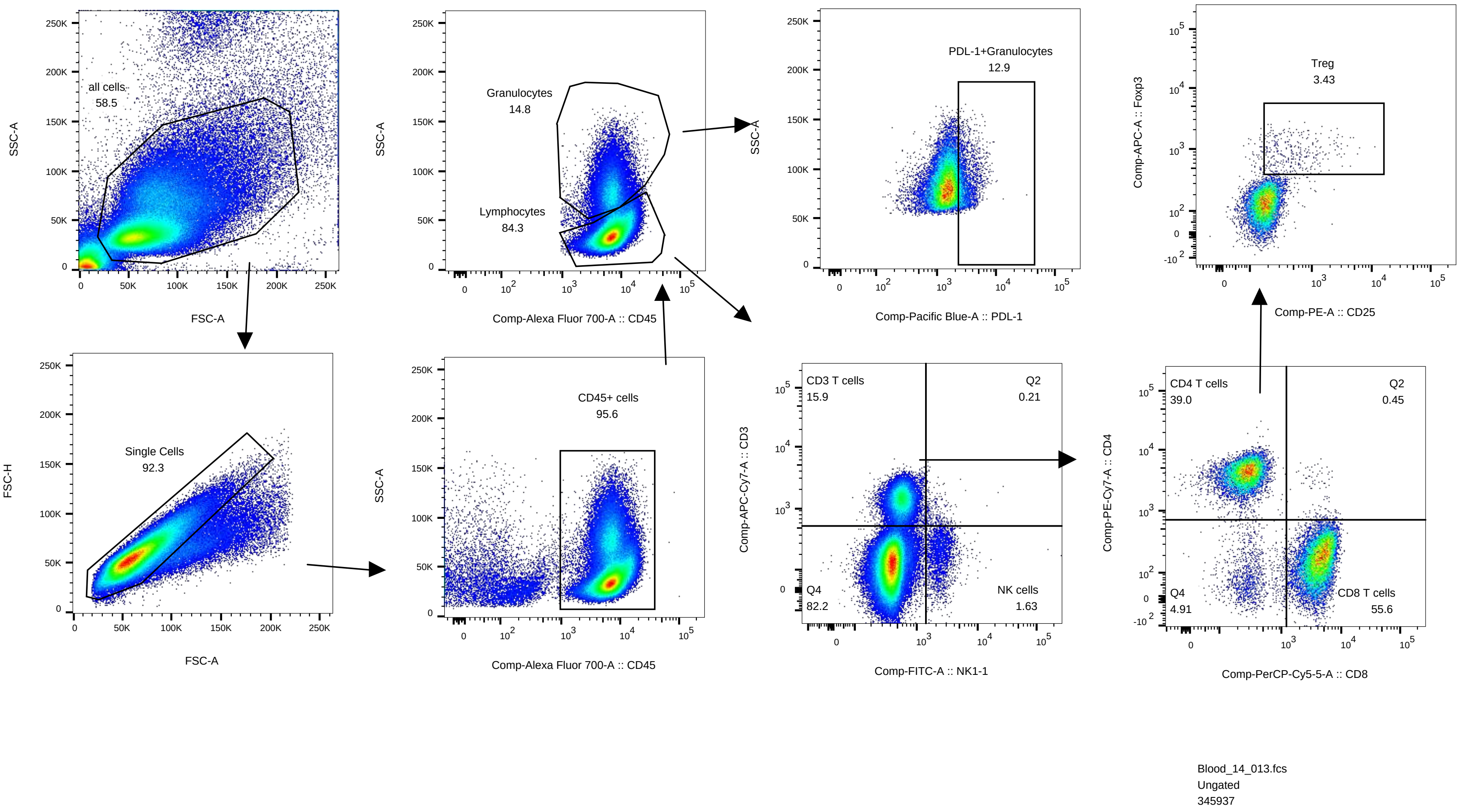
all cells
58.5
SSC-A
FSC-A
Single Cells
92.3
FSC-H
FSC-A
Granulocytes
14.8
Lymphocytes
84.3
SSC-A
Comp-Alexa Fluor 700-A :: CD45
CD45+ cells
95.6
SSC-A
Comp-Alexa Fluor 700-A :: CD45
PDL-1+Granulocytes
12.9
SSC-A
Comp-Pacific Blue-A :: PDL-1
CD3 T cells
15.9
Q2
0.21
Q4
82.2
NK cells
1.63
Comp-APC-Cy7-A :: CD3
Comp-FITC-A :: NK1-1
Treg
3.43
Comp-APC-A :: Foxp3
Comp-PE-A :: CD25
CD4 T cells
39.0
Q2
0.45
Q4
4.91
CD8 T cells
55.6
Comp-PE-Cy7-A :: CD4
Comp-PerCP-Cy5-5-A :: CD8
Blood_14_013.fcs
Ungated
345937

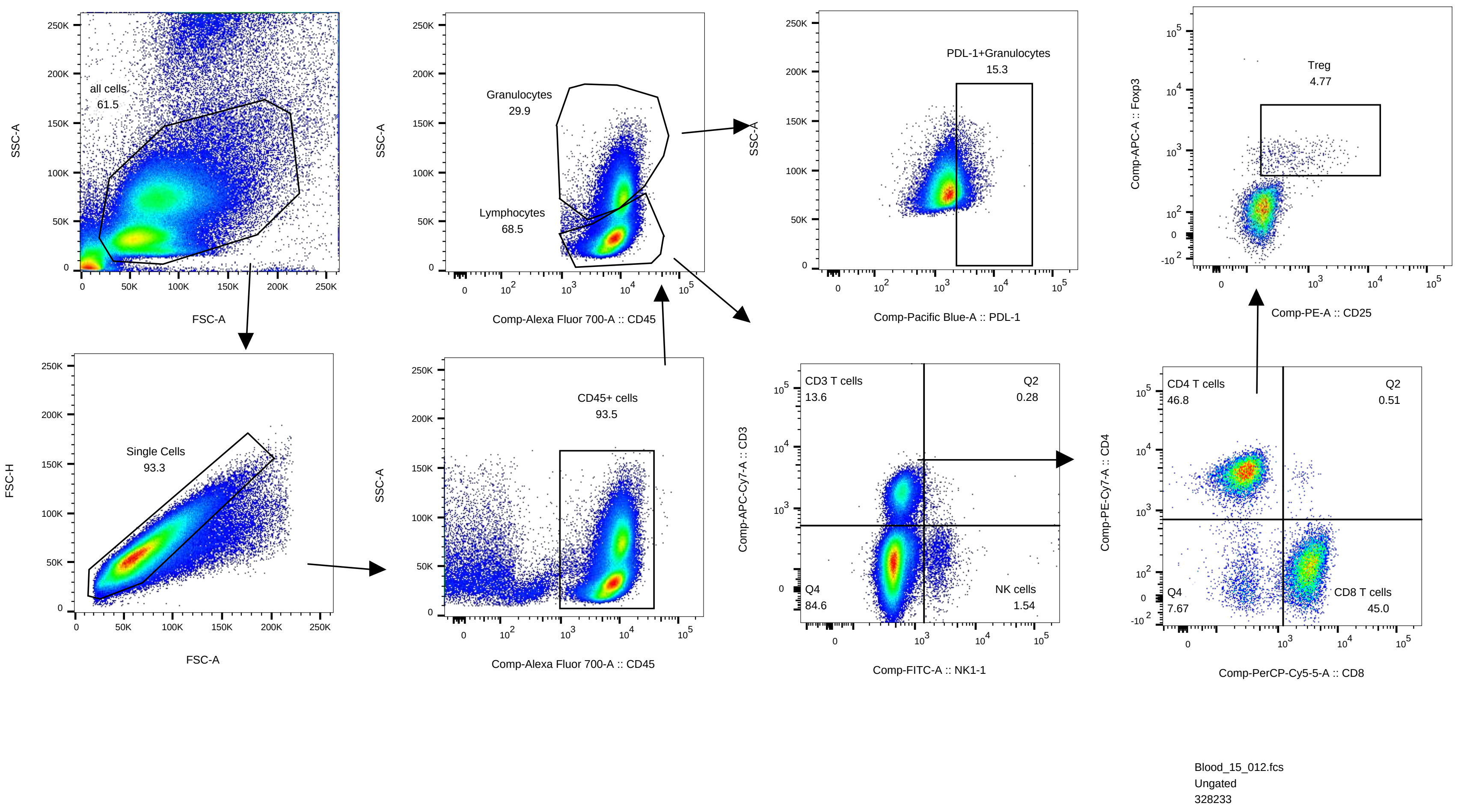
all cells
61.5
SSC-A
FSC-A
Granulocytes
29.9
Lymphocytes
68.5
Comp-Alexa Fluor 700-A :: CD45
PDL-1+Granulocytes
15.3
Comp-Pacific Blue-A :: PDL-1
Treg
4.77
Comp-APC-A :: Foxp3
Comp-PE-A :: CD25
Single Cells
93.3
FSC-H
CD45+ cells
93.5
CD3 T cells
13.6
Q2
0.28
Q4
84.6
NK cells
1.54
Comp-APC-Cy7-A :: CD3
Comp-FITC-A :: NK1-1
CD4 T cells
46.8
Q2
0.51
Q4
7.67
CD8 T cells
45.0
Comp-PE-Cy7-A :: CD4
Comp-PerCP-Cy5-5-A :: CD8
Blood_15_012.fcs
Ungated
328233

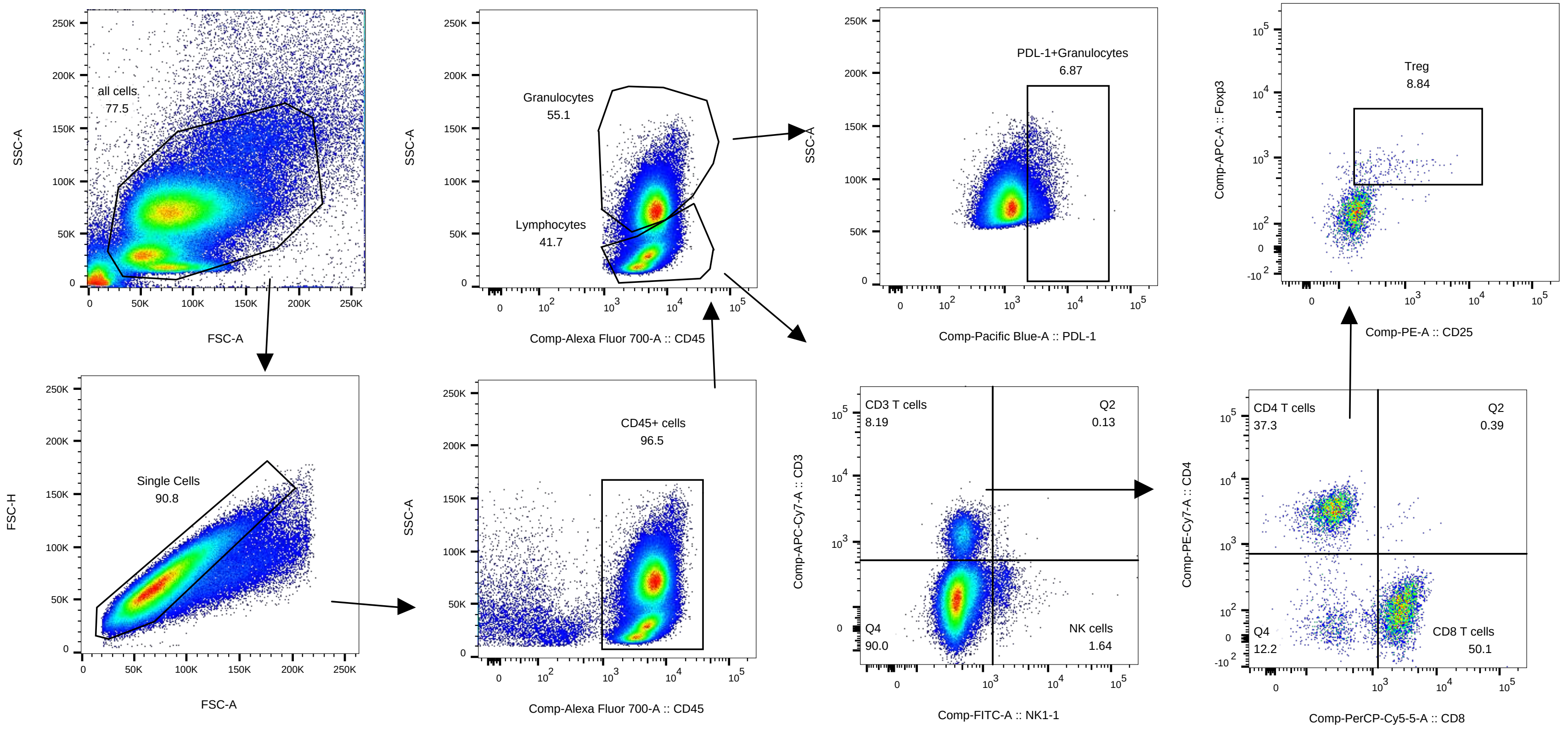

Blood_16_014.fcs
Ungated
265579

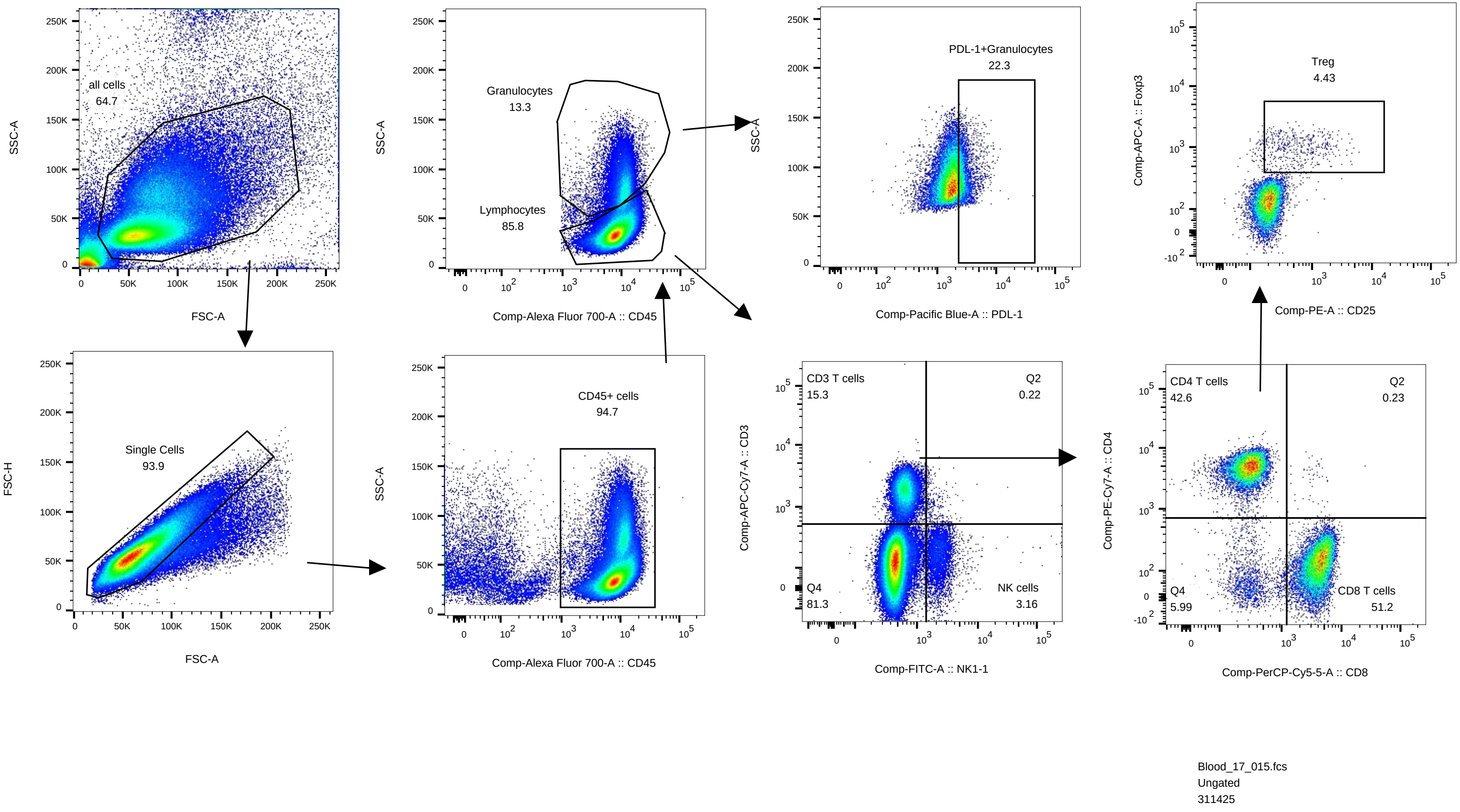
all cells
64.7
SSC-A
FSC-A
Single Cells
93.9
FSC-H
Granulocytes
13.3
Lymphocytes
85.8
Comp-Alexa Fluor 700-A :: CD45
CD45+ cells
94.7
PDL-1+Granulocytes
22.3
Comp-Pacific Blue-A :: PDL-1
CD3 T cells
15.3
Q2
0.22
Q4
81.3
NK cells
3.16
Comp-APC-Cy7-A :: CD3
Comp-FITC-A :: NK1-1
Treg
4.43
Comp-APC-A :: Foxp3
Comp-PE-A :: CD25
CD4 T cells
42.6
Q2
0.23
Q4
5.99
CD8 T cells
51.2
Comp-PE-Cy7-A :: CD4
Comp-PerCP-Cy5-5-A :: CD8
Blood_17_015.fcs
Ungated
311425

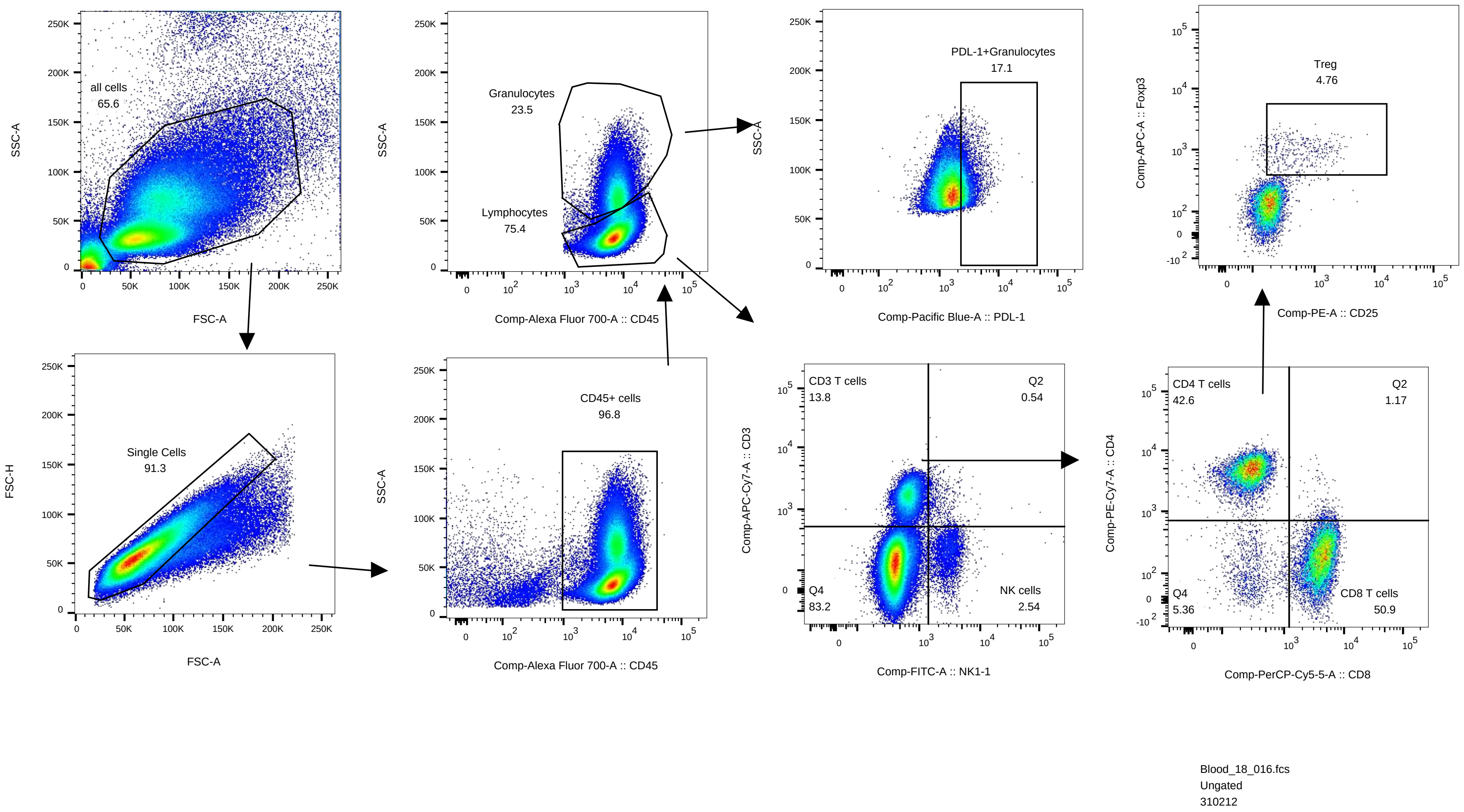
all cells
65.6
SSC-A
FSC-A
Single Cells
91.3
FSC-H
FSC-A
Granulocytes
23.5
Lymphocytes
75.4
SSC-A
Comp-Alexa Fluor 700-A :: CD45
CD45+ cells
96.8
SSC-A
Comp-Alexa Fluor 700-A :: CD45
PDL-1+Granulocytes
17.1
SSC-A
Comp-Pacific Blue-A :: PDL-1
CD3 T cells
13.8
Q2
0.54
Q4
83.2
NK cells
2.54
Comp-APC-Cy7-A :: CD3
Comp-FITC-A :: NK1-1
Treg
4.76
Comp-APC-A :: Foxp3
Comp-PE-A :: CD25
CD4 T cells
42.6
Q2
1.17
Q4
5.36
CD8 T cells
50.9
Comp-PE-Cy7-A :: CD4
Comp-PerCP-Cy5-5-A :: CD8
Blood_18_016.fcs
Ungated
310212

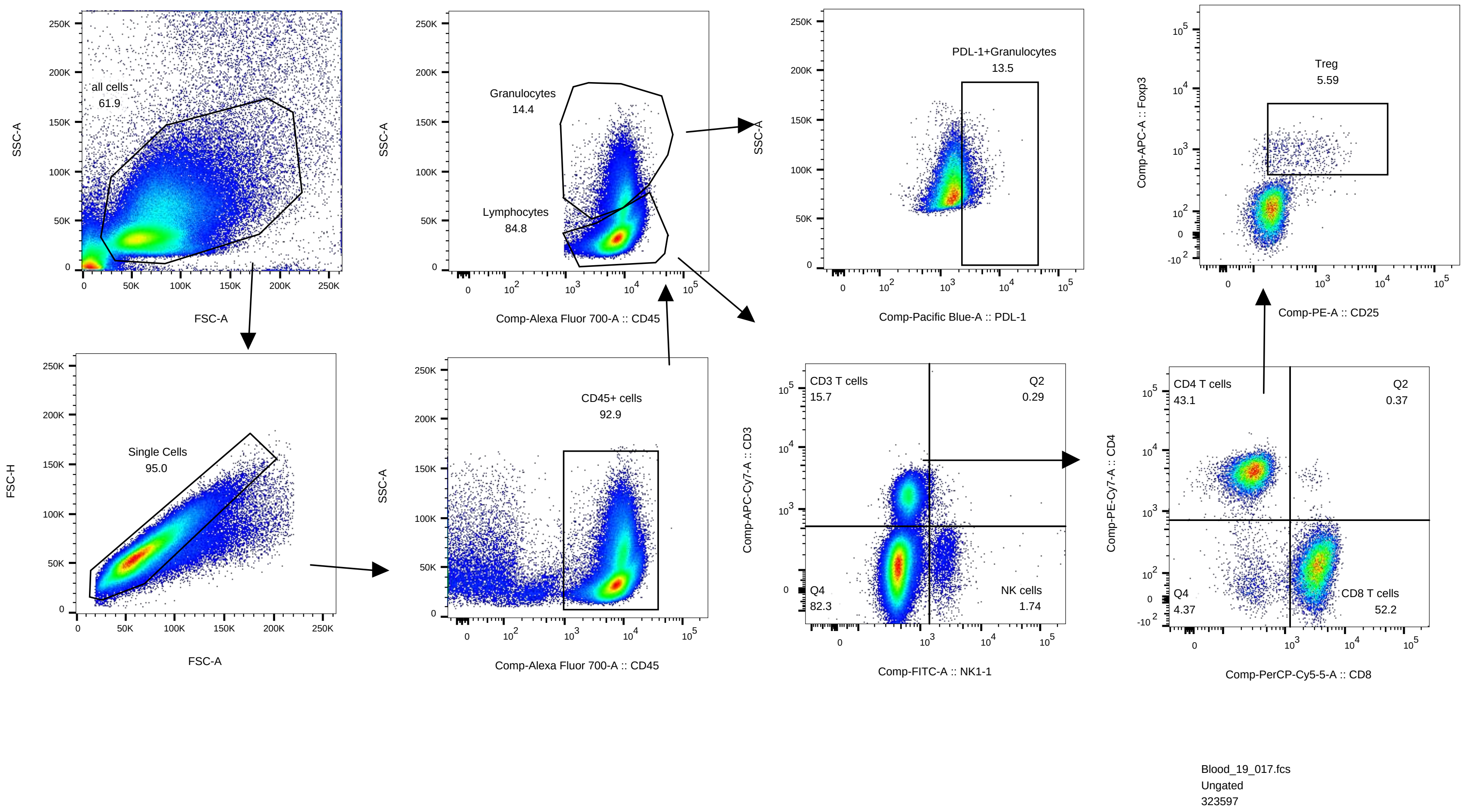

all cells
61.9
SSC-A
FSC-A
Single Cells
95.0
FSC-H
FSC-A
Granulocytes
14.4
Lymphocytes
84.8
SSC-A
Comp-Alexa Fluor 700-A :: CD45
CD45+ cells
92.9
SSC-A
Comp-Alexa Fluor 700-A :: CD45
PDL-1+Granulocytes
13.5
SSC-A
Comp-Pacific Blue-A :: PDL-1
CD3 T cells
15.7
Q2
0.29
Q4
82.3
NK cells
1.74
Comp-APC-Cy7-A :: CD3
Comp-FITC-A :: NK1-1
Treg
5.59
Comp-APC-A :: Foxp3
Comp-PE-A :: CD25
CD4 T cells
43.1
Q2
0.37
Q4
4.37
CD8 T cells
52.2
Comp-PE-Cy7-A :: CD4
Comp-PerCP-Cy5-5-A :: CD8
Blood_19_017.fcs
Ungated
323597

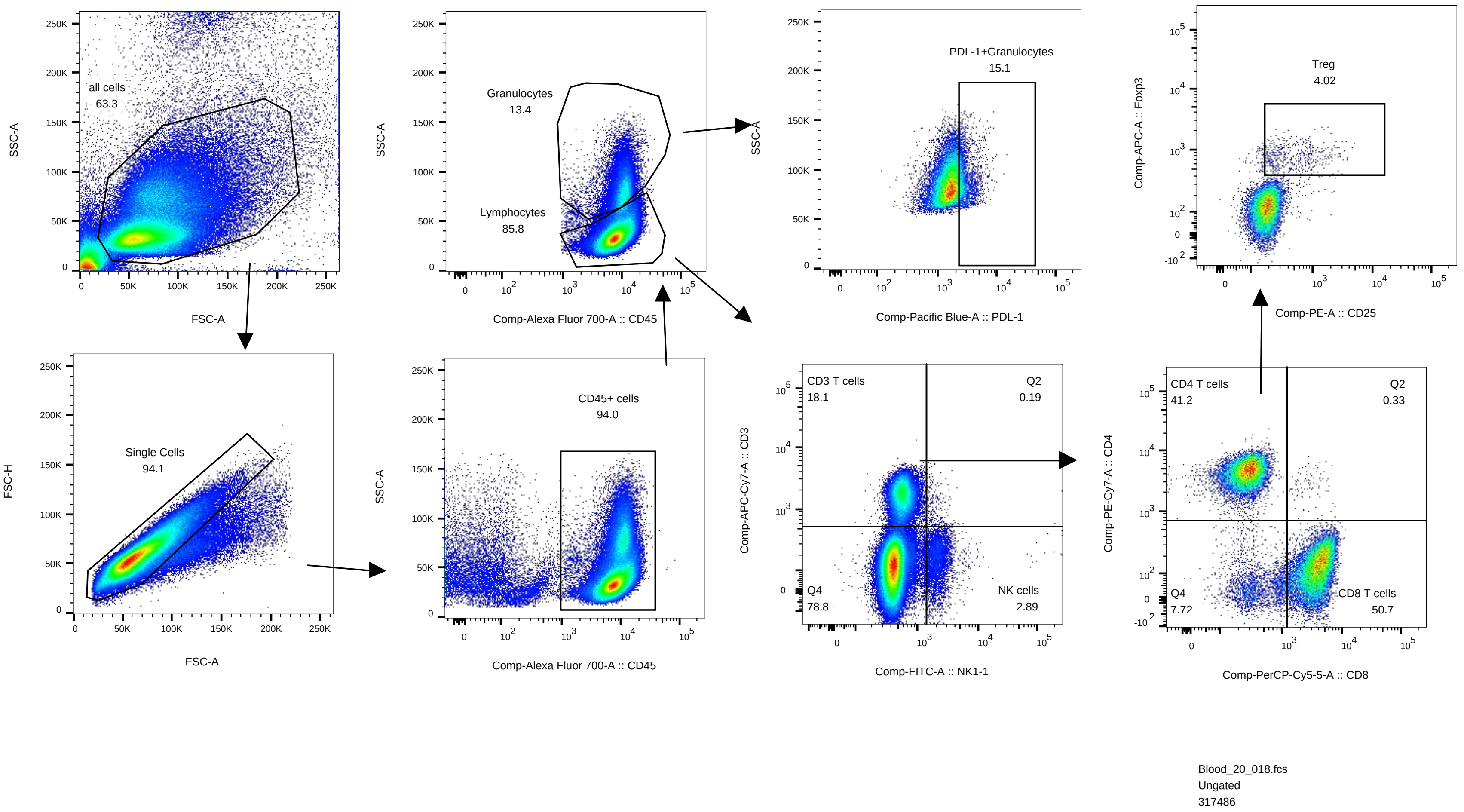
all cells
63.3
SSC-A
FSC-A
Single Cells
94.1
FSC-H
FSC-A
Granulocytes
13.4
Lymphocytes
85.8
SSC-A
Comp-Alexa Fluor 700-A :: CD45
CD45+ cells
94.0
SSC-A
Comp-Alexa Fluor 700-A :: CD45
PDL-1+Granulocytes
15.1
SSC-A
Comp-Pacific Blue-A :: PDL-1
CD3 T cells
18.1
Q2
0.19
Q4
78.8
NK cells
2.89
Comp-APC-Cy7-A :: CD3
Comp-FITC-A :: NK1-1
Treg
4.02
Comp-APC-A :: Foxp3
Comp-PE-A :: CD25
CD4 T cells
41.2
Q2
0.33
Q4
7.72
CD8 T cells
50.7
Comp-PE-Cy7-A :: CD4
Comp-PerCP-Cy5-5-A :: CD8
Blood_20_018.fcs
Ungated
317486

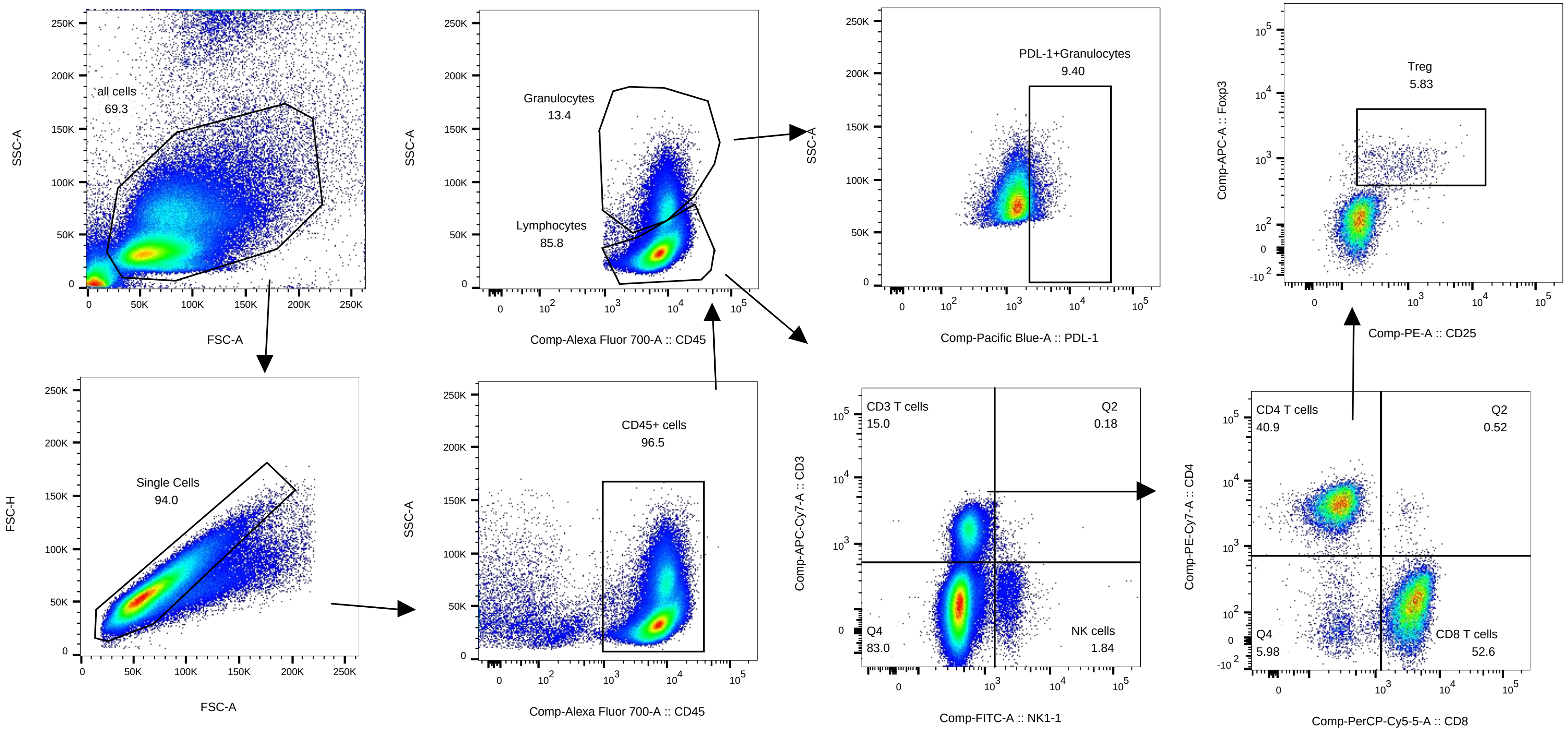

Blood_21_019.fcs
Ungated
290095

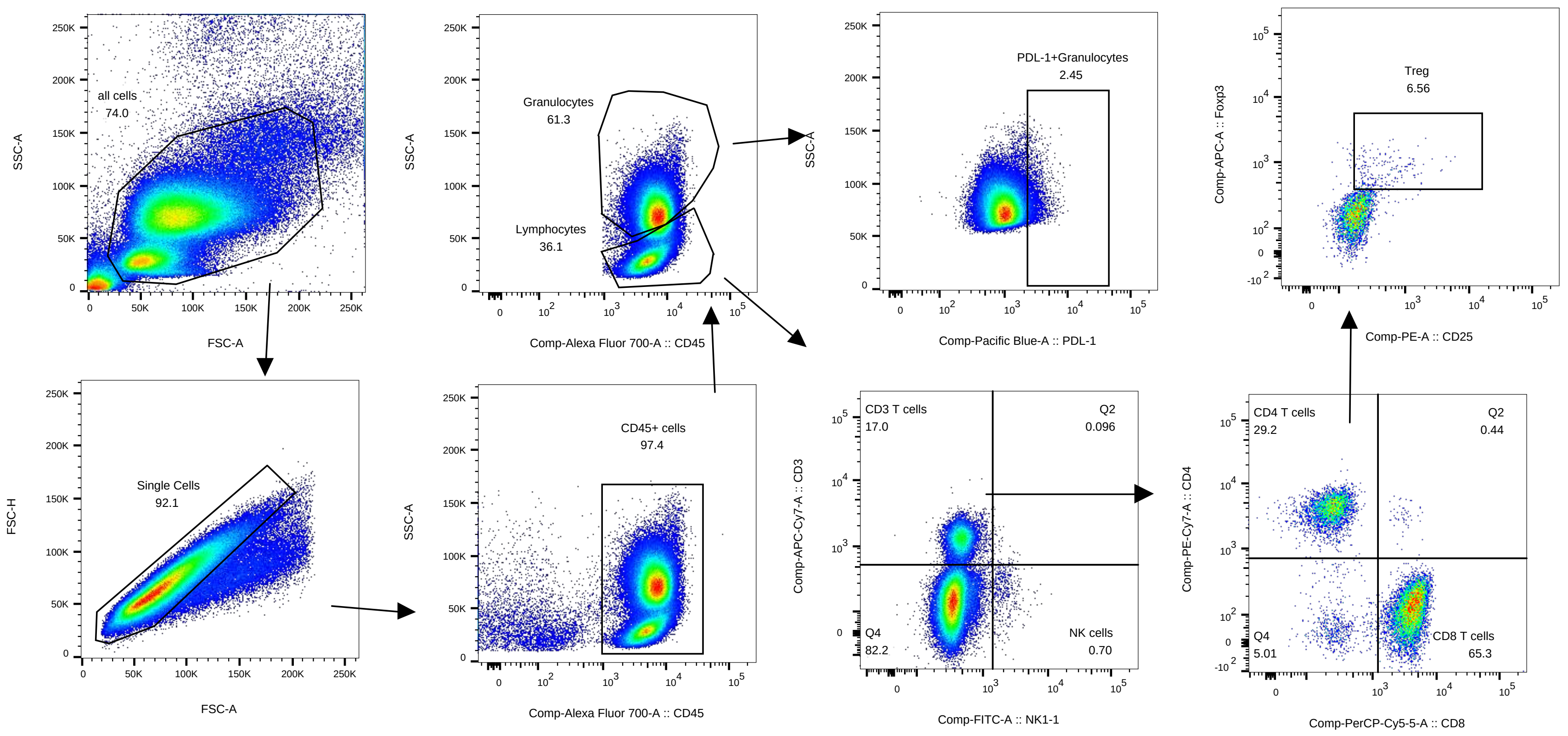

Blood_22_020.fcs
Ungated
279020

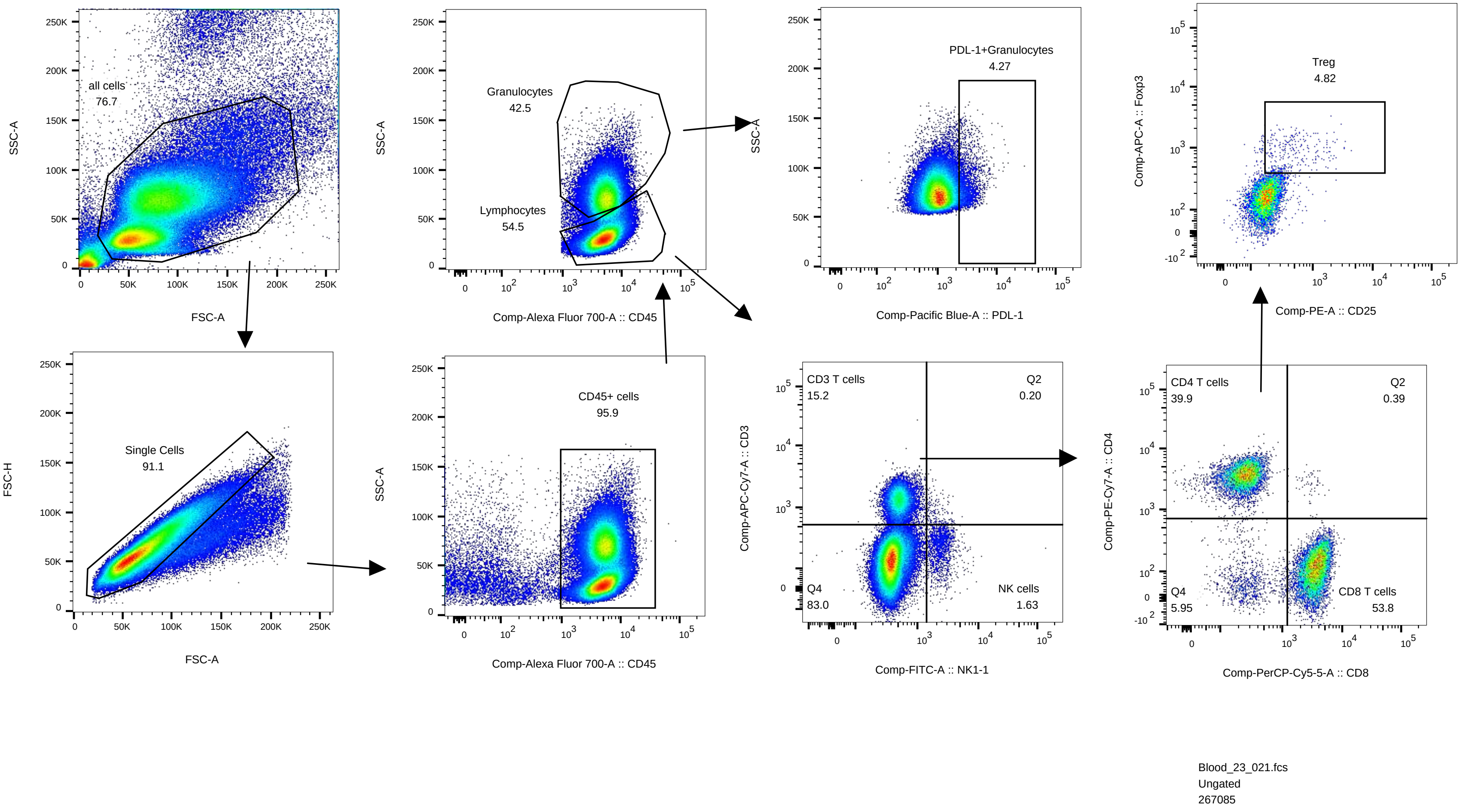

all cells
76.7
SSC-A
FSC-A
Single Cells
91.1
FSC-H
Granulocytes
42.5
Lymphocytes
54.5
Comp-Alexa Fluor 700-A :: CD45
CD45+ cells
95.9
PDL-1+Granulocytes
4.27
Comp-Pacific Blue-A :: PDL-1
CD3 T cells
15.2
Q2
0.20
Q4
83.0
NK cells
1.63
Comp-APC-Cy7-A :: CD3
Comp-FITC-A :: NK1-1
Treg
4.82
Comp-APC-A :: Foxp3
Comp-PE-A :: CD25
CD4 T cells
39.9
Q2
0.39
Q4
5.95
CD8 T cells
53.8
Comp-PE-Cy7-A :: CD4
Comp-PerCP-Cy5-5-A :: CD8
Blood_23_021.fcs
Ungated
267085

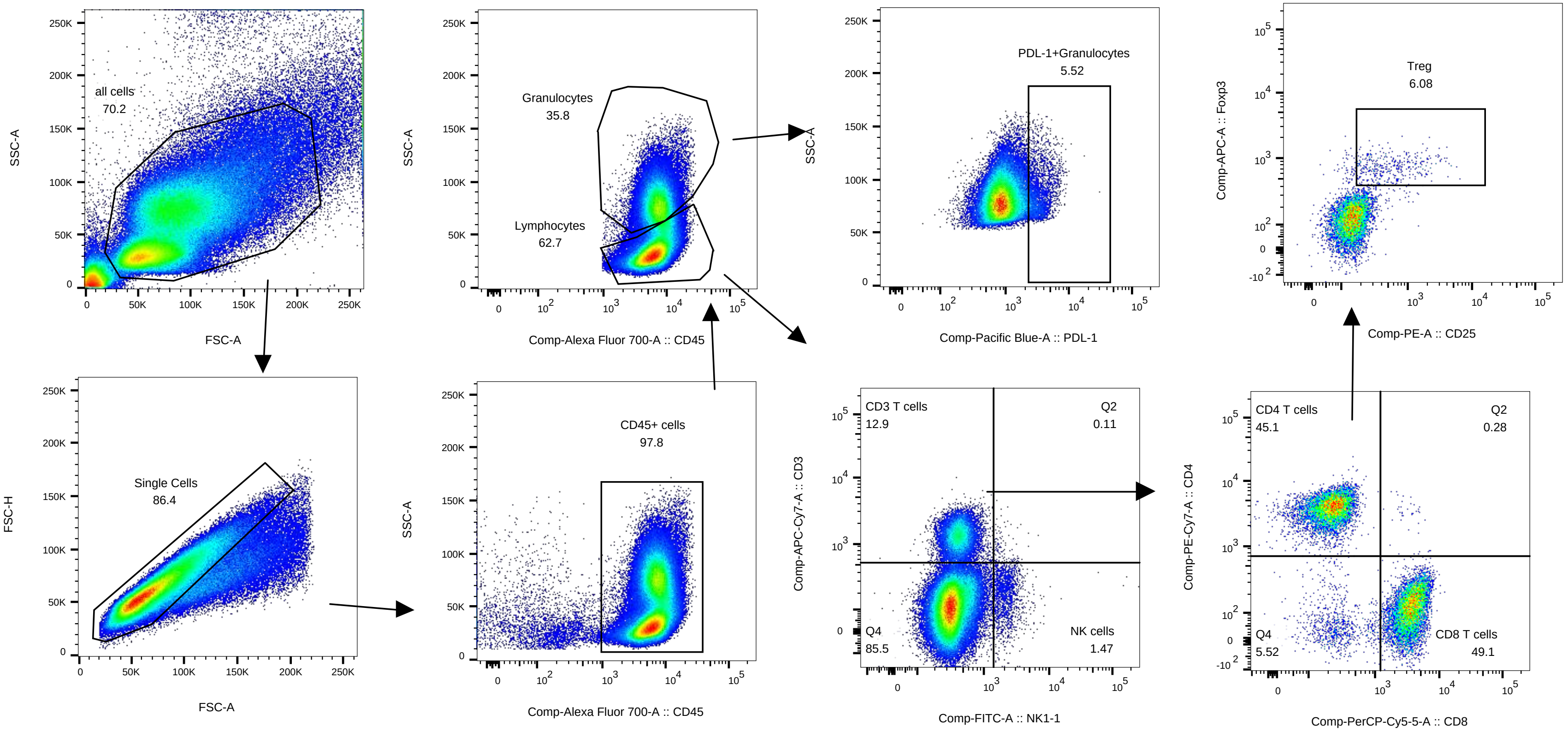

Blood_24_022.fcs
Ungated
297535

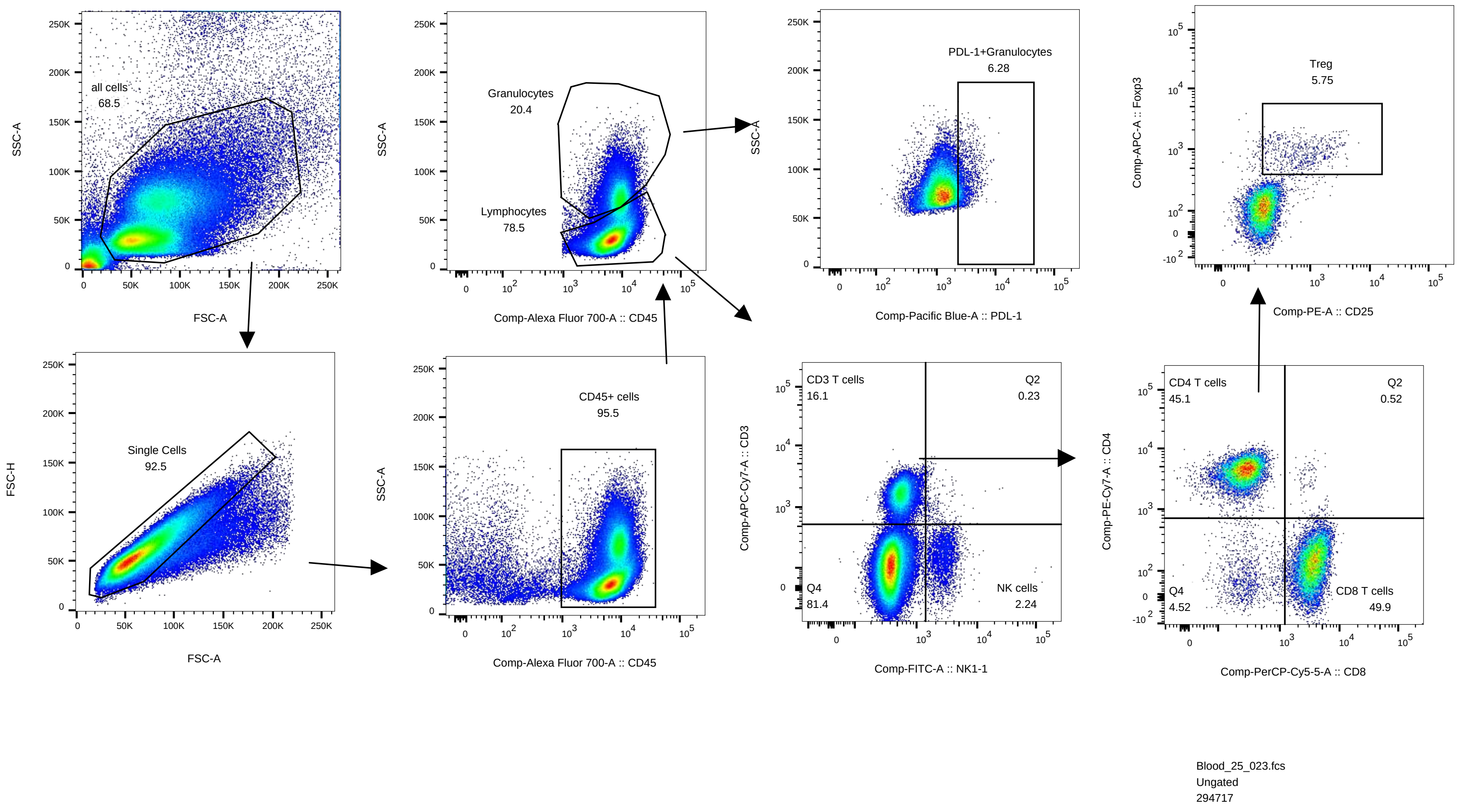
all cells
68.5
SSC-A
FSC-A
Single Cells
92.5
FSC-H
FSC-A
Granulocytes
20.4
Lymphocytes
78.5
SSC-A
Comp-Alexa Fluor 700-A :: CD45
CD45+ cells
95.5
SSC-A
Comp-Alexa Fluor 700-A :: CD45
PDL-1+Granulocytes
6.28
SSC-A
Comp-Pacific Blue-A :: PDL-1
CD3 T cells
16.1
Q2
0.23
Q4
81.4
NK cells
2.24
Comp-APC-Cy7-A :: CD3
Comp-FITC-A :: NK1-1
Treg
5.75
Comp-APC-A :: Foxp3
Comp-PE-A :: CD25
CD4 T cells
45.1
Q2
0.52
Q4
4.52
CD8 T cells
49.9
Comp-PE-Cy7-A :: CD4
Comp-PerCP-Cy5-5-A :: CD8
Blood_25_023.fcs
Ungated
294717

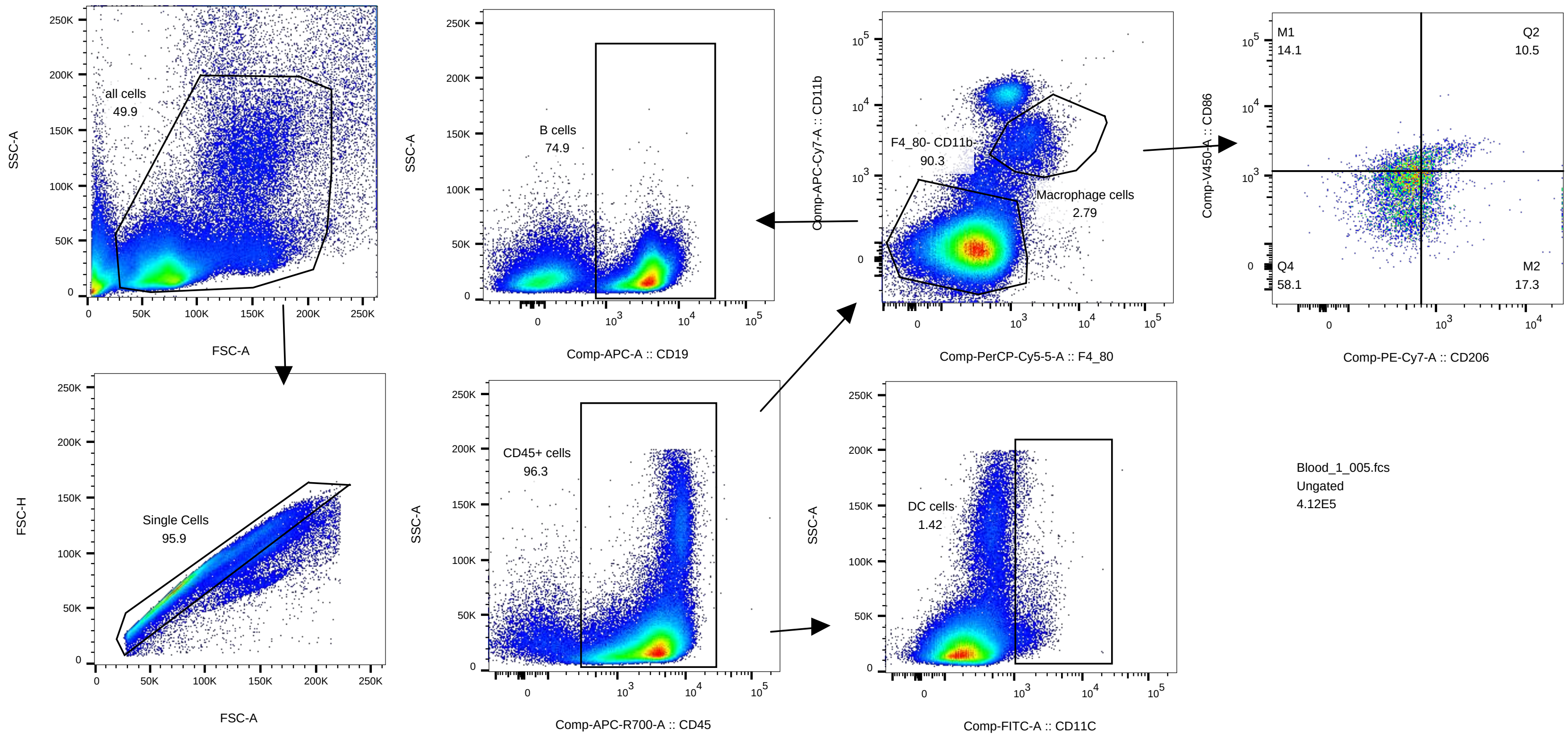
all cells
49.9
SSC-A
FSC-A
Single Cells
95.9
FSC-H
B cells
74.9
Comp-APC-A :: CD19
CD45+ cells
96.3
Comp-APC-R700-A :: CD45
F4_80- CD11b-
90.3
Macrophage cells
2.79
Comp-APC-Cy7-A :: CD11b
Comp-PerCP-Cy5-5-A :: F4_80
DC cells
1.42
Comp-FITC-A :: CD11C
M1
14.1
Q2
10.5
Q4
58.1
M2
17.3
Comp-V450-A :: CD86
Comp-PE-Cy7-A :: CD206
Blood_1_005.fcs
Ungated
4.12E5

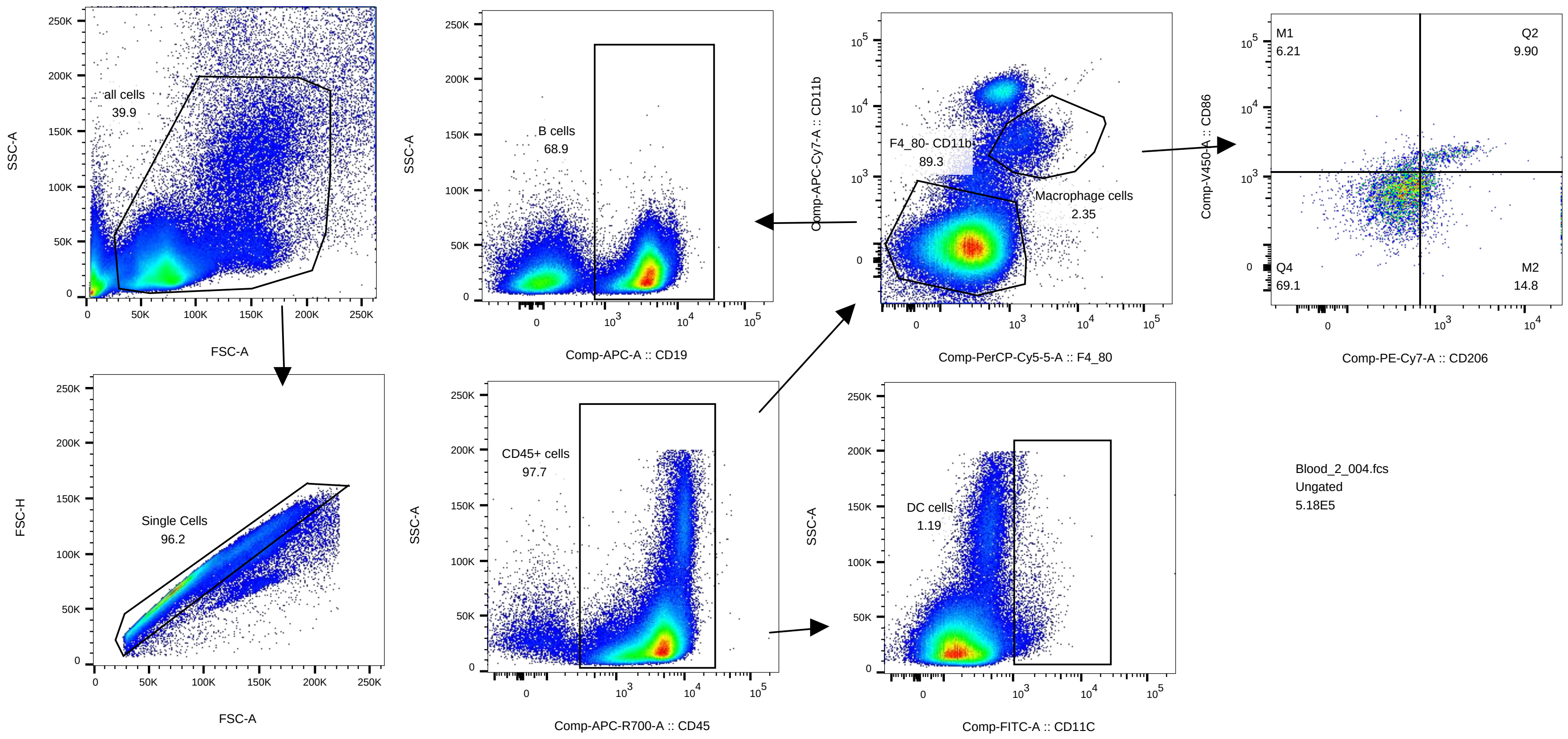
all cells
39.9
SSC-A
FSC-A
Single Cells
96.2
FSC-H
FSC-A
B cells
68.9
SSC-A
Comp-APC-A :: CD19
CD45+ cells
97.7
SSC-A
Comp-APC-R700-A :: CD45
F4_80- CD11b
89.3
Macrophage cells
2.35
Comp-APC-Cy7-A :: CD11b
Comp-PerCP-Cy5-5-A :: F4_80
DC cells
1.19
SSC-A
Comp-FITC-A :: CD11C
M1
6.21
Q2
9.90
Q4
69.1
M2
14.8
Comp-V450-A :: CD86
Comp-PE-Cy7-A :: CD206
Blood_2_004.fcs
Ungated
5.18E5

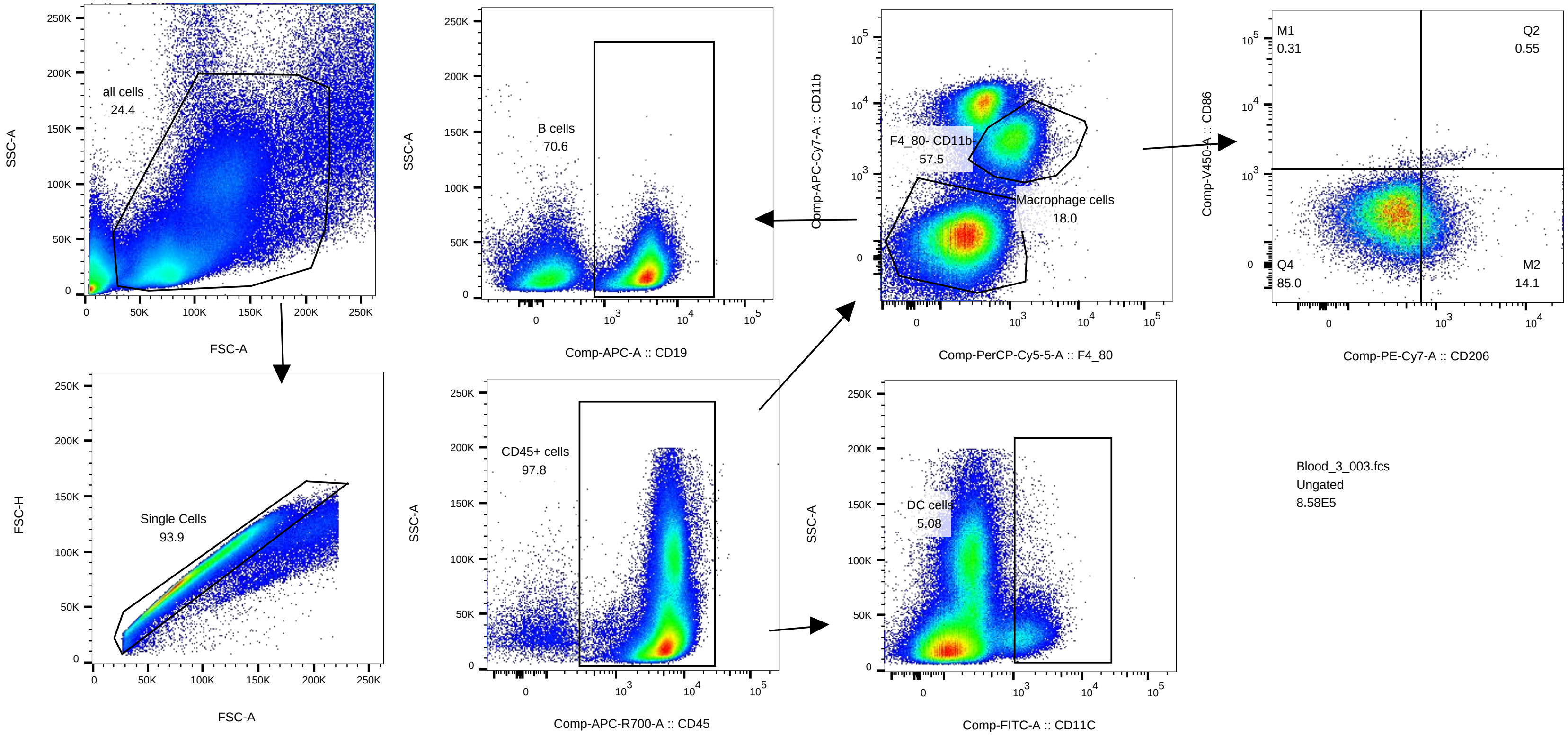
all cells
24.4
SSC-A
FSC-A
Single Cells
93.9
FSC-H
FSC-A
B cells
70.6
SSC-A
Comp-APC-A :: CD19
CD45+ cells
97.8
SSC-A
Comp-APC-R700-A :: CD45
F4_80- CD11b-
57.5
Macrophage cells
18.0
Comp-APC-Cy7-A :: CD11b
Comp-PerCP-Cy5-5-A :: F4_80
DC cells
5.08
SSC-A
Comp-FITC-A :: CD11C
M1
0.31
Q2
0.55
Q4
85.0
M2
14.1
Comp-V450-A :: CD86
Comp-PE-Cy7-A :: CD206
Blood_3_003.fcs
Ungated
8.58E5

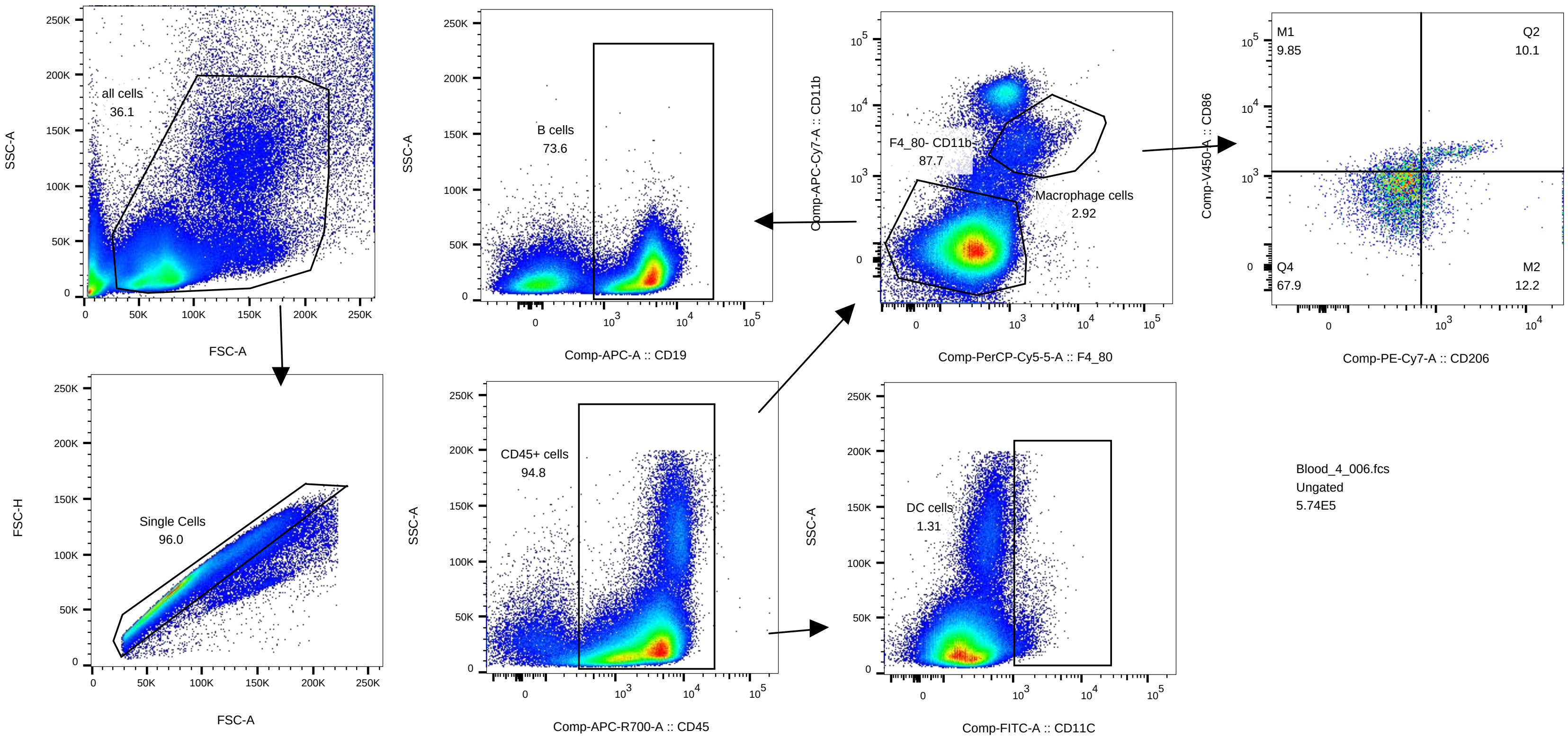
all cells
36.1
SSC-A
FSC-A
Single Cells
96.0
FSC-H
B cells
73.6
Comp-APC-A :: CD19
CD45+ cells
94.8
Comp-APC-R700-A :: CD45
F4_80- CD11b-
87.7
Macrophage cells
2.92
Comp-APC-Cy7-A :: CD11b
Comp-PerCP-Cy5-5-A :: F4_80
DC cells
1.31
Comp-FITC-A :: CD11C
M1
9.85
Q2
10.1
Q4
67.9
M2
12.2
Comp-V450-A :: CD86
Comp-PE-Cy7-A :: CD206
Blood_4_006.fcs
Ungated
5.74E5

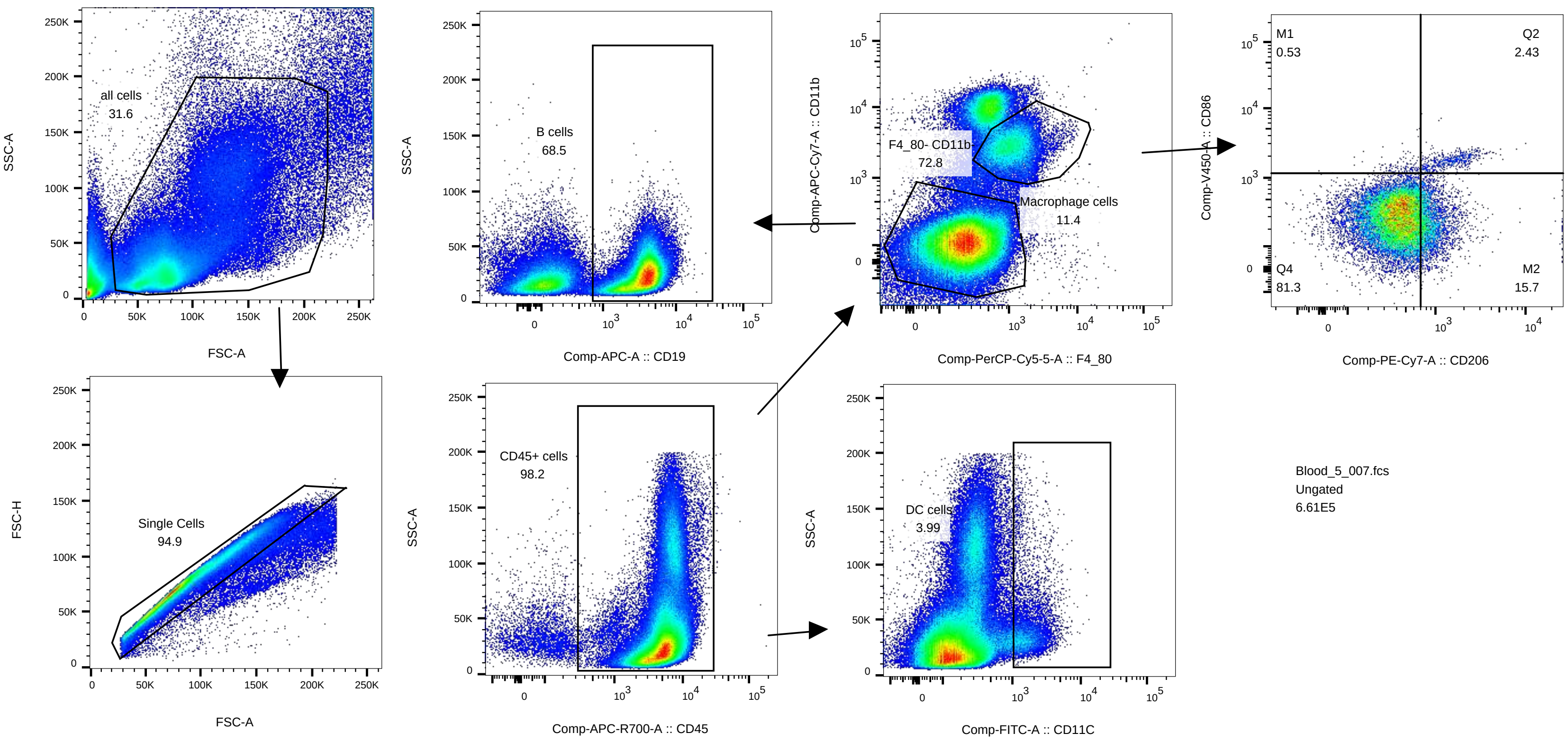

all cells
31.6
SSC-A
FSC-A
Single Cells
94.9
FSC-H
FSC-A
B cells
68.5
SSC-A
Comp-APC-A :: CD19
CD45+ cells
98.2
SSC-A
Comp-APC-R700-A :: CD45
F4_80- CD11b-
72.8
Macrophage cells
11.4
Comp-APC-Cy7-A :: CD11b
Comp-PerCP-Cy5-5-A :: F4_80
DC cells
3.99
SSC-A
Comp-FITC-A :: CD11C
M1
0.53
Q2
2.43
Q4
81.3
M2
15.7
Comp-V450-A :: CD86
Comp-PE-Cy7-A :: CD206
Blood_5_007.fcs
Ungated
6.61E5

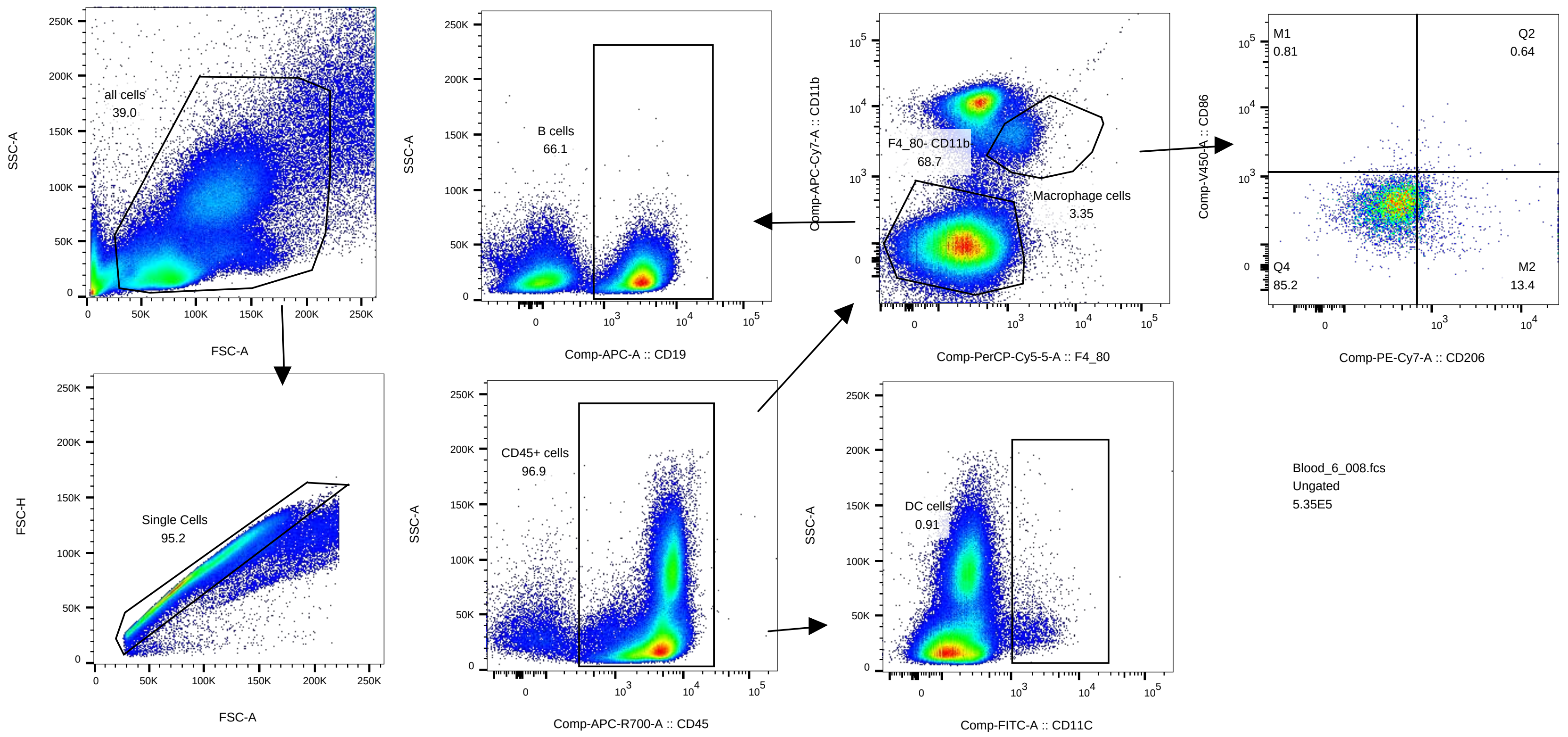

all cells
39.0
SSC-A
FSC-A
Single Cells
95.2
FSC-H
FSC-A
B cells
66.1
SSC-A
Comp-APC-A :: CD19
CD45+ cells
96.9
SSC-A
Comp-APC-R700-A :: CD45
F4_80- CD11b
68.7
Macrophage cells
3.35
Comp-APC-Cy7-A :: CD11b
Comp-PerCP-Cy5-5-A :: F4_80
DC cells
0.91
SSC-A
Comp-FITC-A :: CD11C
M1
0.81
Q2
0.64
Q4
85.2
M2
13.4
Comp-V450-A :: CD86
Comp-PE-Cy7-A :: CD206
Blood_6_008.fcs
Ungated
5.35E5

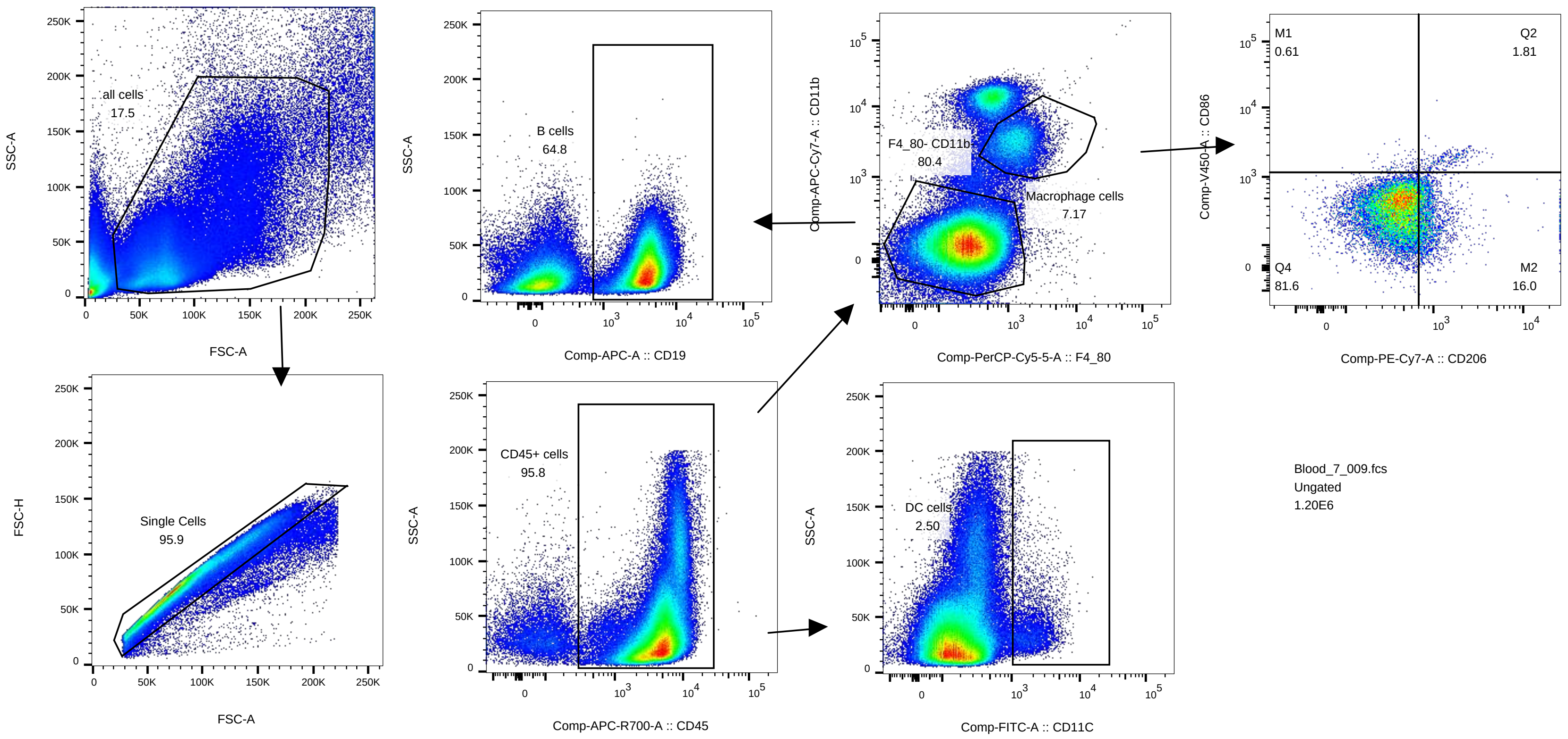
all cells
17.5
SSC-A
FSC-A
Single Cells
95.9
FSC-H
FSC-A
B cells
64.8
SSC-A
Comp-APC-A :: CD19
CD45+ cells
95.8
SSC-A
Comp-APC-R700-A :: CD45
F4_80- CD11b
80.4
Macrophage cells
7.17
Comp-APC-Cy7-A :: CD11b
Comp-PerCP-Cy5-5-A :: F4_80
DC cells
2.50
SSC-A
Comp-FITC-A :: CD11C
M1
0.61
Q2
1.81
Q4
81.6
M2
16.0
Comp-V450-A :: CD86
Comp-PE-Cy7-A :: CD206
Blood_7_009.fcs
Ungated
1.20E6

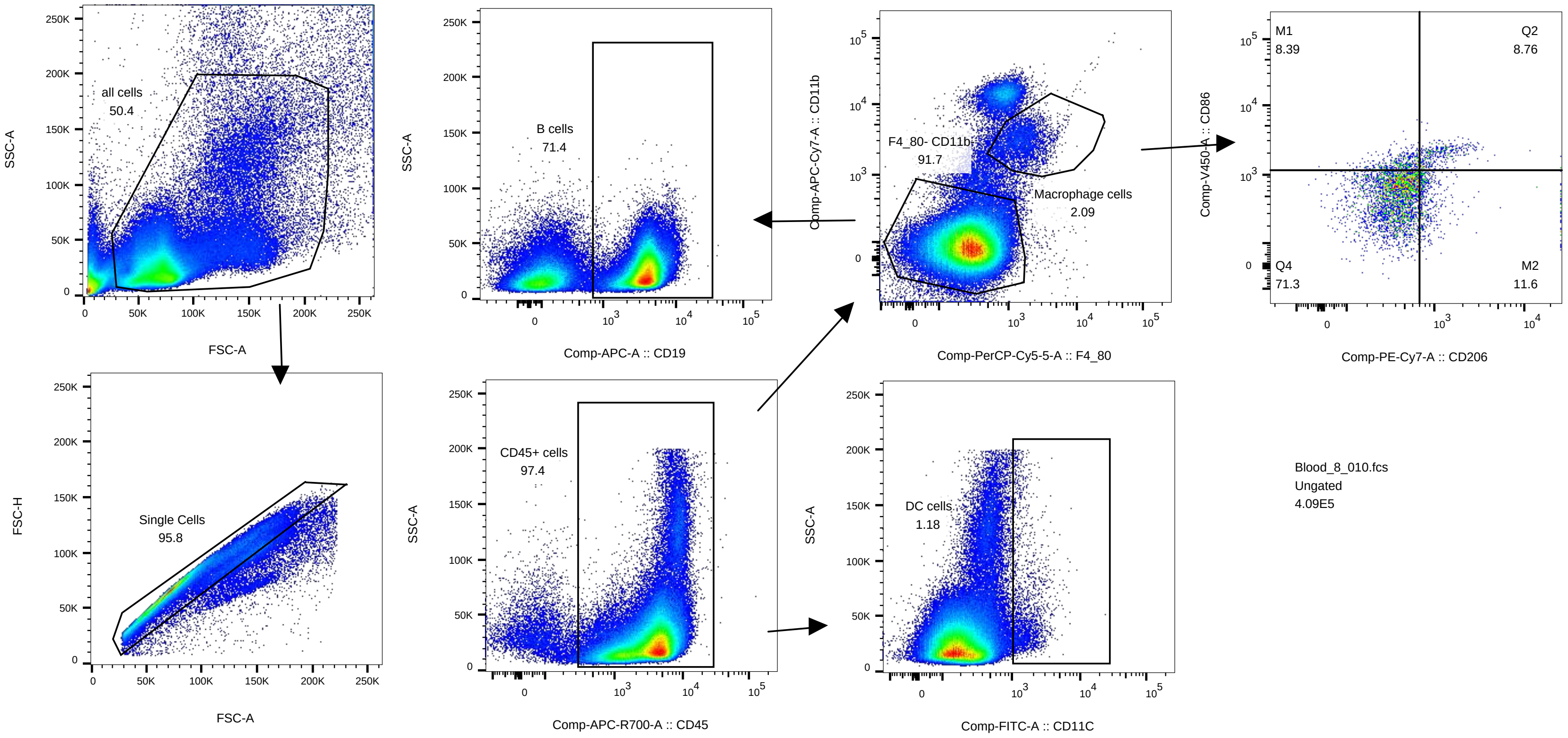

all cells
50.4
SSC-A
FSC-A
Single Cells
95.8
FSC-H
B cells
71.4
Comp-APC-A :: CD19
CD45+ cells
97.4
Comp-APC-R700-A :: CD45
F4_80- CD11b
91.7
Macrophage cells
2.09
Comp-APC-Cy7-A :: CD11b
Comp-PerCP-Cy5-5-A :: F4_80
DC cells
1.18
Comp-FITC-A :: CD11C
M1
8.39
Q2
8.76
Q4
71.3
M2
11.6
Comp-V450-A :: CD86
Comp-PE-Cy7-A :: CD206
Blood_8_010.fcs
Ungated
4.09E5

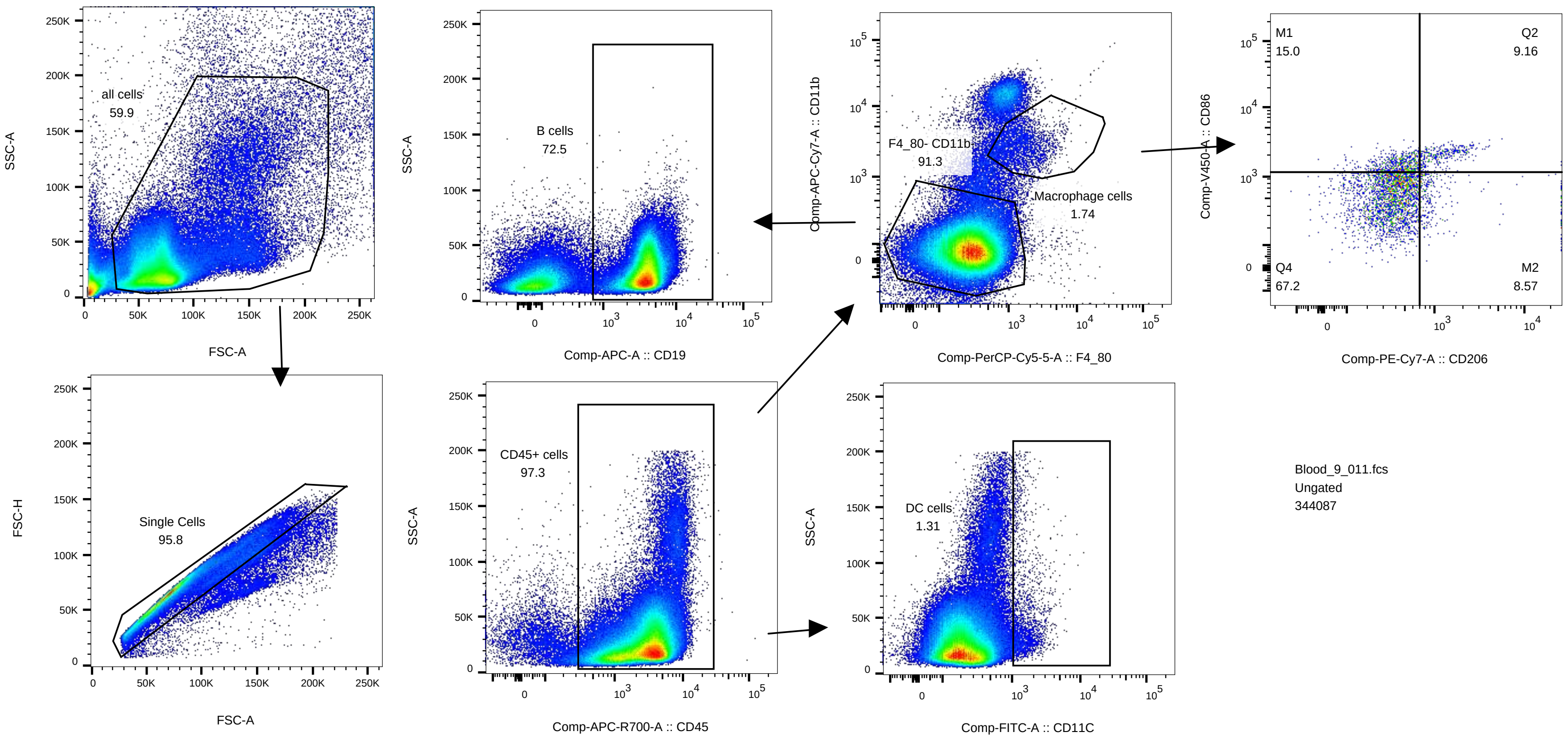
all cells
59.9
SSC-A
FSC-A
B cells
72.5
Comp-APC-A :: CD19
F4_80- CD11b
91.3
Macrophage cells
1.74
Comp-APC-Cy7-A :: CD11b
Comp-PerCP-Cy5-5-A :: F4_80
M1
15.0
Q2
9.16
Q4
67.2
M2
8.57
Comp-V450-A :: CD86
Comp-PE-Cy7-A :: CD206
Single Cells
95.8
FSC-H
CD45+ cells
97.3
Comp-APC-R700-A :: CD45
DC cells
1.31
Comp-FITC-A :: CD11C
Blood_9_011.fcs
Ungated
344087

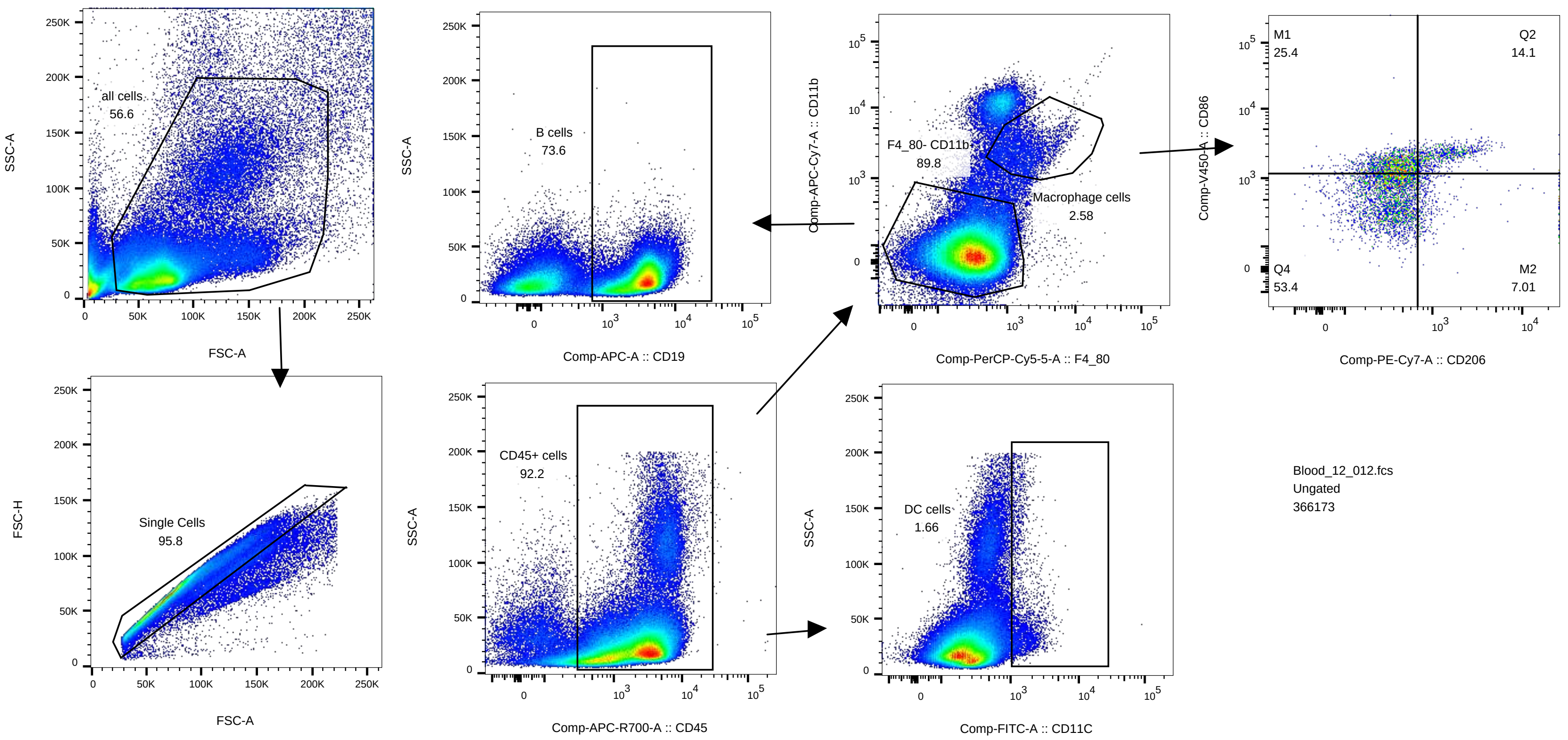
all cells
56.6
SSC-A
FSC-A
Single Cells
95.8
FSC-H
FSC-A
B cells
73.6
SSC-A
Comp-APC-A :: CD19
CD45+ cells
92.2
SSC-A
Comp-APC-R700-A :: CD45
F4_80- CD11b
89.8
Macrophage cells
2.58
Comp-APC-Cy7-A :: CD11b
Comp-PerCP-Cy5-5-A :: F4_80
DC cells
1.66
SSC-A
Comp-FITC-A :: CD11C
M1
25.4
Q2
14.1
Q4
53.4
M2
7.01
Comp-V450-A :: CD86
Comp-PE-Cy7-A :: CD206
Blood_12_012.fcs
Ungated
366173

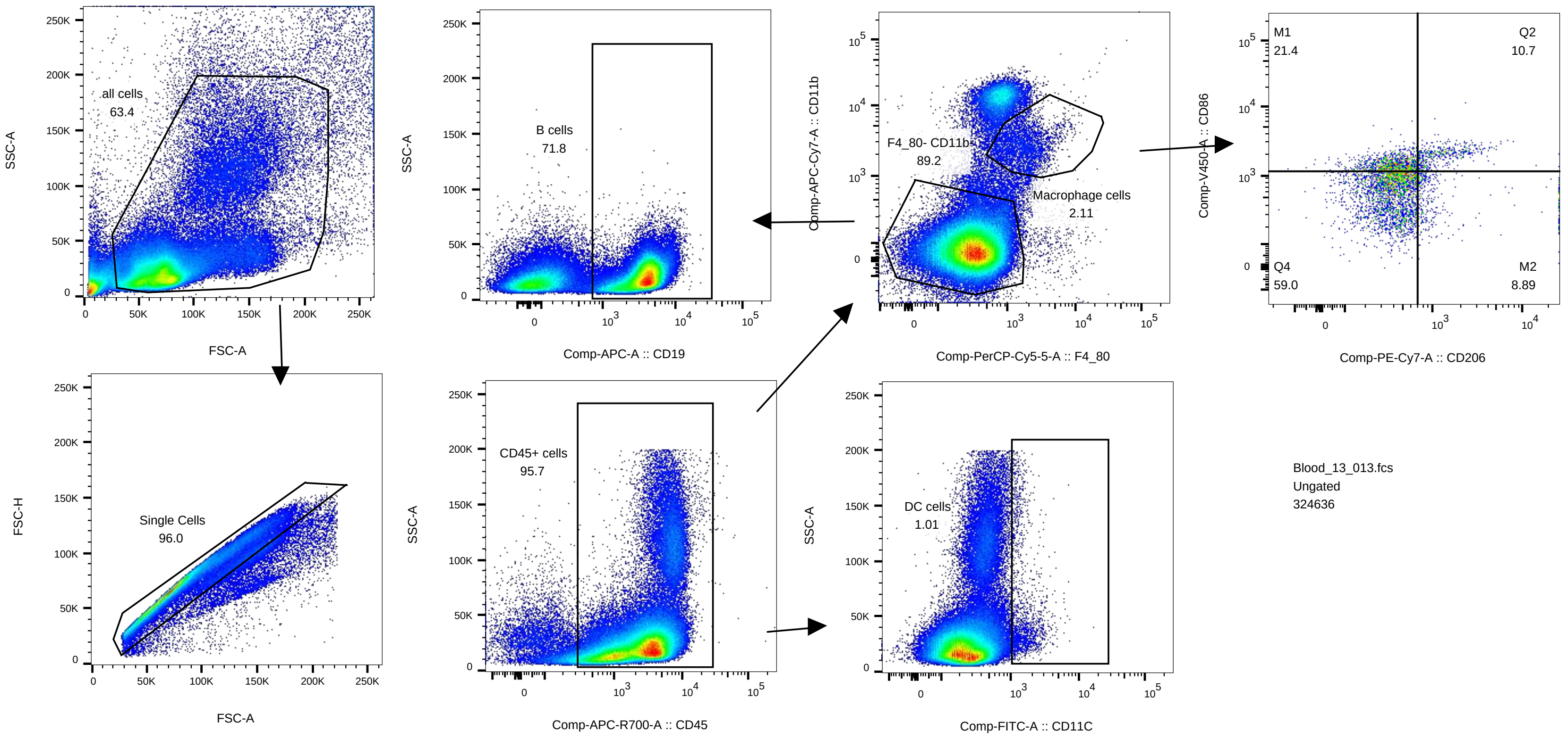

all cells
63.4
SSC-A
FSC-A
Single Cells
96.0
FSC-H
B cells
71.8
Comp-APC-A :: CD19
CD45+ cells
95.7
Comp-APC-R700-A :: CD45
F4_80- CD11b-
89.2
Macrophage cells
2.11
Comp-APC-Cy7-A :: CD11b
Comp-PerCP-Cy5-5-A :: F4_80
DC cells
1.01
Comp-FITC-A :: CD11C
M1
21.4
Q2
10.7
Q4
59.0
M2
8.89
Comp-V450-A :: CD86
Comp-PE-Cy7-A :: CD206
Blood_13_013.fcs
Ungated
324636

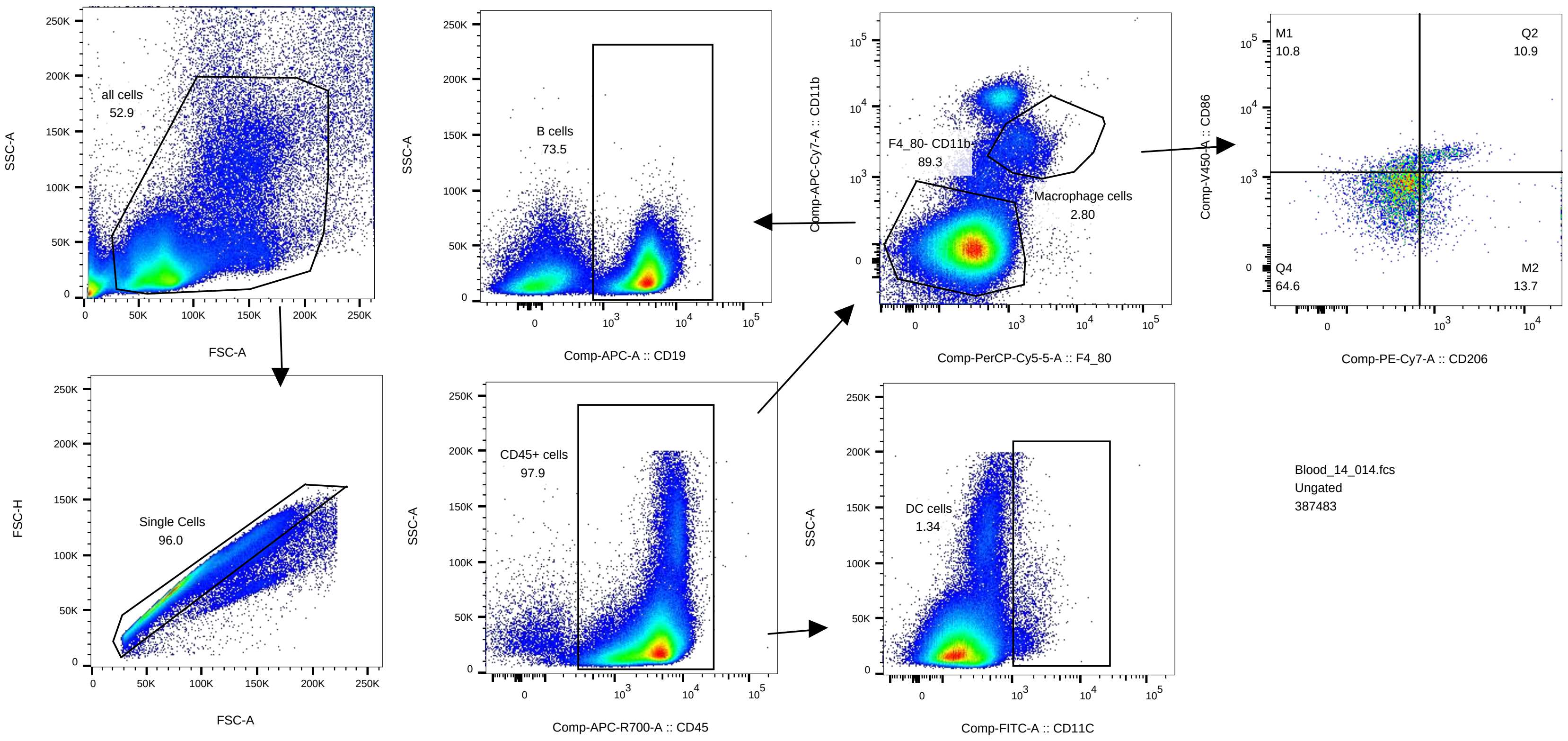
all cells
52.9
SSC-A
FSC-A
Single Cells
96.0
FSC-H
FSC-A
B cells
73.5
SSC-A
Comp-APC-A :: CD19
CD45+ cells
97.9
SSC-A
Comp-APC-R700-A :: CD45
F4_80- CD11b
89.3
Macrophage cells
2.80
Comp-APC-Cy7-A :: CD11b
Comp-PerCP-Cy5-5-A :: F4_80
DC cells
1.34
SSC-A
Comp-FITC-A :: CD11C
M1
10.8
Q2
10.9
Q4
64.6
M2
13.7
Comp-V450-A :: CD86
Comp-PE-Cy7-A :: CD206
Blood_14_014.fcs
Ungated
387483

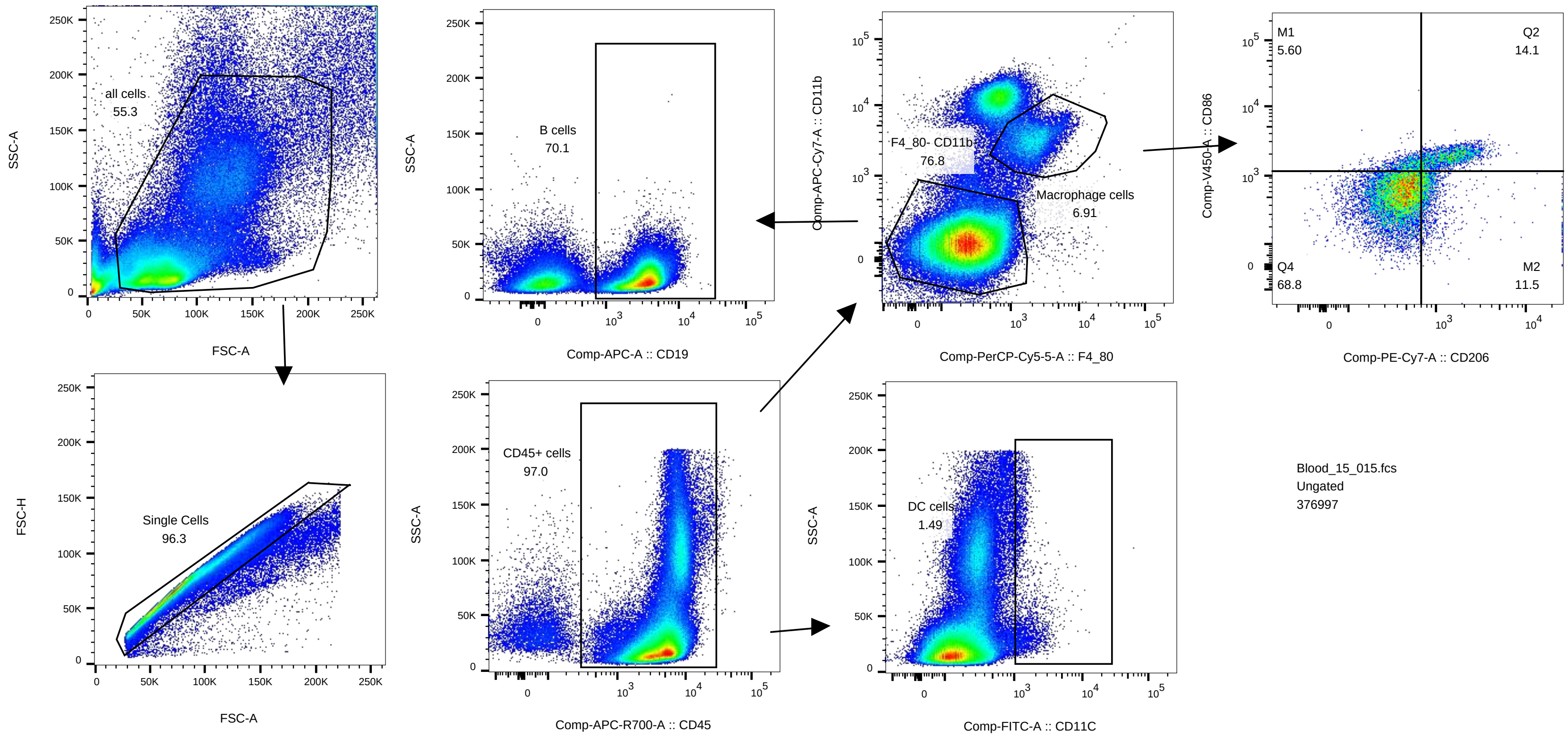
all cells
55.3
SSC-A
FSC-A
Single Cells
96.3
FSC-H
FSC-A
B cells
70.1
SSC-A
Comp-APC-A :: CD19
CD45+ cells
97.0
SSC-A
Comp-APC-R700-A :: CD45
F4_80- CD11b
76.8
Macrophage cells
6.91
Comp-APC-Cy7-A :: CD11b
Comp-PerCP-Cy5-5-A :: F4_80
DC cells
1.49
SSC-A
Comp-FITC-A :: CD11C
M1
5.60
Q2
14.1
Q4
68.8
M2
11.5
Comp-V450-A :: CD86
Comp-PE-Cy7-A :: CD206
Blood_15_015.fcs
Ungated
376997

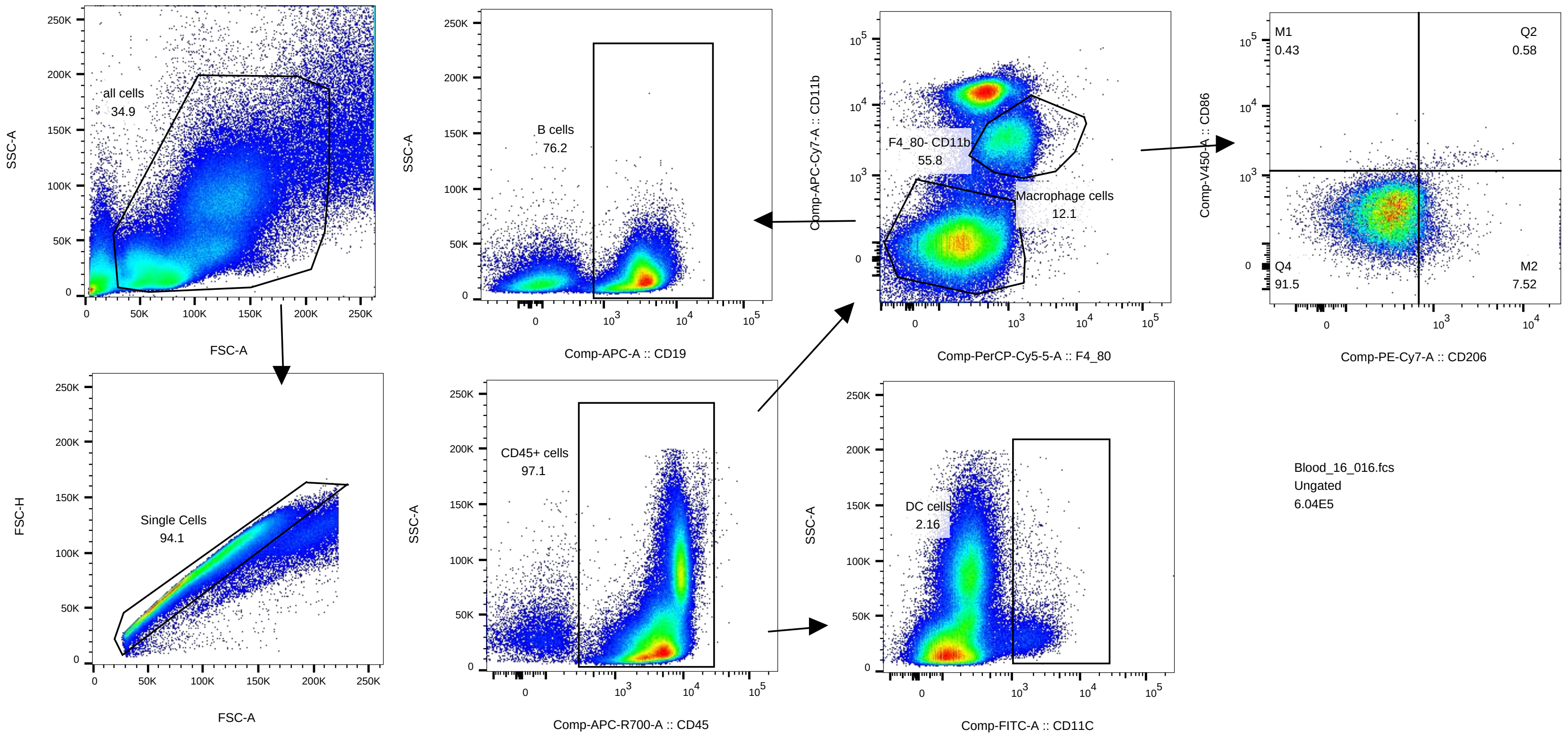
all cells
34.9
SSC-A
FSC-A
Single Cells
94.1
FSC-H
B cells
76.2
Comp-APC-A :: CD19
CD45+ cells
97.1
Comp-APC-R700-A :: CD45
Comp-APC-Cy7-A :: CD11b
F4_80- CD11b
55.8
Macrophage cells
12.1
Comp-PerCP-Cy5-5-A :: F4_80
DC cells
2.16
Comp-FITC-A :: CD11C
Comp-V450-A :: CD86
M1
0.43
Q2
0.58
Q4
91.5
M2
7.52
Comp-PE-Cy7-A :: CD206
Blood_16_016.fcs
Ungated
6.04E5

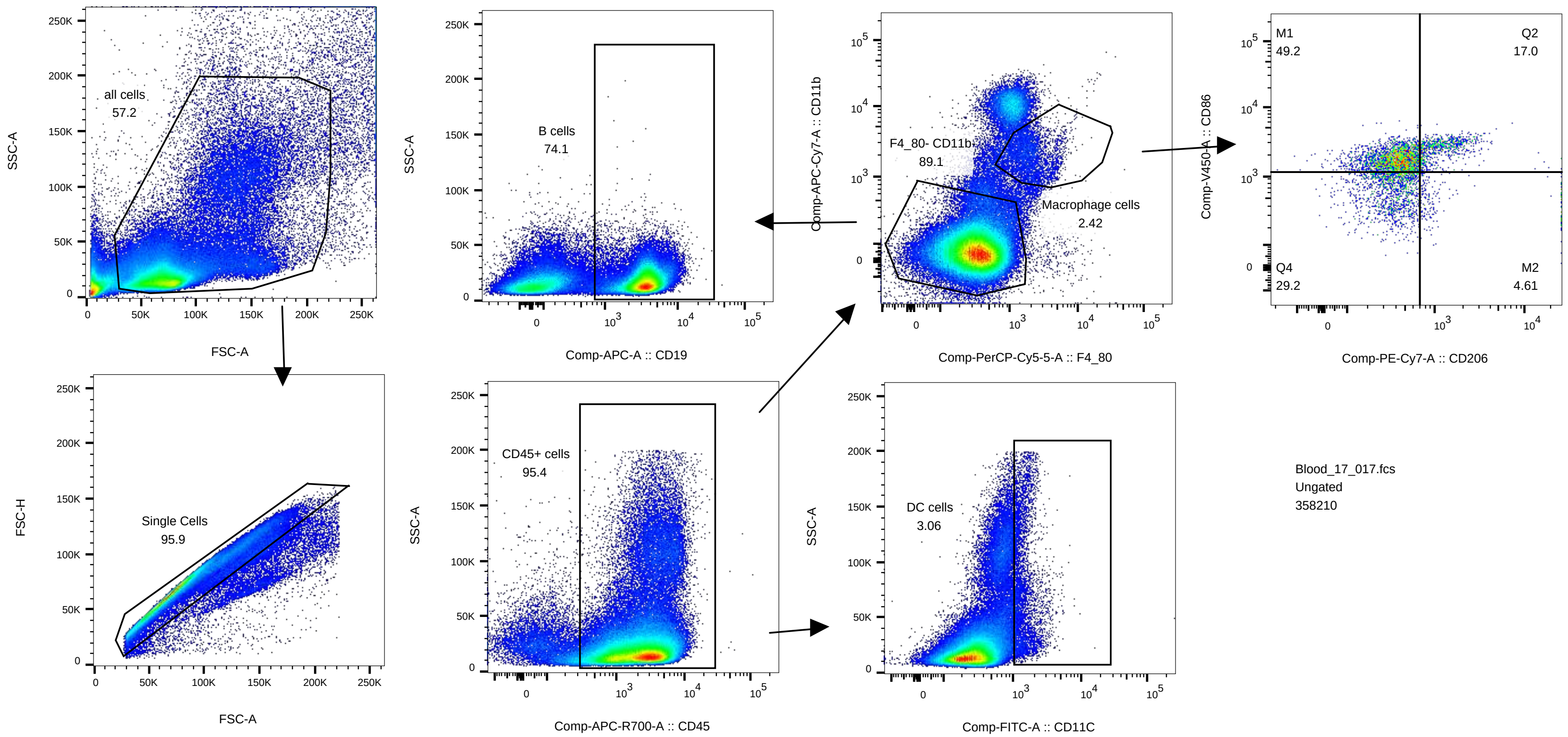

all cells
57.2
SSC-A
FSC-A
Single Cells
95.9
FSC-H
B cells
74.1
Comp-APC-A :: CD19
CD45+ cells
95.4
Comp-APC-R700-A :: CD45
F4_80- CD11b
89.1
Macrophage cells
2.42
Comp-APC-Cy7-A :: CD11b
Comp-PerCP-Cy5-5-A :: F4_80
DC cells
3.06
Comp-FITC-A :: CD11C
M1
49.2
Q2
17.0
Q4
29.2
M2
4.61
Comp-V450-A :: CD86
Comp-PE-Cy7-A :: CD206
Blood_17_017.fcs
Ungated
358210

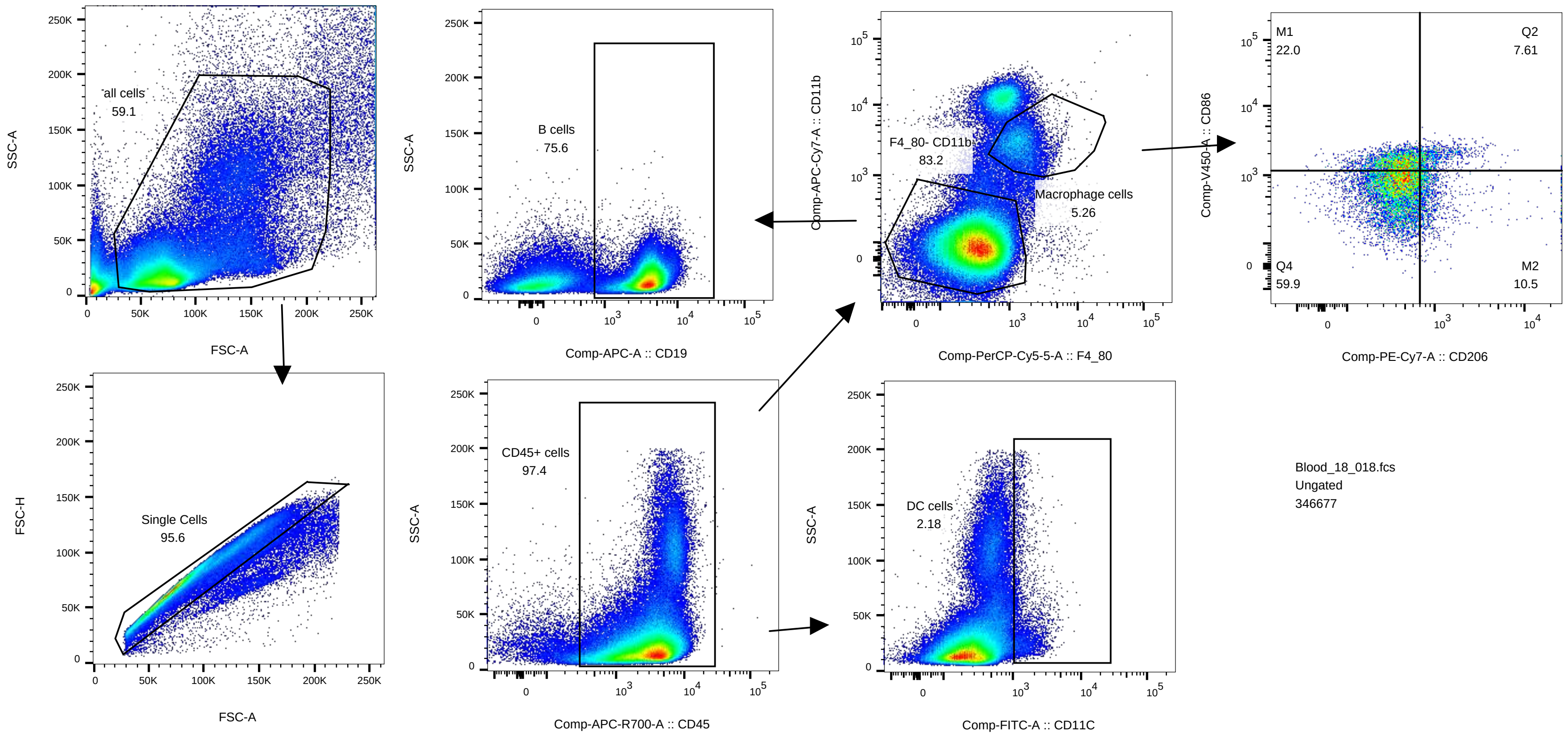
all cells
59.1
SSC-A
FSC-A
Single Cells
95.6
FSC-H
B cells
75.6
Comp-APC-A :: CD19
CD45+ cells
97.4
Comp-APC-R700-A :: CD45
F4_80- CD11b
83.2
Macrophage cells
5.26
Comp-APC-Cy7-A :: CD11b
Comp-PerCP-Cy5-5-A :: F4_80
DC cells
2.18
Comp-FITC-A :: CD11C
M1
22.0
Q2
7.61
Q4
59.9
M2
10.5
Comp-V450-A :: CD86
Comp-PE-Cy7-A :: CD206
Blood_18_018.fcs
Ungated
346677

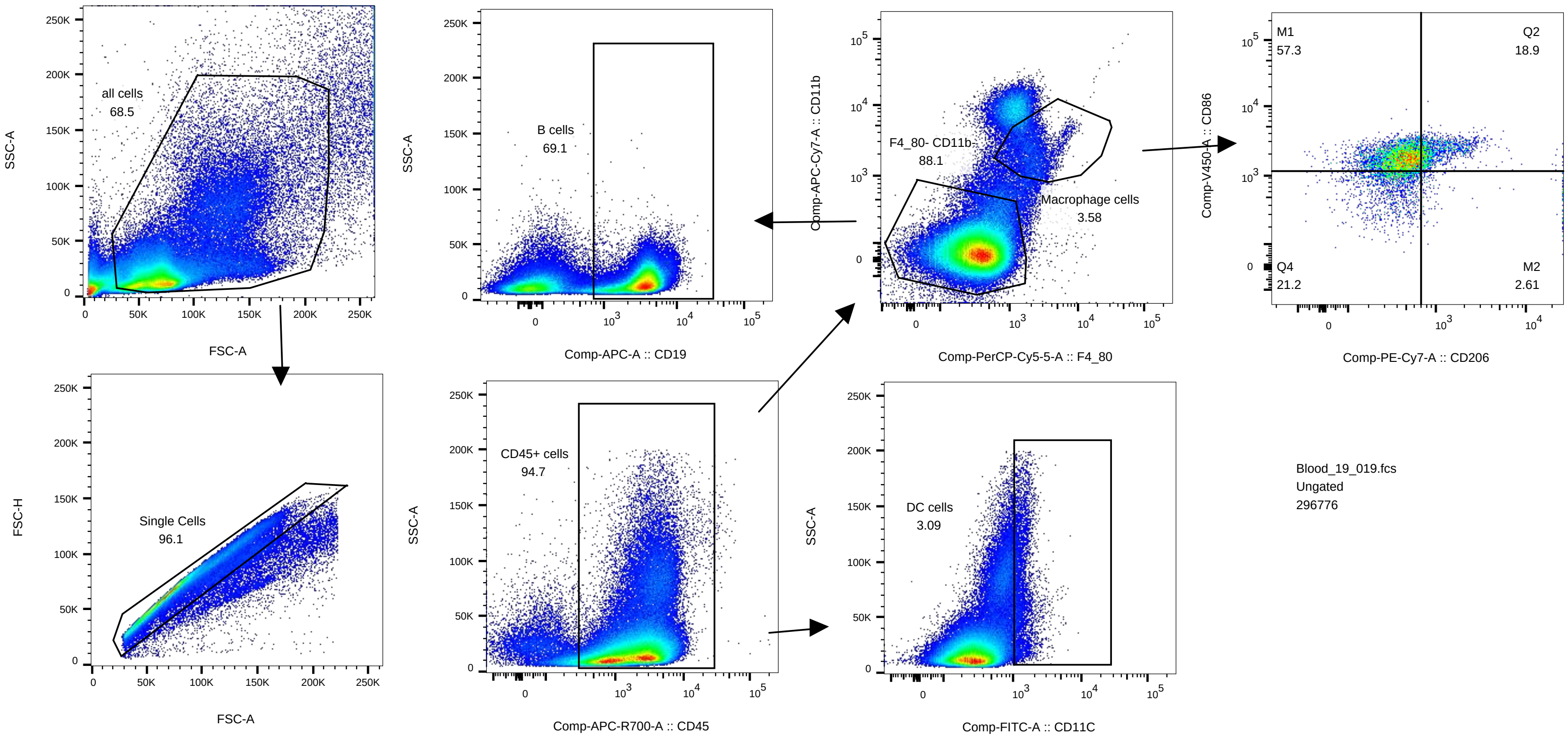
all cells
68.5
SSC-A
FSC-A
Single Cells
96.1
FSC-H
FSC-A
B cells
69.1
SSC-A
Comp-APC-A :: CD19
CD45+ cells
94.7
SSC-A
Comp-APC-R700-A :: CD45
F4_80- CD11b-
88.1
Macrophage cells
3.58
Comp-APC-Cy7-A :: CD11b
Comp-PerCP-Cy5-5-A :: F4_80
DC cells
3.09
SSC-A
Comp-FITC-A :: CD11C
M1
57.3
Q2
18.9
Q4
21.2
M2
2.61
Comp-V450-A :: CD86
Comp-PE-Cy7-A :: CD206
Blood_19_019.fcs
Ungated
296776

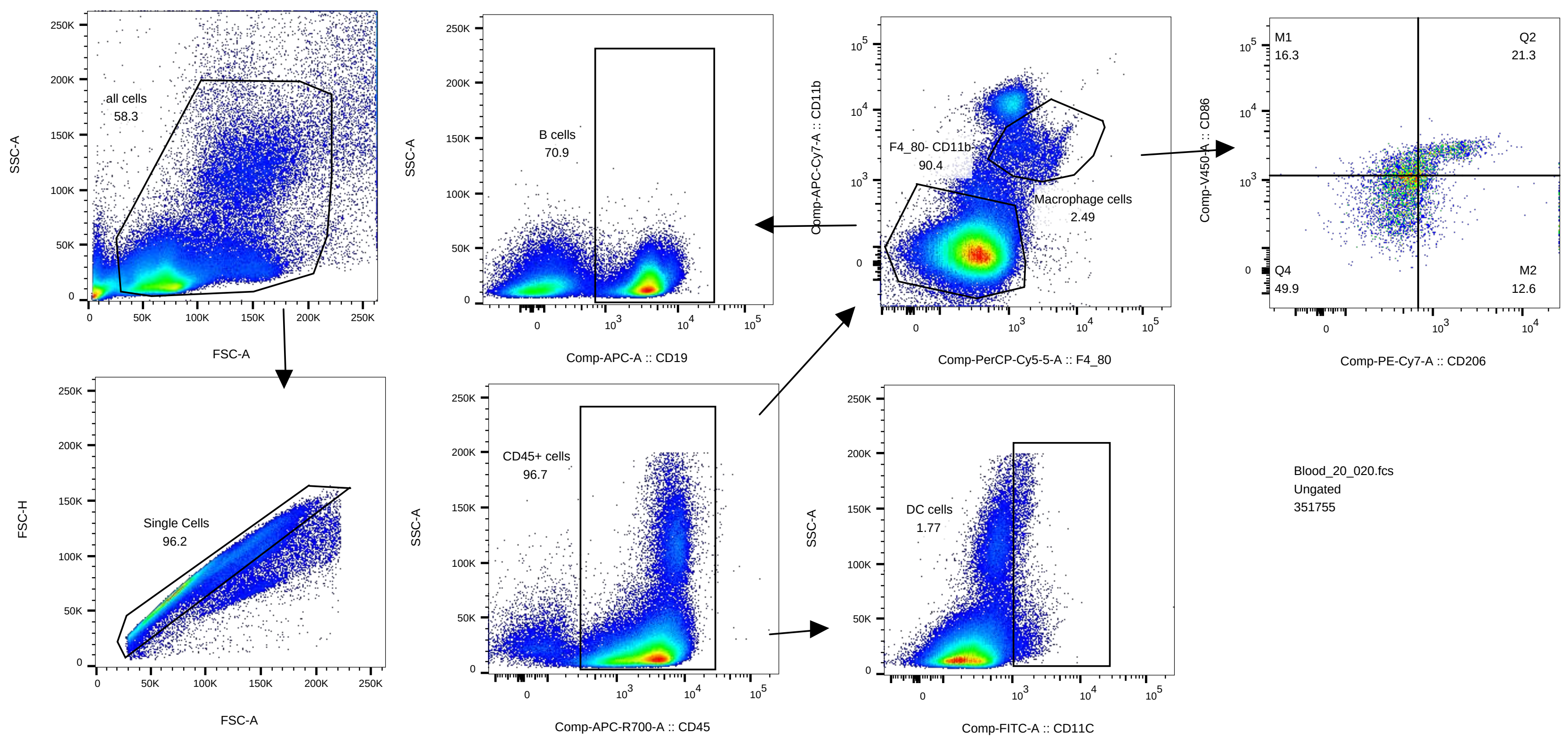
all cells
58.3
SSC-A
FSC-A
Single Cells
96.2
FSC-H
B cells
70.9
Comp-APC-A :: CD19
CD45+ cells
96.7
Comp-APC-R700-A :: CD45
F4_80- CD11b
90.4
Macrophage cells
2.49
Comp-APC-Cy7-A :: CD11b
Comp-PerCP-Cy5-5-A :: F4_80
DC cells
1.77
Comp-FITC-A :: CD11C
M1
16.3
Q2
21.3
Q4
49.9
M2
12.6
Comp-V450-A :: CD86
Comp-PE-Cy7-A :: CD206
Blood_20_020.fcs
Ungated
351755

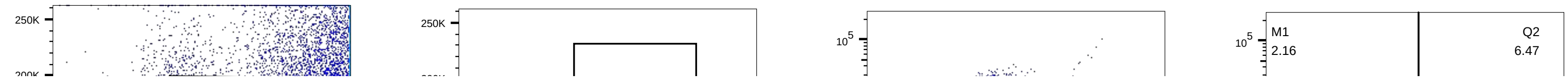
M1
2.16
Q2
6.47

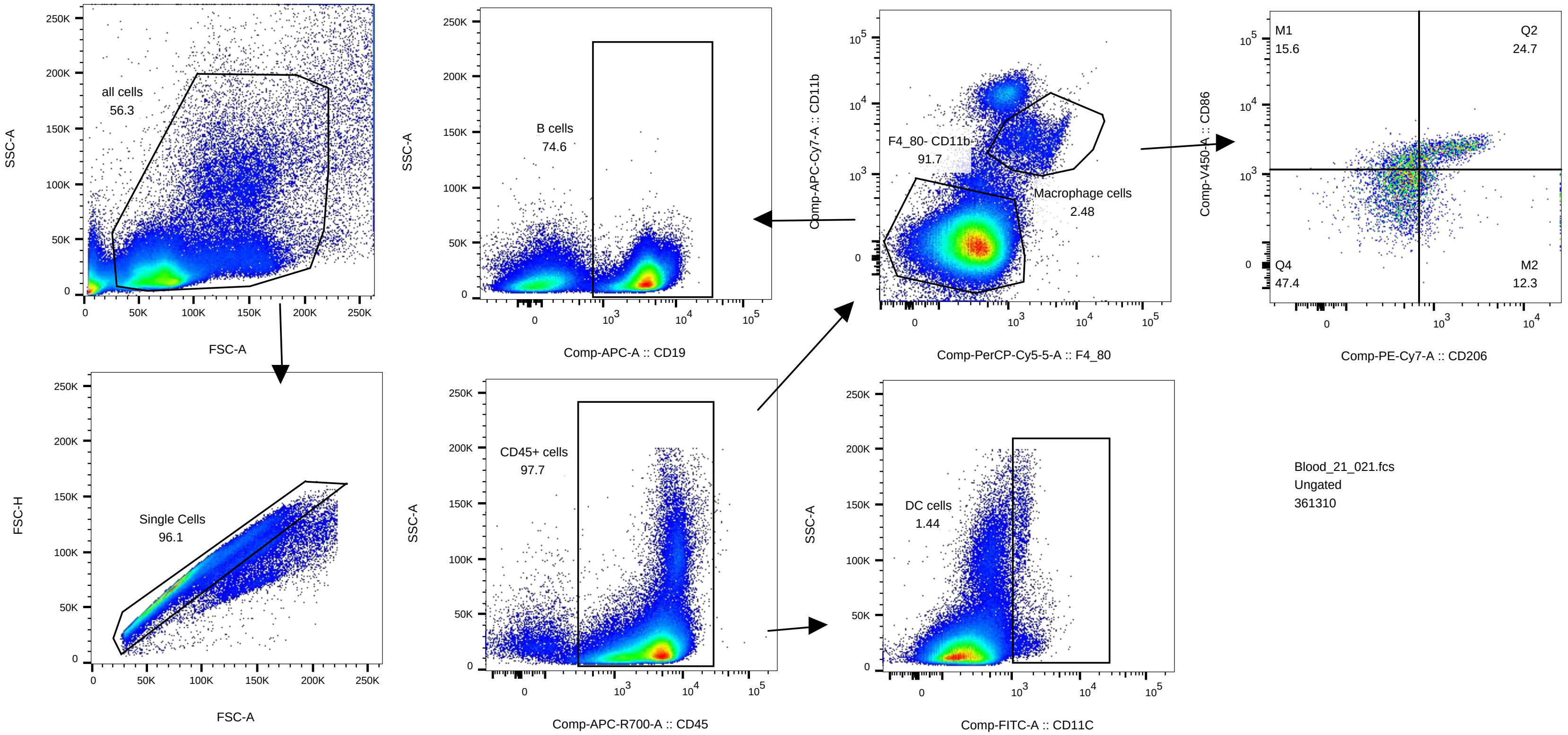
all cells
56.3
SSC-A
FSC-A
Single Cells
96.1
FSC-H
FSC-A
B cells
74.6
SSC-A
Comp-APC-A :: CD19
CD45+ cells
97.7
SSC-A
Comp-APC-R700-A :: CD45
F4_80- CD11b-
91.7
Macrophage cells
2.48
Comp-APC-Cy7-A :: CD11b
Comp-PerCP-Cy5-5-A :: F4_80
DC cells
1.44
SSC-A
Comp-FITC-A :: CD11C
M1
15.6
Q2
24.7
Q4
47.4
M2
12.3
Comp-V450-A :: CD86
Comp-PE-Cy7-A :: CD206
Blood_21_021.fcs
Ungated
361310

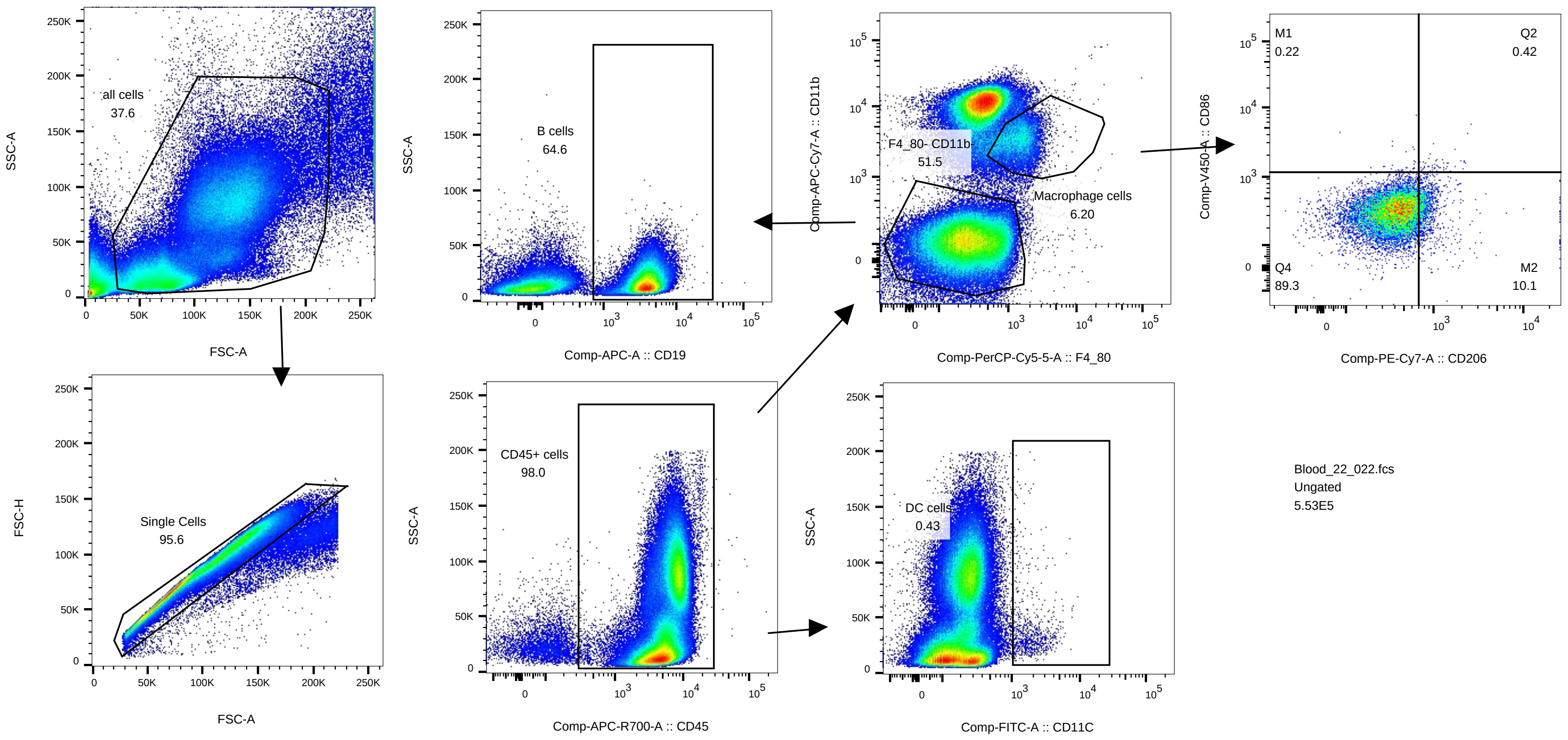
all cells
37.6
SSC-A
FSC-A
Single Cells
95.6
FSC-H
FSC-A
B cells
64.6
SSC-A
Comp-APC-A :: CD19
CD45+ cells
98.0
SSC-A
Comp-APC-R700-A :: CD45
F4_80- CD11b
51.5
Macrophage cells
6.20
Comp-APC-Cy7-A :: CD11b
Comp-PerCP-Cy5-5-A :: F4_80
DC cells
0.43
SSC-A
Comp-FITC-A :: CD11C
M1
0.22
Q2
0.42
Q4
89.3
M2
10.1
Comp-V450-A :: CD86
Comp-PE-Cy7-A :: CD206
Blood_22_022.fcs
Ungated
5.53E5

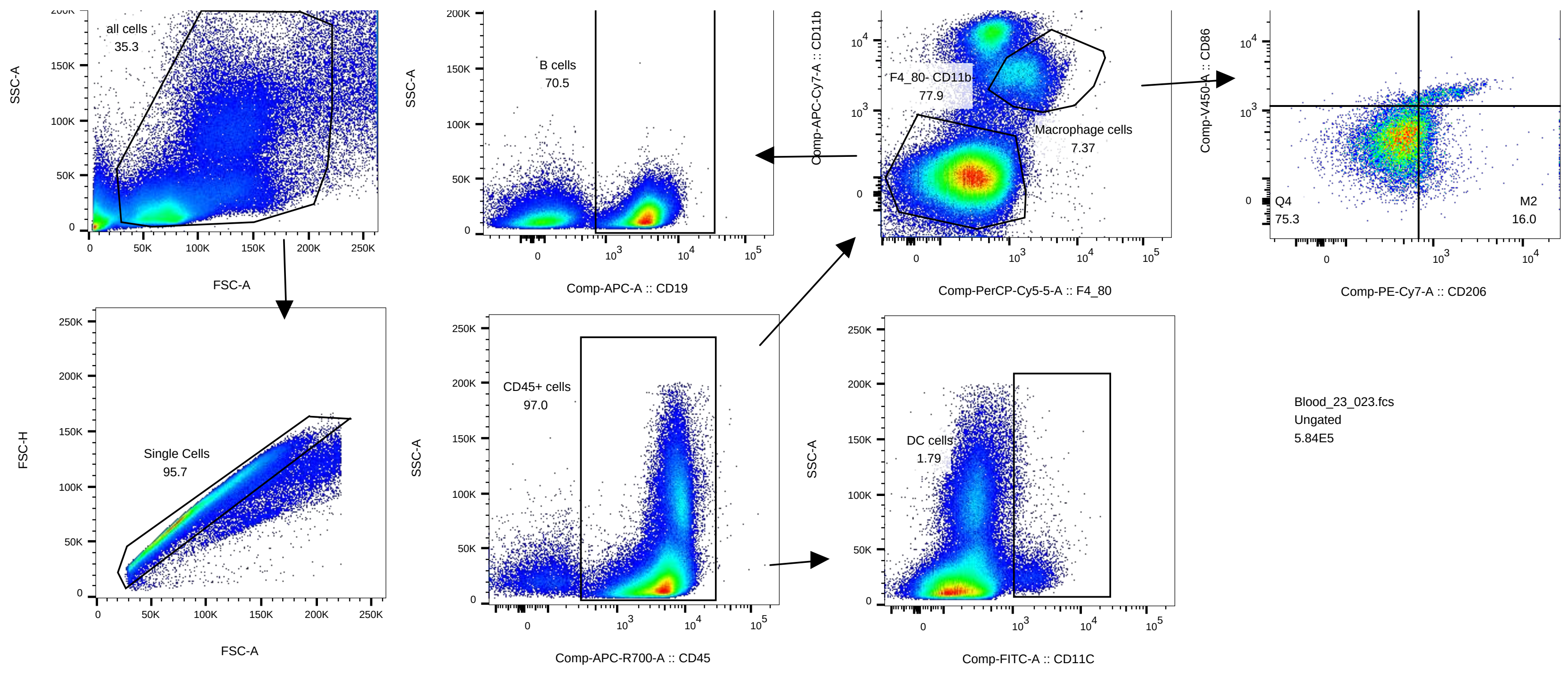

all cells
35.3
SSC-A
FSC-A
Single Cells
95.7
FSC-H
FSC-A
B cells
70.5
SSC-A
Comp-APC-A :: CD19
CD45+ cells
97.0
SSC-A
Comp-APC-R700-A :: CD45
F4_80- CD11b
77.9
Macrophage cells
7.37
Comp-APC-Cy7-A :: CD11b
Comp-PerCP-Cy5-5-A :: F4_80
DC cells
1.79
SSC-A
Comp-FITC-A :: CD11C
Q4
75.3
M2
16.0
Comp-V450-A :: CD86
Comp-PE-Cy7-A :: CD206
Blood_23_023.fcs
Ungated
5.84E5

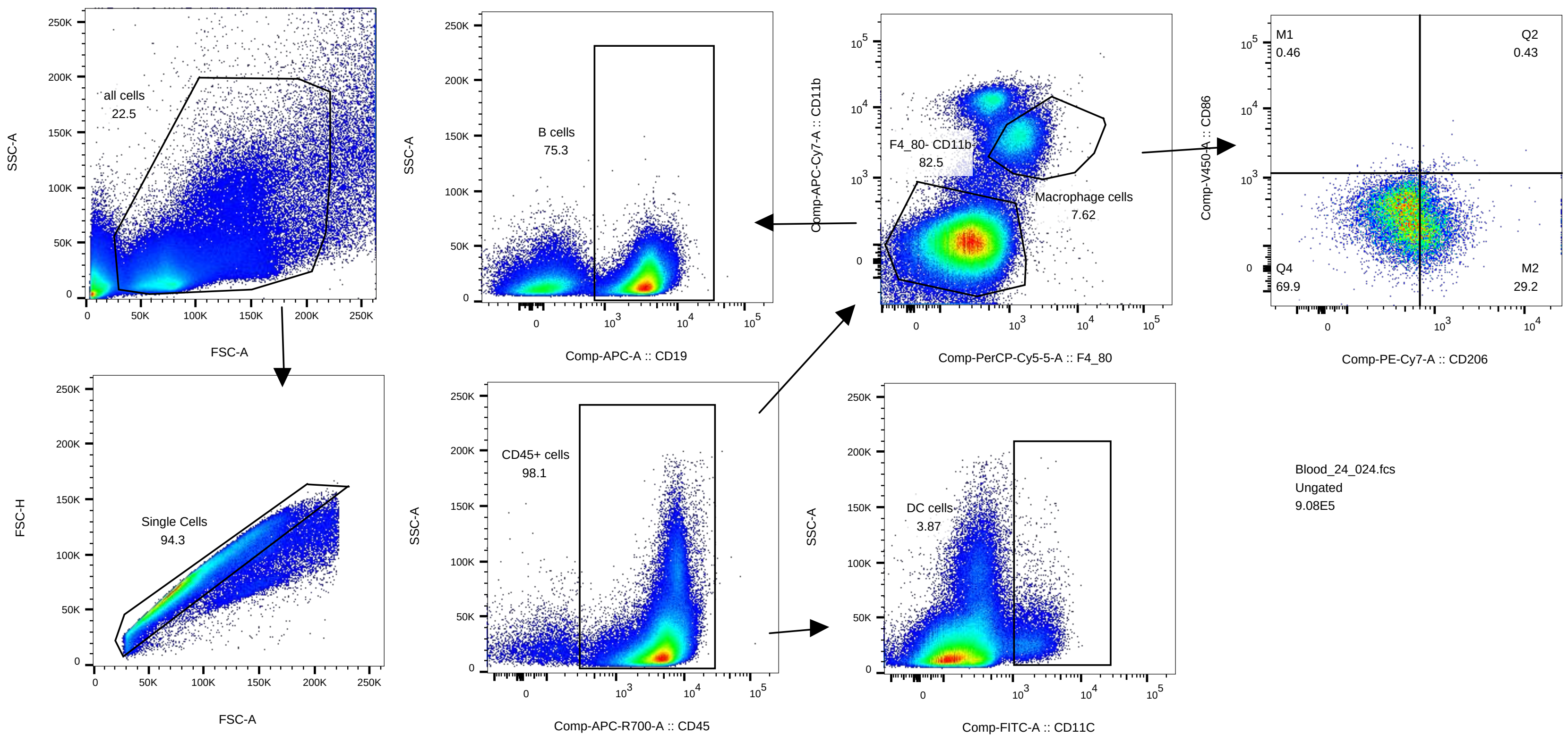

all cells
22.5
SSC-A
FSC-A
Single Cells
94.3
FSC-H
FSC-A
B cells
75.3
SSC-A
Comp-APC-A :: CD19
CD45+ cells
98.1
SSC-A
Comp-APC-R700-A :: CD45
F4_80- CD11b
82.5
Macrophage cells
7.62
Comp-APC-Cy7-A :: CD11b
Comp-PerCP-Cy5-5-A :: F4_80
DC cells
3.87
SSC-A
Comp-FITC-A :: CD11C
M1
0.46
Q2
0.43
Q4
69.9
M2
29.2
Comp-V450-A :: CD86
Comp-PE-Cy7-A :: CD206
Blood_24_024.fcs
Ungated
9.08E5

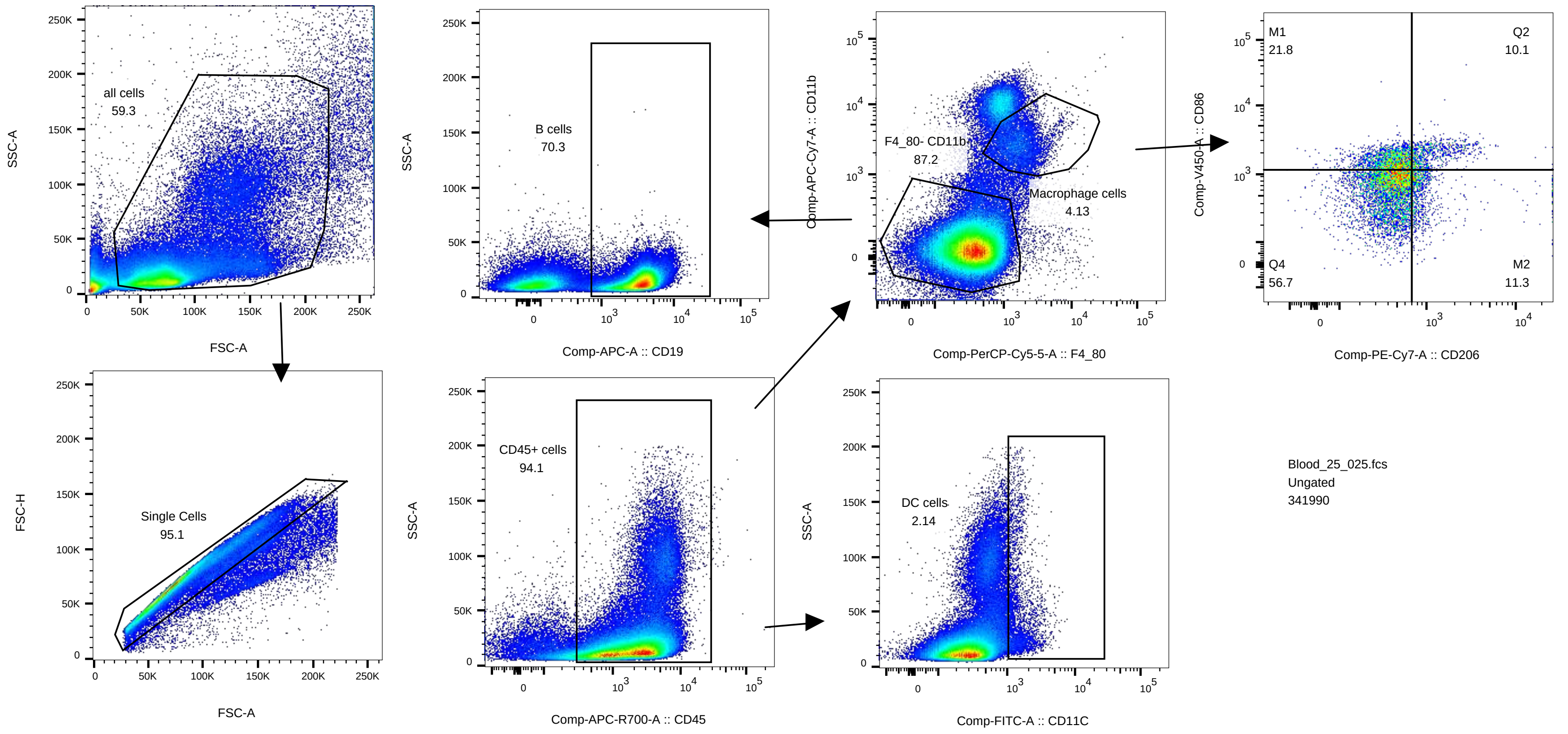

all cells
59.3
SSC-A
FSC-A
Single Cells
95.1
FSC-H
FSC-A
B cells
70.3
SSC-A
Comp-APC-A :: CD19
CD45+ cells
94.1
SSC-A
Comp-APC-R700-A :: CD45
F4_80- CD11b+
87.2
Macrophage cells
4.13
Comp-APC-Cy7-A :: CD11b
Comp-PerCP-Cy5-5-A :: F4_80
DC cells
2.14
SSC-A
Comp-FITC-A :: CD11C
M1
21.8
Q2
10.1
Q4
56.7
M2
11.3
Comp-V450-A :: CD86
Comp-PE-Cy7-A :: CD206
Blood_25_025.fcs
Ungated
341990
