# Supplementary material for: A novel HVEM-Fc recombinant protein for lung cancer immunotherapy
Source: J Exp Clin Cancer Res. 2025 Feb 20;44:62. doi: 10.1186/s13046-025-03324-8 (PMC11841141; doi:10.1186/s13046-025-03324-8)
Supplement: Supplementary file 1 — Flow cytometry gating strategies for the identification of immune cell subsets. [file 13046_2025_3324_MOESM1_ESM.zip › Supplement File2.pdf]

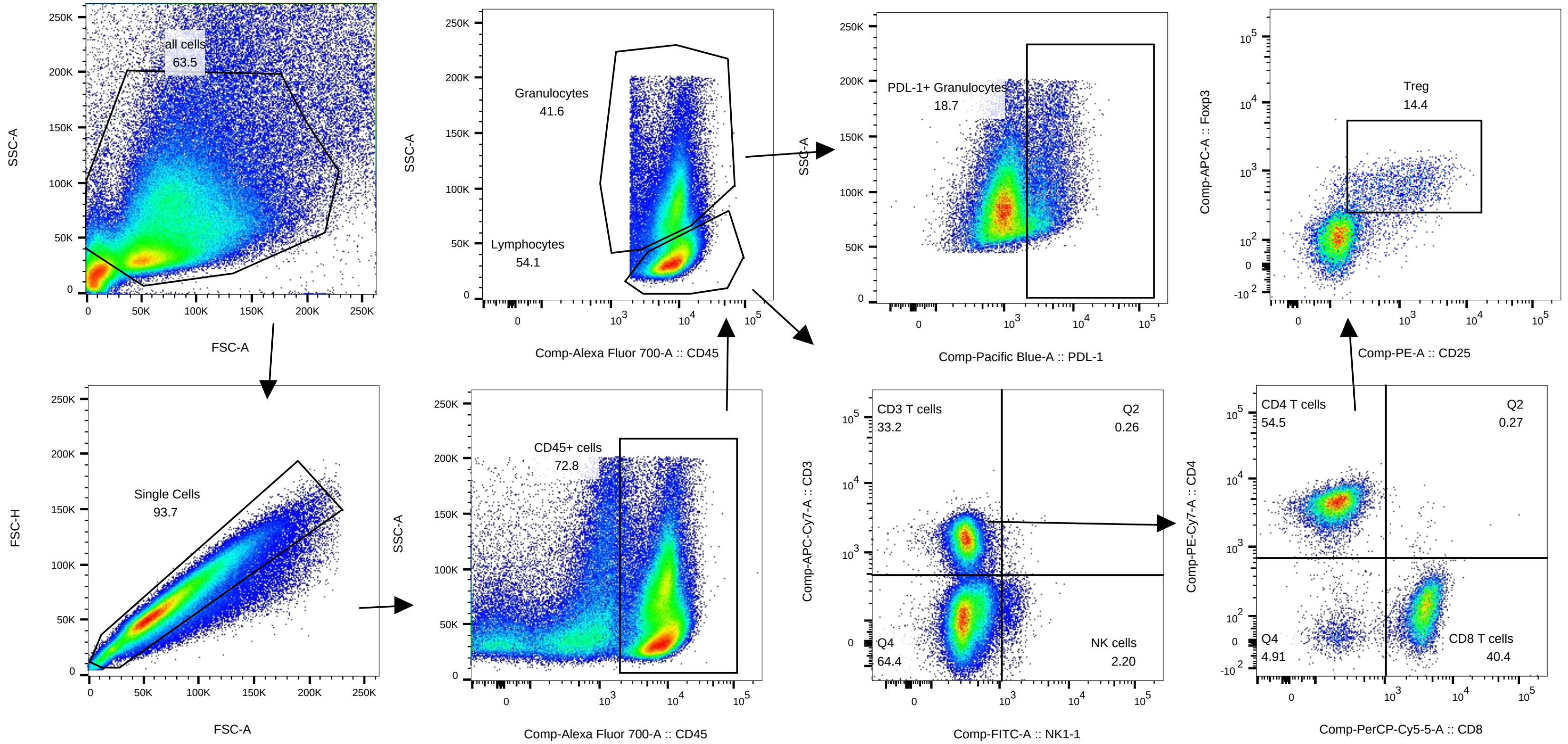

Lung_1_001.fcs
Ungated
256404

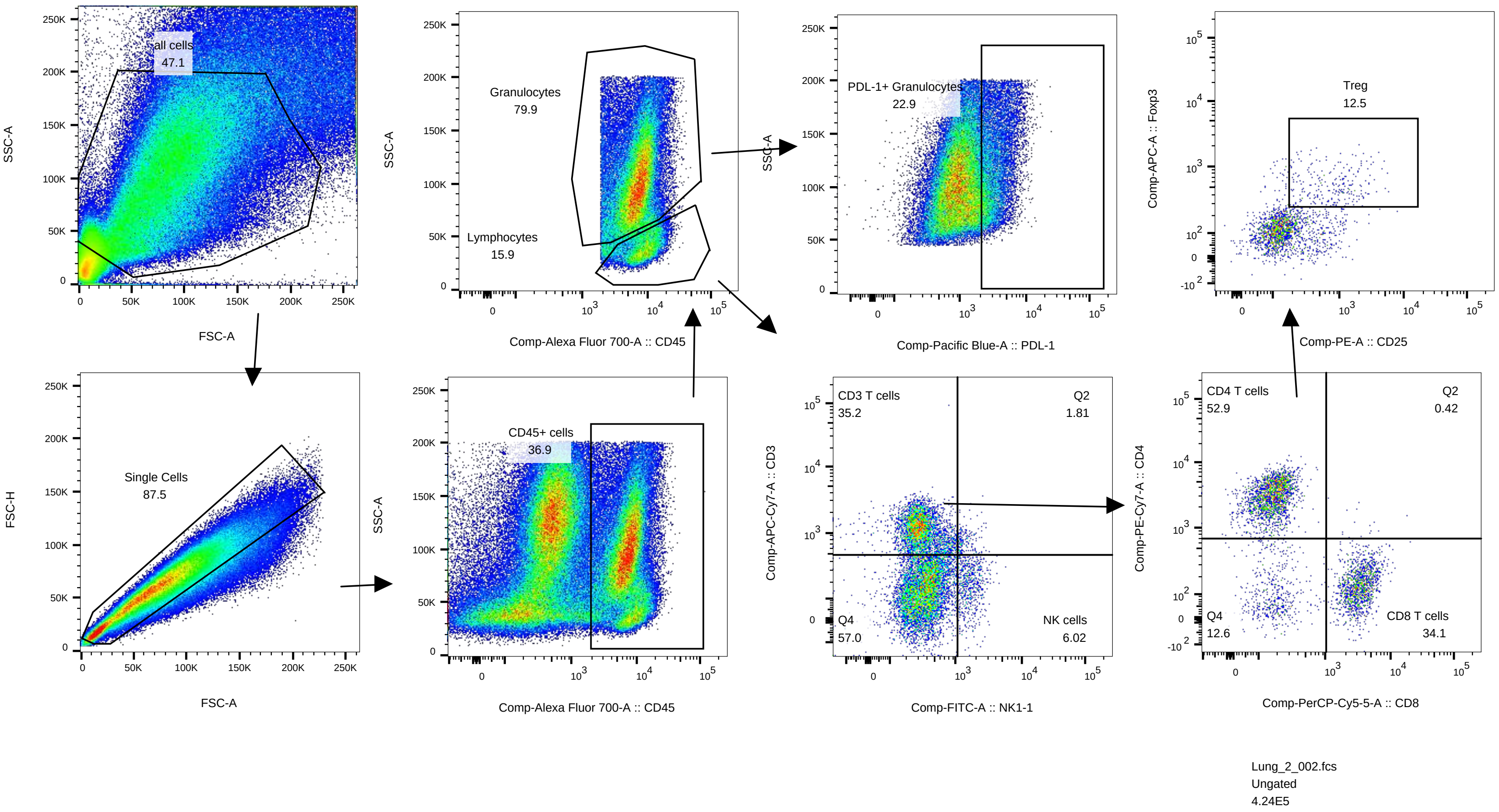
all cells
47.1
SSC-A
FSC-A
Single Cells
87.5
FSC-H
FSC-A
Granulocytes
79.9
Lymphocytes
15.9
SSC-A
Comp-Alexa Fluor 700-A :: CD45
CD45+ cells
36.9
SSC-A
Comp-Alexa Fluor 700-A :: CD45
PDL-1+ Granulocytes
22.9
SSC-A
Comp-Pacific Blue-A :: PDL-1
CD3 T cells
35.2
Q2
1.81
Q4
57.0
NK cells
6.02
Comp-APC-Cy7-A :: CD3
Comp-FITC-A :: NK1-1
Treg
12.5
Comp-APC-A :: Foxp3
Comp-PE-A :: CD25
CD4 T cells
52.9
Q2
0.42
Q4
12.6
CD8 T cells
34.1
Comp-PE-Cy7-A :: CD4
Comp-PerCP-Cy5-5-A :: CD8
Lung_2_002.fcs
Ungated
4.24E5

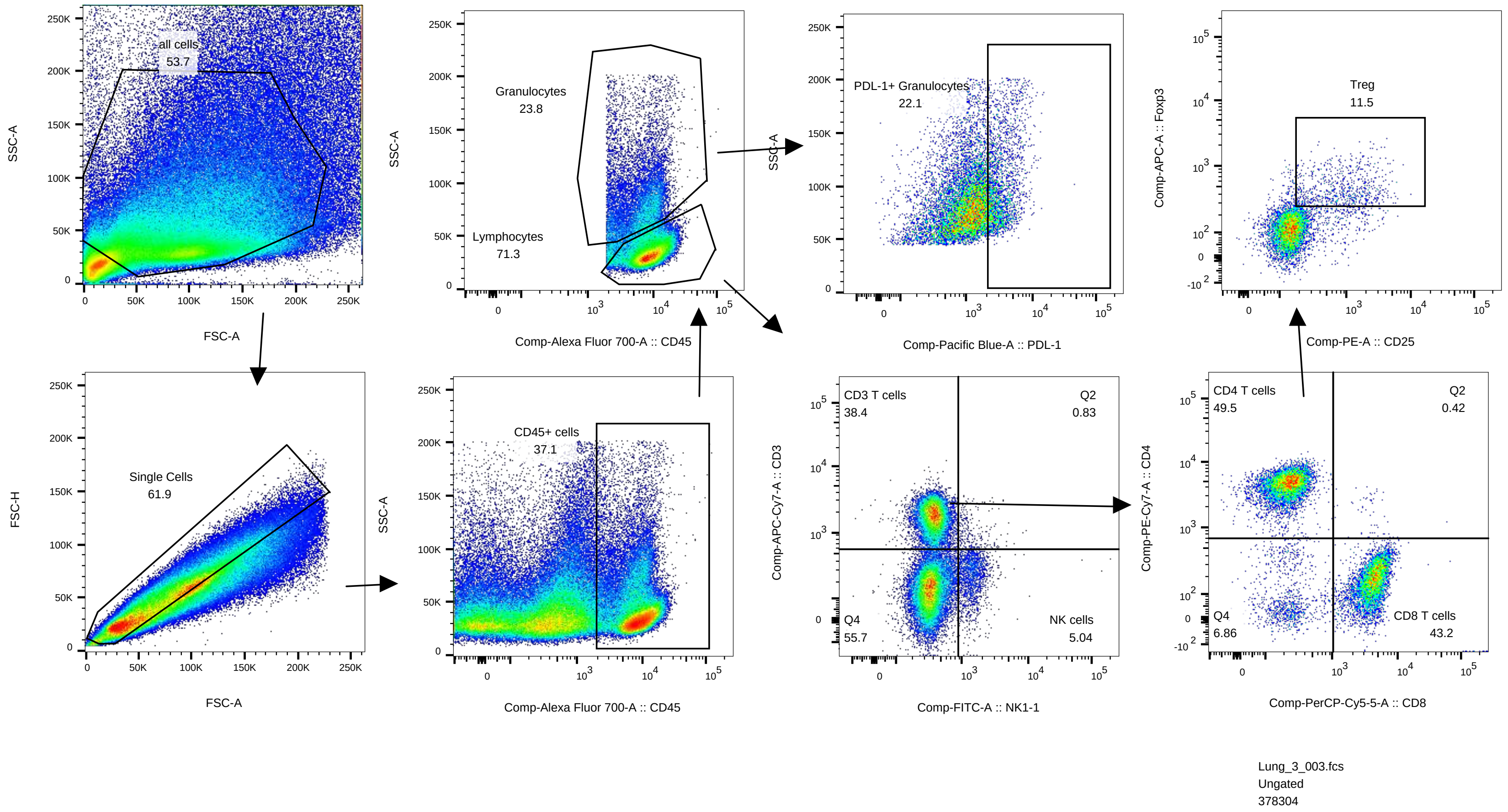
all cells
53.7
SSC-A
FSC-A
Single Cells
61.9
FSC-H
Granulocytes
23.8
Lymphocytes
71.3
Comp-Alexa Fluor 700-A :: CD45
CD45+ cells
37.1
PDL-1+ Granulocytes
22.1
Comp-Pacific Blue-A :: PDL-1
CD3 T cells
38.4
Q2
0.83
Q4
55.7
NK cells
5.04
Comp-APC-Cy7-A :: CD3
Comp-FITC-A :: NK1-1
Treg
11.5
Comp-APC-A :: Foxp3
Comp-PE-A :: CD25
CD4 T cells
49.5
Q2
0.42
Q4
6.86
CD8 T cells
43.2
Comp-PE-Cy7-A :: CD4
Comp-PerCP-Cy5-5-A :: CD8
Lung_3_003.fcs
Ungated
378304

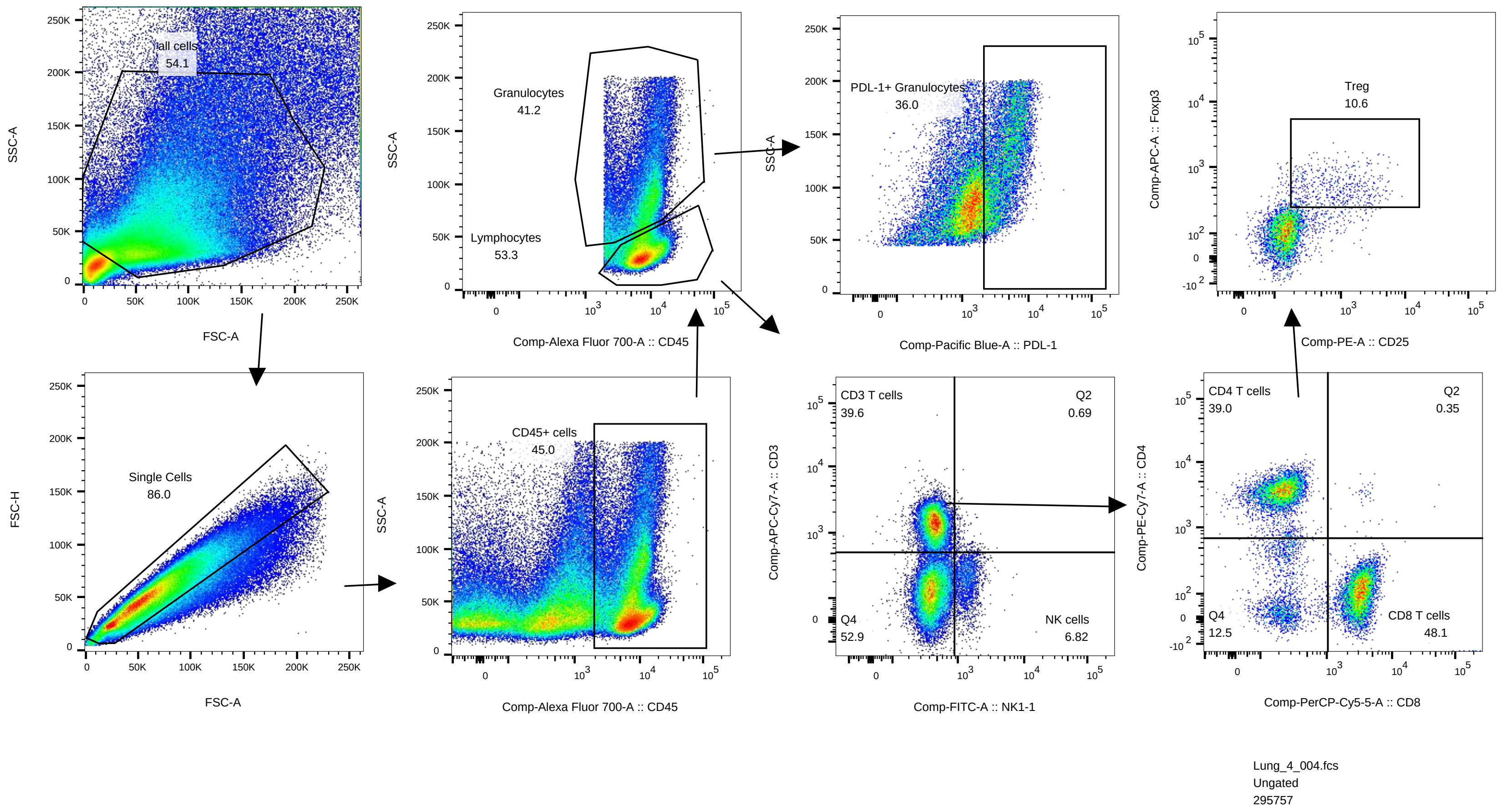

all cells
54.1
SSC-A
FSC-A
Single Cells
86.0
FSC-H
FSC-A
Granulocytes
41.2
Lymphocytes
53.3
SSC-A
Comp-Alexa Fluor 700-A :: CD45
CD45+ cells
45.0
SSC-A
Comp-Alexa Fluor 700-A :: CD45
PDL-1+ Granulocytes
36.0
SSC-A
Comp-Pacific Blue-A :: PDL-1
CD3 T cells
39.6
Q2
0.69
Q4
52.9
NK cells
6.82
Comp-APC-Cy7-A :: CD3
Comp-FITC-A :: NK1-1
Treg
10.6
Comp-APC-A :: Foxp3
Comp-PE-A :: CD25
CD4 T cells
39.0
Q2
0.35
Q4
12.5
CD8 T cells
48.1
Comp-PE-Cy7-A :: CD4
Comp-PerCP-Cy5-5-A :: CD8
Lung_4_004.fcs
Ungated
295757

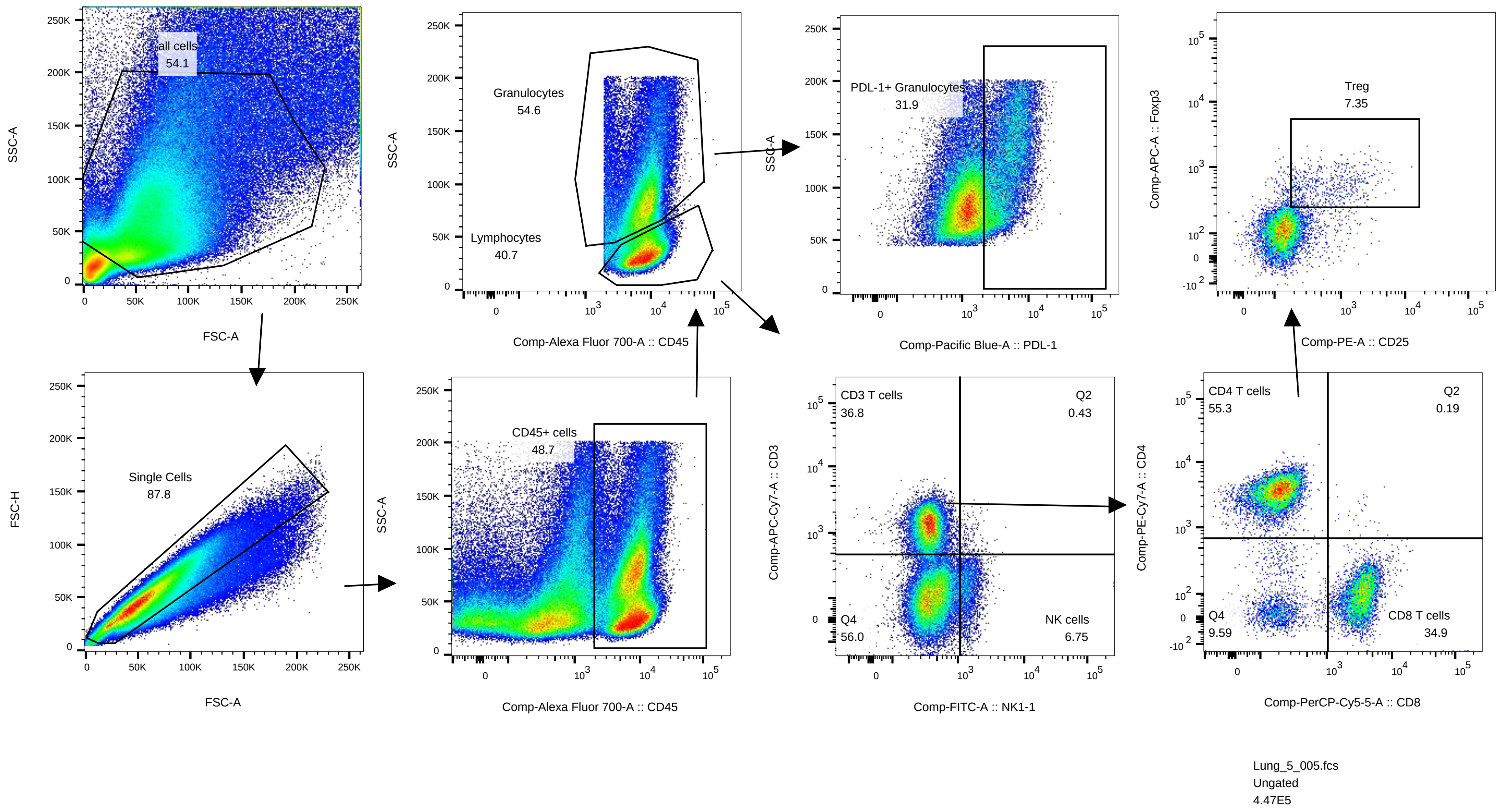

all cells
54.1
SSC-A
FSC-A
Single Cells
87.8
FSC-H
Granulocytes
54.6
Lymphocytes
40.7
Comp-Alexa Fluor 700-A :: CD45
CD45+ cells
48.7
PDL-1+ Granulocytes
31.9
Comp-Pacific Blue-A :: PDL-1
CD3 T cells
36.8
Q2
0.43
Q4
56.0
NK cells
6.75
Comp-APC-Cy7-A :: CD3
Comp-FITC-A :: NK1-1
Treg
7.35
Comp-APC-A :: Foxp3
Comp-PE-A :: CD25
CD4 T cells
55.3
Q2
0.19
Q4
9.59
CD8 T cells
34.9
Comp-PE-Cy7-A :: CD4
Comp-PerCP-Cy5-5-A :: CD8
Lung_5_005.fcs
Ungated
4.47E5

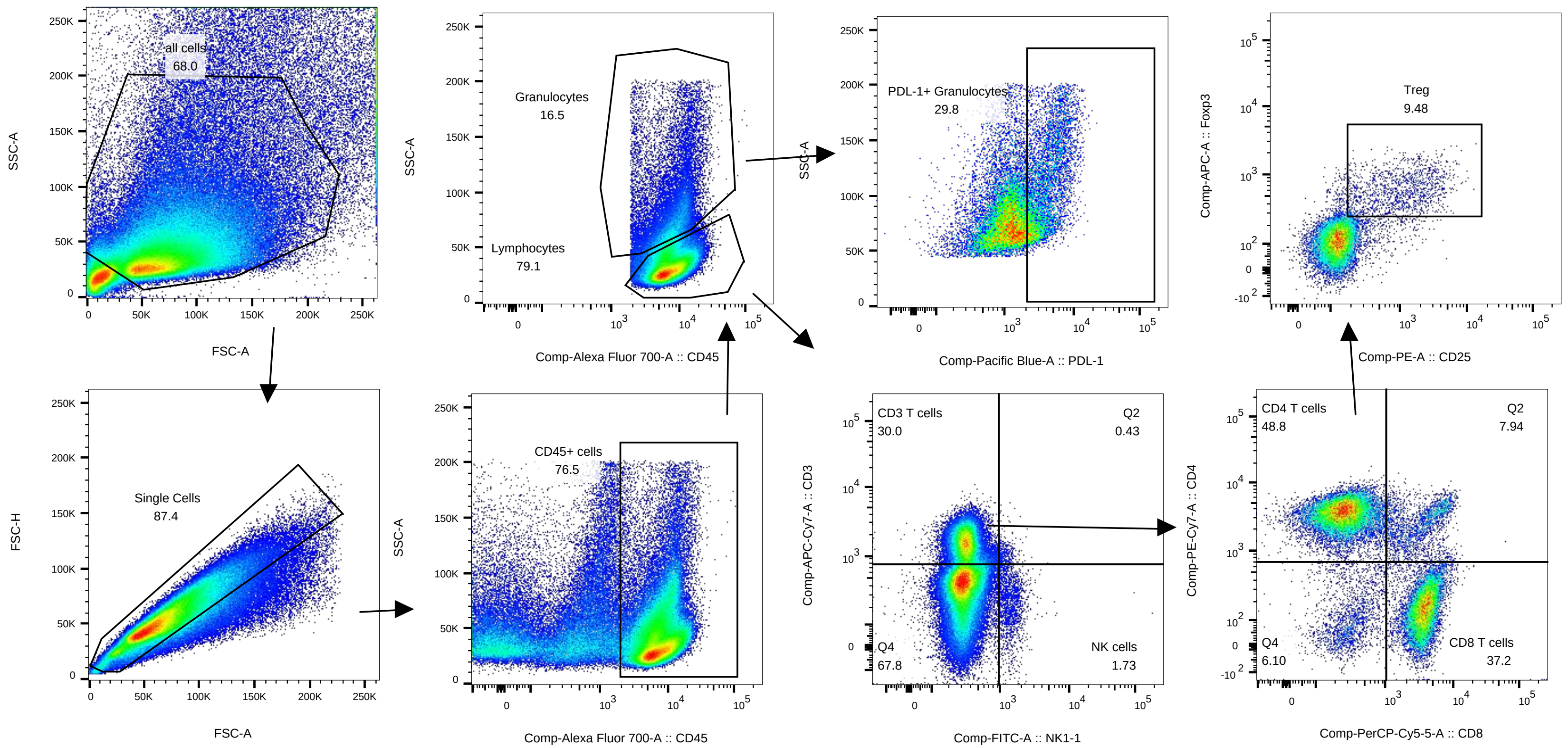

Lung_6_006.fcs
Ungated
277633

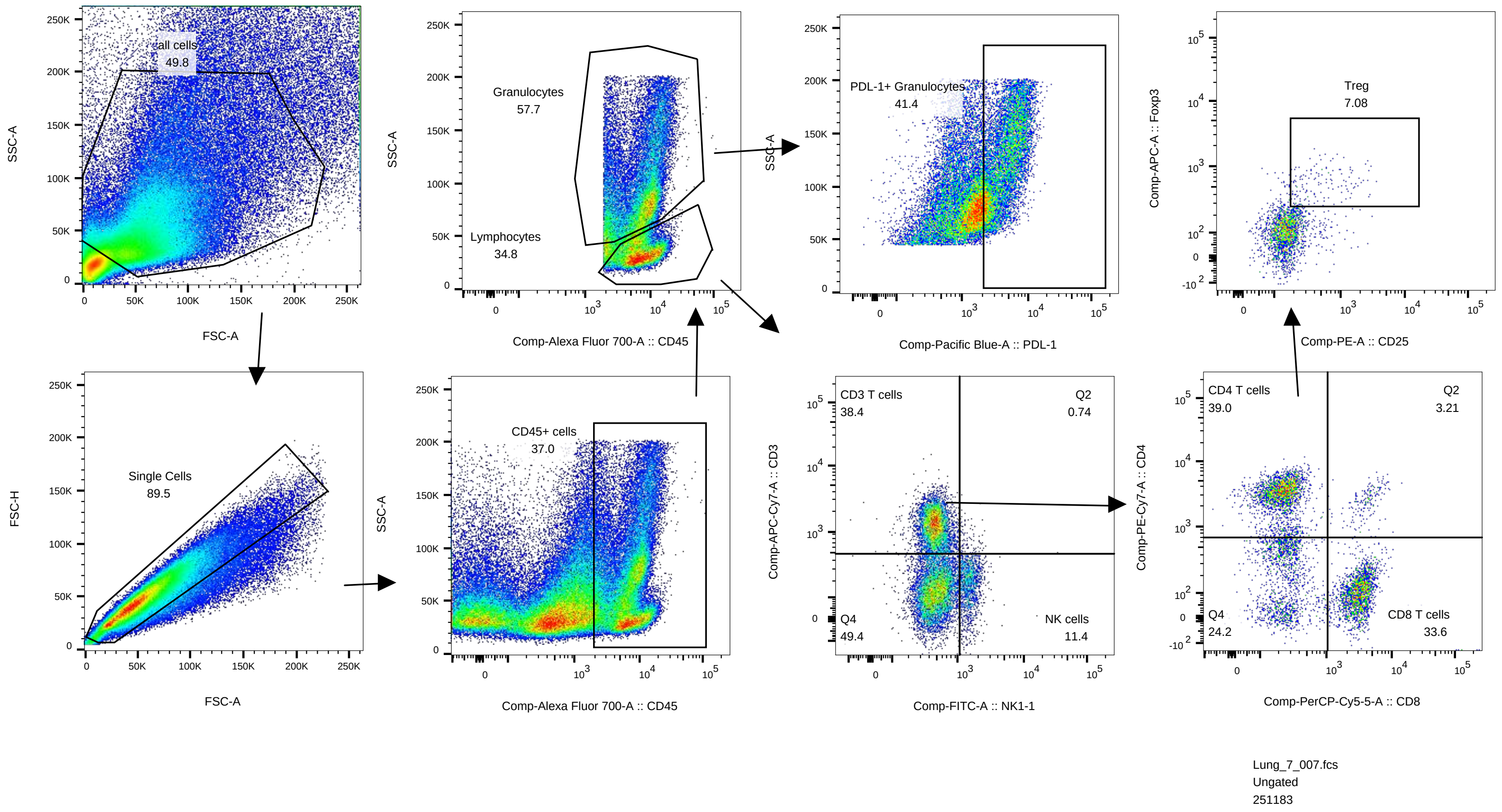
all cells
49.8
SSC-A
FSC-A
Single Cells
89.5
FSC-H
FSC-A
Granulocytes
57.7
Lymphocytes
34.8
SSC-A
Comp-Alexa Fluor 700-A :: CD45
CD45+ cells
37.0
SSC-A
Comp-Alexa Fluor 700-A :: CD45
PDL-1+ Granulocytes
41.4
SSC-A
Comp-Pacific Blue-A :: PDL-1
CD3 T cells
38.4
Q2
0.74
Q4
49.4
NK cells
11.4
Comp-APC-Cy7-A :: CD3
Comp-FITC-A :: NK1-1
Treg
7.08
Comp-APC-A :: Foxp3
Comp-PE-A :: CD25
CD4 T cells
39.0
Q2
3.21
Q4
24.2
CD8 T cells
33.6
Comp-PE-Cy7-A :: CD4
Comp-PerCP-Cy5-5-A :: CD8
Lung_7_007.fcs
Ungated
251183

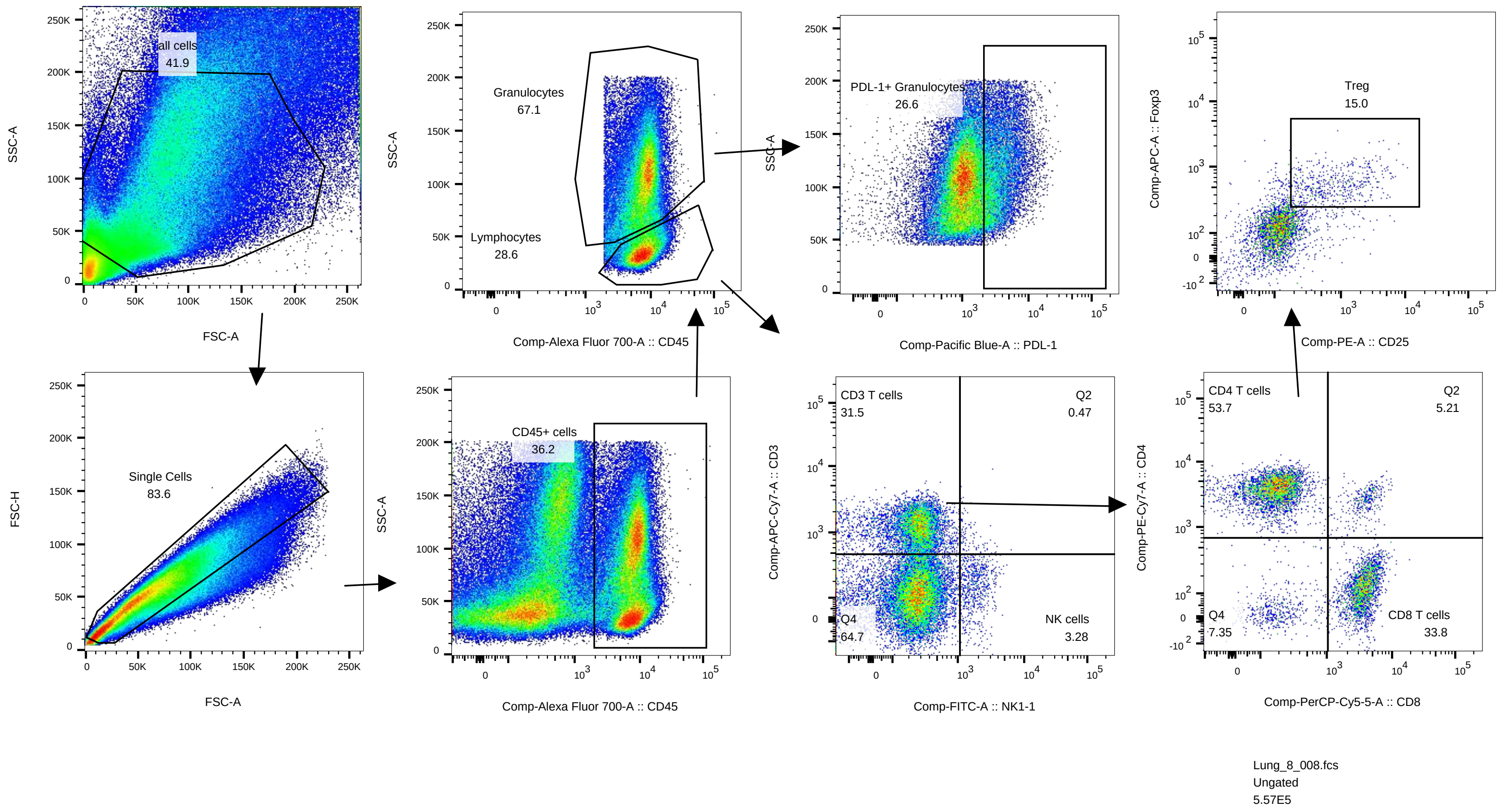

all cells
41.9
SSC-A
FSC-A
Single Cells
83.6
FSC-H
Granulocytes
67.1
Lymphocytes
28.6
Comp-Alexa Fluor 700-A :: CD45
CD45+ cells
36.2
PDL-1+ Granulocytes
26.6
Comp-Pacific Blue-A :: PDL-1
CD3 T cells
31.5
Q2
0.47
Q4
64.7
NK cells
3.28
Comp-APC-Cy7-A :: CD3
Comp-FITC-A :: NK1-1
Treg
15.0
Comp-APC-A :: Foxp3
Comp-PE-A :: CD25
CD4 T cells
53.7
Q2
5.21
Q4
7.35
CD8 T cells
33.8
Comp-PE-Cy7-A :: CD4
Comp-PerCP-Cy5-5-A :: CD8
Lung_8_008.fcs
Ungated
5.57E5

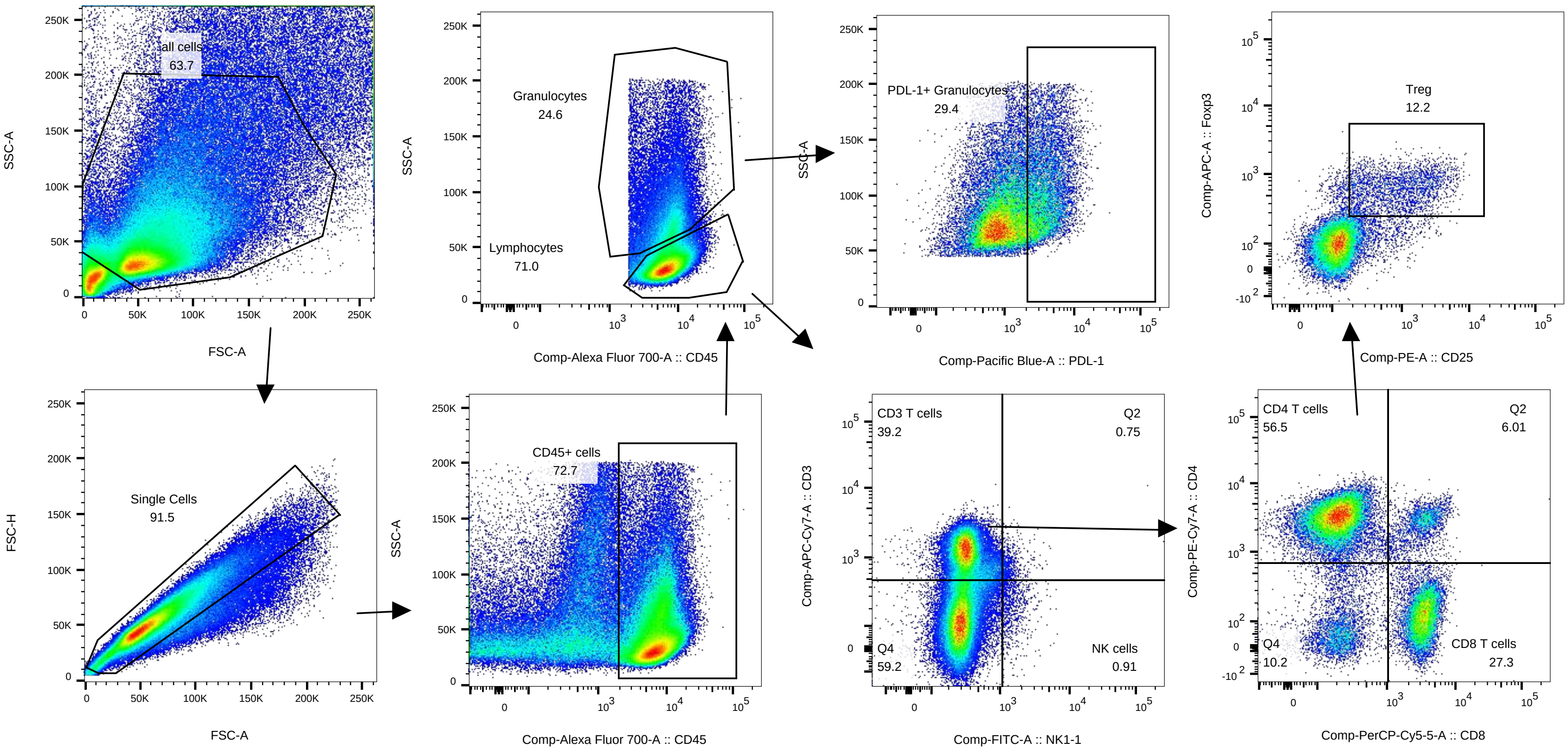

Lung_9_009.fcs
Ungated
315764

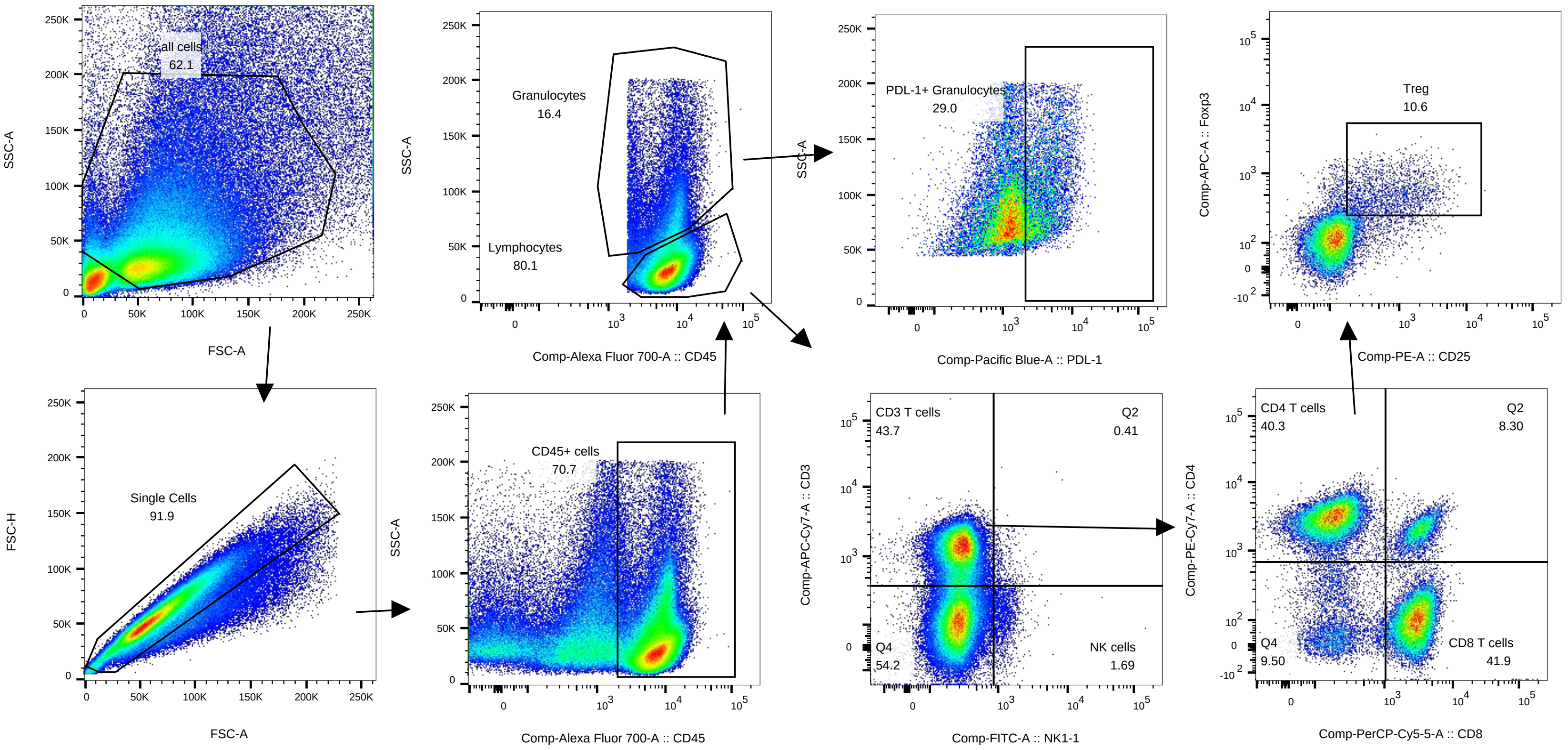

Lung_10_010.fcs
Ungated
318090

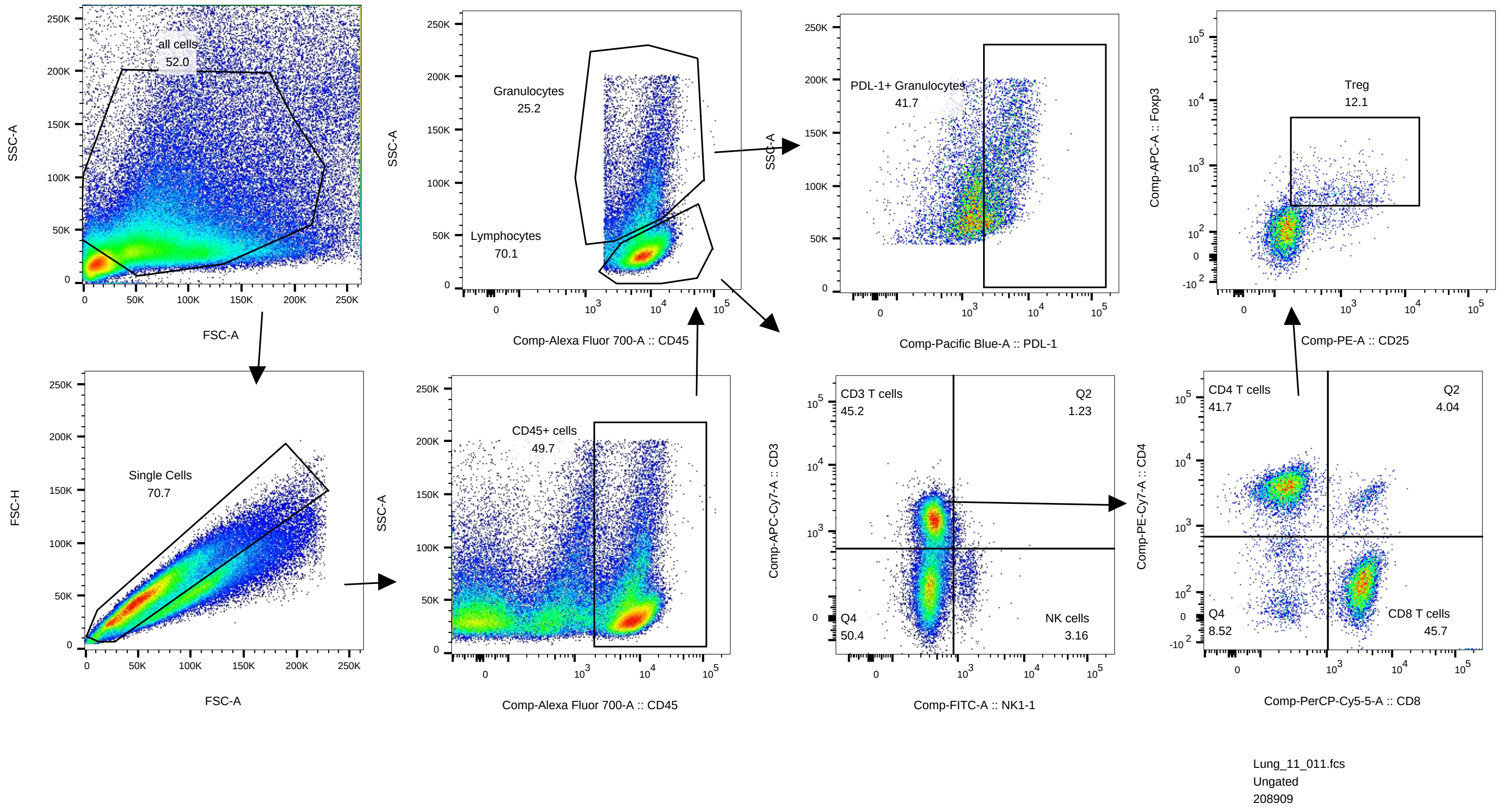
all cells
52.0
SSC-A
FSC-A
Single Cells
70.7
FSC-H
FSC-A
Granulocytes
25.2
Lymphocytes
70.1
SSC-A
Comp-Alexa Fluor 700-A :: CD45
CD45+ cells
49.7
SSC-A
Comp-Alexa Fluor 700-A :: CD45
PDL-1+ Granulocytes
41.7
SSC-A
Comp-Pacific Blue-A :: PDL-1
CD3 T cells
45.2
Q2
1.23
Q4
50.4
NK cells
3.16
Comp-APC-Cy7-A :: CD3
Comp-FITC-A :: NK1-1
Treg
12.1
Comp-APC-A :: Foxp3
Comp-PE-A :: CD25
CD4 T cells
41.7
Q2
4.04
Q4
8.52
CD8 T cells
45.7
Comp-PE-Cy7-A :: CD4
Comp-PerCP-Cy5-5-A :: CD8
Lung_11_011.fcs
Ungated
208909

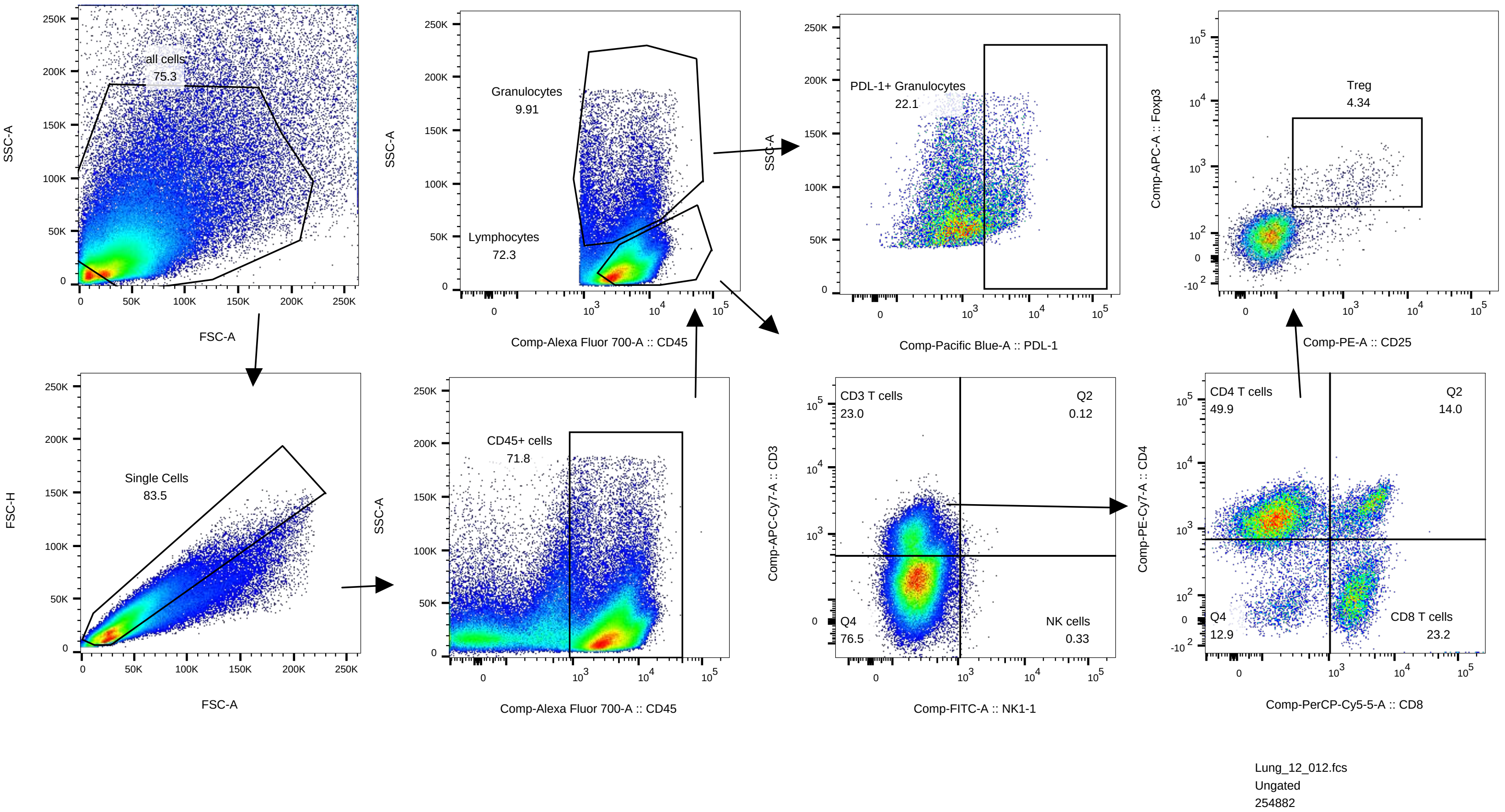

all cells
75.3
SSC-A
FSC-A
Single Cells
83.5
FSC-H
FSC-A
Granulocytes
9.91
Lymphocytes
72.3
SSC-A
Comp-Alexa Fluor 700-A :: CD45
CD45+ cells
71.8
SSC-A
Comp-Alexa Fluor 700-A :: CD45
PDL-1+ Granulocytes
22.1
SSC-A
Comp-Pacific Blue-A :: PDL-1
CD3 T cells
23.0
Q2
0.12
Q4
76.5
NK cells
0.33
Comp-APC-Cy7-A :: CD3
Comp-FITC-A :: NK1-1
Treg
4.34
Comp-APC-A :: Foxp3
Comp-PE-A :: CD25
CD4 T cells
49.9
Q2
14.0
Q4
12.9
CD8 T cells
23.2
Comp-PE-Cy7-A :: CD4
Comp-PerCP-Cy5-5-A :: CD8
Lung_12_012.fcs
Ungated
254882

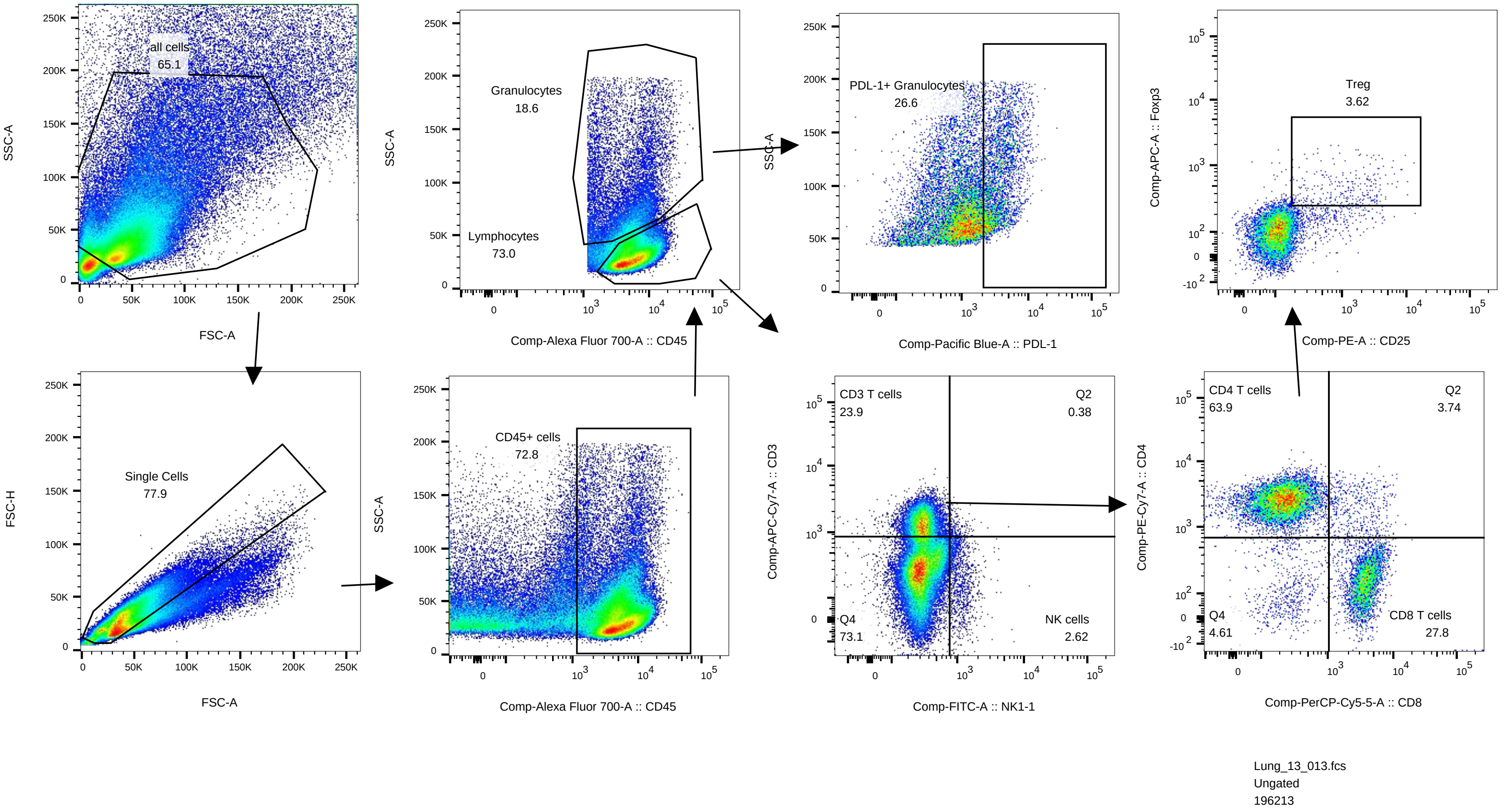
all cells
65.1
SSC-A
FSC-A
Single Cells
77.9
FSC-H
FSC-A
Granulocytes
18.6
Lymphocytes
73.0
SSC-A
Comp-Alexa Fluor 700-A :: CD45
CD45+ cells
72.8
SSC-A
Comp-Alexa Fluor 700-A :: CD45
PDL-1+ Granulocytes
26.6
SSC-A
Comp-Pacific Blue-A :: PDL-1
CD3 T cells
23.9
Q2
0.38
Q4
73.1
NK cells
2.62
Comp-APC-Cy7-A :: CD3
Comp-FITC-A :: NK1-1
Treg
3.62
Comp-APC-A :: Foxp3
Comp-PE-A :: CD25
CD4 T cells
63.9
Q2
3.74
Q4
4.61
CD8 T cells
27.8
Comp-PE-Cy7-A :: CD4
Comp-PerCP-Cy5-5-A :: CD8
Lung_13_013.fcs
Ungated
196213

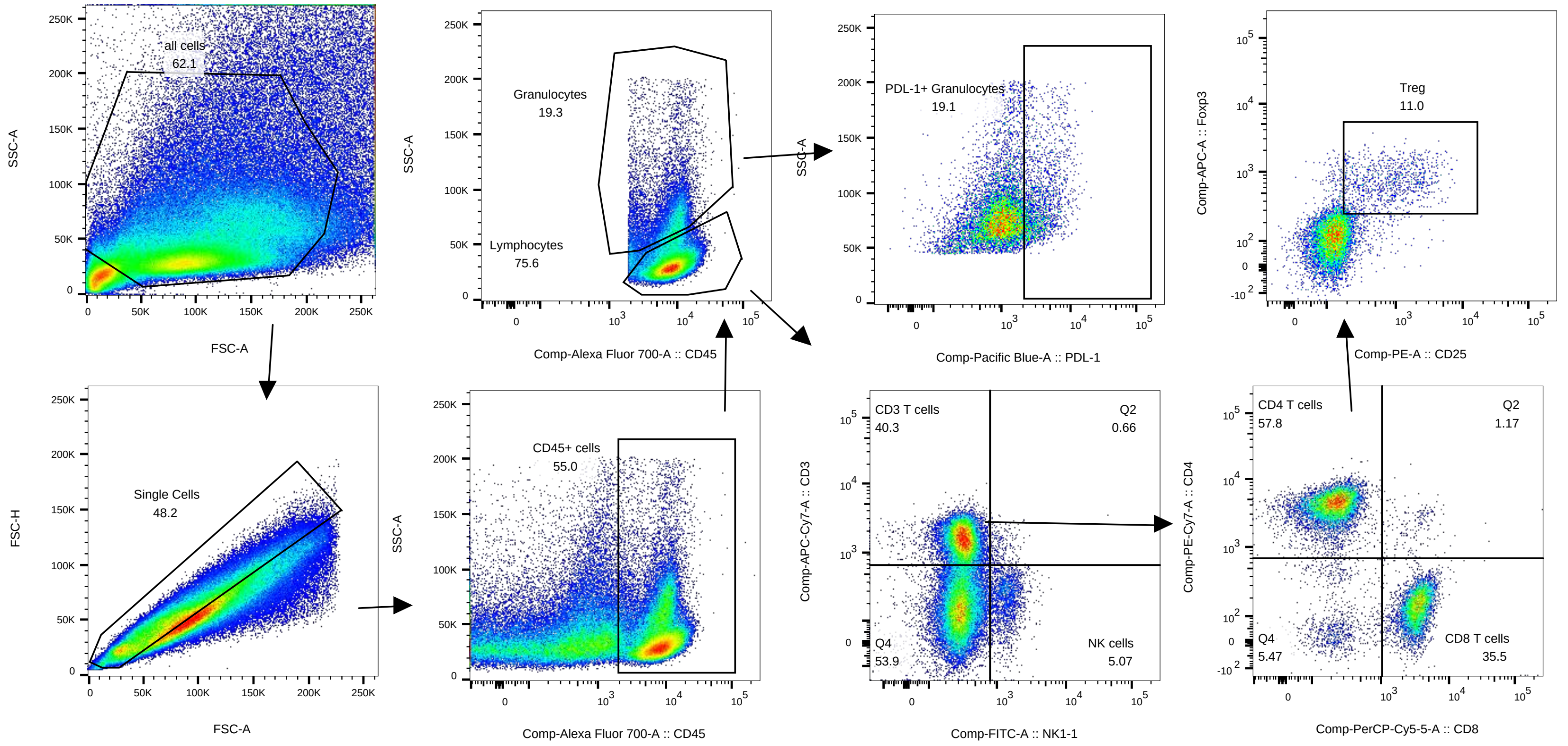

Lung_14_014.fcs
Ungated
309046

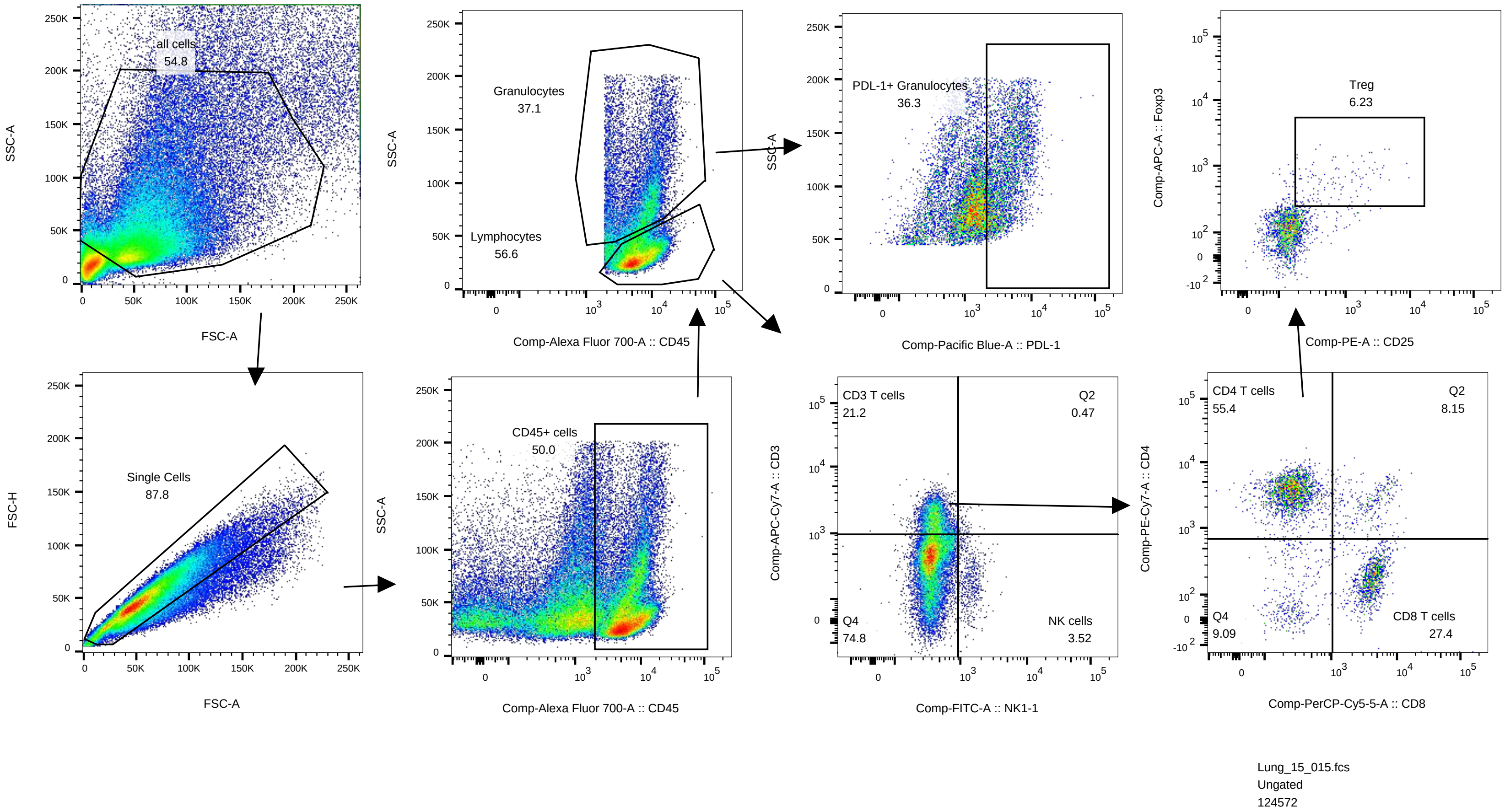
all cells
54.8
SSC-A
FSC-A
Single Cells
87.8
FSC-H
Granulocytes
37.1
Lymphocytes
56.6
Comp-Alexa Fluor 700-A :: CD45
CD45+ cells
50.0
PDL-1+ Granulocytes
36.3
Comp-Pacific Blue-A :: PDL-1
CD3 T cells
21.2
Q2
0.47
Q4
74.8
NK cells
3.52
Comp-APC-Cy7-A :: CD3
Comp-FITC-A :: NK1-1
Treg
6.23
Comp-APC-A :: Foxp3
Comp-PE-A :: CD25
CD4 T cells
55.4
Q2
8.15
Q4
9.09
CD8 T cells
27.4
Comp-PE-Cy7-A :: CD4
Comp-PerCP-Cy5-5-A :: CD8
Lung_15_015.fcs
Ungated
124572

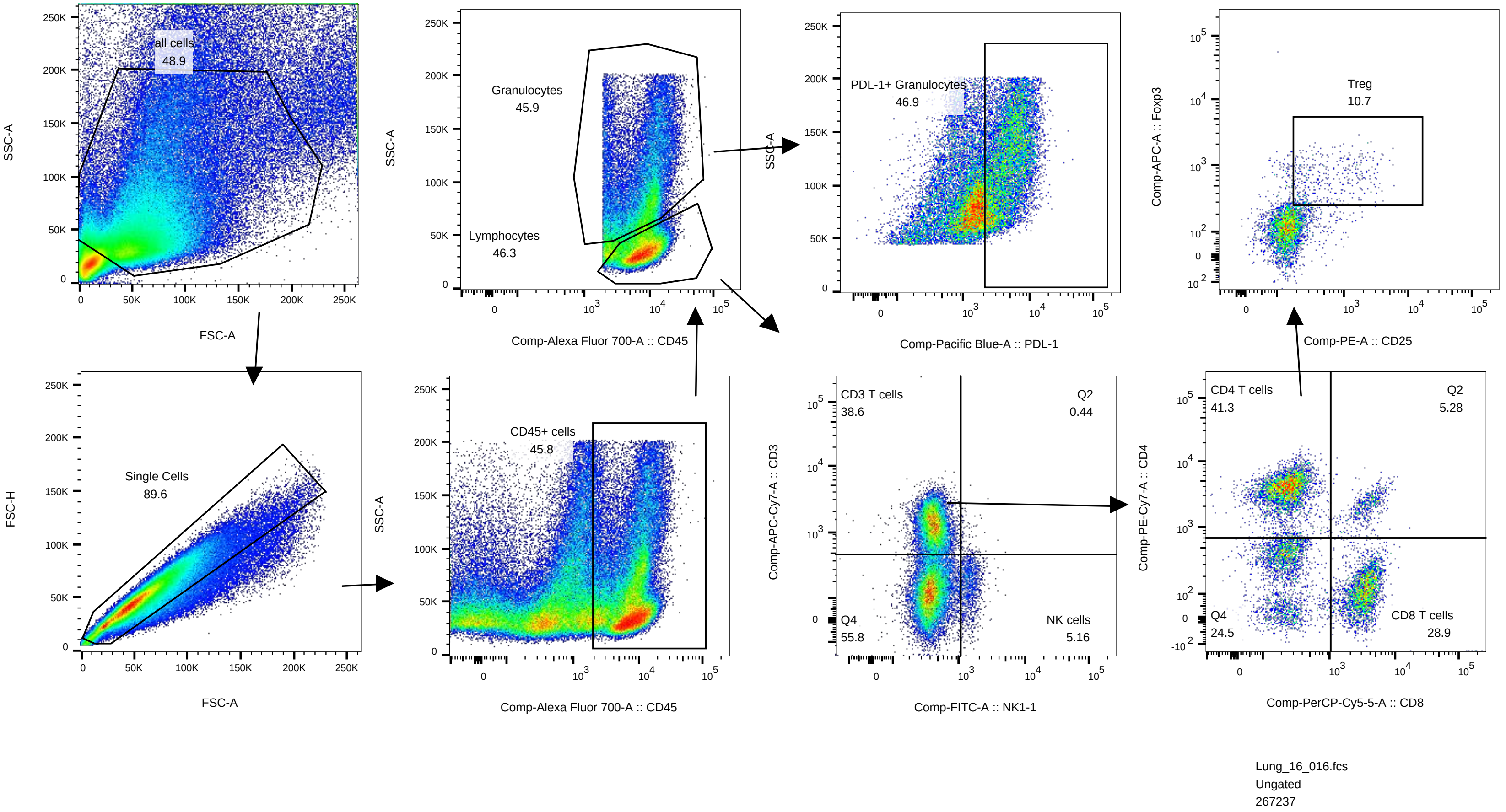

SSC-A
FSC-A
all cells
48.9
FSC-H
Single Cells
89.6
Granulocytes
45.9
Lymphocytes
46.3
Comp-Alexa Fluor 700-A :: CD45
CD45+ cells
45.8
PDL-1+ Granulocytes
46.9
Comp-Pacific Blue-A :: PDL-1
Comp-APC-Cy7-A :: CD3
CD3 T cells
38.6
Q2
0.44
Q4
55.8
NK cells
5.16
Comp-FITC-A :: NK1-1
Comp-APC-A :: Foxp3
Treg
10.7
Comp-PE-A :: CD25
Comp-PE-Cy7-A :: CD4
CD4 T cells
41.3
Q2
5.28
Q4
24.5
CD8 T cells
28.9
Comp-PerCP-Cy5-5-A :: CD8
Lung_16_016.fcs
Ungated
267237

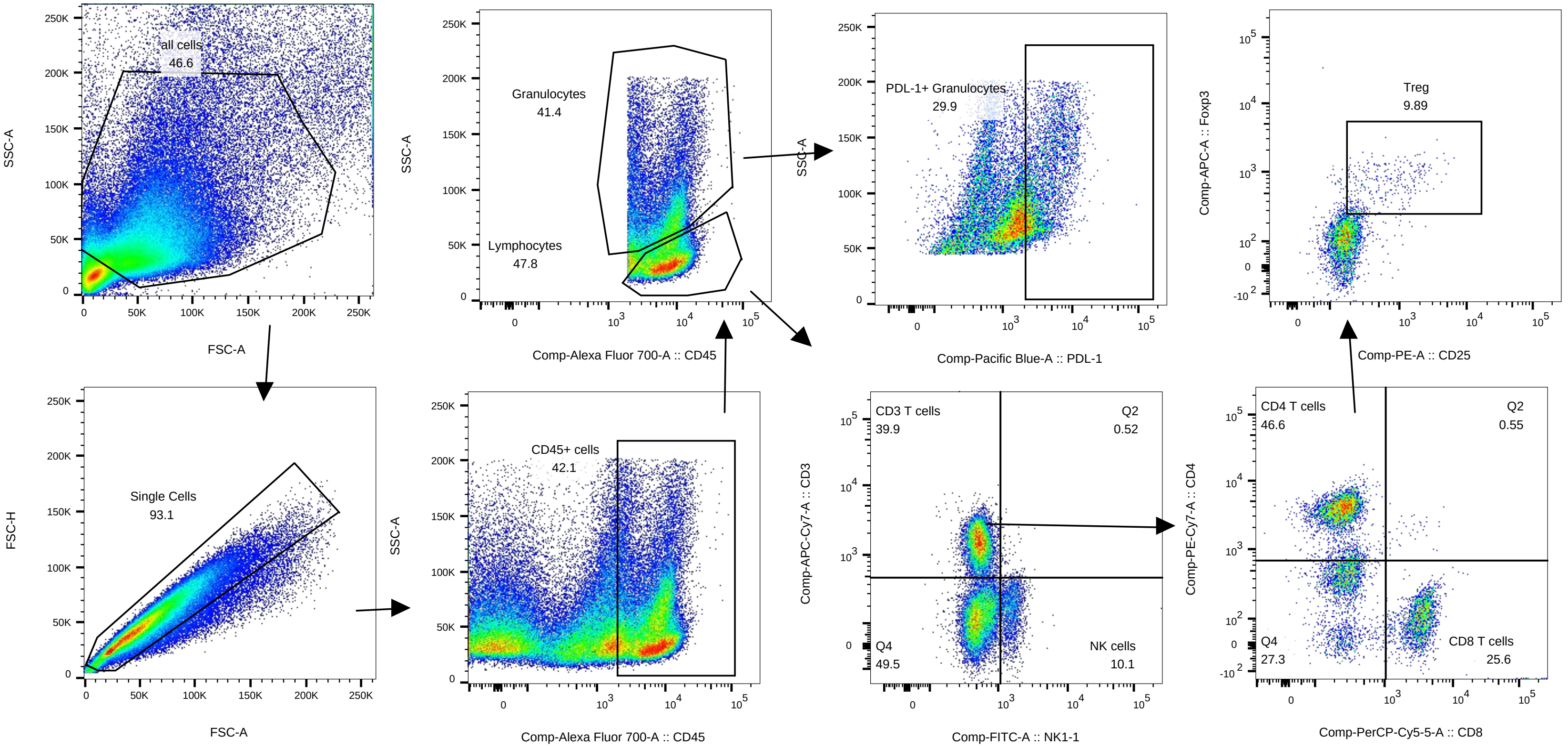

Lung_17_017.fcs
Ungated
209898

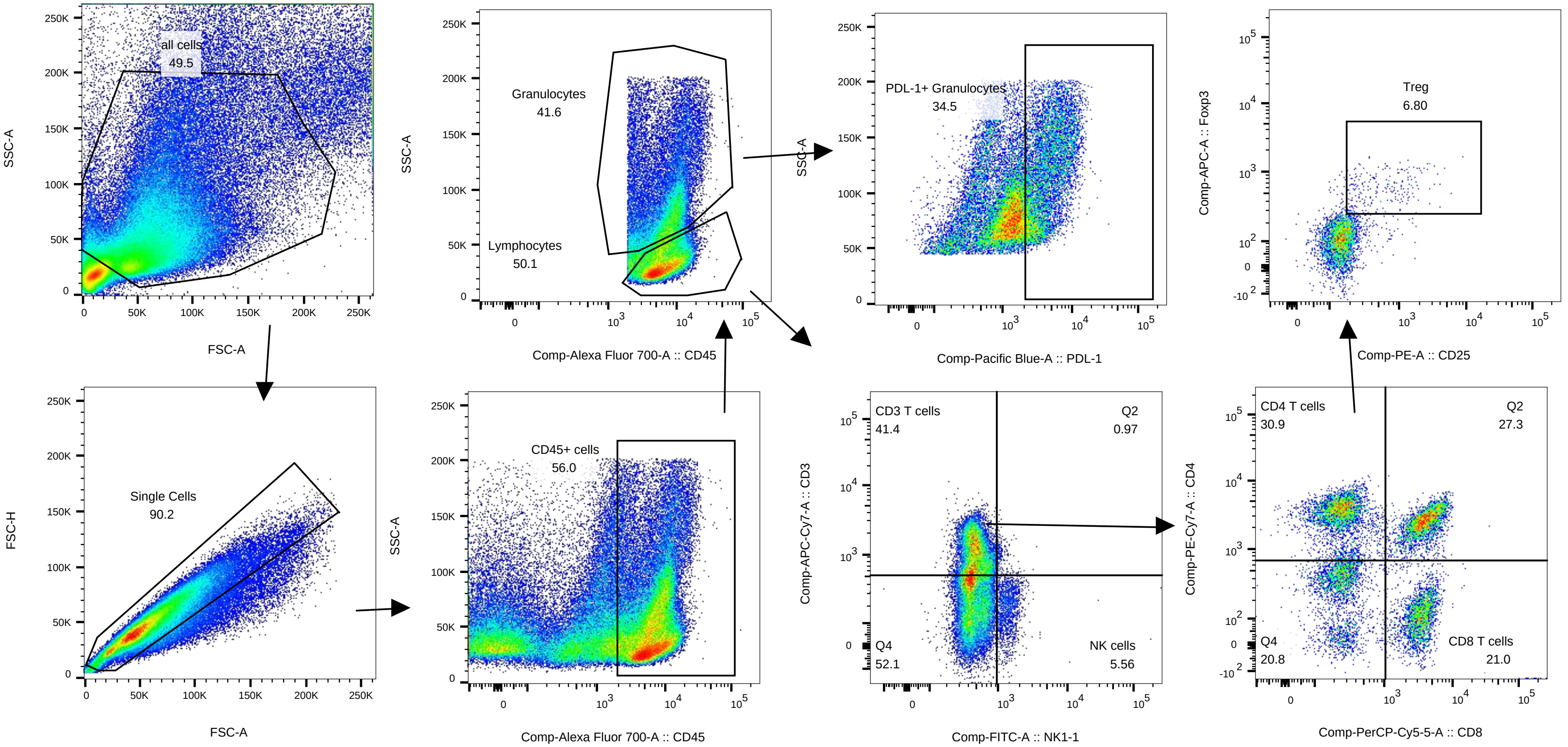

Lung_18_018.fcs
Ungated
223675

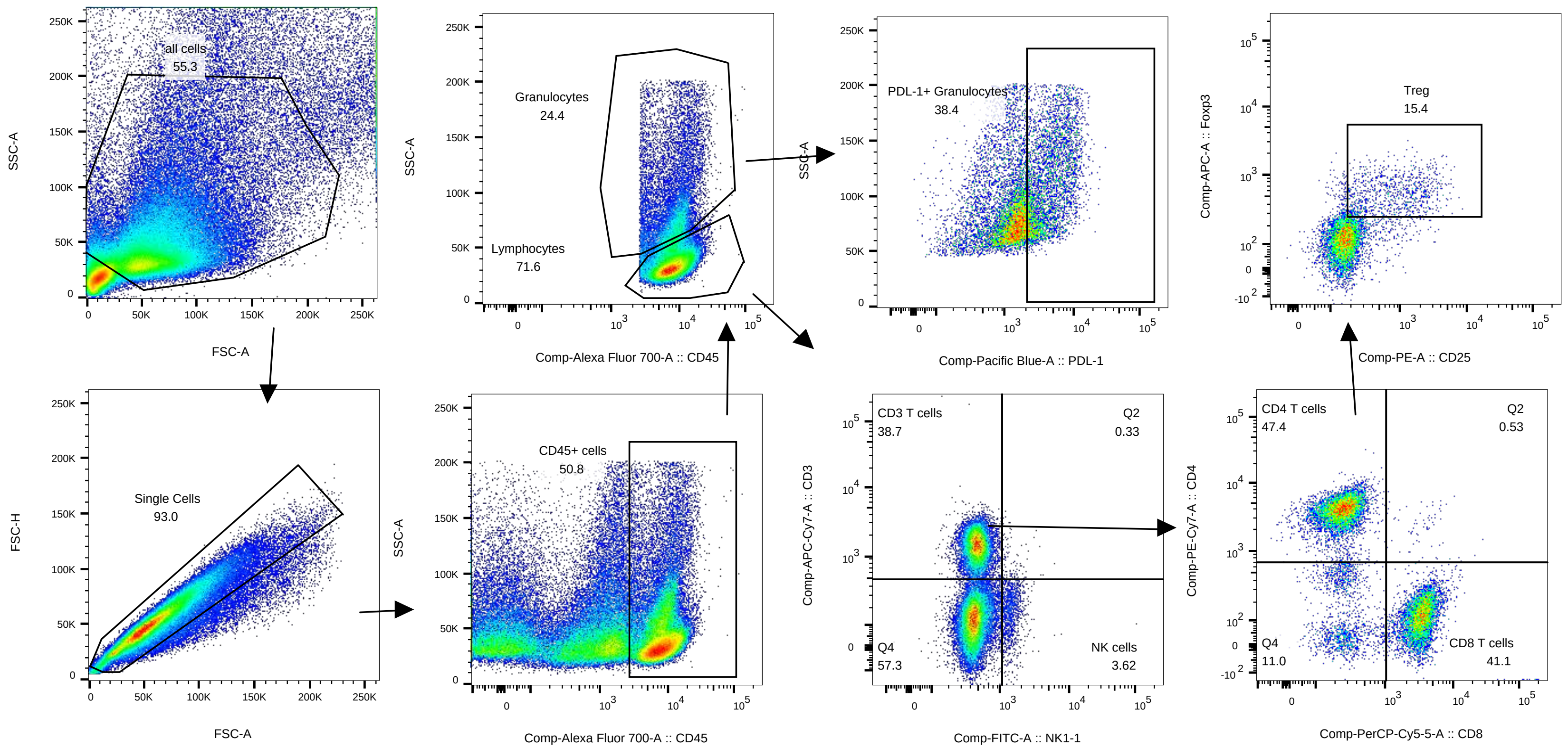

Lung_19_019.fcs
Ungated
178089

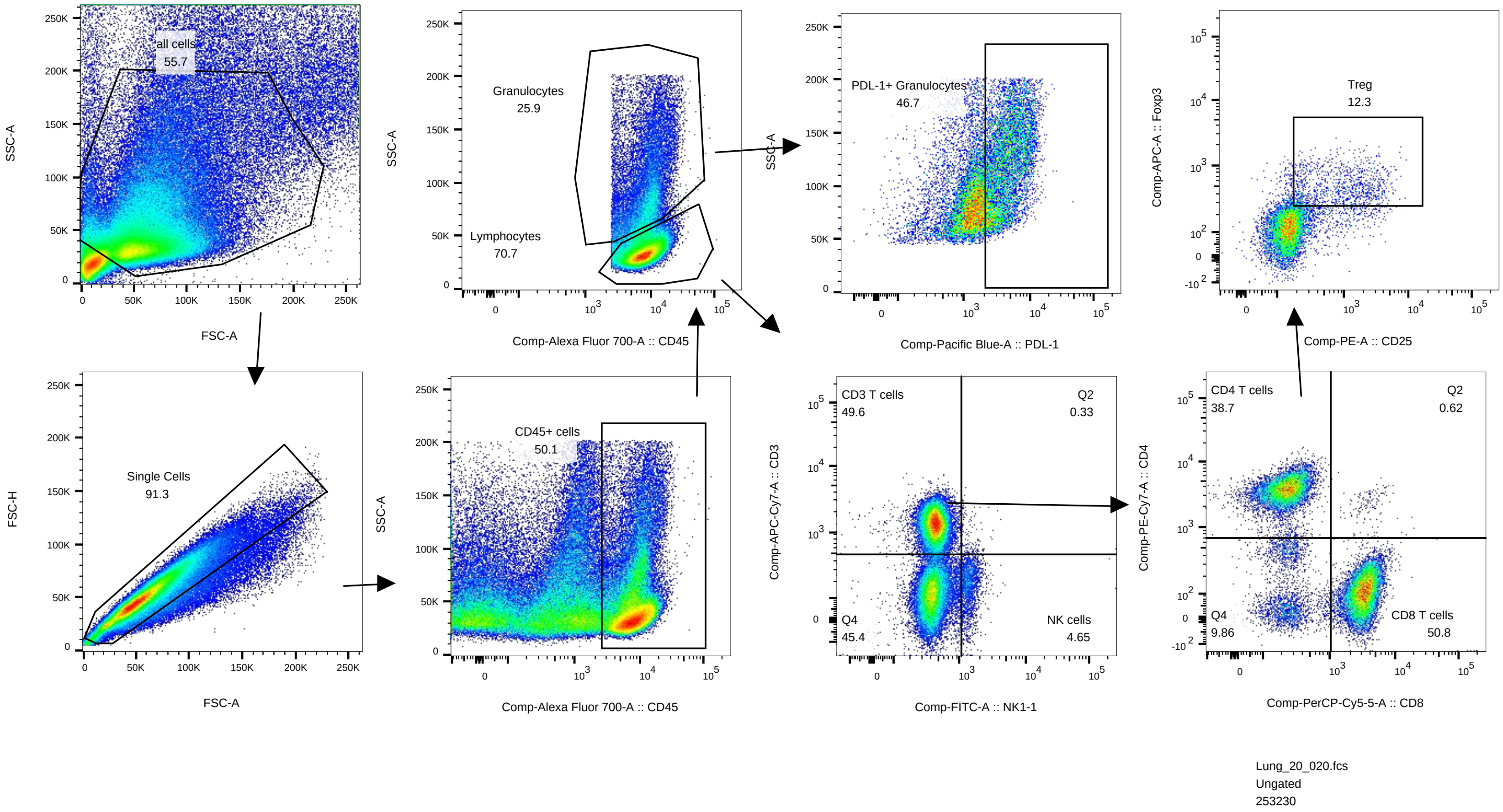
all cells
55.7
SSC-A
FSC-A
Single Cells
91.3
FSC-H
Granulocytes
25.9
Lymphocytes
70.7
Comp-Alexa Fluor 700-A :: CD45
CD45+ cells
50.1
PDL-1+ Granulocytes
46.7
Comp-Pacific Blue-A :: PDL-1
CD3 T cells
49.6
Q2
0.33
Q4
45.4
NK cells
4.65
Comp-APC-Cy7-A :: CD3
Comp-FITC-A :: NK1-1
Treg
12.3
Comp-APC-A :: Foxp3
Comp-PE-A :: CD25
CD4 T cells
38.7
Q2
0.62
Q4
9.86
CD8 T cells
50.8
Comp-PE-Cy7-A :: CD4
Comp-PerCP-Cy5-5-A :: CD8
Lung_20_020.fcs
Ungated
253230

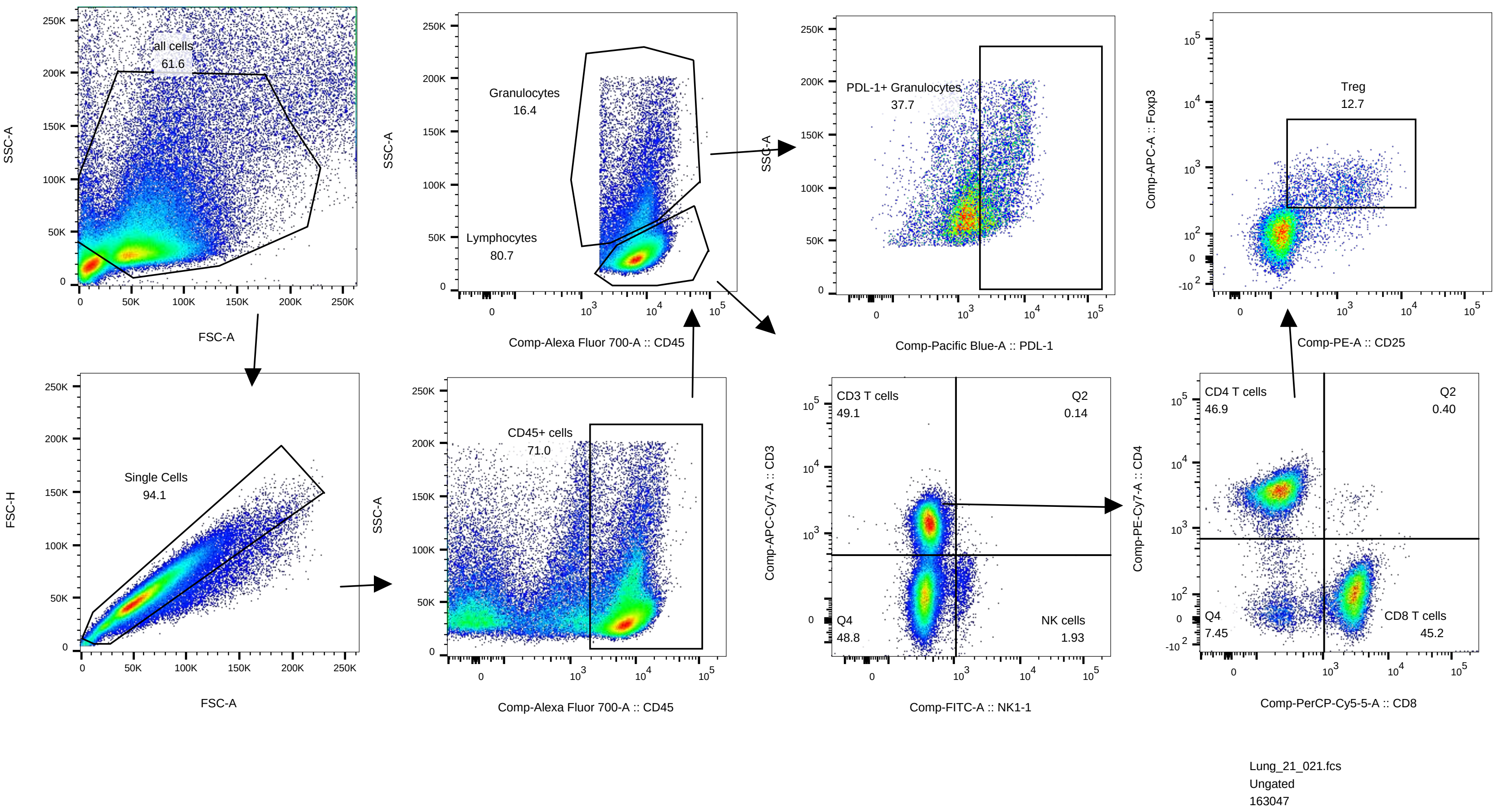

all cells
61.6
SSC-A
FSC-A
Single Cells
94.1
FSC-H
FSC-A
Granulocytes
16.4
Lymphocytes
80.7
SSC-A
Comp-Alexa Fluor 700-A :: CD45
CD45+ cells
71.0
SSC-A
Comp-Alexa Fluor 700-A :: CD45
PDL-1+ Granulocytes
37.7
SSC-A
Comp-Pacific Blue-A :: PDL-1
CD3 T cells
49.1
Q2
0.14
Q4
48.8
NK cells
1.93
Comp-APC-Cy7-A :: CD3
Comp-FITC-A :: NK1-1
Treg
12.7
Comp-APC-A :: Foxp3
Comp-PE-A :: CD25
CD4 T cells
46.9
Q2
0.40
Q4
7.45
CD8 T cells
45.2
Comp-PE-Cy7-A :: CD4
Comp-PerCP-Cy5-5-A :: CD8
Lung_21_021.fcs
Ungated
163047

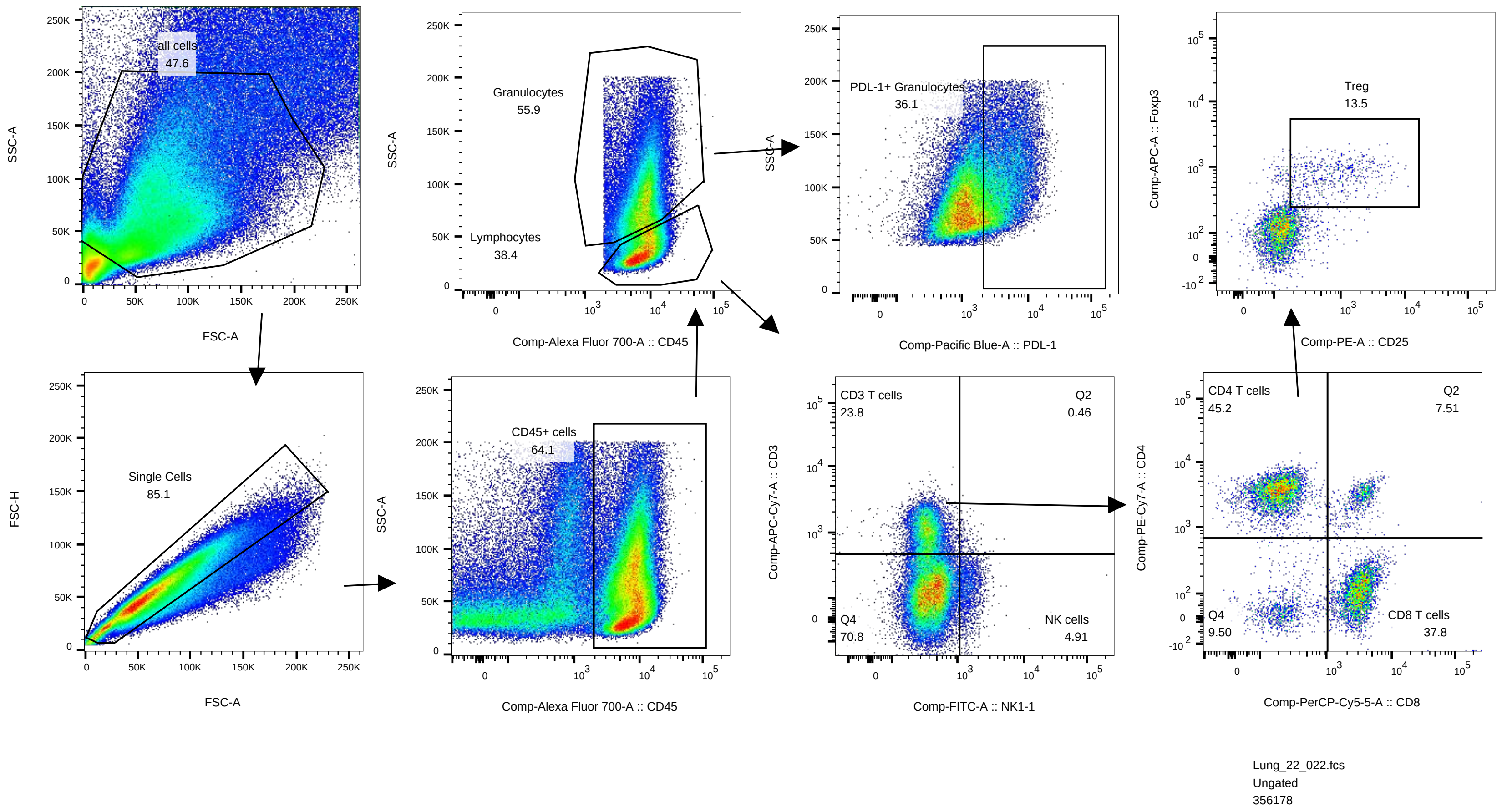
all cells
47.6
SSC-A
FSC-A
Single Cells
85.1
FSC-H
FSC-A
Granulocytes
55.9
Lymphocytes
38.4
SSC-A
Comp-Alexa Fluor 700-A :: CD45
CD45+ cells
64.1
SSC-A
Comp-Alexa Fluor 700-A :: CD45
PDL-1+ Granulocytes
36.1
SSC-A
Comp-Pacific Blue-A :: PDL-1
CD3 T cells
23.8
Q2
0.46
Q4
70.8
NK cells
4.91
Comp-APC-Cy7-A :: CD3
Comp-FITC-A :: NK1-1
Treg
13.5
Comp-APC-A :: Foxp3
Comp-PE-A :: CD25
CD4 T cells
45.2
Q2
7.51
Q4
9.50
CD8 T cells
37.8
Comp-PE-Cy7-A :: CD4
Comp-PerCP-Cy5-5-A :: CD8
Lung_22_022.fcs
Ungated
356178

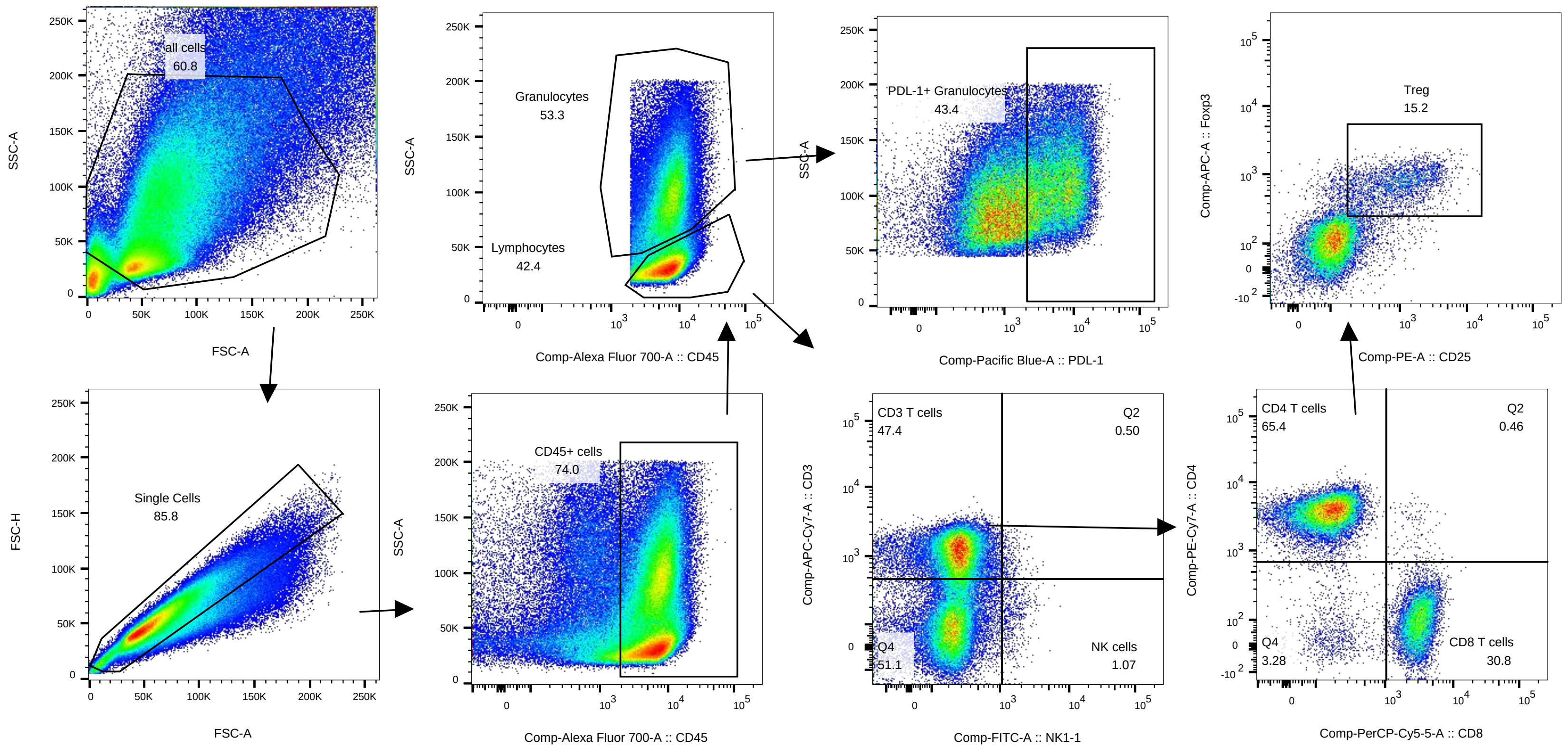

Lung_23_023.fcs
Ungated
356490

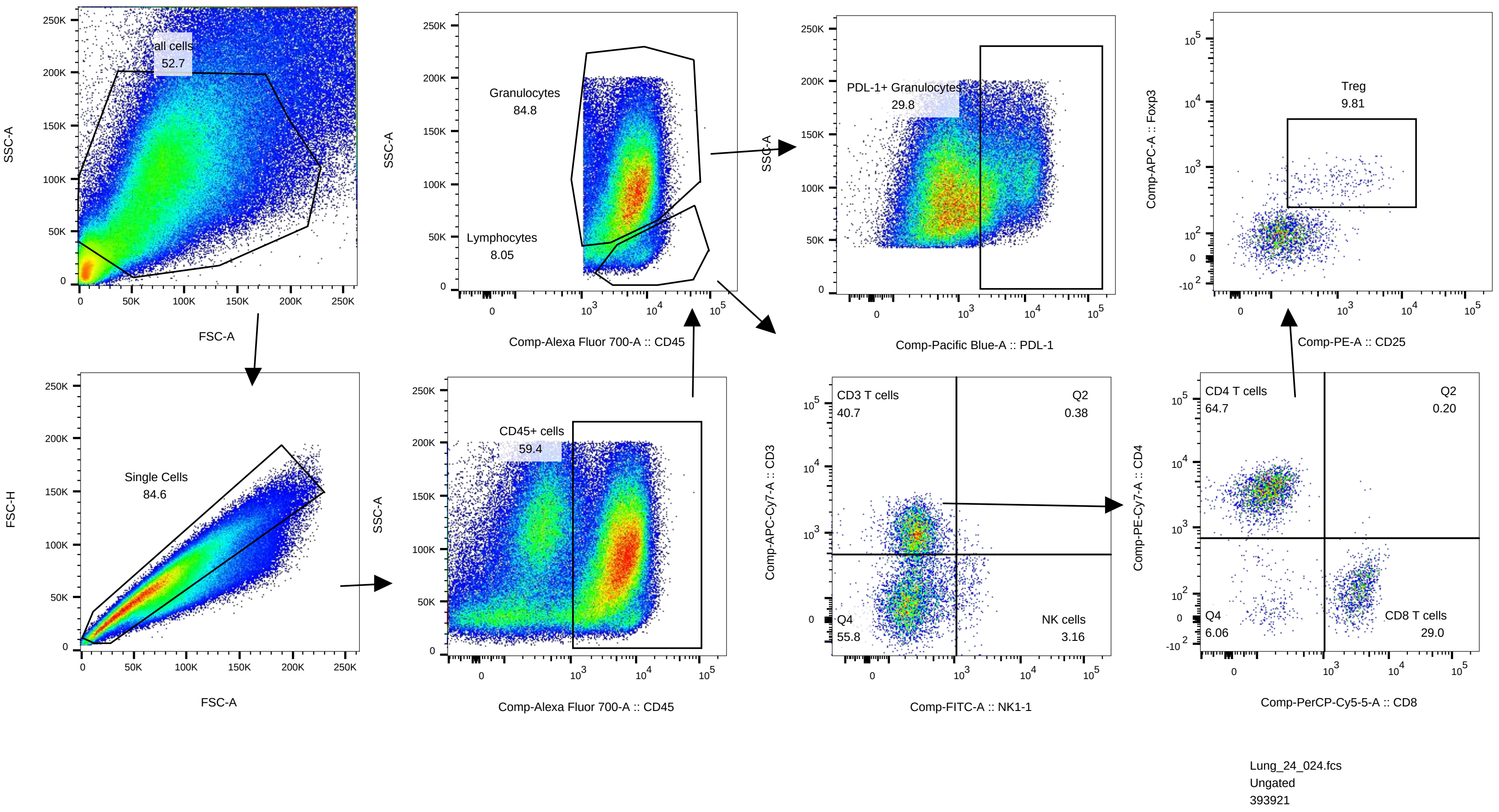

all cells
52.7
SSC-A
FSC-A
Single Cells
84.6
FSC-H
FSC-A
Granulocytes
84.8
Lymphocytes
8.05
SSC-A
Comp-Alexa Fluor 700-A :: CD45
CD45+ cells
59.4
SSC-A
Comp-Alexa Fluor 700-A :: CD45
PDL-1+ Granulocytes
29.8
SSC-A
Comp-Pacific Blue-A :: PDL-1
CD3 T cells
40.7
Q2
0.38
Q4
55.8
NK cells
3.16
Comp-APC-Cy7-A :: CD3
Comp-FITC-A :: NK1-1
Treg
9.81
Comp-APC-A :: Foxp3
Comp-PE-A :: CD25
CD4 T cells
64.7
Q2
0.20
Q4
6.06
CD8 T cells
29.0
Comp-PE-Cy7-A :: CD4
Comp-PerCP-Cy5-5-A :: CD8
Lung_24_024.fcs
Ungated
393921

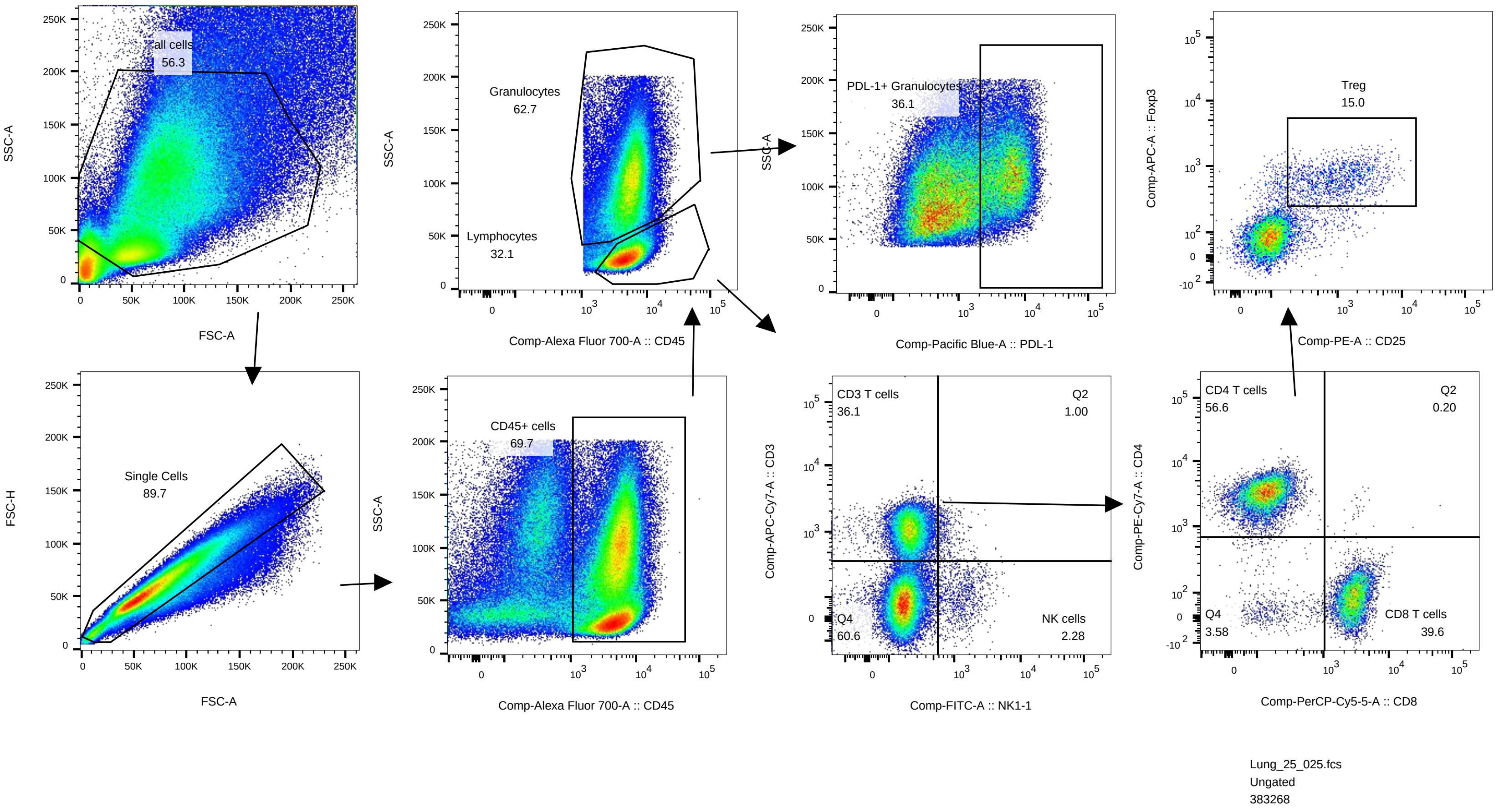
all cells
56.3
SSC-A
FSC-A
Single Cells
89.7
FSC-H
FSC-A
Granulocytes
62.7
Lymphocytes
32.1
SSC-A
Comp-Alexa Fluor 700-A :: CD45
CD45+ cells
69.7
SSC-A
Comp-Alexa Fluor 700-A :: CD45
PDL-1+ Granulocytes
36.1
SSC-A
Comp-Pacific Blue-A :: PDL-1
CD3 T cells
36.1
Q2
1.00
Q4
60.6
NK cells
2.28
Comp-APC-Cy7-A :: CD3
Comp-FITC-A :: NK1-1
Treg
15.0
Comp-APC-A :: Foxp3
Comp-PE-A :: CD25
CD4 T cells
56.6
Q2
0.20
Q4
3.58
CD8 T cells
39.6
Comp-PE-Cy7-A :: CD4
Comp-PerCP-Cy5-5-A :: CD8
Lung_25_025.fcs
Ungated
383268

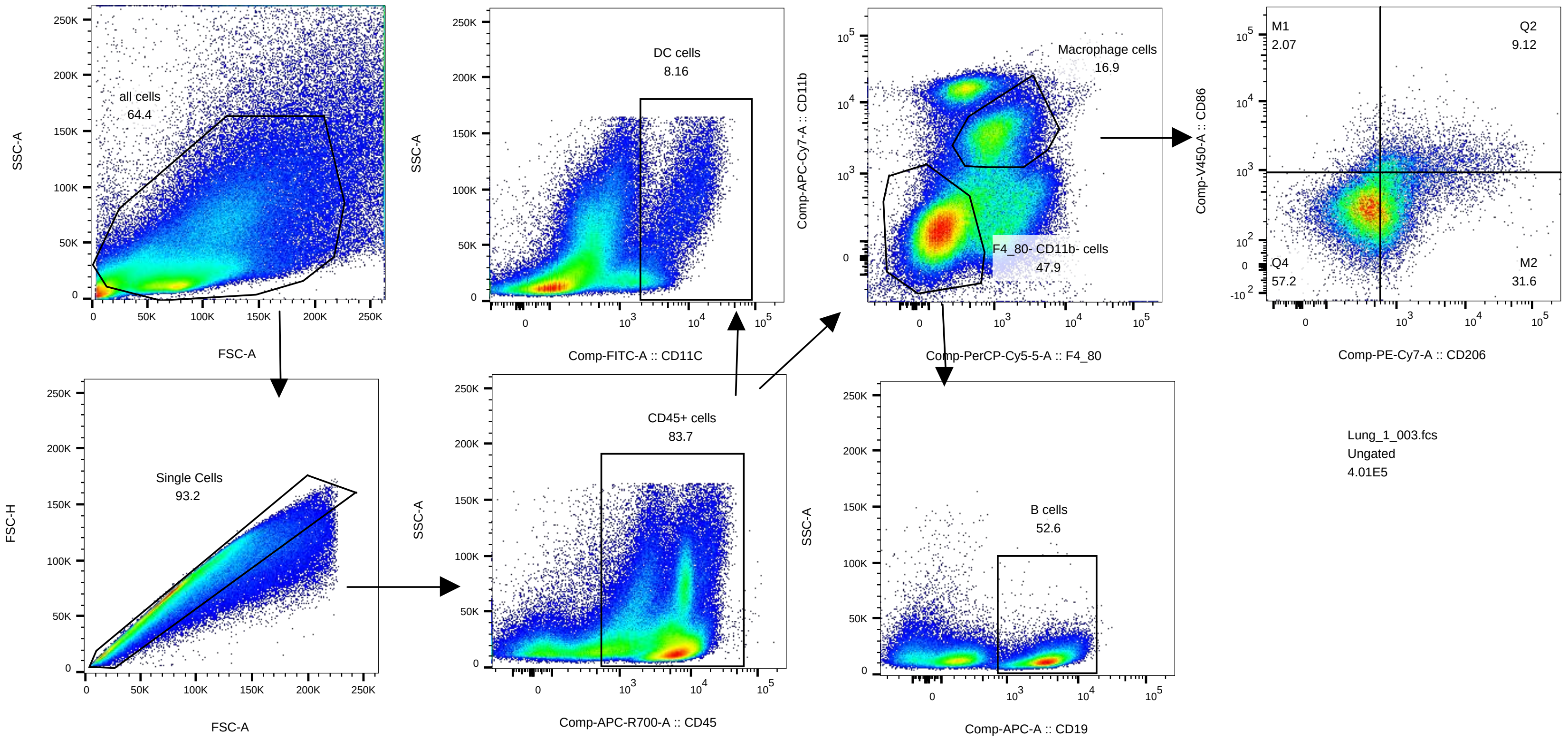
all cells
64.4
SSC-A
FSC-A
Single Cells
93.2
FSC-H
DC cells
8.16
Comp-FITC-A :: CD11C
CD45+ cells
83.7
Comp-APC-R700-A :: CD45
Macrophage cells
16.9
F4_80- CD11b- cells
47.9
Comp-APC-Cy7-A :: CD11b
Comp-PerCP-Cy5-5-A :: F4_80
B cells
52.6
Comp-APC-A :: CD19
M1
2.07
Q2
9.12
Q4
57.2
M2
31.6
Comp-V450-A :: CD86
Comp-PE-Cy7-A :: CD206
Lung_1_003.fcs
Ungated
4.01E5

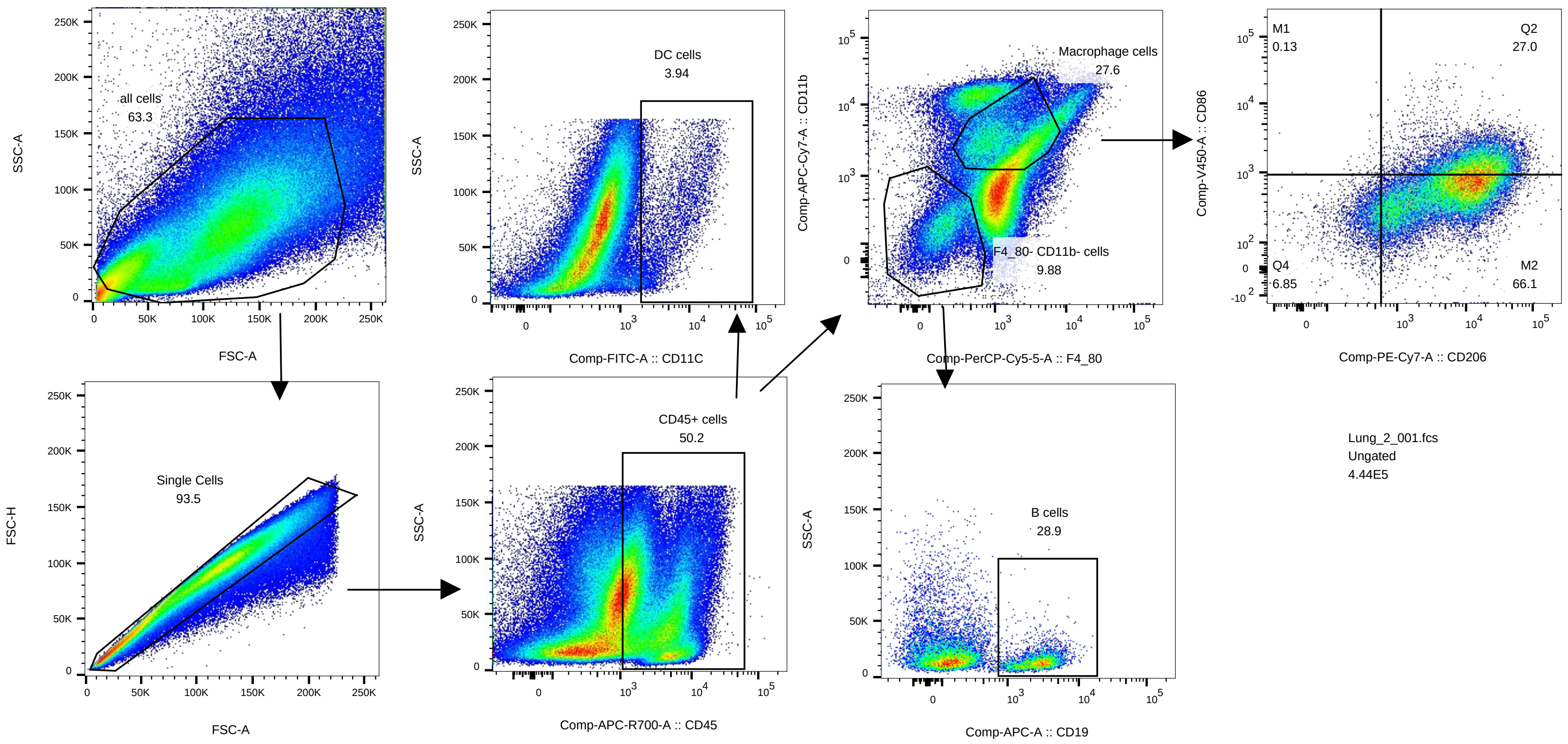

all cells
63.3
SSC-A
FSC-A
Single Cells
93.5
FSC-H
DC cells
3.94
Comp-FITC-A :: CD11C
CD45+ cells
50.2
Comp-APC-R700-A :: CD45
Macrophage cells
27.6
F4_80- CD11b- cells
9.88
Comp-APC-Cy7-A :: CD11b
Comp-PerCP-Cy5-5-A :: F4_80
B cells
28.9
Comp-APC-A :: CD19
M1
0.13
Q2
27.0
Q4
6.85
M2
66.1
Comp-V450-A :: CD86
Comp-PE-Cy7-A :: CD206
Lung_2_001.fcs
Ungated
4.44E5

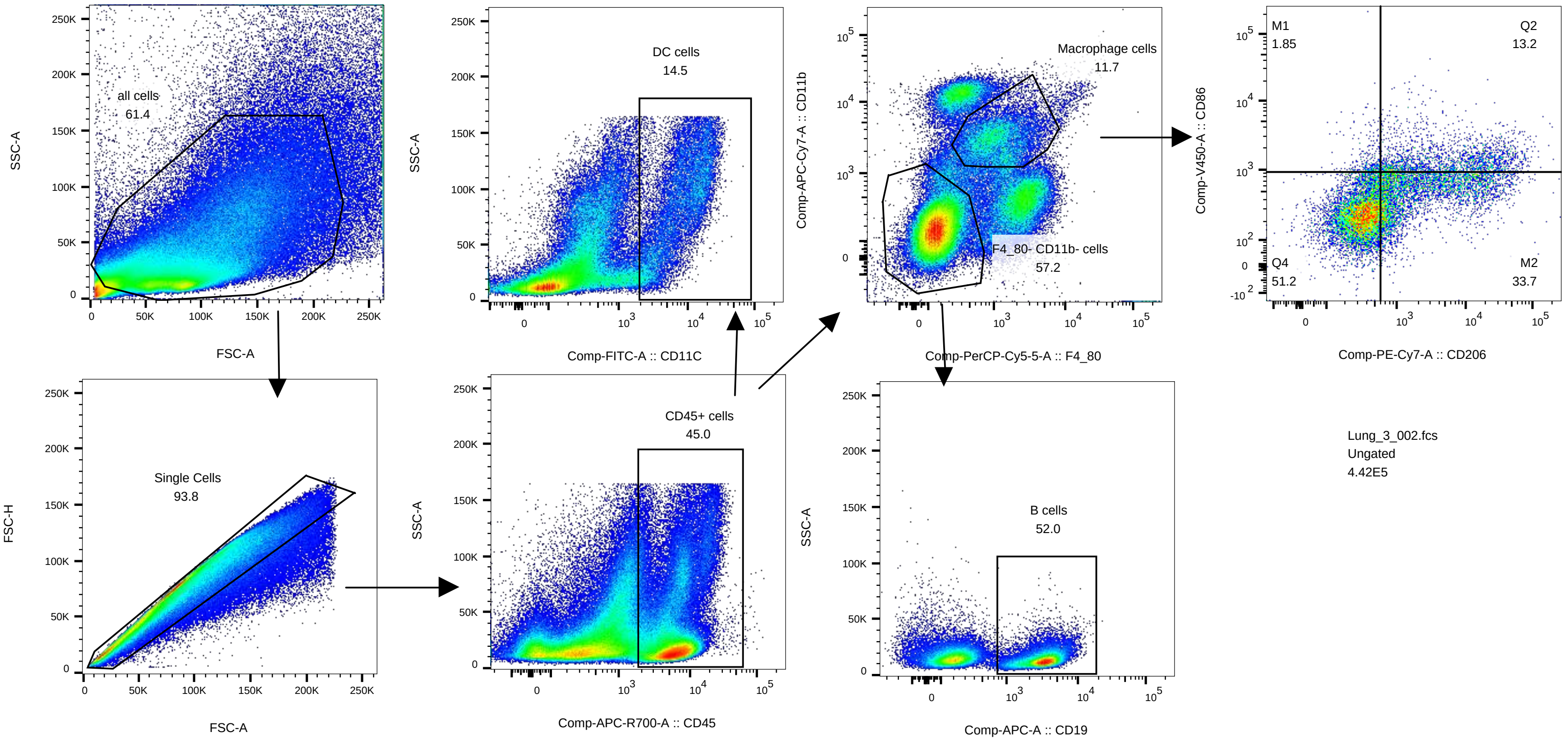
all cells
61.4
SSC-A
FSC-A
Single Cells
93.8
FSC-H
FSC-A
DC cells
14.5
SSC-A
Comp-FITC-A :: CD11C
CD45+ cells
45.0
SSC-A
Comp-APC-R700-A :: CD45
Macrophage cells
11.7
F4_80- CD11b- cells
57.2
Comp-APC-Cy7-A :: CD11b
Comp-PerCP-Cy5-5-A :: F4_80
B cells
52.0
SSC-A
Comp-APC-A :: CD19
M1
1.85
Q2
13.2
Q4
51.2
M2
33.7
Comp-V450-A :: CD86
Comp-PE-Cy7-A :: CD206
Lung_3_002.fcs
Ungated
4.42E5

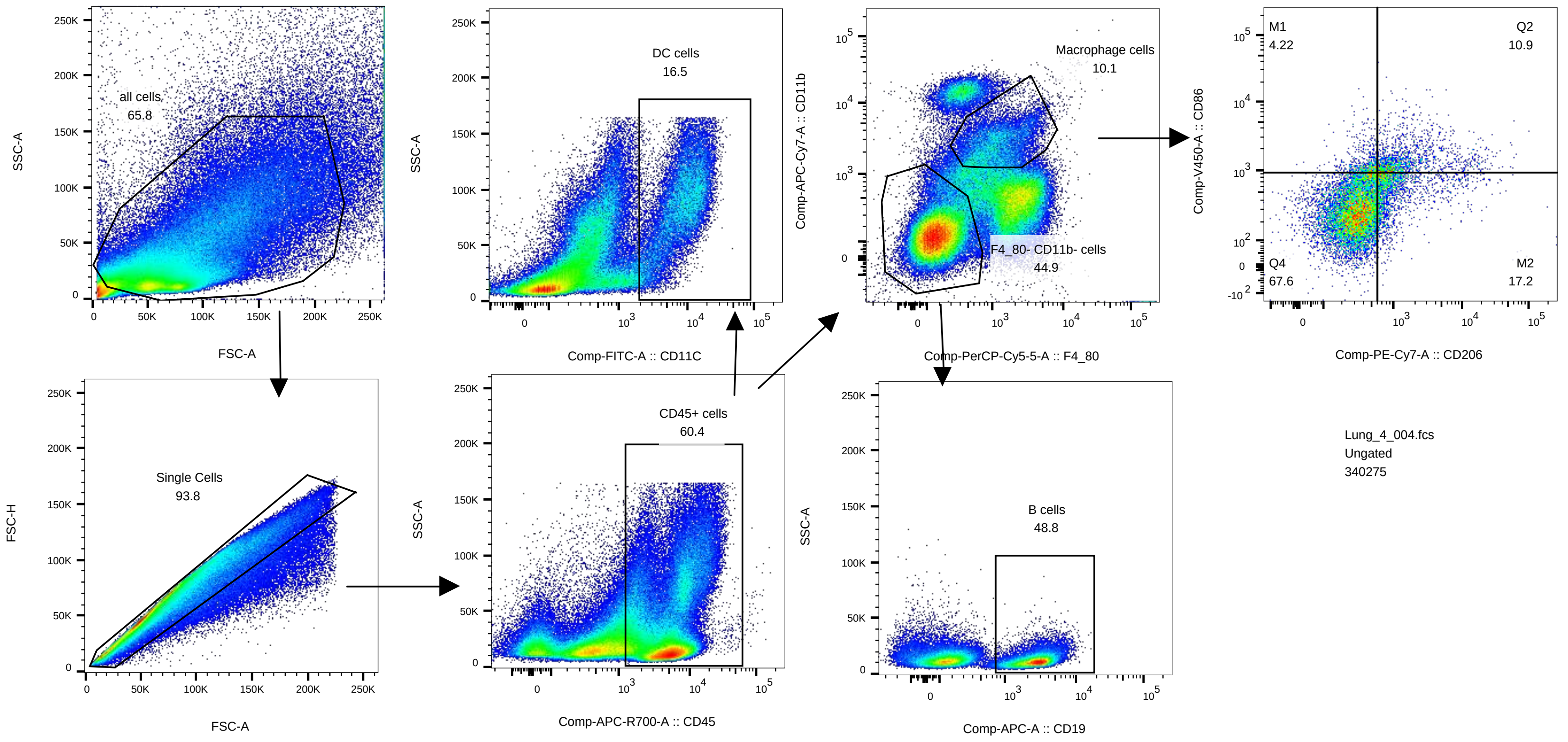
all cells
65.8
SSC-A
FSC-A
Single Cells
93.8
FSC-H
FSC-A
DC cells
16.5
SSC-A
Comp-FITC-A :: CD11C
CD45+ cells
60.4
SSC-A
Comp-APC-R700-A :: CD45
Macrophage cells
10.1
F4_80- CD11b- cells
44.9
Comp-APC-Cy7-A :: CD11b
Comp-PerCP-Cy5-5-A :: F4_80
B cells
48.8
SSC-A
Comp-APC-A :: CD19
M1
4.22
Q2
10.9
Q4
67.6
M2
17.2
Comp-V450-A :: CD86
Comp-PE-Cy7-A :: CD206
Lung_4_004.fcs
Ungated
340275

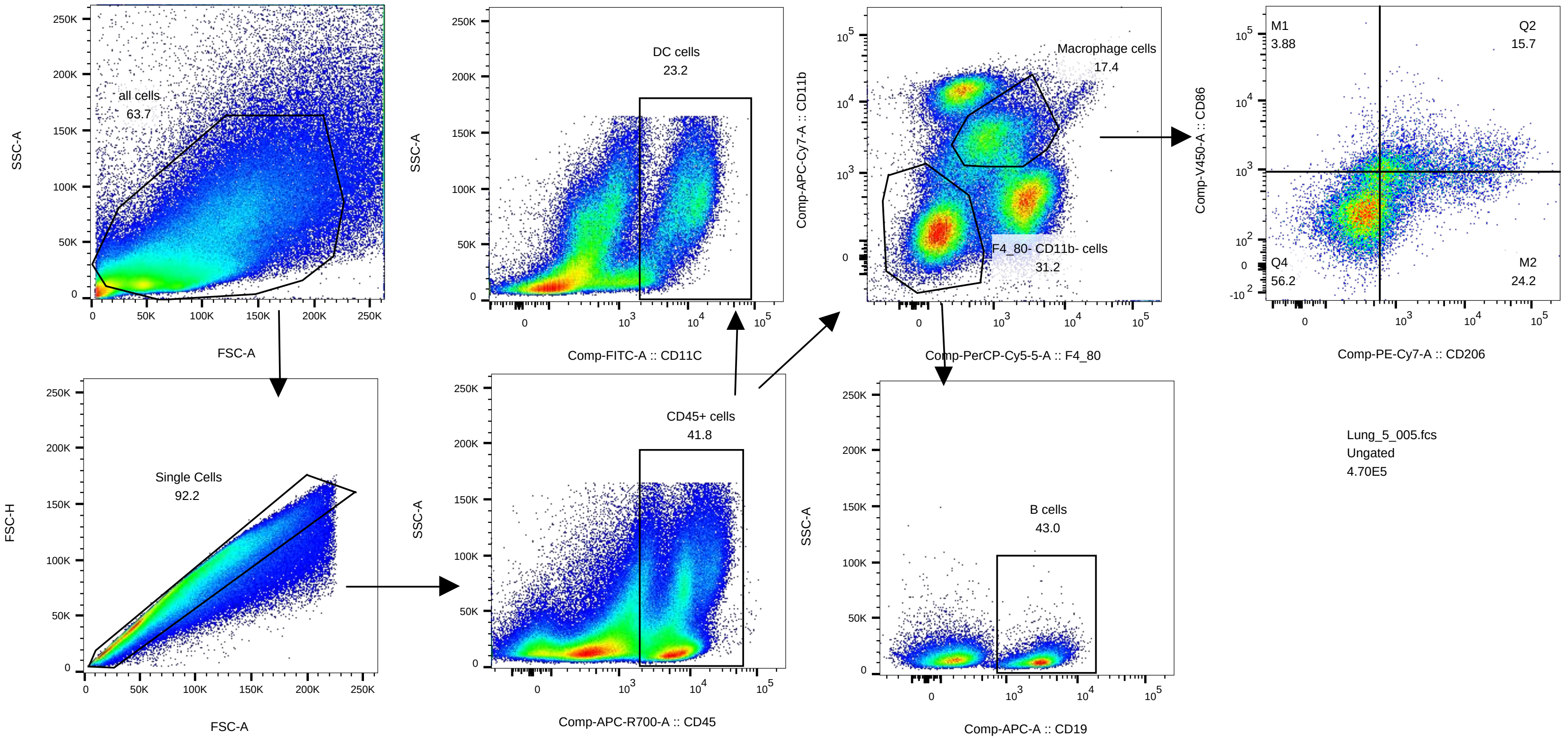
all cells
63.7
SSC-A
FSC-A
Single Cells
92.2
FSC-H
DC cells
23.2
Comp-FITC-A :: CD11C
CD45+ cells
41.8
Comp-APC-R700-A :: CD45
Macrophage cells
17.4
F4_80- CD11b- cells
31.2
Comp-APC-Cy7-A :: CD11b
Comp-PerCP-Cy5-5-A :: F4_80
B cells
43.0
Comp-APC-A :: CD19
M1
3.88
Q2
15.7
Q4
56.2
M2
24.2
Comp-V450-A :: CD86
Comp-PE-Cy7-A :: CD206
Lung_5_005.fcs
Ungated
4.70E5

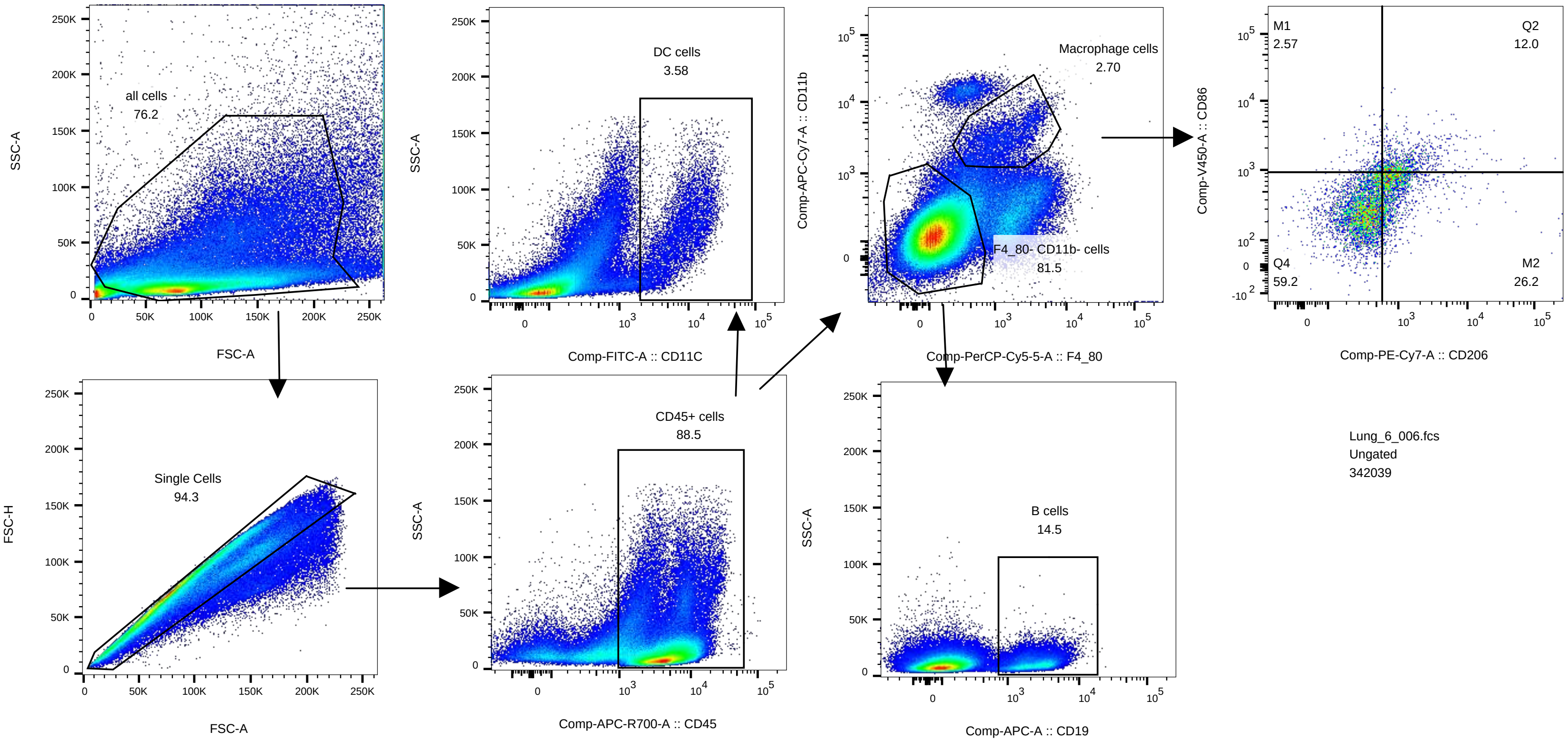
all cells
76.2
SSC-A
FSC-A
DC cells
3.58
SSC-A
Comp-FITC-A :: CD11C
Macrophage cells
2.70
F4_80- CD11b- cells
81.5
Comp-APC-Cy7-A :: CD11b
Comp-PerCP-Cy5-5-A :: F4_80
M1
2.57
Q2
12.0
Q4
59.2
M2
26.2
Comp-V450-A :: CD86
Comp-PE-Cy7-A :: CD206
Single Cells
94.3
FSC-H
FSC-A
CD45+ cells
88.5
SSC-A
Comp-APC-R700-A :: CD45
B cells
14.5
SSC-A
Comp-APC-A :: CD19
Lung_6_006.fcs
Ungated
342039

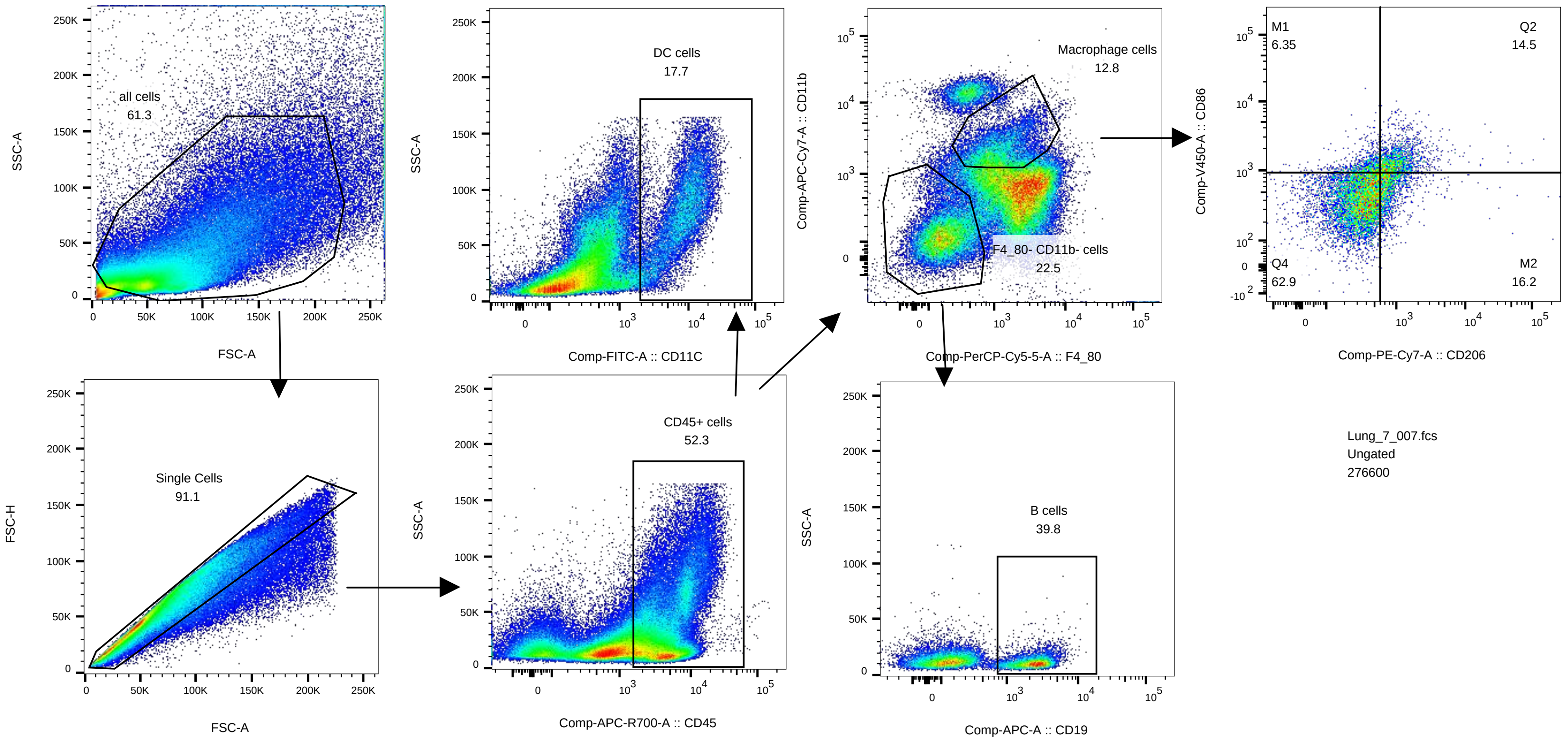

all cells
61.3
FSC-A
SSC-A
Single Cells
91.1
FSC-H
FSC-A
DC cells
17.7
Comp-FITC-A :: CD11C
CD45+ cells
52.3
Comp-APC-R700-A :: CD45
Macrophage cells
12.8
F4_80- CD11b- cells
22.5
Comp-APC-Cy7-A :: CD11b
Comp-PerCP-Cy5-5-A :: F4_80
B cells
39.8
Comp-APC-A :: CD19
M1
6.35
Q2
14.5
Q4
62.9
M2
16.2
Comp-V450-A :: CD86
Comp-PE-Cy7-A :: CD206
Lung_7_007.fcs
Ungated
276600

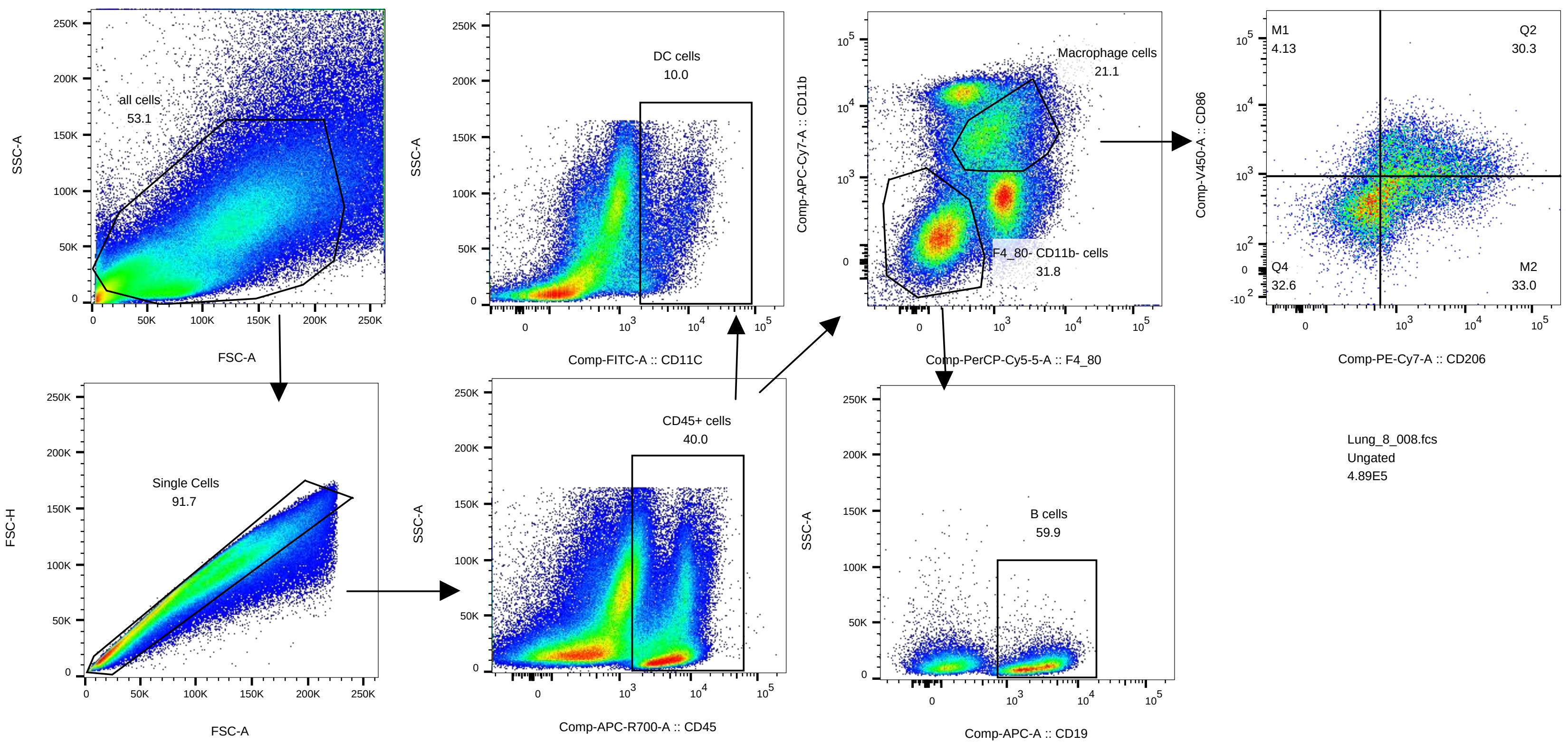
all cells
53.1
SSC-A
FSC-A
Single Cells
91.7
FSC-H
FSC-A
DC cells
10.0
SSC-A
Comp-FITC-A :: CD11C
CD45+ cells
40.0
SSC-A
Comp-APC-R700-A :: CD45
Macrophage cells
21.1
F4_80- CD11b- cells
31.8
Comp-APC-Cy7-A :: CD11b
Comp-PerCP-Cy5-5-A :: F4_80
B cells
59.9
SSC-A
Comp-APC-A :: CD19
M1
4.13
Q2
30.3
Q4
32.6
M2
33.0
Comp-V450-A :: CD86
Comp-PE-Cy7-A :: CD206
Lung_8_008.fcs
Ungated
4.89E5

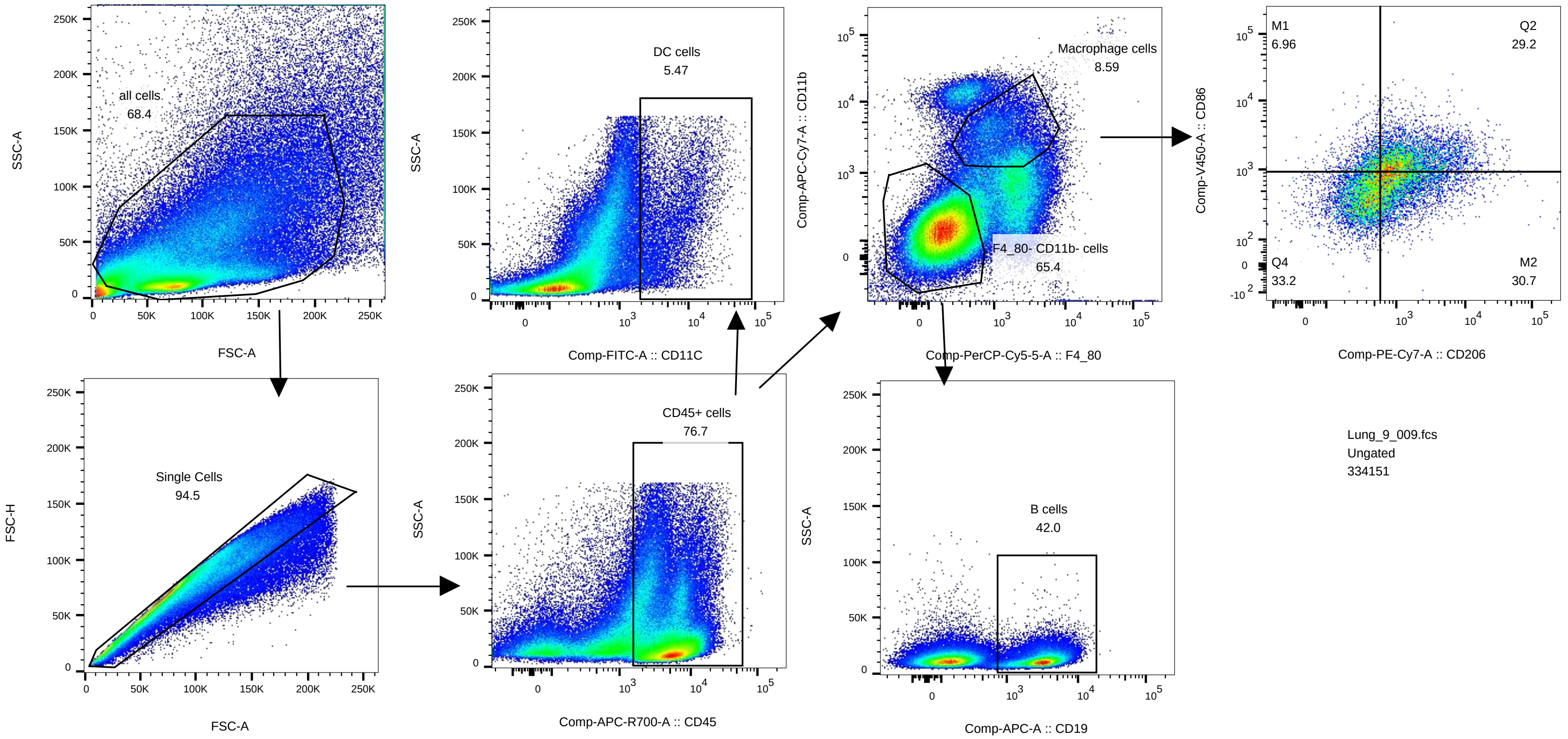
all cells
68.4
SSC-A
FSC-A
Single Cells
94.5
FSC-H
DC cells
5.47
Comp-FITC-A :: CD11C
CD45+ cells
76.7
Comp-APC-R700-A :: CD45
Macrophage cells
8.59
F4_80- CD11b- cells
65.4
Comp-APC-Cy7-A :: CD11b
Comp-PerCP-Cy5-5-A :: F4_80
B cells
42.0
Comp-APC-A :: CD19
M1
6.96
Q2
29.2
Q4
33.2
M2
30.7
Comp-V450-A :: CD86
Comp-PE-Cy7-A :: CD206
Lung_9_009.fcs
Ungated
334151

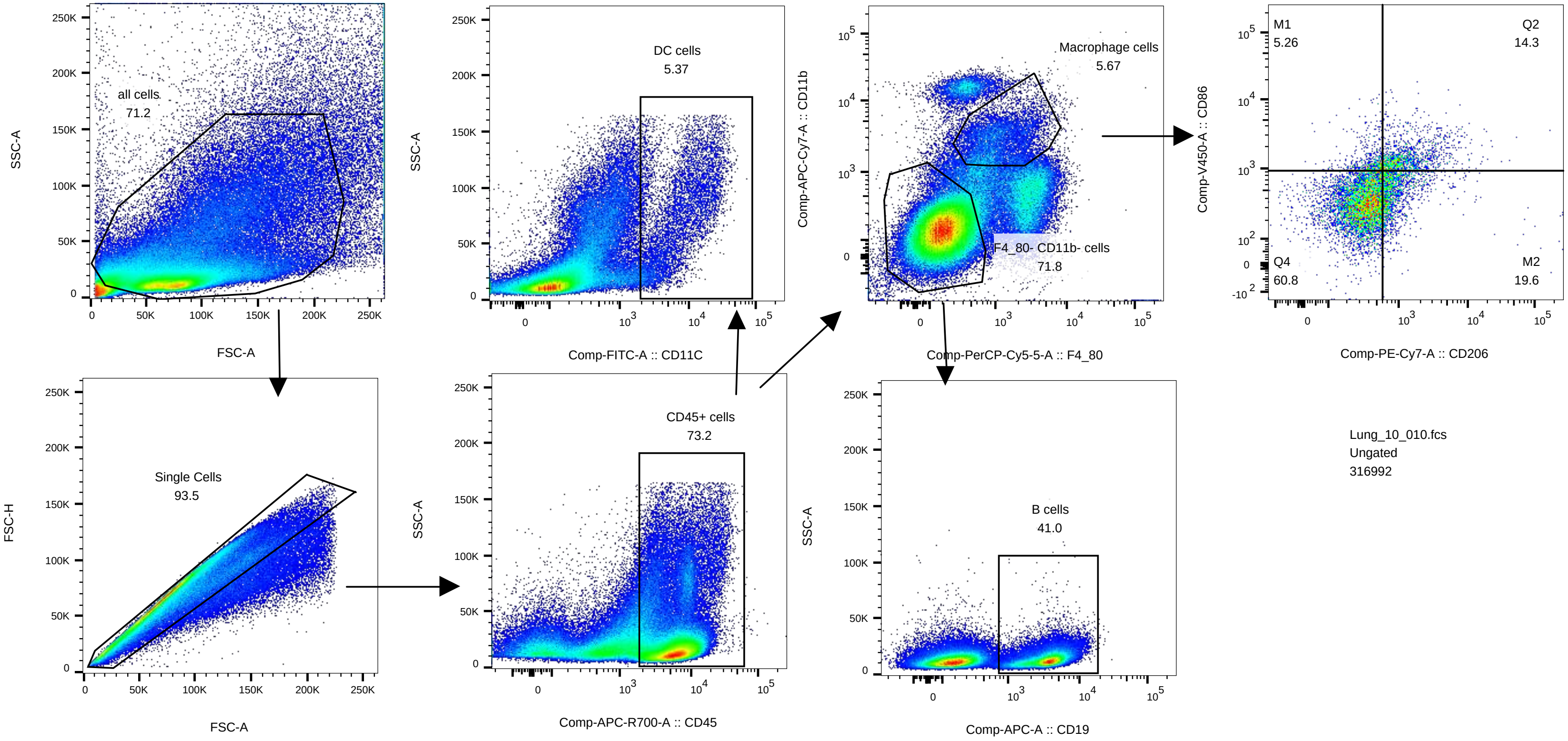
all cells
71.2
SSC-A
FSC-A
Single Cells
93.5
FSC-H
FSC-A
DC cells
5.37
SSC-A
Comp-FITC-A :: CD11C
CD45+ cells
73.2
SSC-A
Comp-APC-R700-A :: CD45
Macrophage cells
5.67
F4_80- CD11b- cells
71.8
Comp-APC-Cy7-A :: CD11b
Comp-PerCP-Cy5-5-A :: F4_80
B cells
41.0
SSC-A
Comp-APC-A :: CD19
M1
5.26
Q2
14.3
Q4
60.8
M2
19.6
Comp-V450-A :: CD86
Comp-PE-Cy7-A :: CD206
Lung_10_010.fcs
Ungated
316992

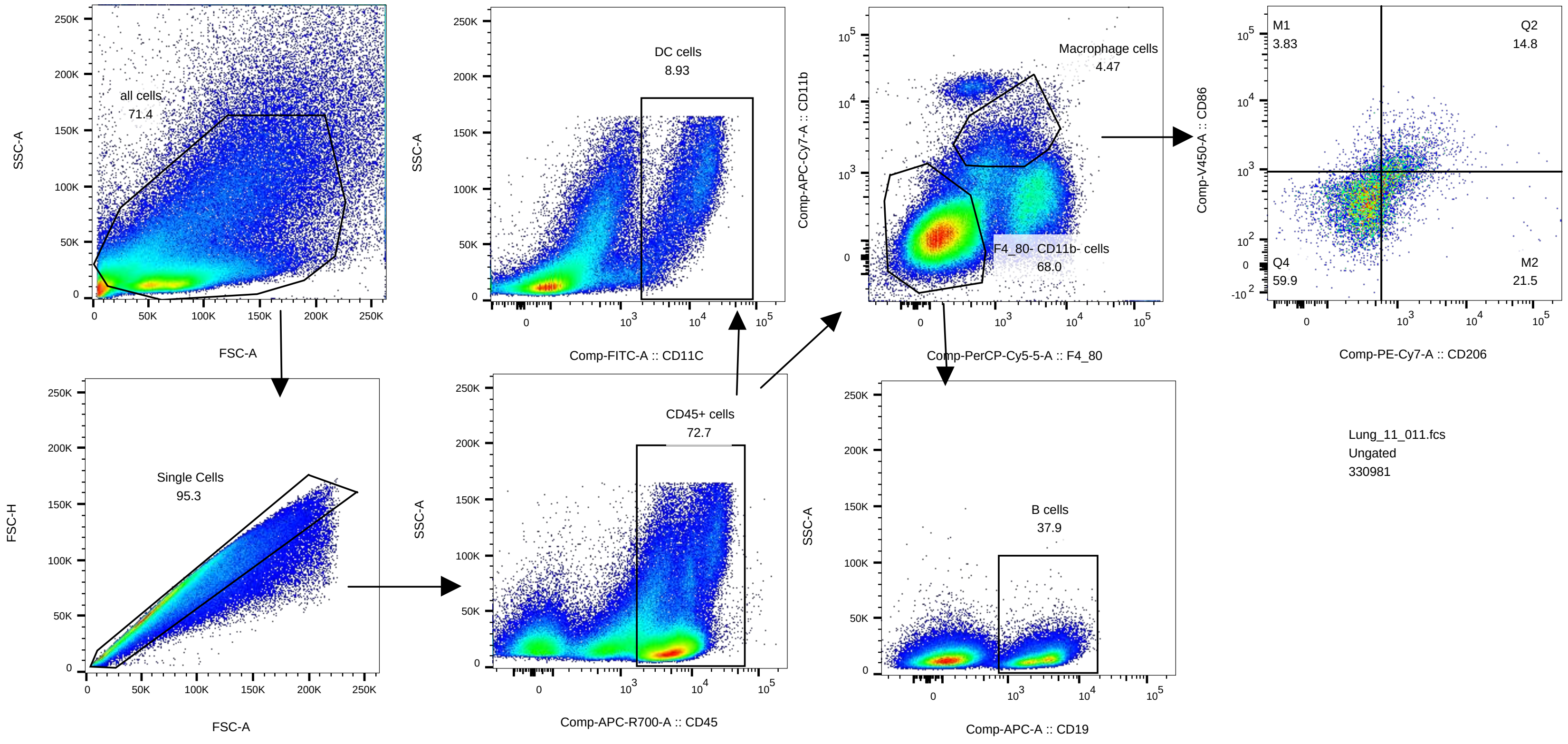
all cells
71.4
SSC-A
FSC-A
DC cells
8.93
SSC-A
Comp-FITC-A :: CD11C
Macrophage cells
4.47
F4_80- CD11b- cells
68.0
Comp-APC-Cy7-A :: CD11b
Comp-PerCP-Cy5-5-A :: F4_80
M1
3.83
Q2
14.8
Q4
59.9
M2
21.5
Comp-V450-A :: CD86
Comp-PE-Cy7-A :: CD206
Single Cells
95.3
FSC-H
FSC-A
CD45+ cells
72.7
SSC-A
Comp-APC-R700-A :: CD45
B cells
37.9
SSC-A
Comp-APC-A :: CD19
Lung_11_011.fcs
Ungated
330981

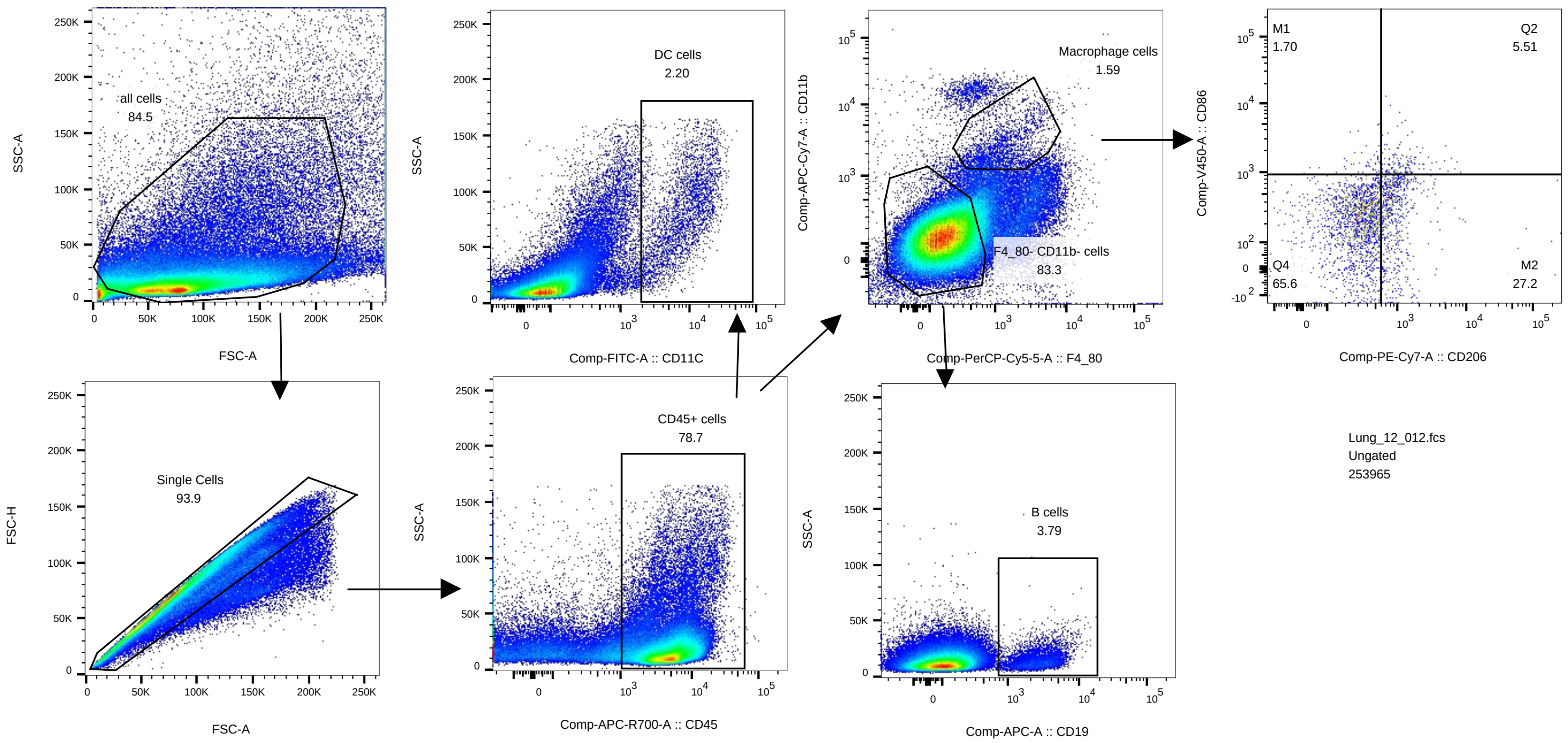

all cells
84.5
SSC-A
FSC-A
Single Cells
93.9
FSC-H
FSC-A
DC cells
2.20
SSC-A
Comp-FITC-A :: CD11C
CD45+ cells
78.7
SSC-A
Comp-APC-R700-A :: CD45
Macrophage cells
1.59
F4_80- CD11b- cells
83.3
Comp-APC-Cy7-A :: CD11b
Comp-PerCP-Cy5-5-A :: F4_80
B cells
3.79
SSC-A
Comp-APC-A :: CD19
M1
1.70
Q2
5.51
Q4
65.6
M2
27.2
Comp-V450-A :: CD86
Comp-PE-Cy7-A :: CD206
Lung_12_012.fcs
Ungated
253965

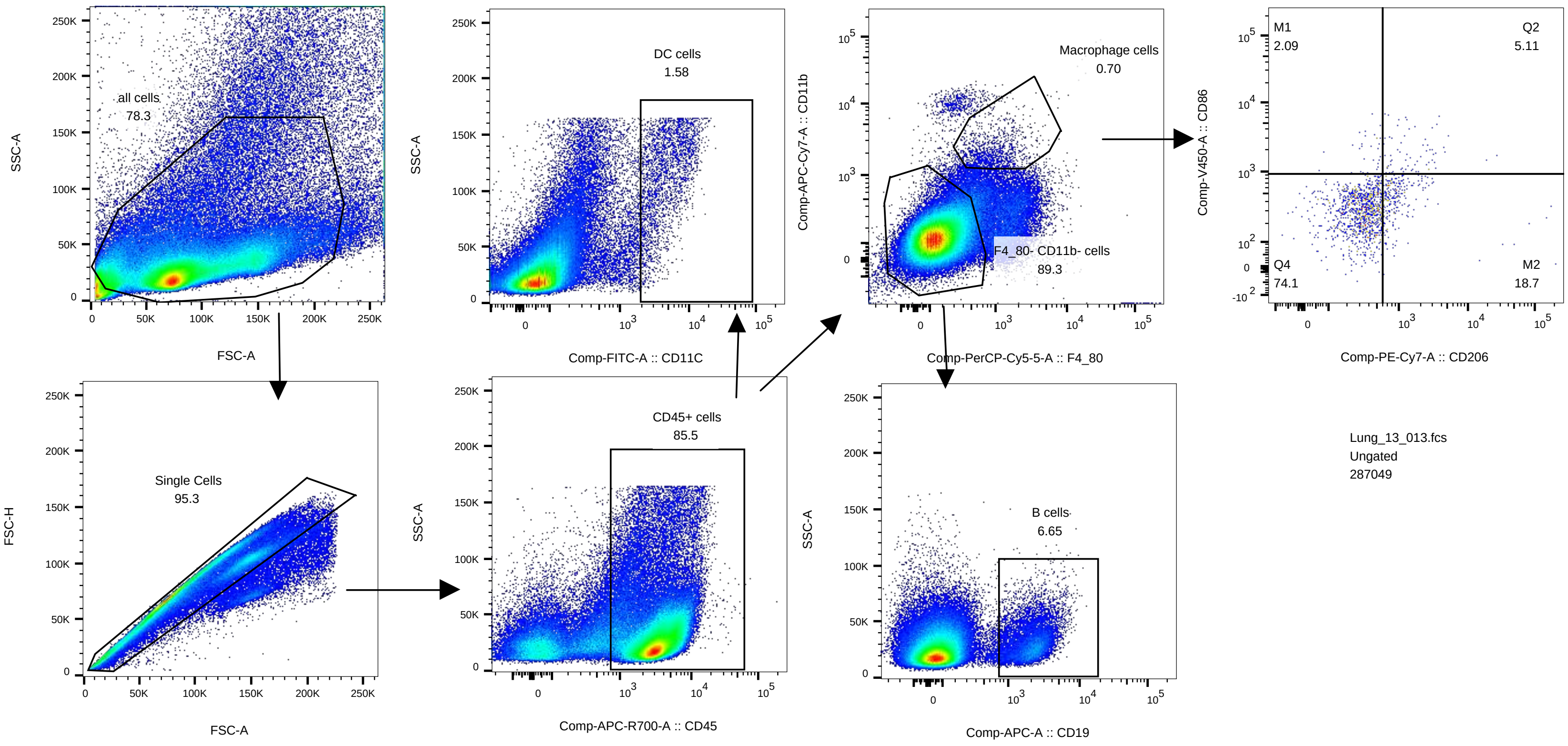
all cells
78.3
SSC-A
FSC-A
Single Cells
95.3
FSC-H
FSC-A
DC cells
1.58
SSC-A
Comp-FITC-A :: CD11C
CD45+ cells
85.5
SSC-A
Comp-APC-R700-A :: CD45
Macrophage cells
0.70
F4_80- CD11b- cells
89.3
Comp-APC-Cy7-A :: CD11b
Comp-PerCP-Cy5-5-A :: F4_80
B cells
6.65
SSC-A
Comp-APC-A :: CD19
M1
2.09
Q2
5.11
Q4
74.1
M2
18.7
Comp-V450-A :: CD86
Comp-PE-Cy7-A :: CD206
Lung_13_013.fcs
Ungated
287049

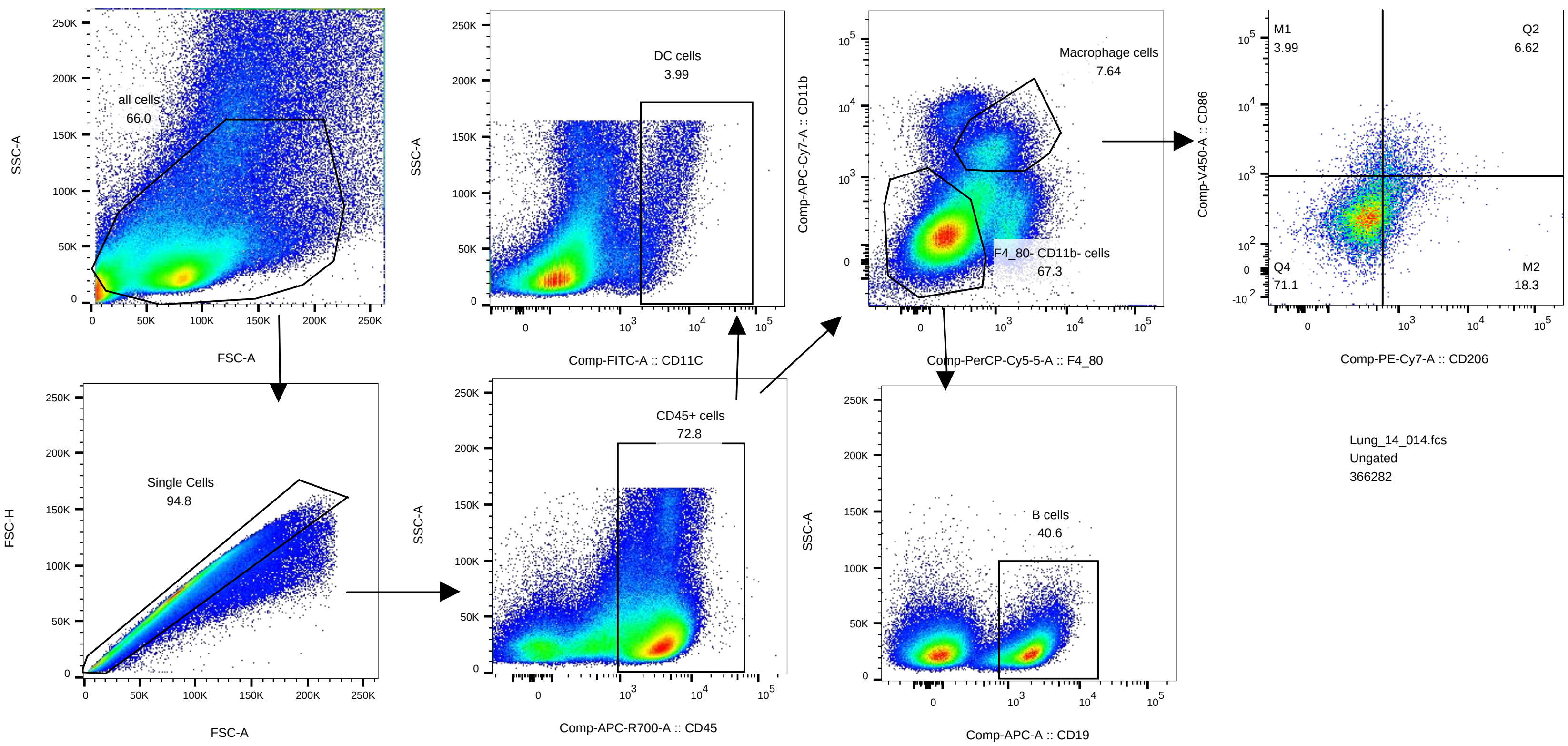

Lung_14_014.fcs
Ungated
366282

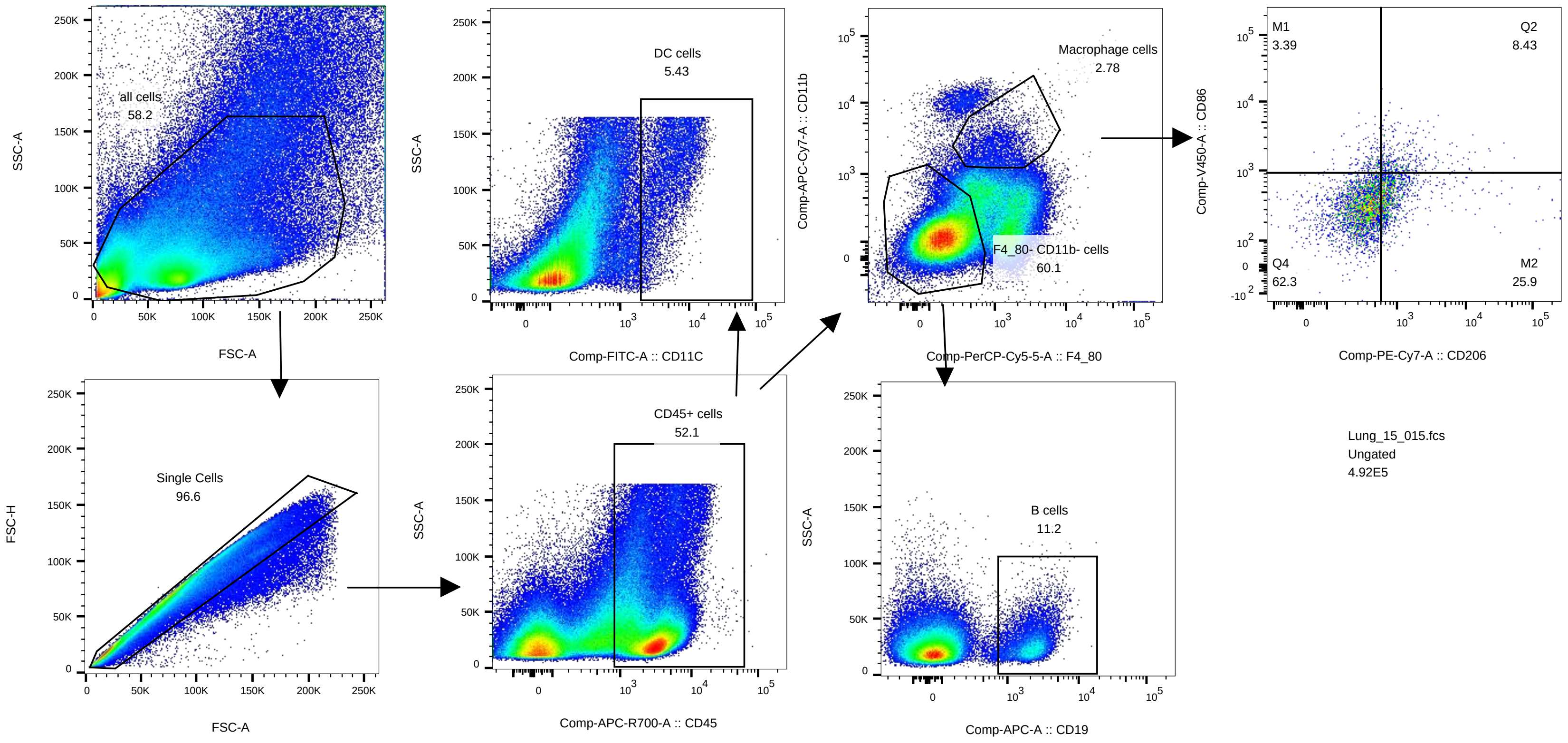
all cells
58.2
SSC-A
FSC-A
Single Cells
96.6
FSC-H
FSC-A
DC cells
5.43
SSC-A
Comp-FITC-A :: CD11C
CD45+ cells
52.1
SSC-A
Comp-APC-R700-A :: CD45
Macrophage cells
2.78
F4_80- CD11b- cells
60.1
Comp-APC-Cy7-A :: CD11b
Comp-PerCP-Cy5-5-A :: F4_80
B cells
11.2
SSC-A
Comp-APC-A :: CD19
M1
3.39
Q2
8.43
Q4
62.3
M2
25.9
Comp-V450-A :: CD86
Comp-PE-Cy7-A :: CD206
Lung_15_015.fcs
Ungated
4.92E5

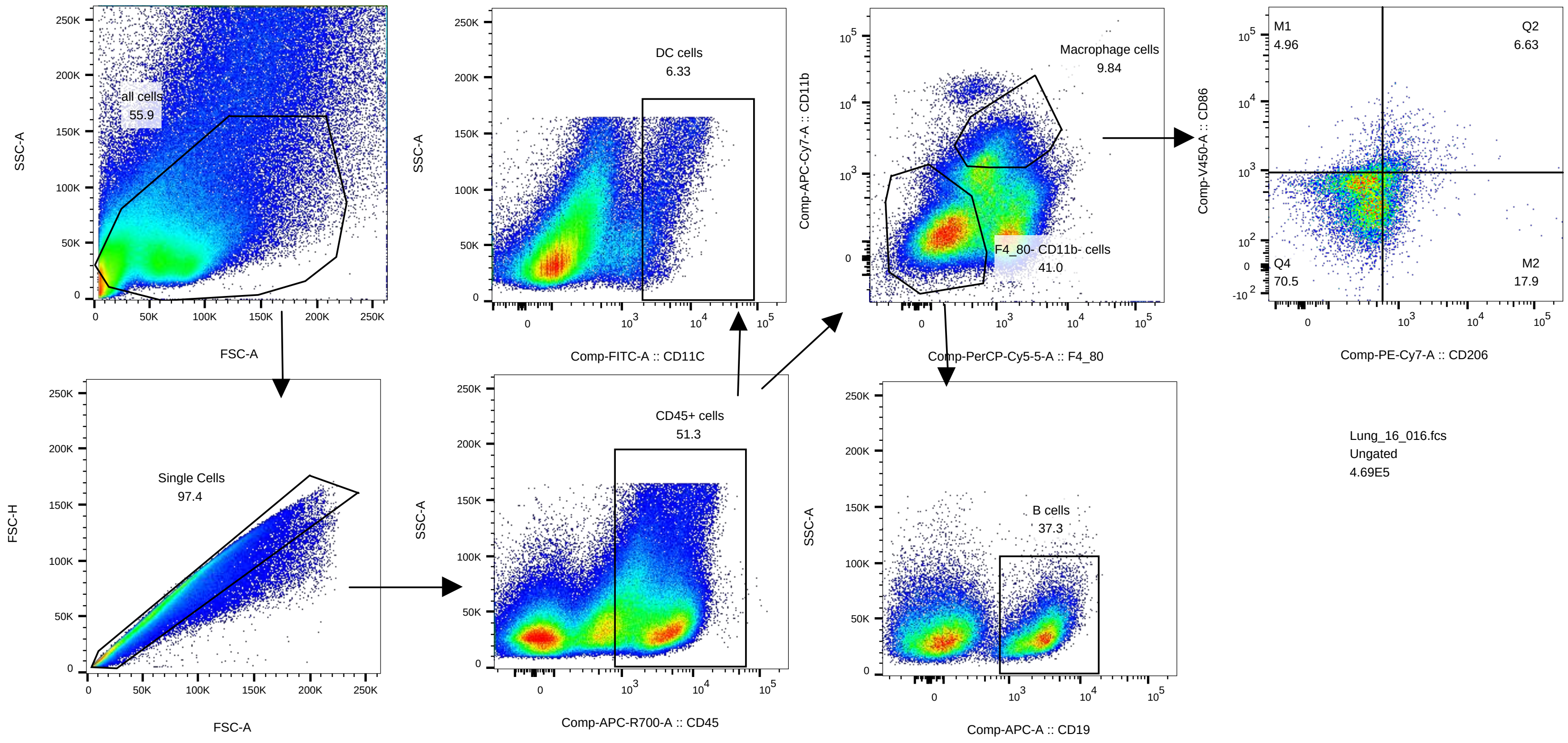
all cells
55.9
SSC-A
FSC-A
Single Cells
97.4
FSC-H
DC cells
6.33
Comp-FITC-A :: CD11C
CD45+ cells
51.3
Comp-APC-R700-A :: CD45
Macrophage cells
9.84
F4_80- CD11b- cells
41.0
Comp-APC-Cy7-A :: CD11b
Comp-PerCP-Cy5-5-A :: F4_80
B cells
37.3
Comp-APC-A :: CD19
M1
4.96
Q2
6.63
Q4
70.5
M2
17.9
Comp-V450-A :: CD86
Comp-PE-Cy7-A :: CD206
Lung_16_016.fcs
Ungated
4.69E5

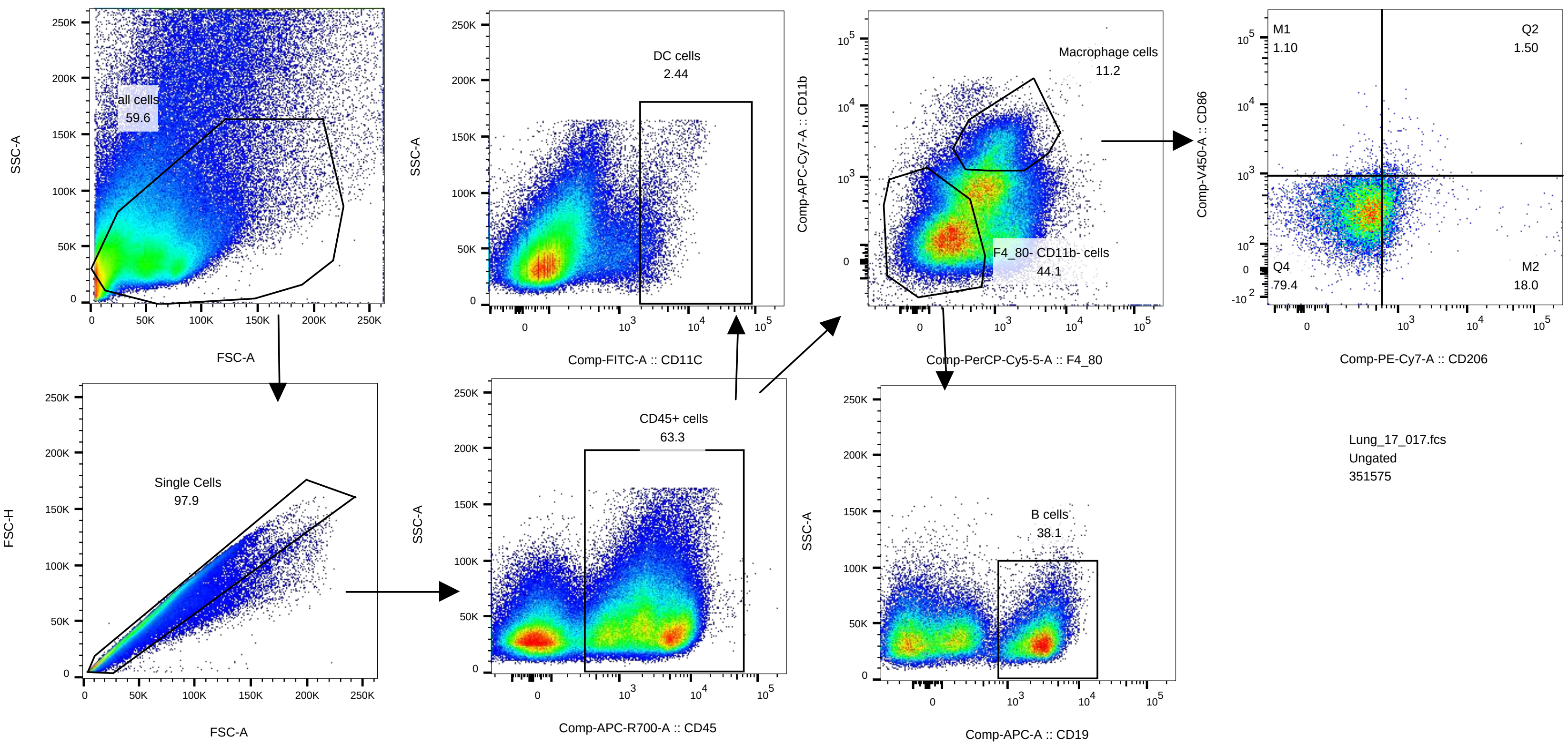

all cells
59.6
SSC-A
FSC-A
Single Cells
97.9
FSC-H
DC cells
2.44
Comp-FITC-A :: CD11C
CD45+ cells
63.3
Comp-APC-R700-A :: CD45
Macrophage cells
11.2
F4_80- CD11b- cells
44.1
Comp-APC-Cy7-A :: CD11b
Comp-PerCP-Cy5-5-A :: F4_80
B cells
38.1
Comp-APC-A :: CD19
M1
1.10
Q2
1.50
Q4
79.4
M2
18.0
Comp-V450-A :: CD86
Comp-PE-Cy7-A :: CD206
Lung_17_017.fcs
Ungated
351575

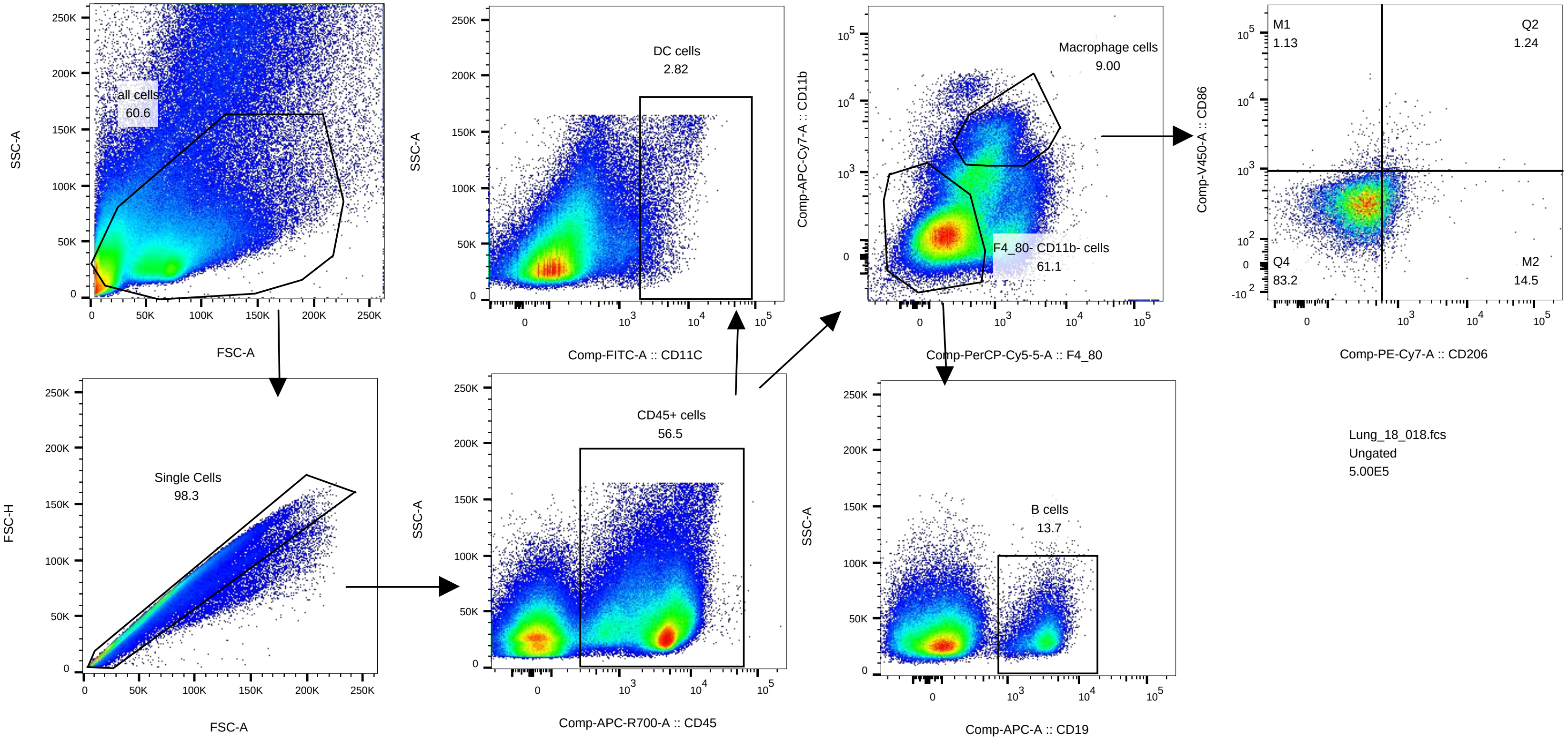

all cells
60.6
SSC-A
FSC-A
Single Cells
98.3
FSC-H
FSC-A
DC cells
2.82
SSC-A
Comp-FITC-A :: CD11C
CD45+ cells
56.5
SSC-A
Comp-APC-R700-A :: CD45
Macrophage cells
9.00
F4_80- CD11b- cells
61.1
Comp-APC-Cy7-A :: CD11b
Comp-PerCP-Cy5-5-A :: F4_80
B cells
13.7
SSC-A
Comp-APC-A :: CD19
M1
1.13
Q2
1.24
Q4
83.2
M2
14.5
Comp-V450-A :: CD86
Comp-PE-Cy7-A :: CD206
Lung_18_018.fcs
Ungated
5.00E5

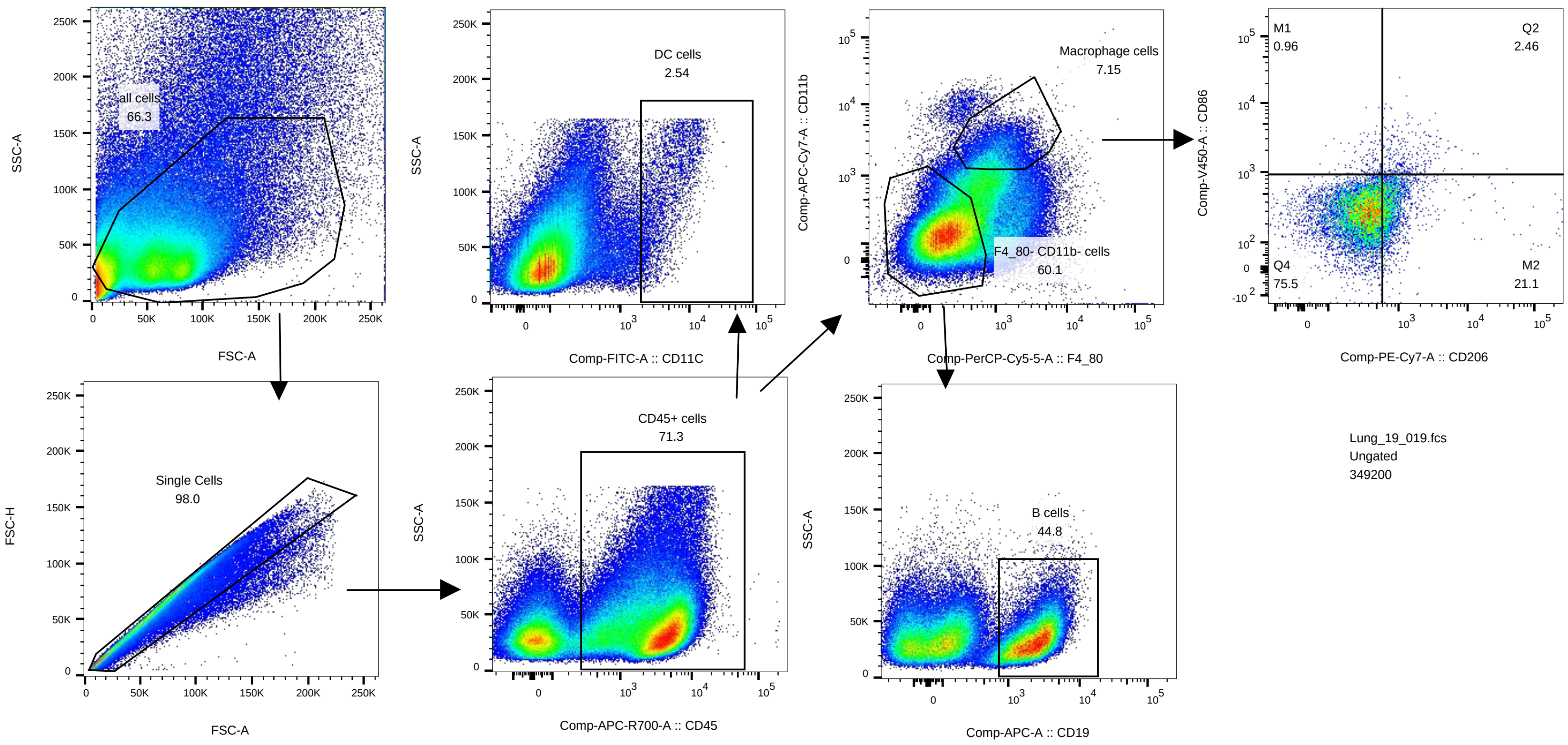

all cells
66.3
SSC-A
FSC-A
Single Cells
98.0
FSC-H
FSC-A
DC cells
2.54
SSC-A
Comp-FITC-A :: CD11C
CD45+ cells
71.3
SSC-A
Comp-APC-R700-A :: CD45
Macrophage cells
7.15
F4_80- CD11b- cells
60.1
Comp-APC-Cy7-A :: CD11b
Comp-PerCP-Cy5-5-A :: F4_80
B cells
44.8
SSC-A
Comp-APC-A :: CD19
M1
0.96
Q2
2.46
Q4
75.5
M2
21.1
Comp-V450-A :: CD86
Comp-PE-Cy7-A :: CD206
Lung_19_019.fcs
Ungated
349200

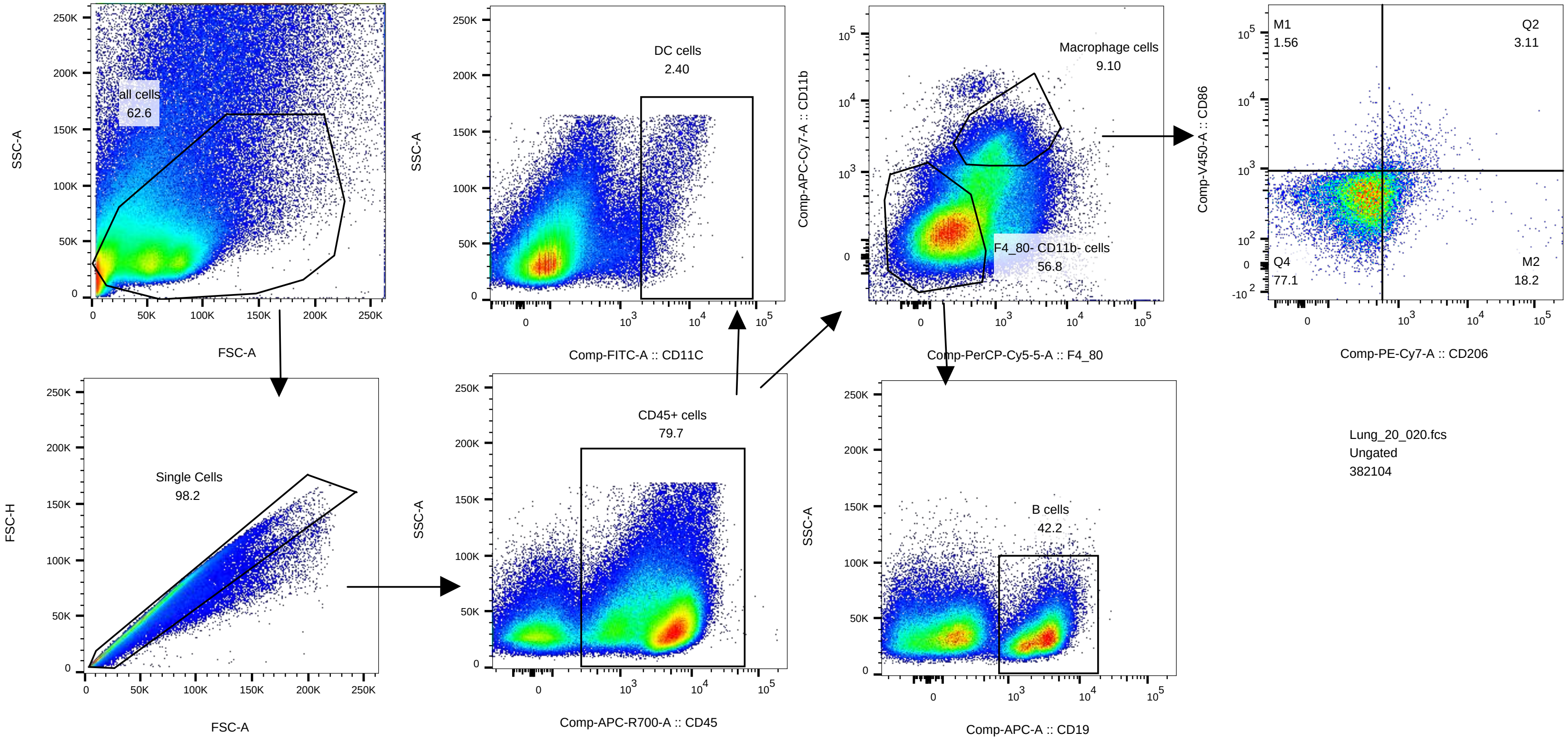
all cells
62.6
SSC-A
FSC-A
Single Cells
98.2
FSC-H
FSC-A
DC cells
2.40
SSC-A
Comp-FITC-A :: CD11C
CD45+ cells
79.7
SSC-A
Comp-APC-R700-A :: CD45
Macrophage cells
9.10
F4_80- CD11b- cells
56.8
Comp-APC-Cy7-A :: CD11b
Comp-PerCP-Cy5-5-A :: F4_80
B cells
42.2
SSC-A
Comp-APC-A :: CD19
M1
1.56
Q2
3.11
Q4
77.1
M2
18.2
Comp-V450-A :: CD86
Comp-PE-Cy7-A :: CD206
Lung_20_020.fcs
Ungated
382104

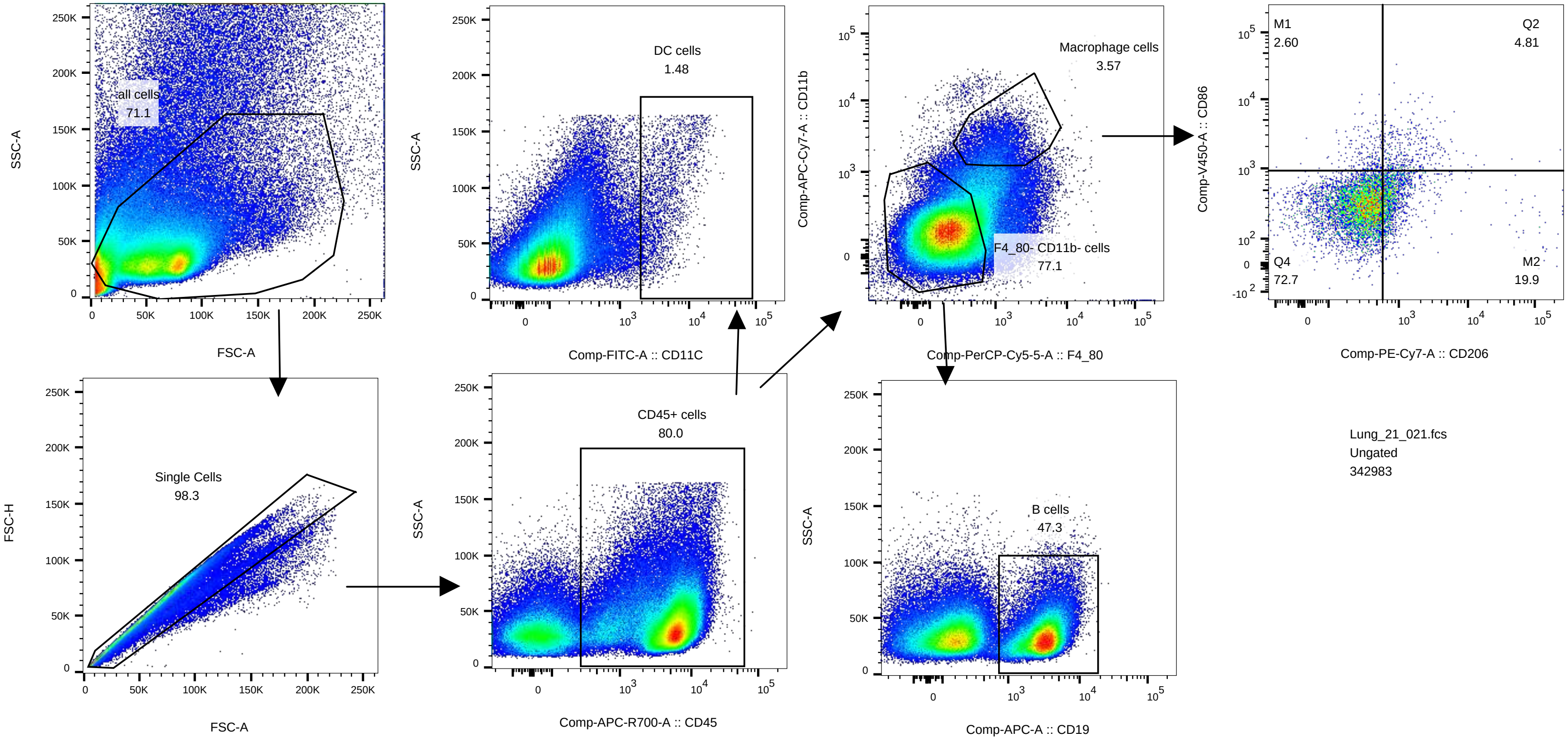

SSC-A
FSC-A
all cells
71.1
FSC-H
Single Cells
98.3
DC cells
1.48
Comp-FITC-A :: CD11C
CD45+ cells
80.0
Comp-APC-R700-A :: CD45
Comp-APC-Cy7-A :: CD11b
Macrophage cells
3.57
F4_80- CD11b- cells
77.1
Comp-PerCP-Cy5-5-A :: F4_80
B cells
47.3
Comp-APC-A :: CD19
Comp-V450-A :: CD86
M1
2.60
Q2
4.81
Q4
72.7
M2
19.9
Comp-PE-Cy7-A :: CD206
Lung_21_021.fcs
Ungated
342983

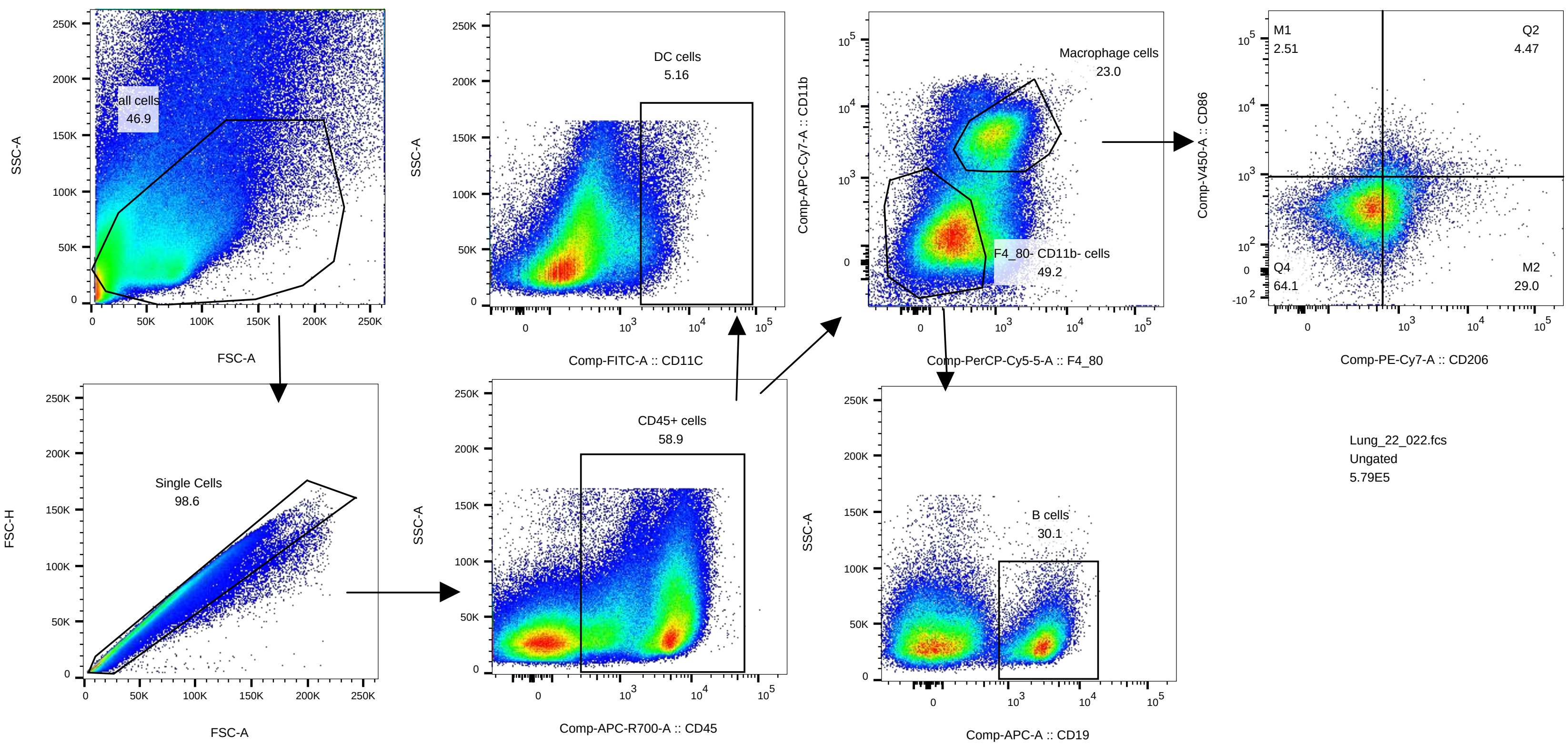

all cells
46.9
FSC-A
SSC-A
Single Cells
98.6
FSC-H
DC cells
5.16
Comp-FITC-A :: CD11C
CD45+ cells
58.9
Comp-APC-R700-A :: CD45
Macrophage cells
23.0
F4_80- CD11b- cells
49.2
Comp-APC-Cy7-A :: CD11b
Comp-PerCP-Cy5-5-A :: F4_80
B cells
30.1
Comp-APC-A :: CD19
M1
2.51
Q2
4.47
Q4
64.1
M2
29.0
Comp-V450-A :: CD86
Comp-PE-Cy7-A :: CD206
Lung_22_022.fcs
Ungated
5.79E5

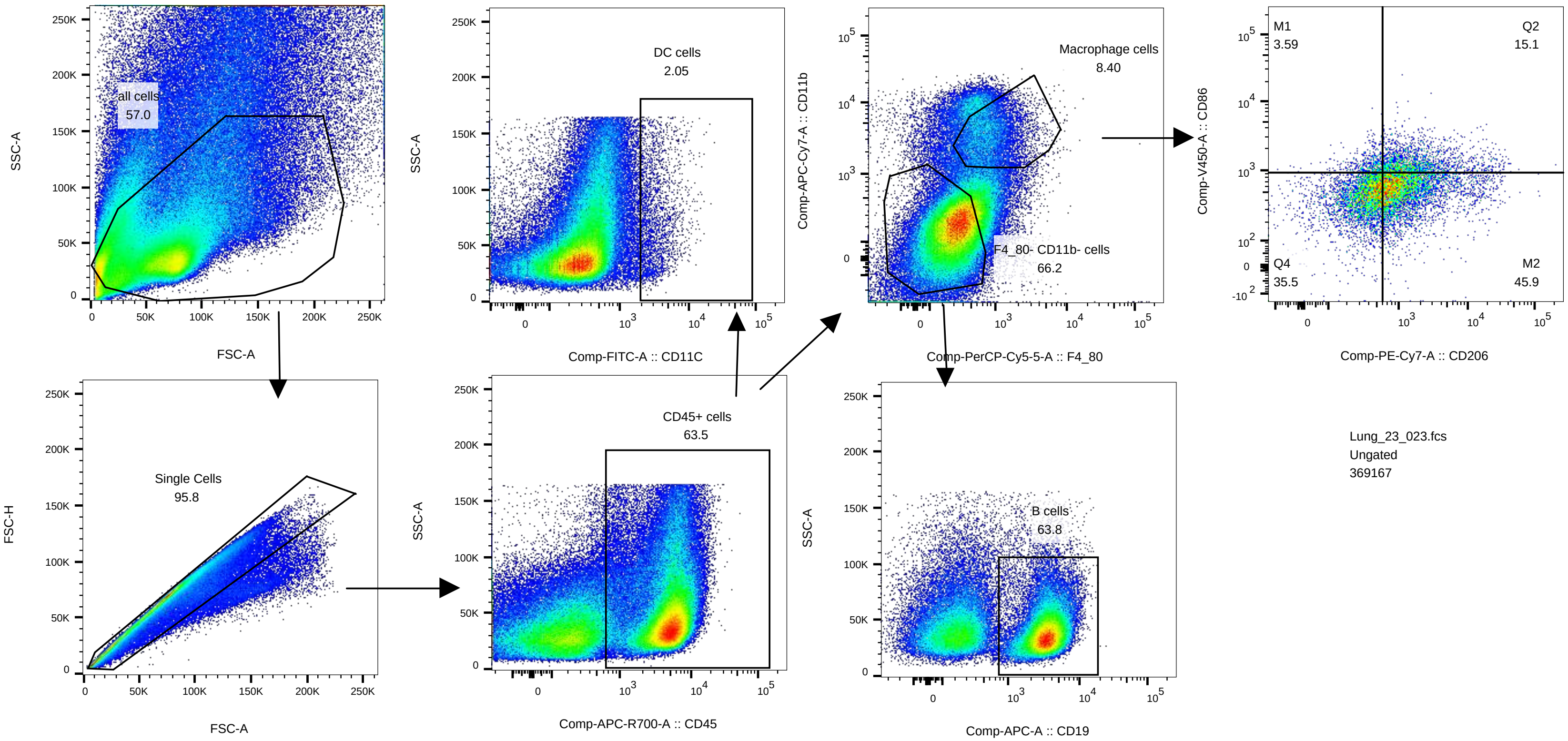
all cells
57.0
SSC-A
FSC-A
Single Cells
95.8
FSC-H
FSC-A
DC cells
2.05
SSC-A
Comp-FITC-A :: CD11C
CD45+ cells
63.5
SSC-A
Comp-APC-R700-A :: CD45
Macrophage cells
8.40
F4_80- CD11b- cells
66.2
Comp-APC-Cy7-A :: CD11b
Comp-PerCP-Cy5-5-A :: F4_80
B cells
63.8
SSC-A
Comp-APC-A :: CD19
M1
3.59
Q2
15.1
Q4
35.5
M2
45.9
Comp-V450-A :: CD86
Comp-PE-Cy7-A :: CD206
Lung_23_023.fcs
Ungated
369167

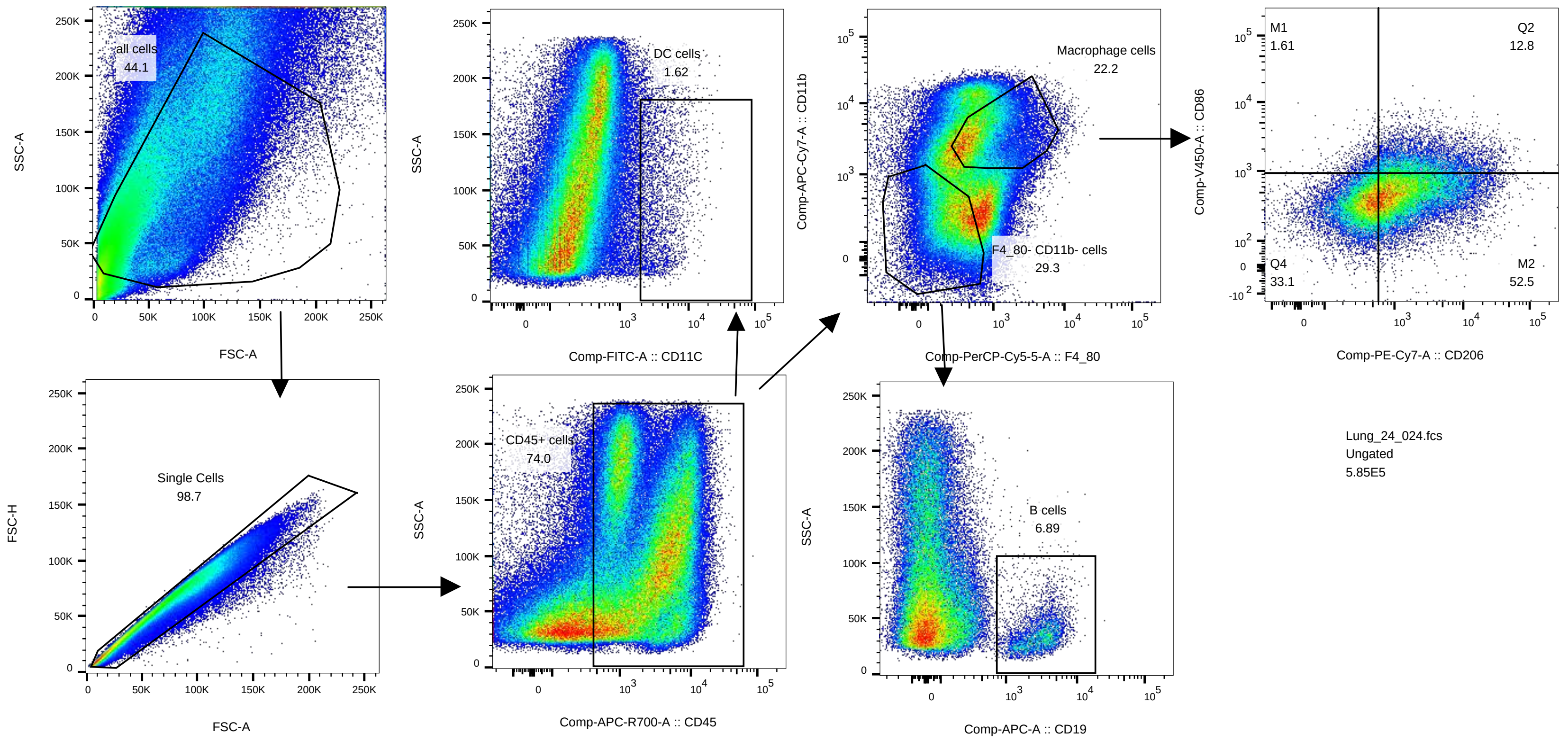
all cells
44.1
SSC-A
FSC-A
Single Cells
98.7
FSC-H
DC cells
1.62
Comp-FITC-A :: CD11C
CD45+ cells
74.0
Comp-APC-R700-A :: CD45
Macrophage cells
22.2
F4_80- CD11b- cells
29.3
Comp-APC-Cy7-A :: CD11b
Comp-PerCP-Cy5-5-A :: F4_80
B cells
6.89
Comp-APC-A :: CD19
M1
1.61
Q2
12.8
Q4
33.1
M2
52.5
Comp-V450-A :: CD86
Comp-PE-Cy7-A :: CD206
Lung_24_024.fcs
Ungated
5.85E5

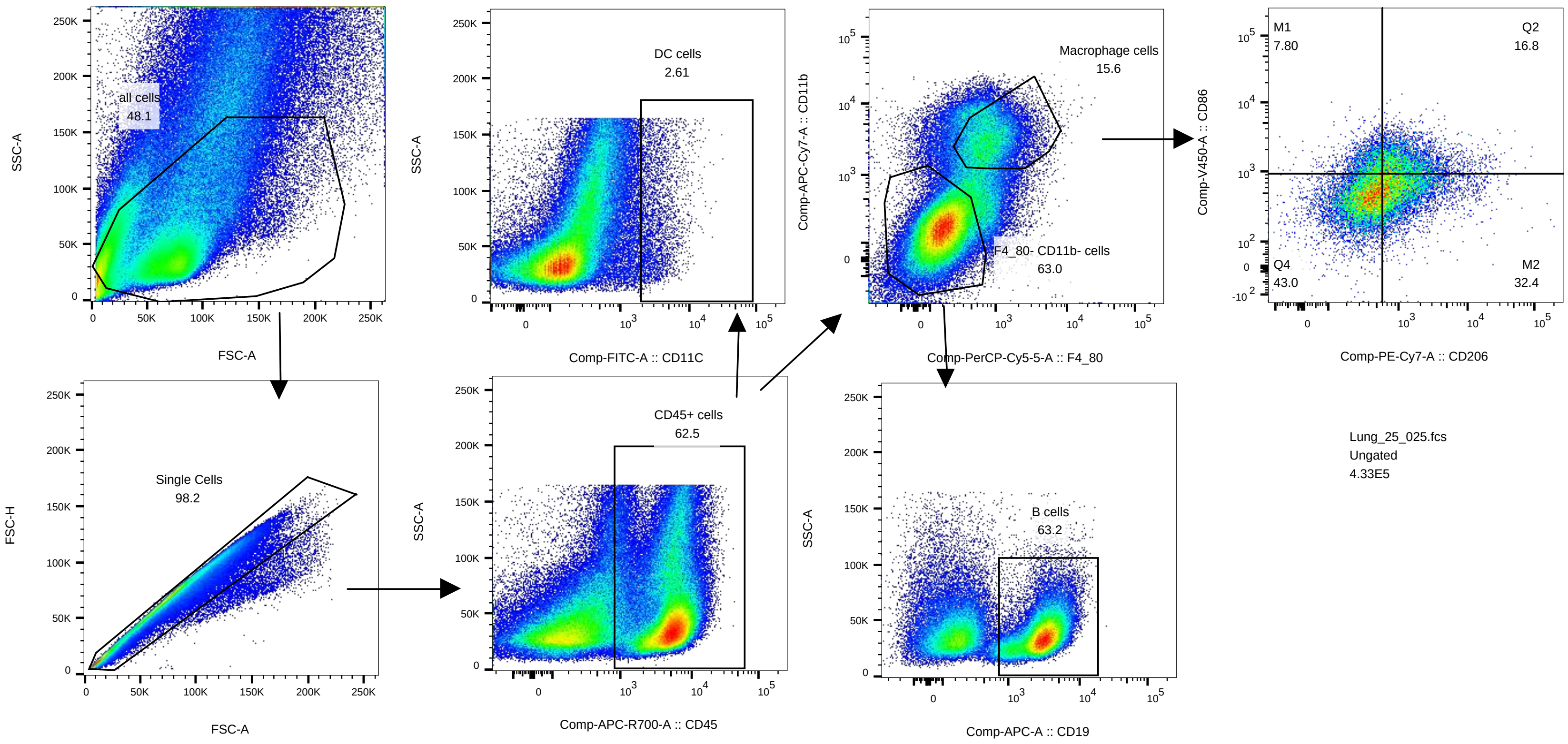
all cells
48.1
SSC-A
FSC-A
Single Cells
98.2
FSC-H
FSC-A
DC cells
2.61
SSC-A
Comp-FITC-A :: CD11C
CD45+ cells
62.5
SSC-A
Comp-APC-R700-A :: CD45
Macrophage cells
15.6
F4_80- CD11b- cells
63.0
Comp-APC-Cy7-A :: CD11b
Comp-PerCP-Cy5-5-A :: F4_80
B cells
63.2
SSC-A
Comp-APC-A :: CD19
M1
7.80
Q2
16.8
Q4
43.0
M2
32.4
Comp-V450-A :: CD86
Comp-PE-Cy7-A :: CD206
Lung_25_025.fcs
Ungated
4.33E5
