# Supplementary material for: A novel HVEM-Fc recombinant protein for lung cancer immunotherapy
Source: J Exp Clin Cancer Res. 2025 Feb 20;44:62. doi: 10.1186/s13046-025-03324-8 (PMC11841141; doi:10.1186/s13046-025-03324-8)
Supplement: Supplementary file 5 — Flow cytometry gating strategies for the identification of immune cell subsets. [file 13046_2025_3324_MOESM5_ESM.zip › Supplement File7.pdf]

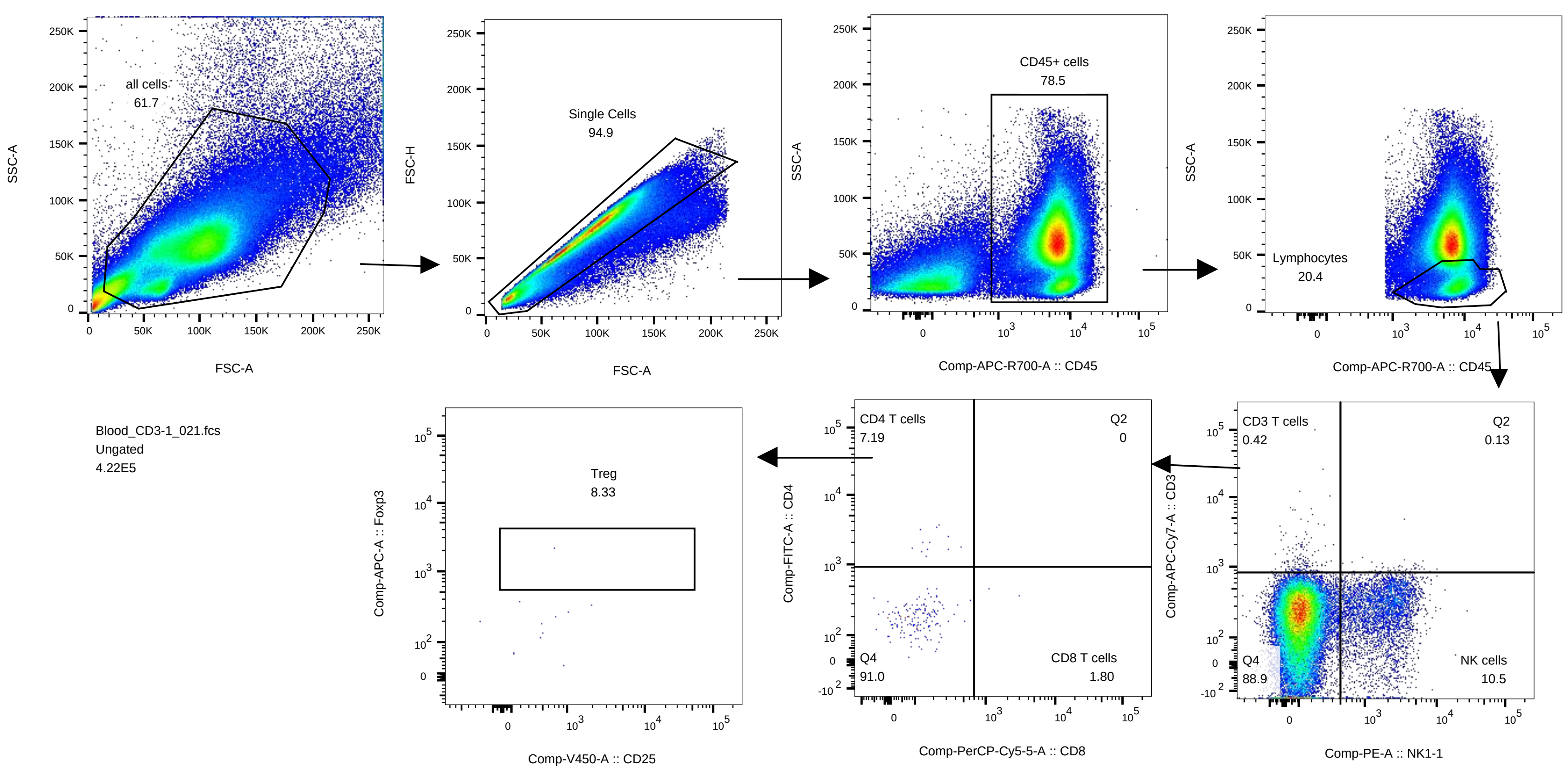
all cells
61.7
SSC-A
FSC-A
Single Cells
94.9
FSC-H
FSC-A
CD45+ cells
78.5
SSC-A
Comp-APC-R700-A :: CD45
Lymphocytes
20.4
SSC-A
Comp-APC-R700-A :: CD45
Blood_CD3-1_021.fcs
Ungated
4.22E5
Treg
8.33
Comp-APC-A :: Foxp3
Comp-V450-A :: CD25
CD4 T cells
7.19
Q2
0
Q4
91.0
CD8 T cells
1.80
Comp-FITC-A :: CD4
Comp-PerCP-Cy5-5-A :: CD8
CD3 T cells
0.42
Q2
0.13
Q4
88.9
NK cells
10.5
Comp-APC-Cy7-A :: CD3
Comp-PE-A :: NK1-1

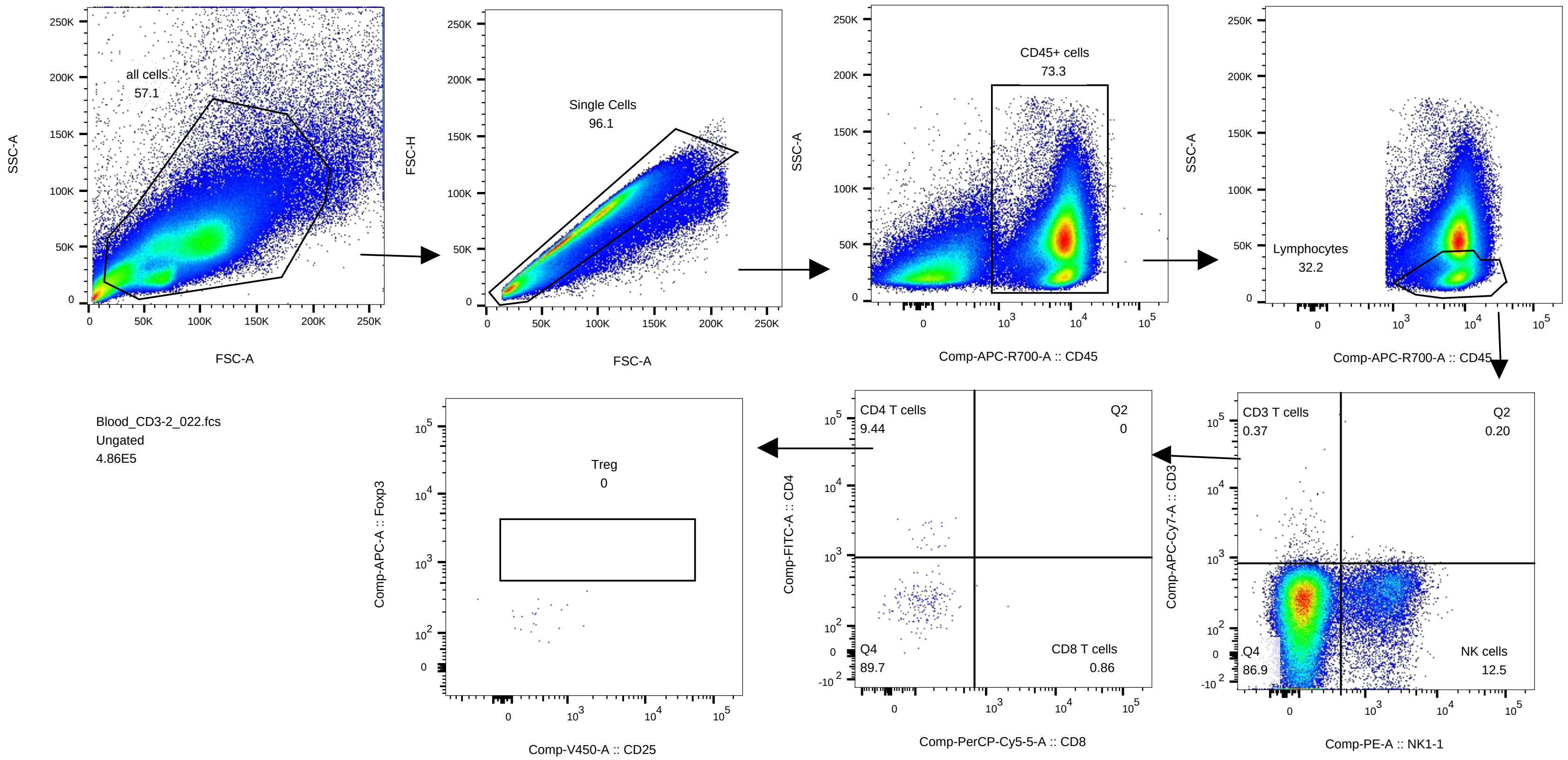

all cells
57.1
SSC-A
FSC-A
Single Cells
96.1
FSC-H
CD45+ cells
73.3
Comp-APC-R700-A :: CD45
Lymphocytes
32.2
CD3 T cells
0.37
Q2
0.20
Q4
86.9
NK cells
12.5
Comp-APC-Cy7-A :: CD3
Comp-PE-A :: NK1-1
CD4 T cells
9.44
Q2
0
Q4
89.7
CD8 T cells
0.86
Comp-FITC-A :: CD4
Comp-PerCP-Cy5-5-A :: CD8
Treg
0
Comp-APC-A :: Foxp3
Comp-V450-A :: CD25
Blood_CD3-2_022.fcs
Ungated
4.86E5

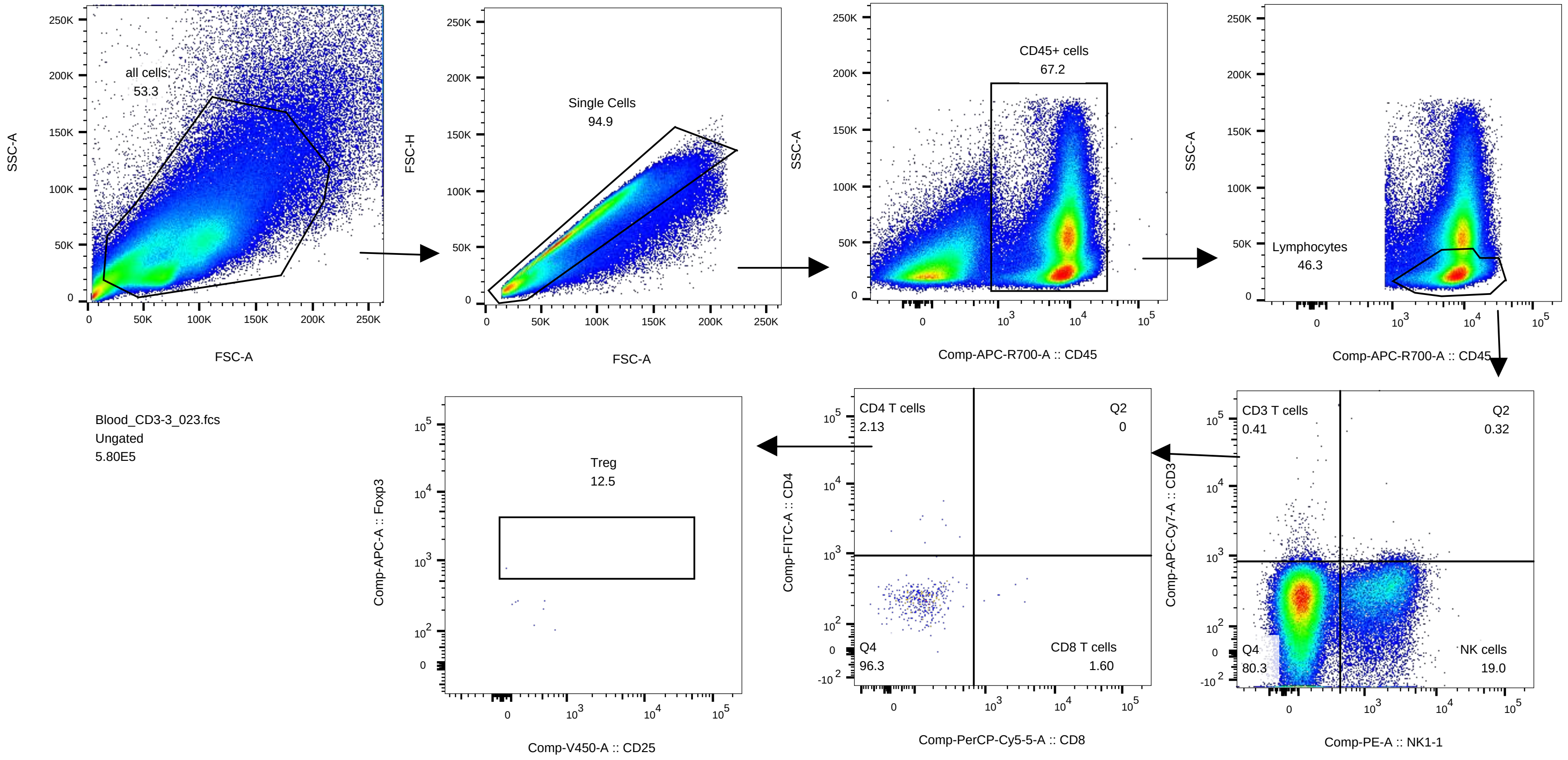
all cells
53.3
SSC-A
FSC-A
Blood_CD3-3_023.fcs
Ungated
5.80E5
Single Cells
94.9
FSC-H
FSC-A
CD45+ cells
67.2
SSC-A
Comp-APC-R700-A :: CD45
Lymphocytes
46.3
SSC-A
Comp-APC-R700-A :: CD45
CD3 T cells
0.41
Q2
0.32
Q4
80.3
NK cells
19.0
Comp-APC-Cy7-A :: CD3
Comp-PE-A :: NK1-1
CD4 T cells
2.13
Q2
0
Q4
96.3
CD8 T cells
1.60
Comp-FITC-A :: CD4
Comp-PerCP-Cy5-5-A :: CD8
Treg
12.5
Comp-APC-A :: Foxp3
Comp-V450-A :: CD25

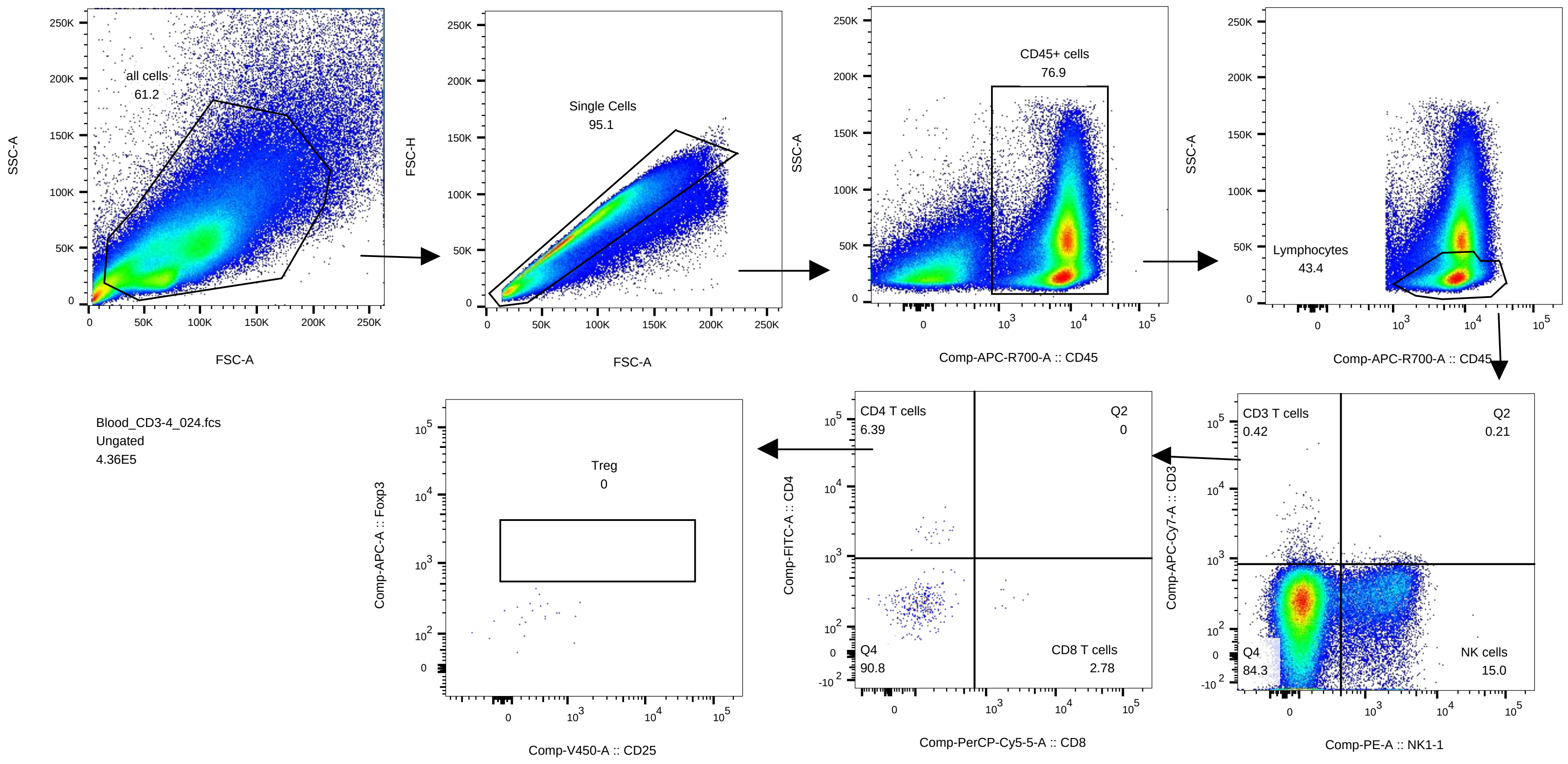

all cells
61.2
SSC-A
FSC-A
Single Cells
95.1
FSC-H
FSC-A
CD45+ cells
76.9
SSC-A
Comp-APC-R700-A :: CD45
Lymphocytes
43.4
SSC-A
Comp-APC-R700-A :: CD45
Blood_CD3-4_024.fcs
Ungated
4.36E5
Treg
0
Comp-APC-A :: Foxp3
Comp-V450-A :: CD25
CD4 T cells
6.39
Q2
0
Q4
90.8
CD8 T cells
2.78
Comp-FITC-A :: CD4
Comp-PerCP-Cy5-5-A :: CD8
CD3 T cells
0.42
Q2
0.21
Q4
84.3
NK cells
15.0
Comp-APC-Cy7-A :: CD3
Comp-PE-A :: NK1-1

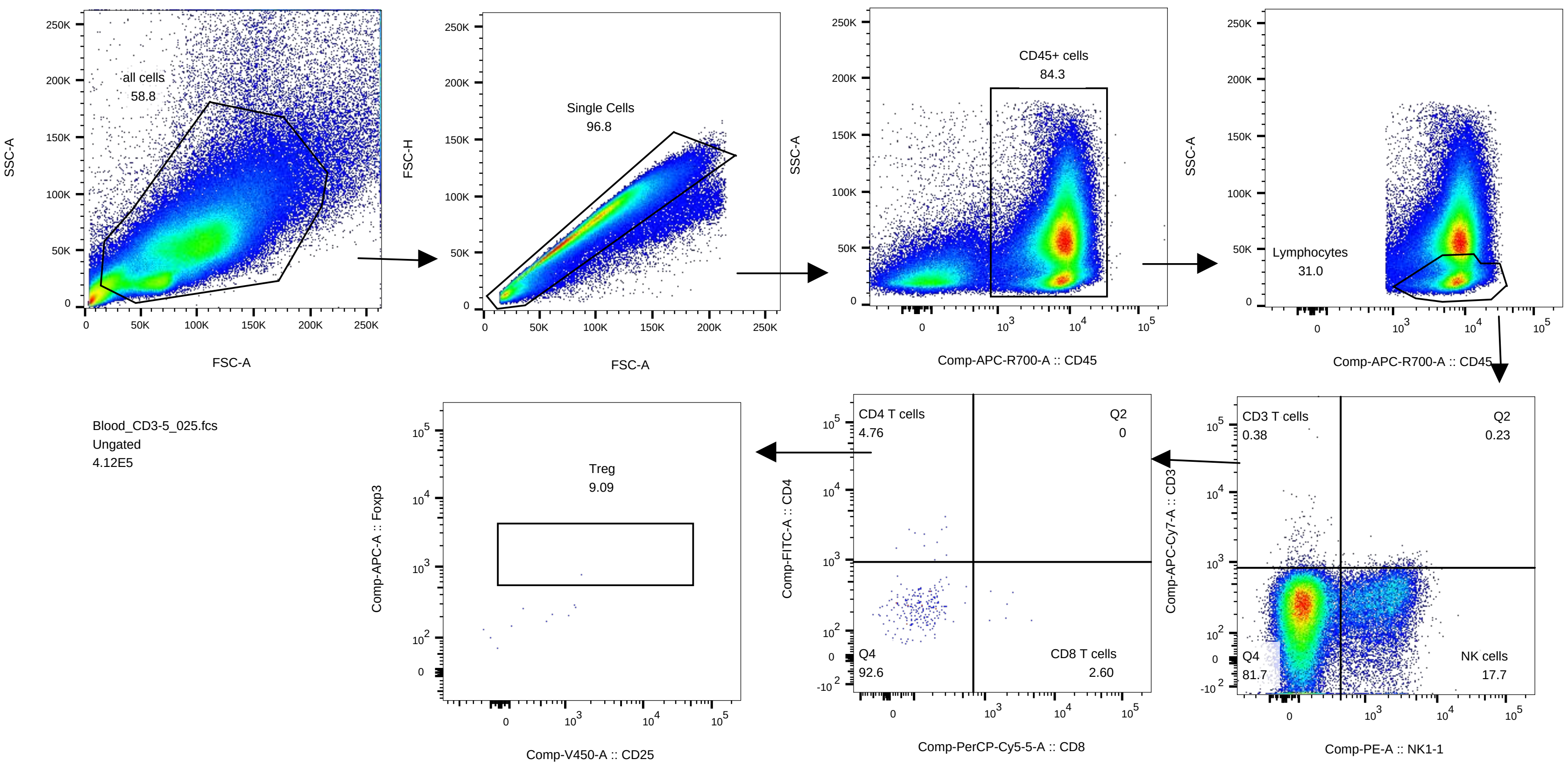

all cells
58.8
SSC-A
FSC-A
Single Cells
96.8
FSC-H
FSC-A
CD45+ cells
84.3
SSC-A
Comp-APC-R700-A :: CD45
Lymphocytes
31.0
SSC-A
Comp-APC-R700-A :: CD45
Blood_CD3-5_025.fcs
Ungated
4.12E5
CD3 T cells
0.38
Q2
0.23
Q4
81.7
NK cells
17.7
Comp-APC-Cy7-A :: CD3
Comp-PE-A :: NK1-1
CD4 T cells
4.76
Q2
0
Q4
92.6
CD8 T cells
2.60
Comp-FITC-A :: CD4
Comp-PerCP-Cy5-5-A :: CD8
Treg
9.09
Comp-APC-A :: Foxp3
Comp-V450-A :: CD25

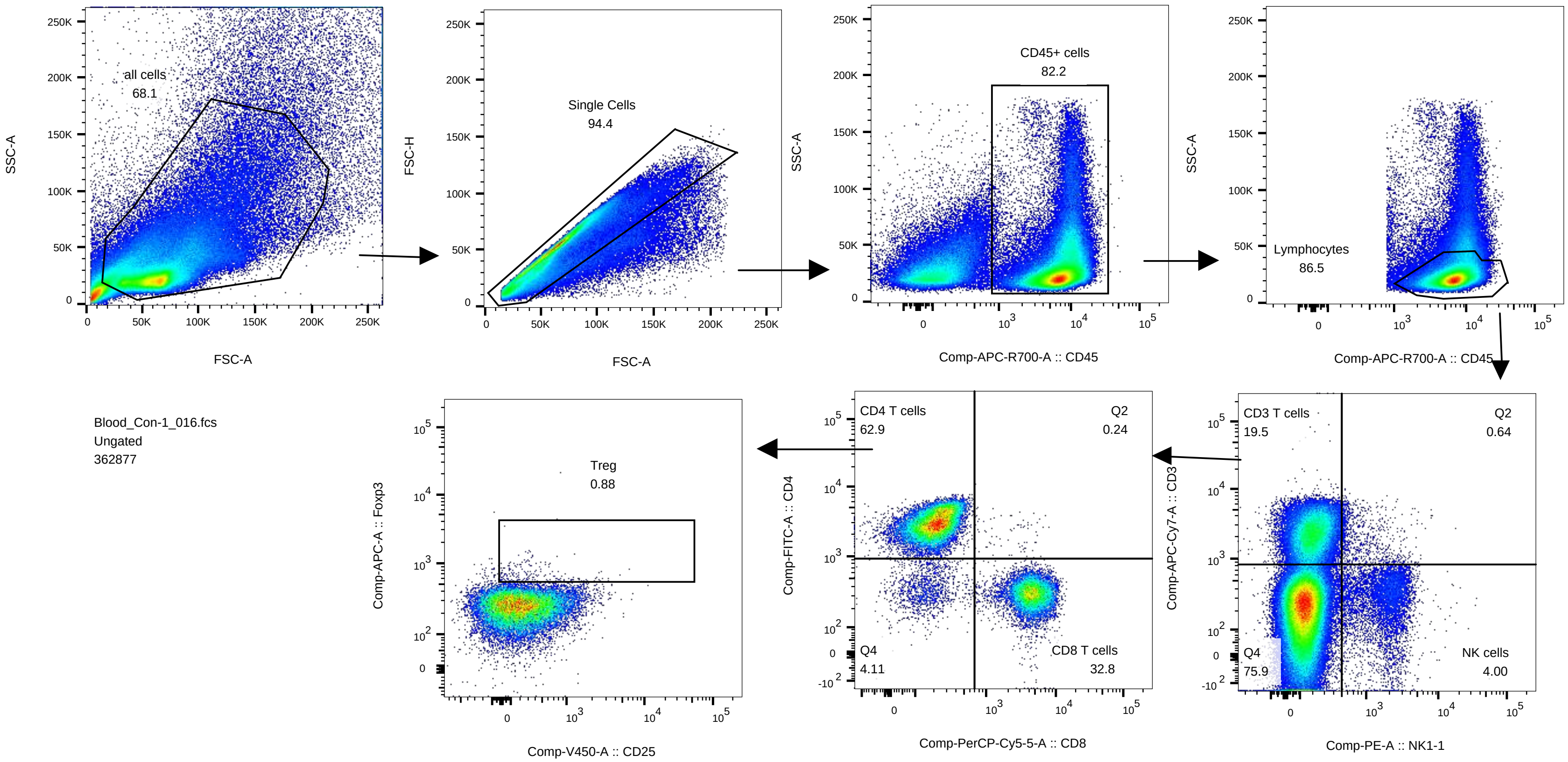
all cells
68.1
SSC-A
FSC-A
Single Cells
94.4
FSC-H
FSC-A
CD45+ cells
82.2
SSC-A
Comp-APC-R700-A :: CD45
Lymphocytes
86.5
SSC-A
Comp-APC-R700-A :: CD45
Blood_Con-1_016.fcs
Ungated
362877
Treg
0.88
Comp-APC-A :: Foxp3
Comp-V450-A :: CD25
CD4 T cells
62.9
Q2
0.24
Q4
4.11
CD8 T cells
32.8
Comp-FITC-A :: CD4
Comp-PerCP-Cy5-5-A :: CD8
CD3 T cells
19.5
Q2
0.64
Q4
75.9
NK cells
4.00
Comp-APC-Cy7-A :: CD3
Comp-PE-A :: NK1-1

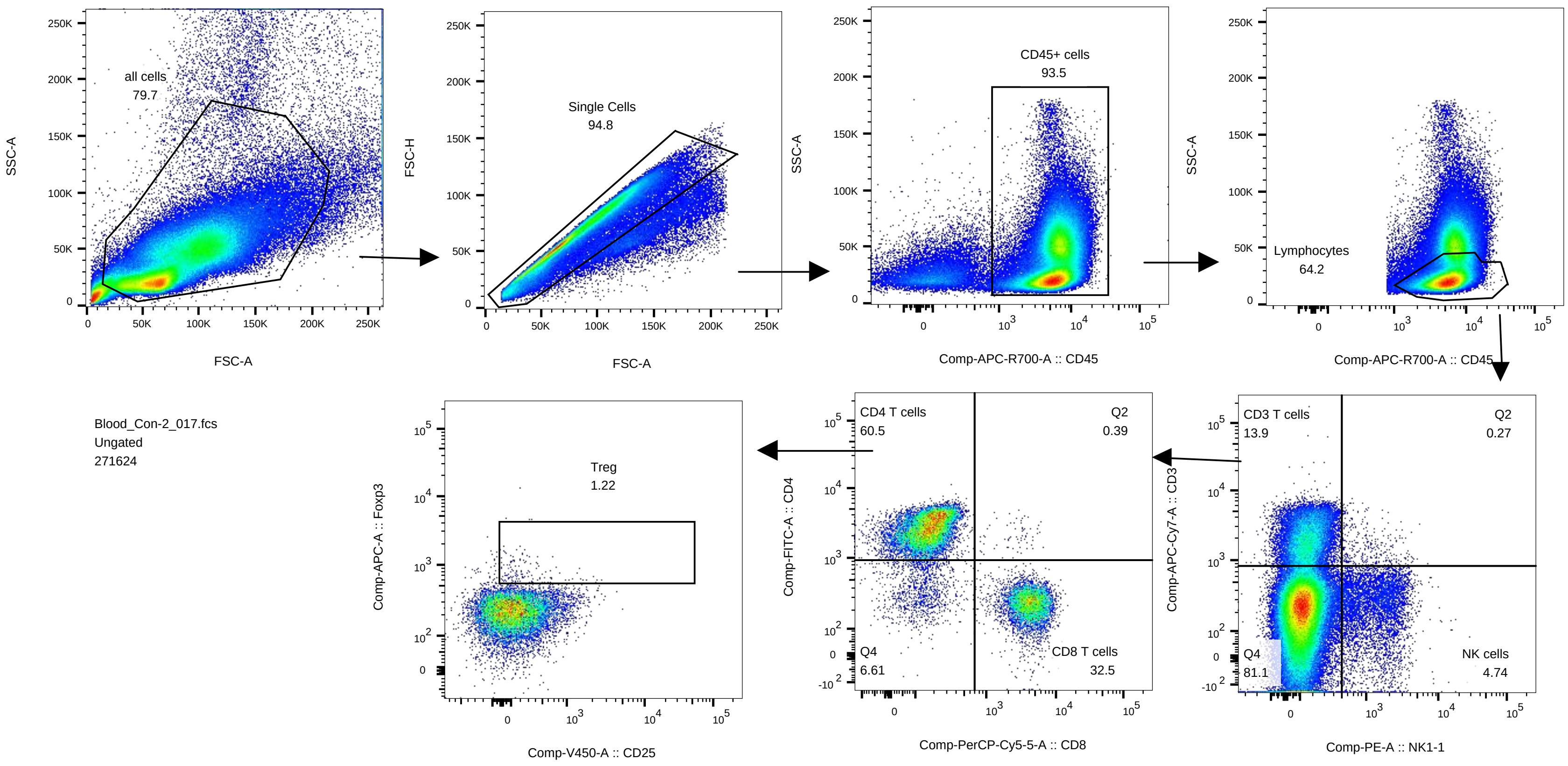

all cells
79.7
SSC-A
FSC-A
Single Cells
94.8
FSC-H
FSC-A
CD45+ cells
93.5
SSC-A
Comp-APC-R700-A :: CD45
Lymphocytes
64.2
SSC-A
Comp-APC-R700-A :: CD45
Blood_Con-2_017.fcs
Ungated
271624
CD3 T cells
13.9
Q2
0.27
Q4
81.1
NK cells
4.74
Comp-APC-Cy7-A :: CD3
Comp-PE-A :: NK1-1
CD4 T cells
60.5
Q2
0.39
Q4
6.61
CD8 T cells
32.5
Comp-FITC-A :: CD4
Comp-PerCP-Cy5-5-A :: CD8
Treg
1.22
Comp-APC-A :: Foxp3
Comp-V450-A :: CD25

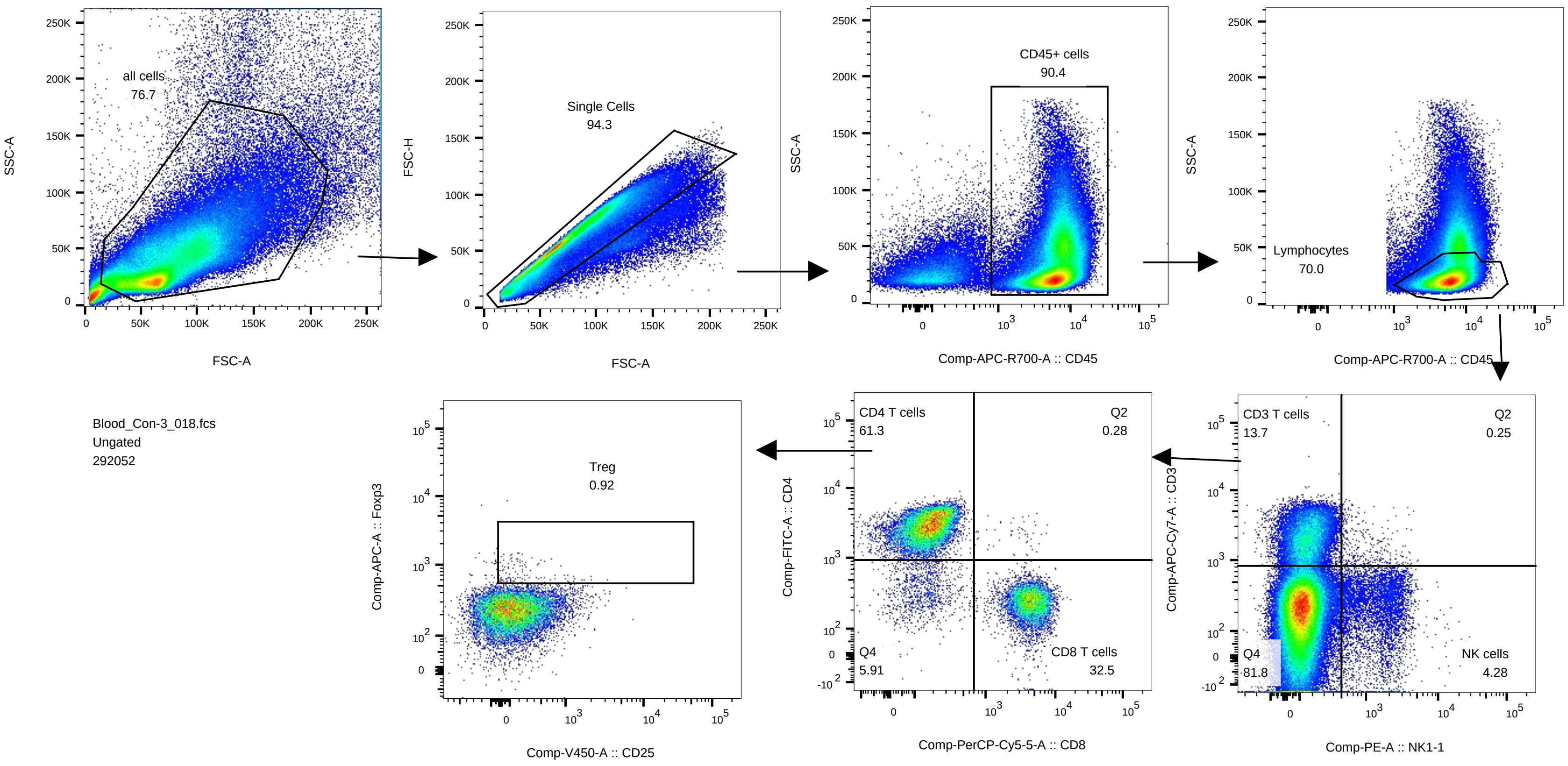
all cells
76.7
SSC-A
FSC-A
Single Cells
94.3
FSC-H
FSC-A
CD45+ cells
90.4
SSC-A
Comp-APC-R700-A :: CD45
Lymphocytes
70.0
SSC-A
Comp-APC-R700-A :: CD45
Blood_Con-3_018.fcs
Ungated
292052
Treg
0.92
Comp-APC-A :: Foxp3
Comp-V450-A :: CD25
CD4 T cells
61.3
Q2
0.28
Q4
5.91
CD8 T cells
32.5
Comp-FITC-A :: CD4
Comp-PerCP-Cy5-5-A :: CD8
CD3 T cells
13.7
Q2
0.25
Q4
81.8
NK cells
4.28
Comp-APC-Cy7-A :: CD3
Comp-PE-A :: NK1-1

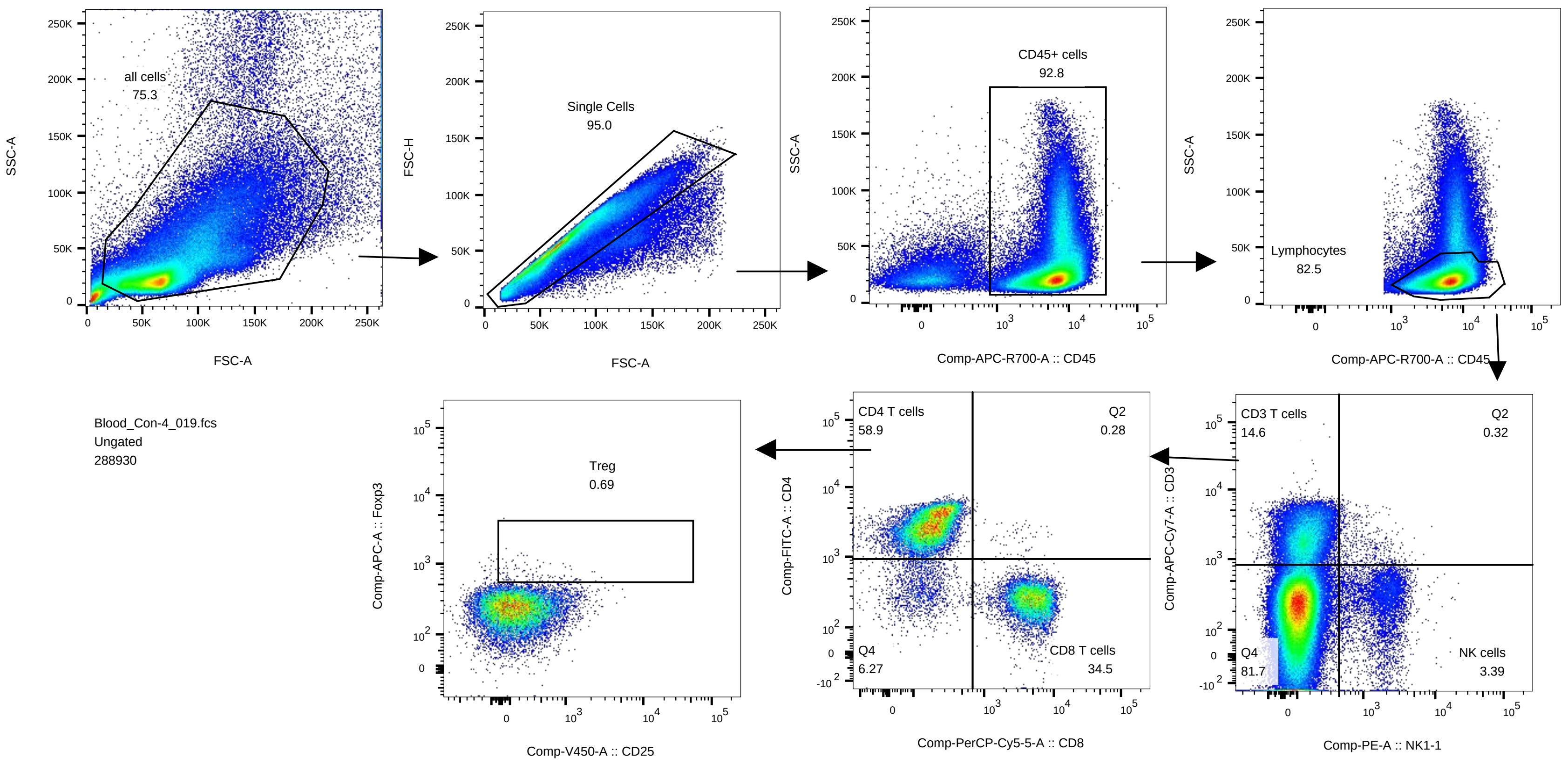
all cells
75.3
SSC-A
FSC-A
Single Cells
95.0
FSC-H
FSC-A
CD45+ cells
92.8
SSC-A
Comp-APC-R700-A :: CD45
Lymphocytes
82.5
SSC-A
Comp-APC-R700-A :: CD45
Blood_Con-4_019.fcs
Ungated
288930
Treg
0.69
Comp-APC-A :: Foxp3
Comp-V450-A :: CD25
CD4 T cells
58.9
Q2
0.28
Q4
6.27
CD8 T cells
34.5
Comp-FITC-A :: CD4
Comp-PerCP-Cy5-5-A :: CD8
CD3 T cells
14.6
Q2
0.32
Q4
81.7
NK cells
3.39
Comp-APC-Cy7-A :: CD3
Comp-PE-A :: NK1-1

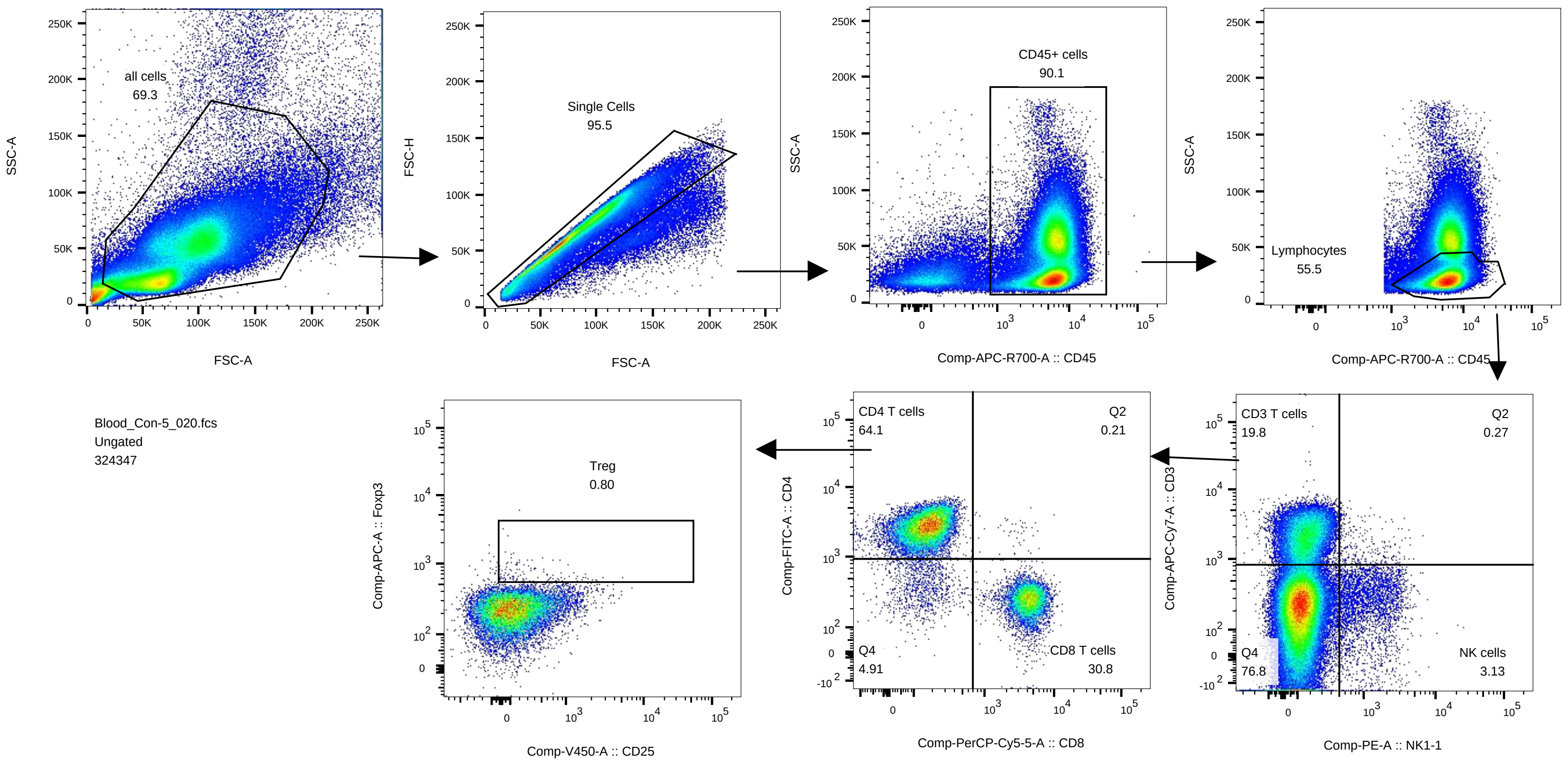
all cells
69.3
SSC-A
FSC-A
Single Cells
95.5
FSC-H
FSC-A
CD45+ cells
90.1
SSC-A
Comp-APC-R700-A :: CD45
Lymphocytes
55.5
SSC-A
Comp-APC-R700-A :: CD45
Blood_Con-5_020.fcs
Ungated
324347
Treg
0.80
Comp-APC-A :: Foxp3
Comp-V450-A :: CD25
CD4 T cells
64.1
Q2
0.21
Q4
4.91
CD8 T cells
30.8
Comp-FITC-A :: CD4
Comp-PerCP-Cy5-5-A :: CD8
CD3 T cells
19.8
Q2
0.27
Q4
76.8
NK cells
3.13
Comp-APC-Cy7-A :: CD3
Comp-PE-A :: NK1-1

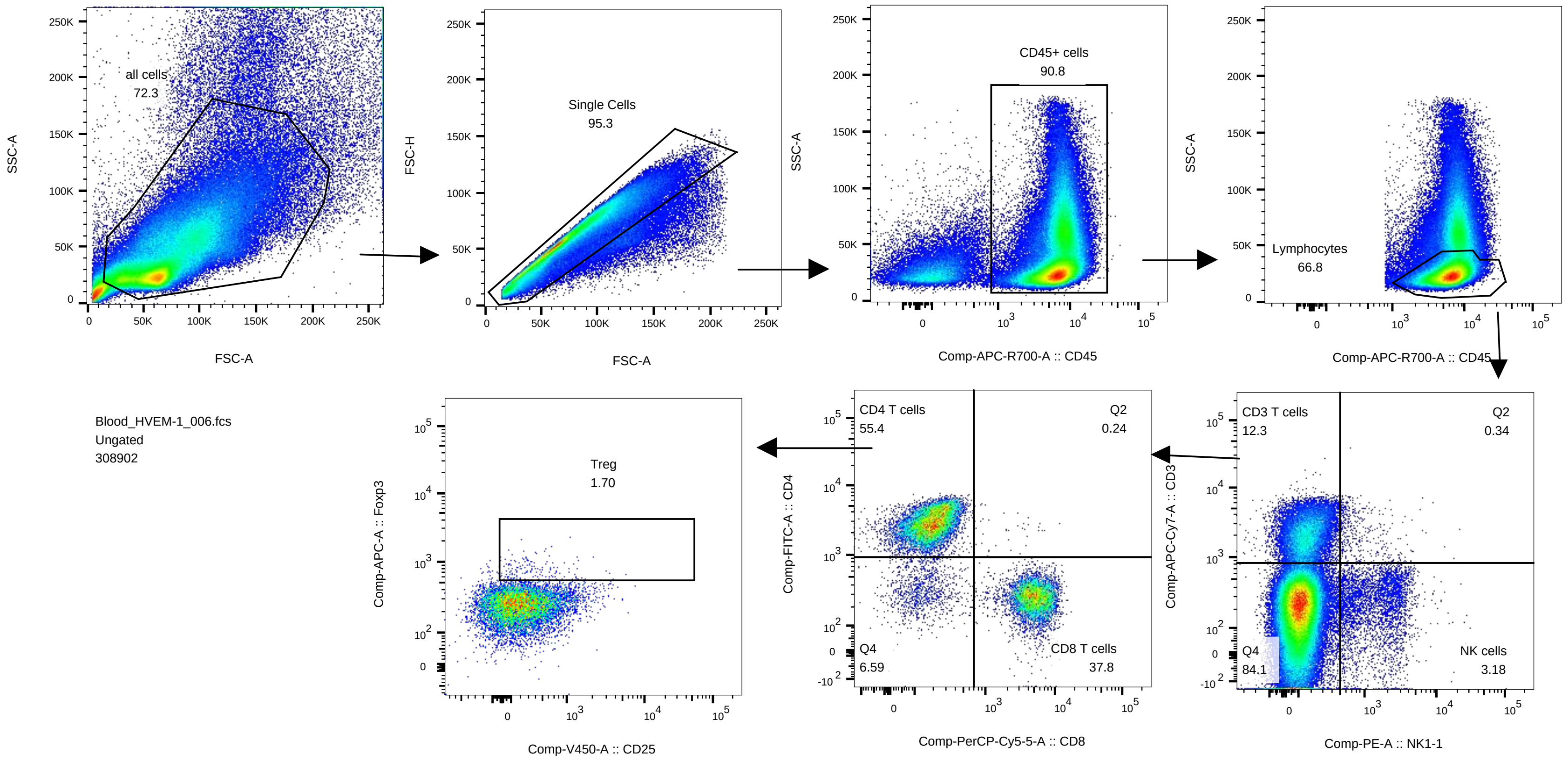

all cells
72.3
SSC-A
FSC-A
Single Cells
95.3
FSC-H
CD45+ cells
90.8
Comp-APC-R700-A :: CD45
Lymphocytes
66.8
CD3 T cells
12.3
Q2
0.34
Q4
84.1
NK cells
3.18
Comp-APC-Cy7-A :: CD3
Comp-PE-A :: NK1-1
CD4 T cells
55.4
Q2
0.24
Q4
6.59
CD8 T cells
37.8
Comp-FITC-A :: CD4
Comp-PerCP-Cy5-5-A :: CD8
Treg
1.70
Comp-APC-A :: Foxp3
Comp-V450-A :: CD25
Blood_HVEM-1_006.fcs
Ungated
308902

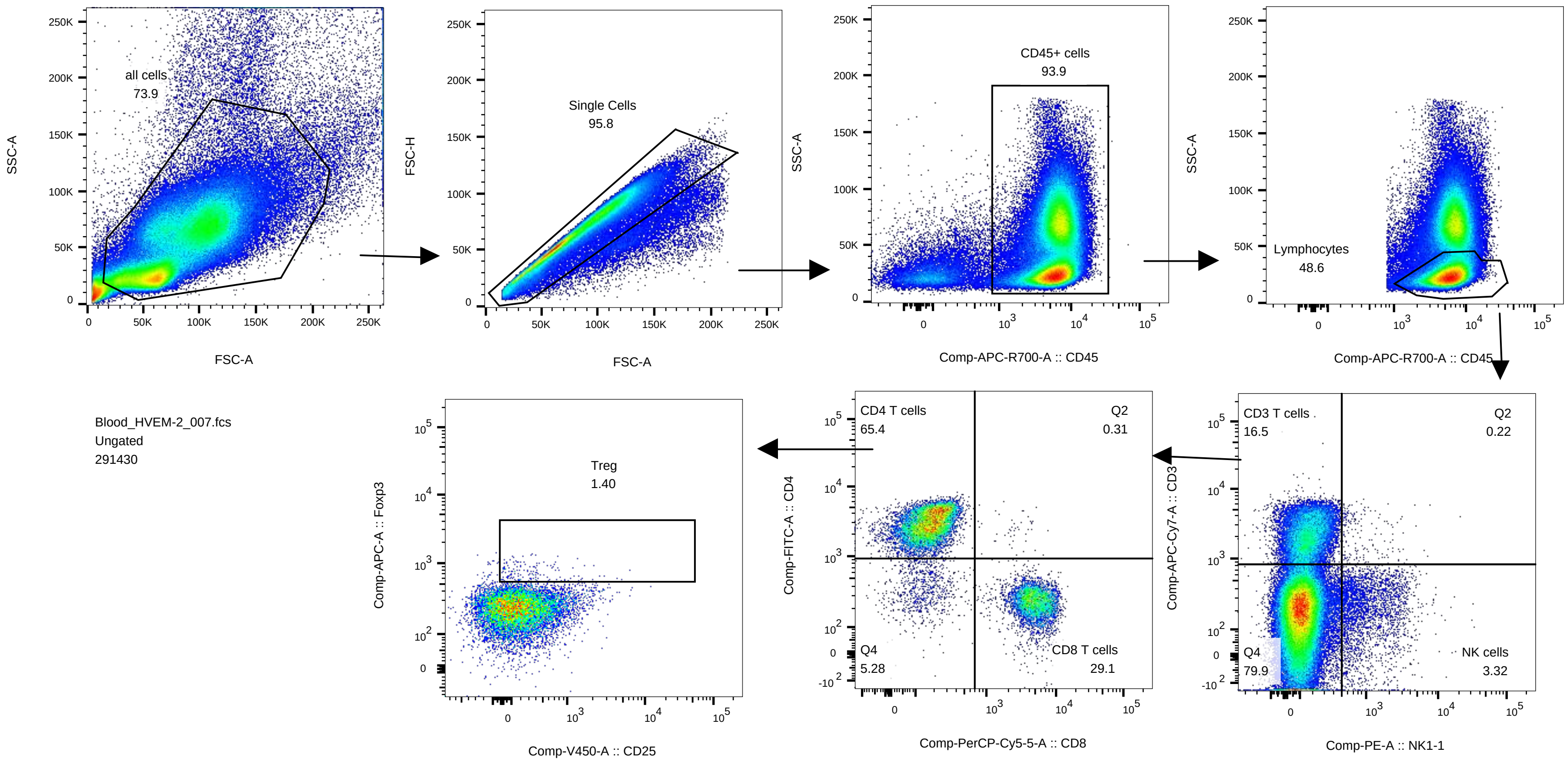
all cells
73.9
SSC-A
FSC-A
Single Cells
95.8
FSC-H
CD45+ cells
93.9
Comp-APC-R700-A :: CD45
Lymphocytes
48.6
Blood_HVEM-2_007.fcs
Ungated
291430
Treg
1.40
Comp-APC-A :: Foxp3
Comp-V450-A :: CD25
CD4 T cells
65.4
Q2
0.31
Q4
5.28
CD8 T cells
29.1
Comp-FITC-A :: CD4
Comp-PerCP-Cy5-5-A :: CD8
CD3 T cells
16.5
Q2
0.22
Q4
79.9
NK cells
3.32
Comp-APC-Cy7-A :: CD3
Comp-PE-A :: NK1-1

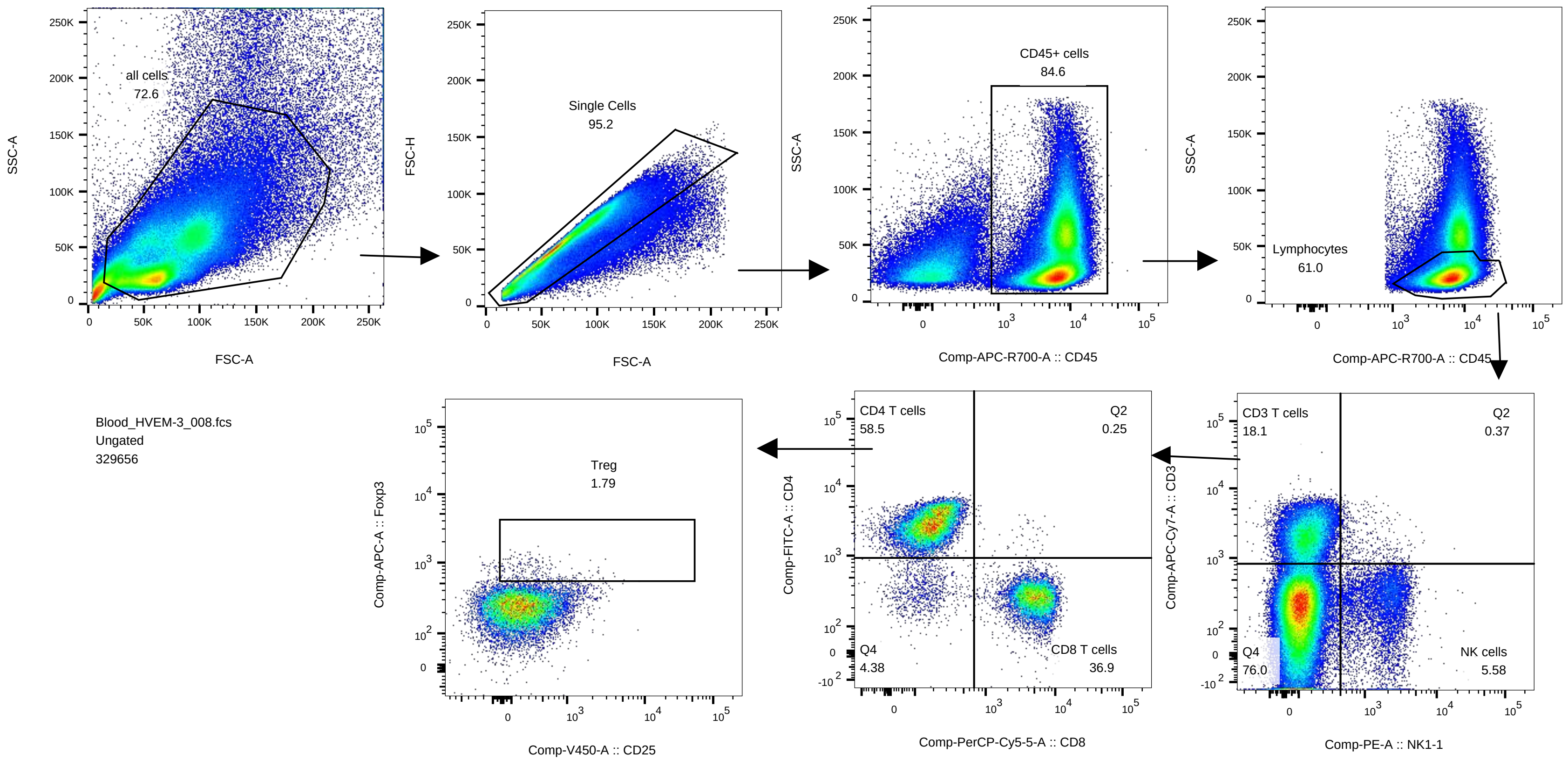

all cells
72.6
SSC-A
FSC-A
Single Cells
95.2
FSC-H
CD45+ cells
84.6
Comp-APC-R700-A :: CD45
Lymphocytes
61.0
Blood_HVEM-3_008.fcs
Ungated
329656
Treg
1.79
Comp-APC-A :: Foxp3
Comp-V450-A :: CD25
CD4 T cells
58.5
Q2
0.25
Q4
4.38
CD8 T cells
36.9
Comp-FITC-A :: CD4
Comp-PerCP-Cy5-5-A :: CD8
CD3 T cells
18.1
Q2
0.37
Q4
76.0
NK cells
5.58
Comp-APC-Cy7-A :: CD3
Comp-PE-A :: NK1-1

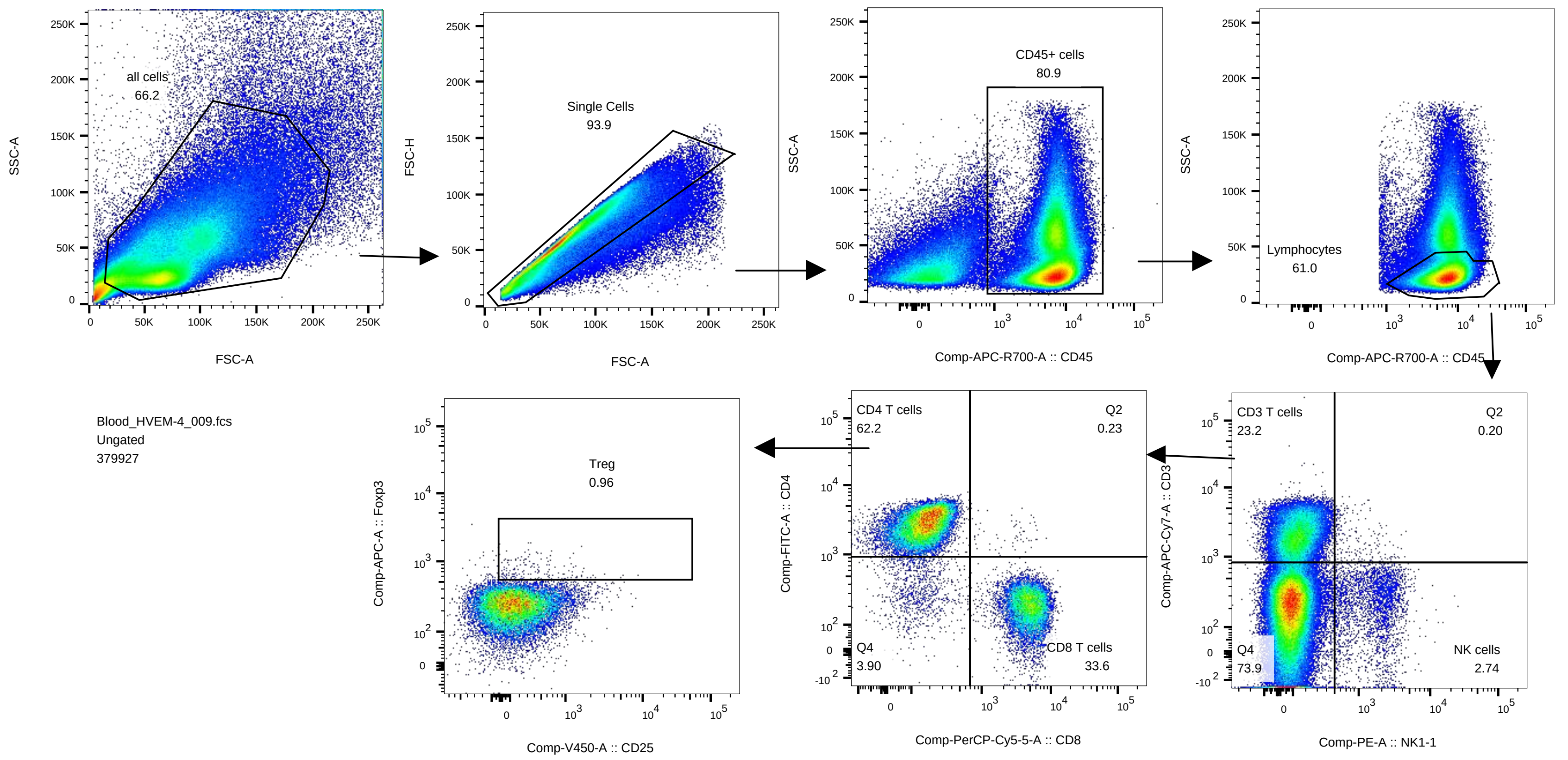
all cells
66.2
Single Cells
93.9
CD45+ cells
80.9
Lymphocytes
61.0
SSC-A
FSC-H
FSC-A
Comp-APC-R700-A :: CD45
Blood_HVEM-4_009.fcs
Ungated
379927
Treg
0.96
Comp-APC-A :: Foxp3
Comp-V450-A :: CD25
CD4 T cells
62.2
Q2
0.23
Q4
3.90
CD8 T cells
33.6
Comp-FITC-A :: CD4
Comp-PerCP-Cy5-5-A :: CD8
CD3 T cells
23.2
Q2
0.20
Q4
73.9
NK cells
2.74
Comp-APC-Cy7-A :: CD3
Comp-PE-A :: NK1-1

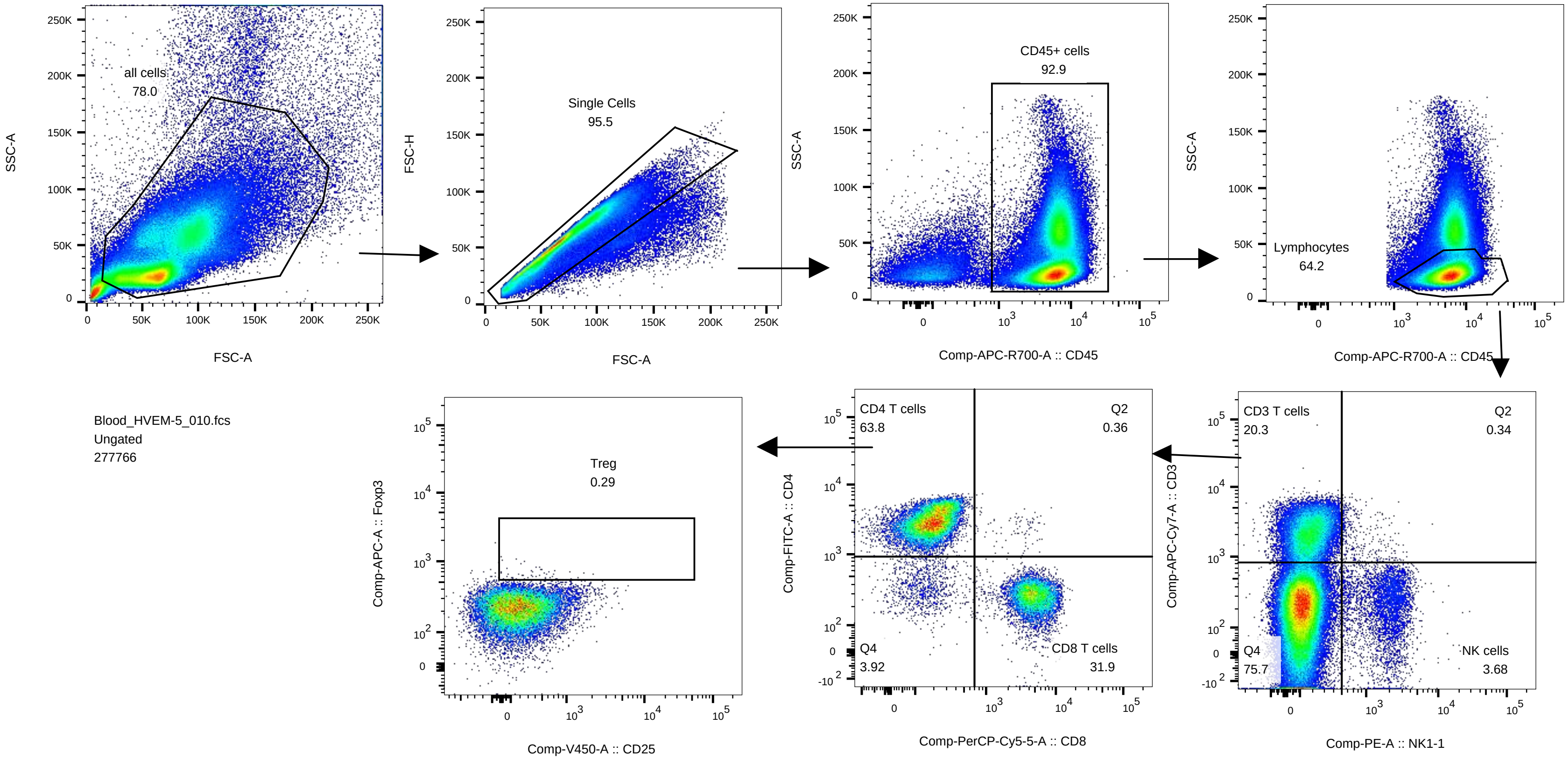

all cells
78.0
SSC-A
FSC-A
Single Cells
95.5
FSC-H
FSC-A
CD45+ cells
92.9
SSC-A
Comp-APC-R700-A :: CD45
Lymphocytes
64.2
SSC-A
Comp-APC-R700-A :: CD45
Blood_HVEM-5_010.fcs
Ungated
277766
Treg
0.29
Comp-APC-A :: Foxp3
Comp-V450-A :: CD25
CD4 T cells
63.8
Q2
0.36
Q4
3.92
CD8 T cells
31.9
Comp-FITC-A :: CD4
Comp-PerCP-Cy5-5-A :: CD8
CD3 T cells
20.3
Q2
0.34
Q4
75.7
NK cells
3.68
Comp-APC-Cy7-A :: CD3
Comp-PE-A :: NK1-1

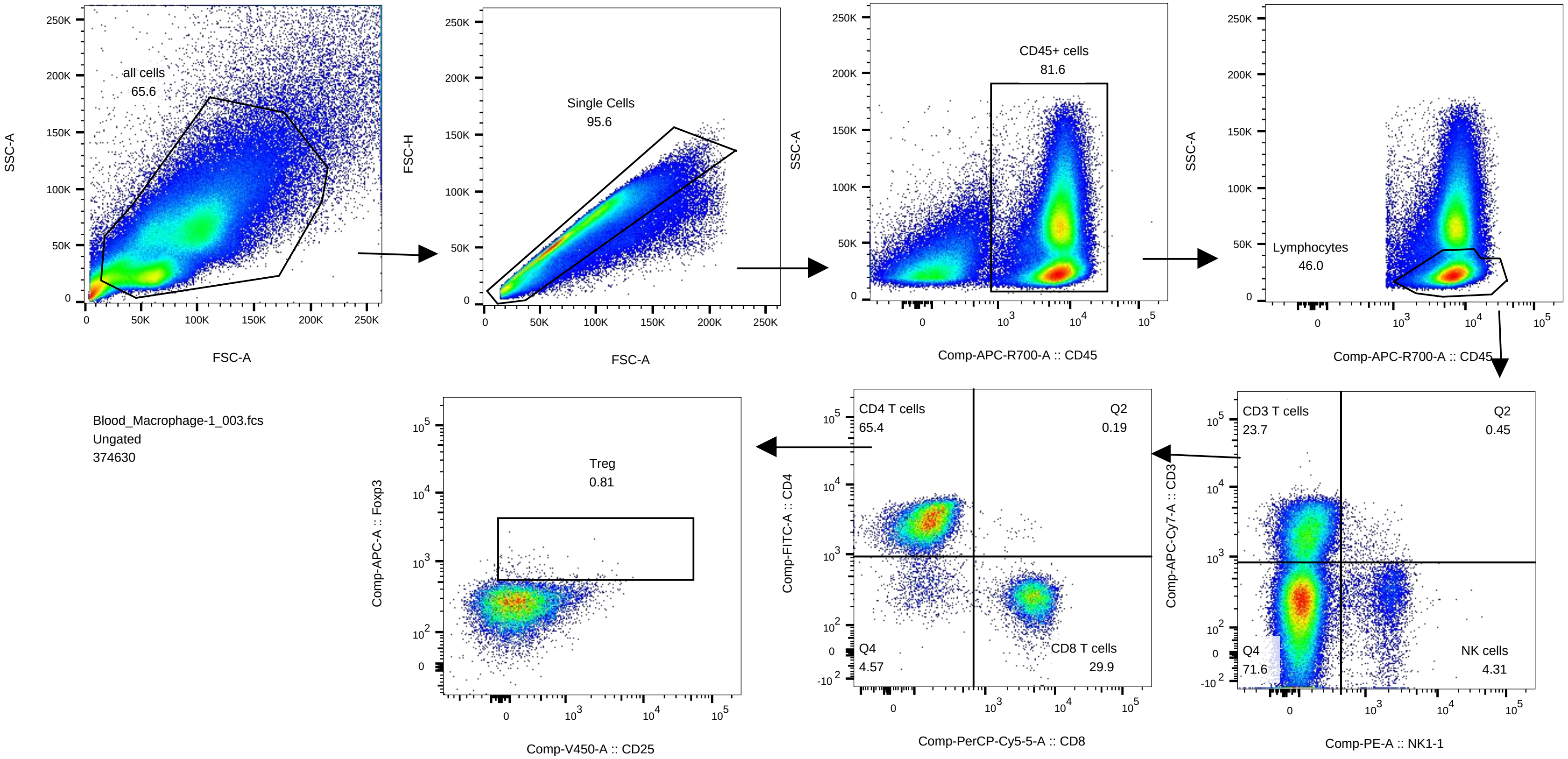
all cells
65.6
SSC-A
FSC-A
Single Cells
95.6
FSC-H
FSC-A
CD45+ cells
81.6
SSC-A
Comp-APC-R700-A :: CD45
Lymphocytes
46.0
SSC-A
Comp-APC-R700-A :: CD45
Blood_Macrophage-1_003.fcs
Ungated
374630
Treg
0.81
Comp-APC-A :: Foxp3
Comp-V450-A :: CD25
CD4 T cells
65.4
Q2
0.19
Q4
4.57
CD8 T cells
29.9
Comp-FITC-A :: CD4
Comp-PerCP-Cy5-5-A :: CD8
CD3 T cells
23.7
Q2
0.45
Q4
71.6
NK cells
4.31
Comp-APC-Cy7-A :: CD3
Comp-PE-A :: NK1-1

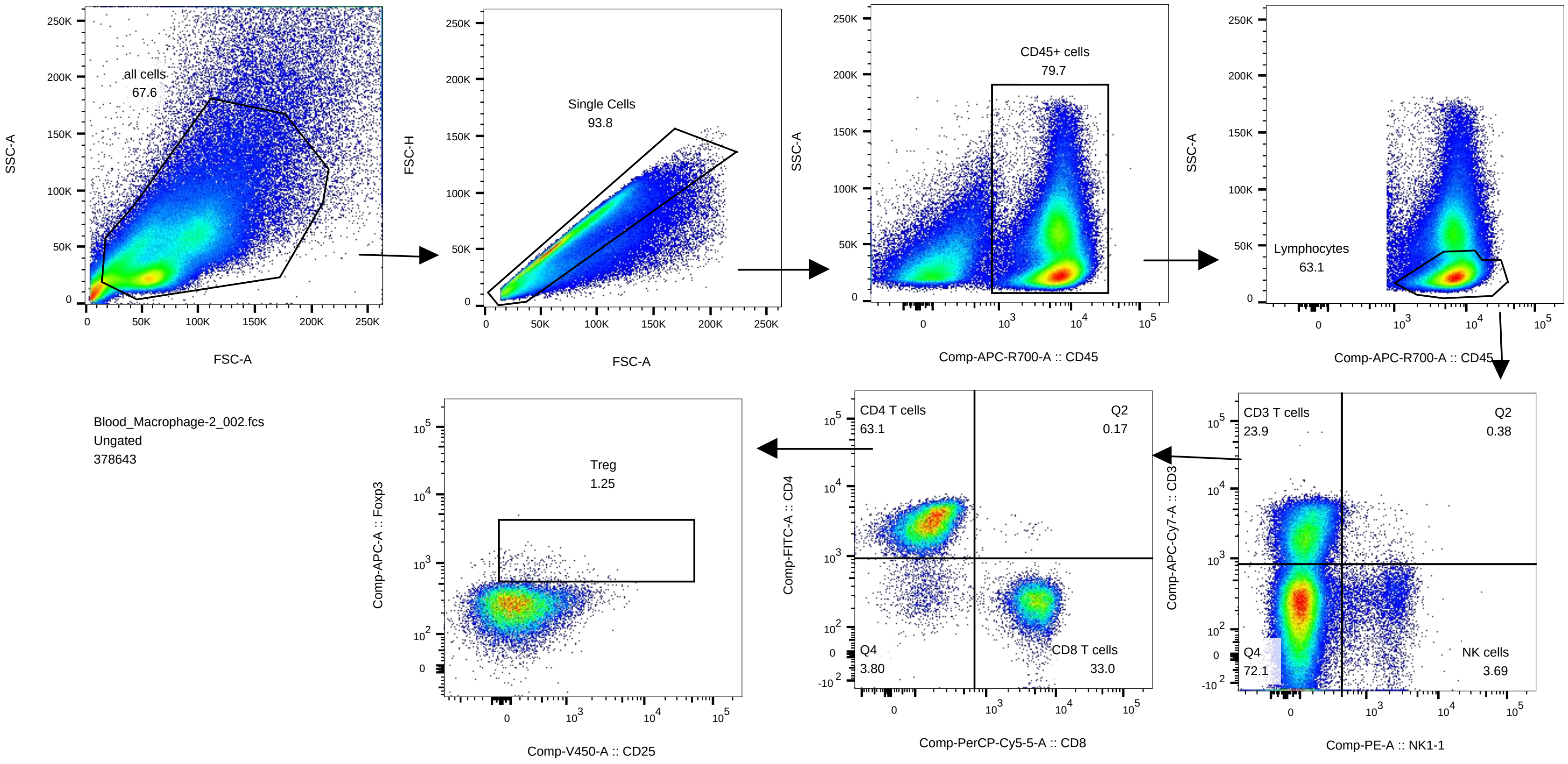
all cells
67.6
SSC-A
FSC-A
Single Cells
93.8
FSC-H
FSC-A
CD45+ cells
79.7
SSC-A
Comp-APC-R700-A :: CD45
Lymphocytes
63.1
SSC-A
Comp-APC-R700-A :: CD45
Blood_Macrophage-2_002.fcs
Ungated
378643
Treg
1.25
Comp-APC-A :: Foxp3
Comp-V450-A :: CD25
CD4 T cells
63.1
Q2
0.17
Q4
3.80
CD8 T cells
33.0
Comp-FITC-A :: CD4
Comp-PerCP-Cy5-5-A :: CD8
CD3 T cells
23.9
Q2
0.38
Q4
72.1
NK cells
3.69
Comp-APC-Cy7-A :: CD3
Comp-PE-A :: NK1-1

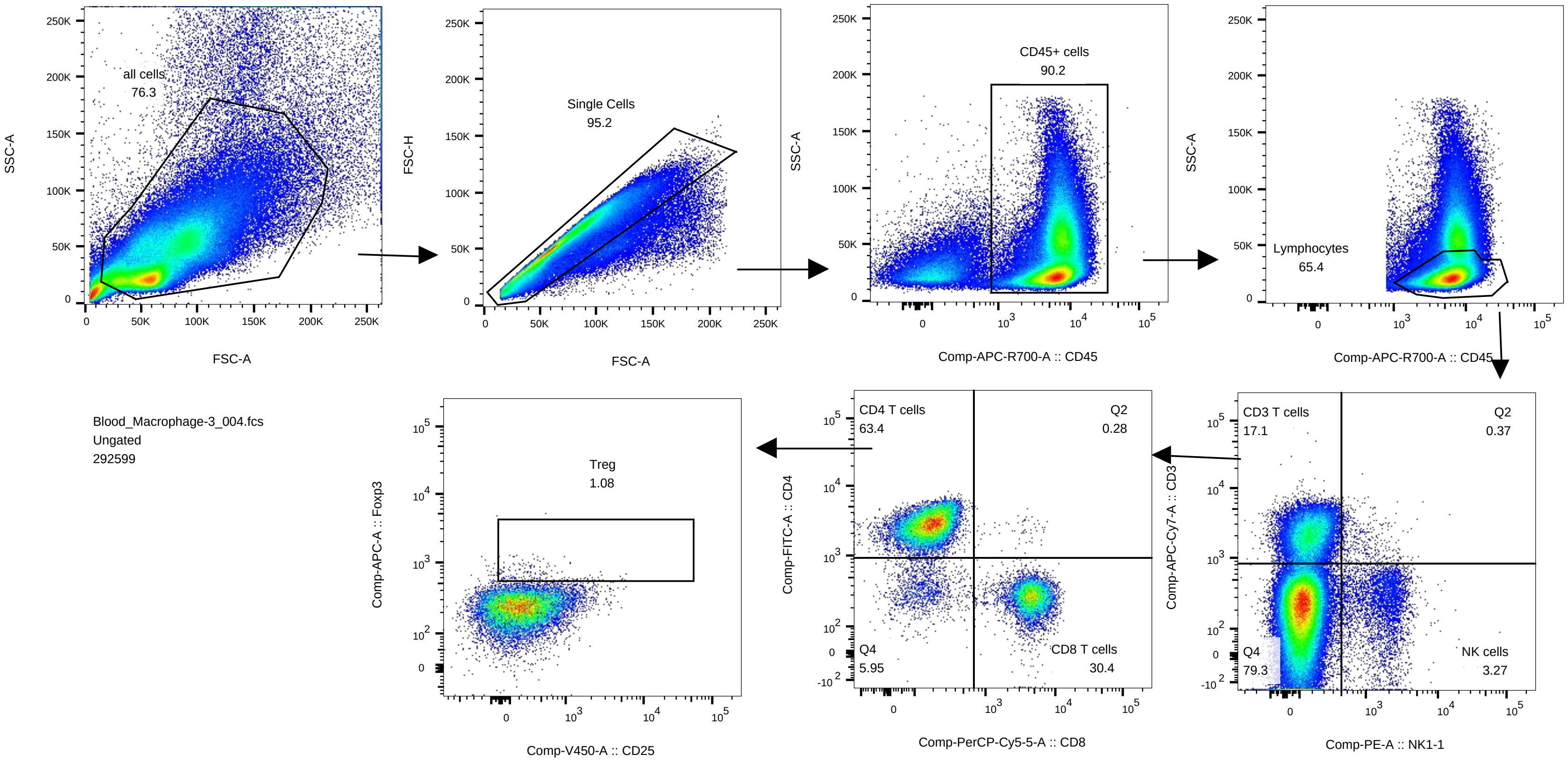
all cells
76.3
SSC-A
FSC-A
Single Cells
95.2
FSC-H
FSC-A
CD45+ cells
90.2
SSC-A
Comp-APC-R700-A :: CD45
Lymphocytes
65.4
SSC-A
Comp-APC-R700-A :: CD45
Blood_Macrophage-3_004.fcs
Ungated
292599
Treg
1.08
Comp-APC-A :: Foxp3
Comp-V450-A :: CD25
CD4 T cells
63.4
Q2
0.28
Q4
5.95
CD8 T cells
30.4
Comp-FITC-A :: CD4
Comp-PerCP-Cy5-5-A :: CD8
CD3 T cells
17.1
Q2
0.37
Q4
79.3
NK cells
3.27
Comp-APC-Cy7-A :: CD3
Comp-PE-A :: NK1-1

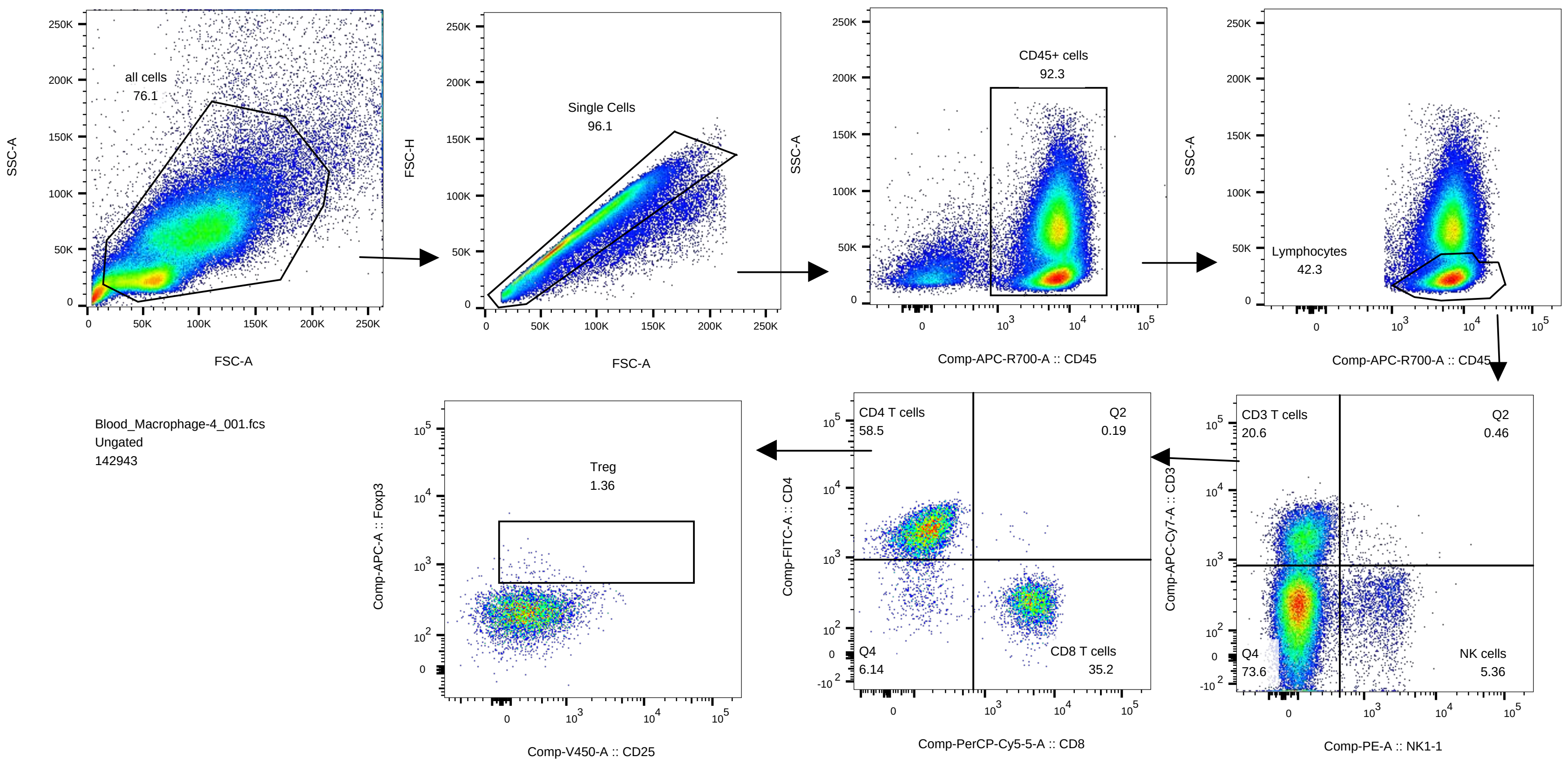
all cells
76.1
FSC-A
SSC-A
Single Cells
96.1
FSC-H
CD45+ cells
92.3
Comp-APC-R700-A :: CD45
Lymphocytes
42.3
Blood_Macrophage-4_001.fcs
Ungated
142943
Treg
1.36
Comp-APC-A :: Foxp3
Comp-V450-A :: CD25
CD4 T cells
58.5
Q2
0.19
Q4
6.14
CD8 T cells
35.2
Comp-FITC-A :: CD4
Comp-PerCP-Cy5-5-A :: CD8
CD3 T cells
20.6
Q2
0.46
Q4
73.6
NK cells
5.36
Comp-APC-Cy7-A :: CD3
Comp-PE-A :: NK1-1

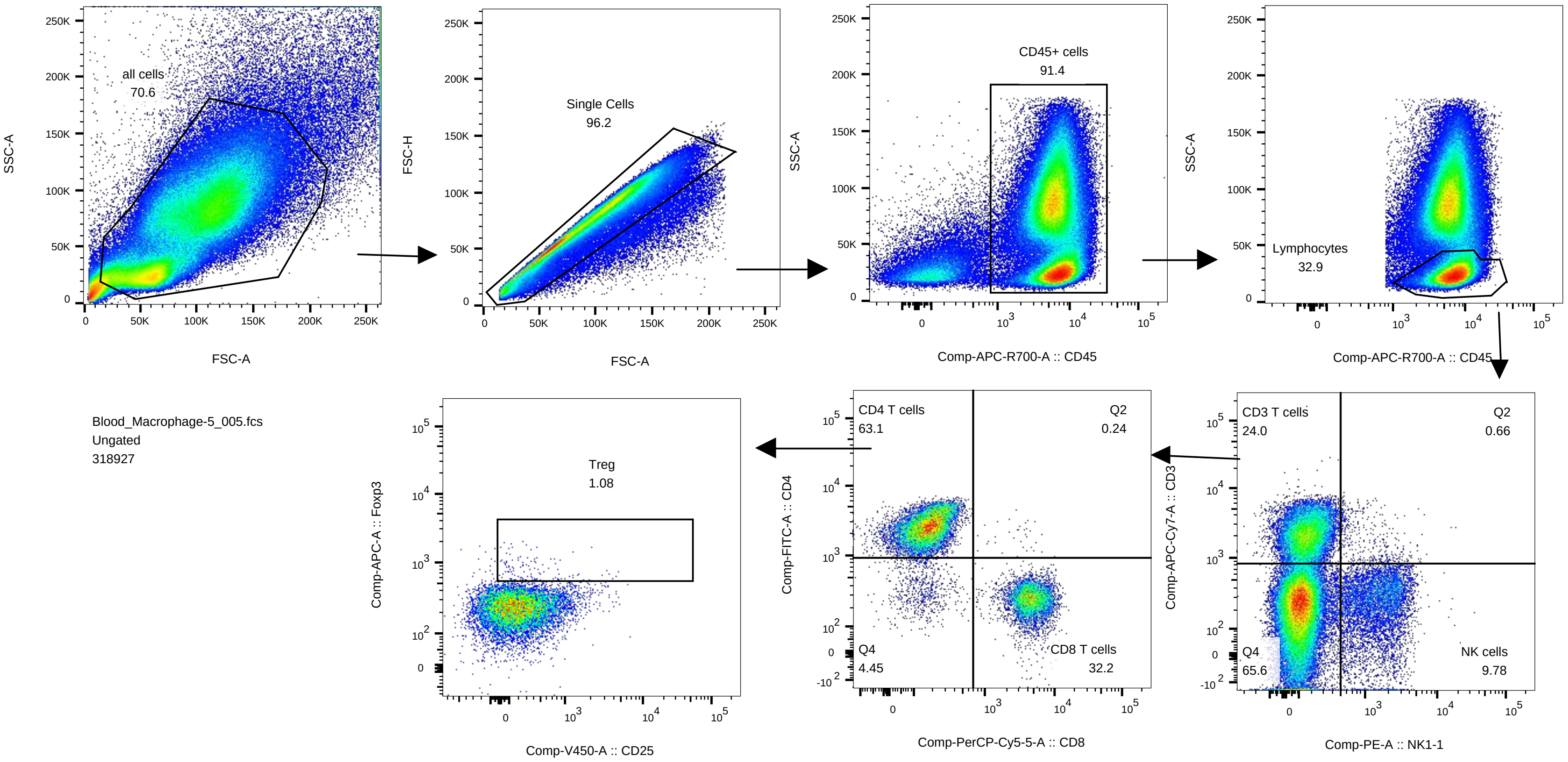
all cells
70.6
SSC-A
FSC-A
Single Cells
96.2
FSC-H
CD45+ cells
91.4
Comp-APC-R700-A :: CD45
Lymphocytes
32.9
Blood_Macrophage-5_005.fcs
Ungated
318927
Treg
1.08
Comp-APC-A :: Foxp3
Comp-V450-A :: CD25
CD4 T cells
63.1
Q2
0.24
Q4
4.45
CD8 T cells
32.2
Comp-FITC-A :: CD4
Comp-PerCP-Cy5-5-A :: CD8
CD3 T cells
24.0
Q2
0.66
Q4
65.6
NK cells
9.78
Comp-APC-Cy7-A :: CD3
Comp-PE-A :: NK1-1

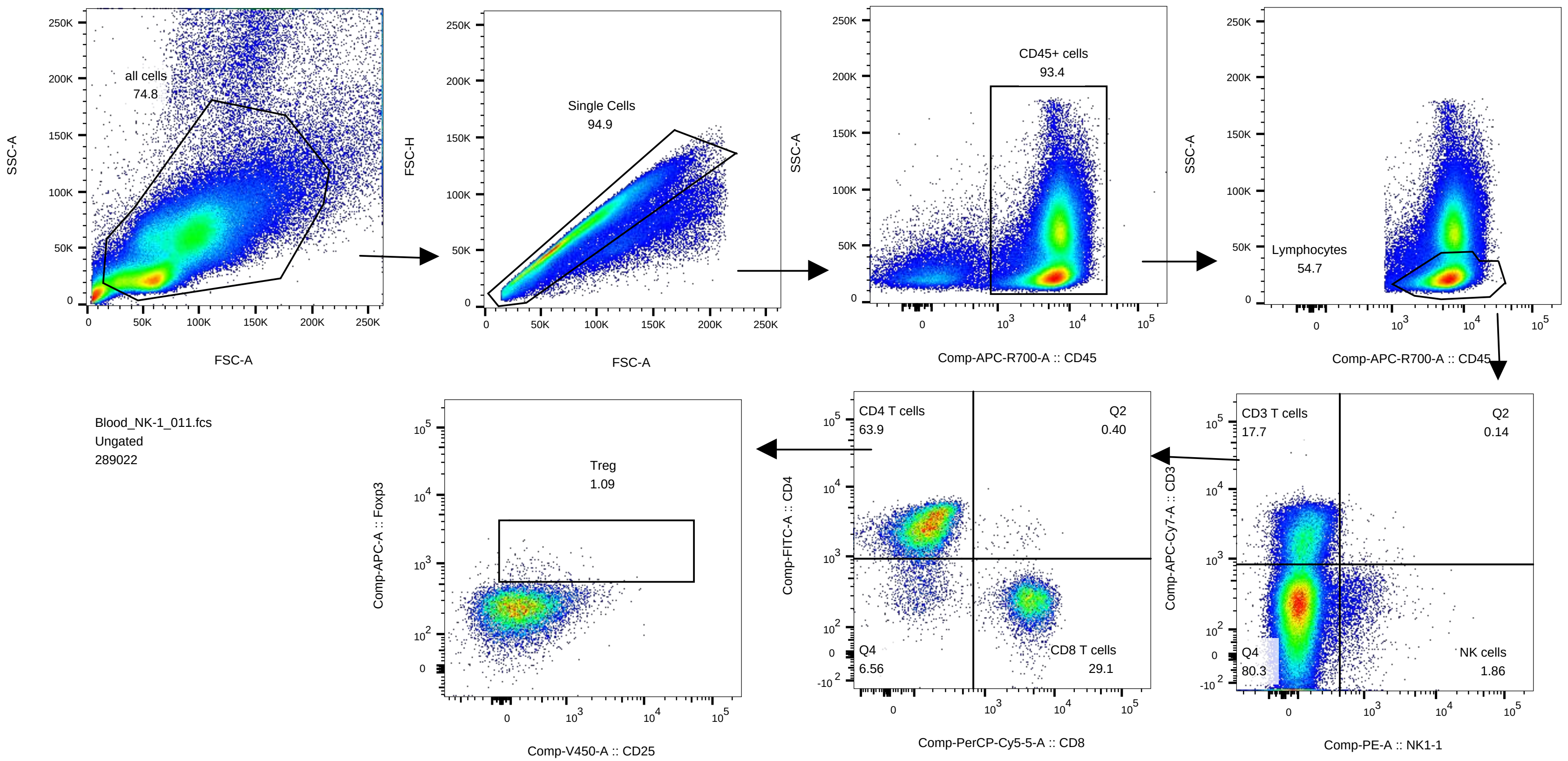

all cells
74.8
SSC-A
FSC-A
Single Cells
94.9
FSC-H
FSC-A
CD45+ cells
93.4
SSC-A
Comp-APC-R700-A :: CD45
Lymphocytes
54.7
SSC-A
Comp-APC-R700-A :: CD45
Blood_NK-1_011.fcs
Ungated
289022
Treg
1.09
Comp-APC-A :: Foxp3
Comp-V450-A :: CD25
CD4 T cells
63.9
Q2
0.40
Q4
6.56
CD8 T cells
29.1
Comp-FITC-A :: CD4
Comp-PerCP-Cy5-5-A :: CD8
CD3 T cells
17.7
Q2
0.14
Q4
80.3
NK cells
1.86
Comp-APC-Cy7-A :: CD3
Comp-PE-A :: NK1-1

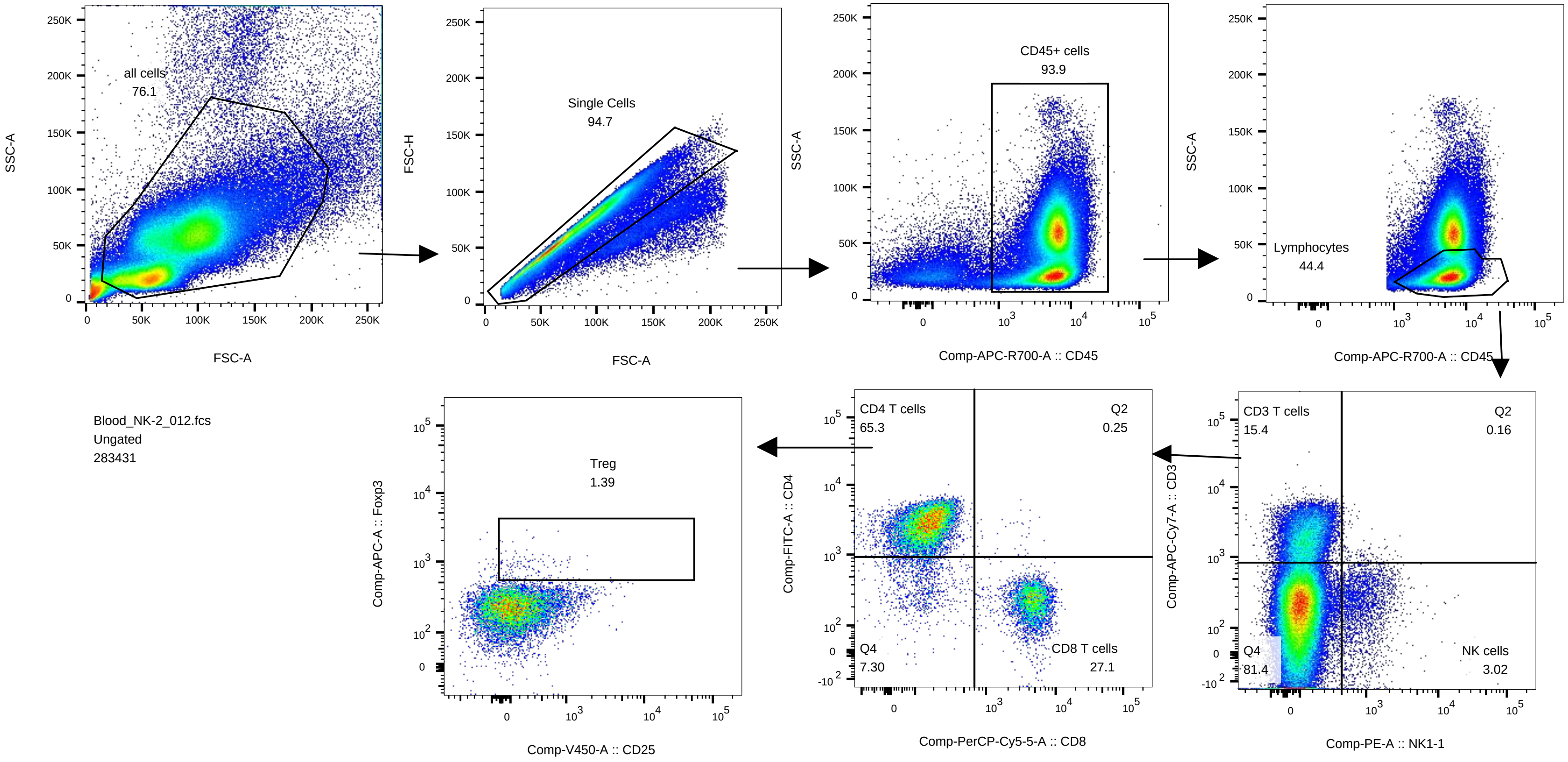

all cells
76.1
SSC-A
FSC-A
Single Cells
94.7
FSC-H
FSC-A
CD45+ cells
93.9
SSC-A
Comp-APC-R700-A :: CD45
Lymphocytes
44.4
SSC-A
Comp-APC-R700-A :: CD45
Blood_NK-2_012.fcs
Ungated
283431
Treg
1.39
Comp-APC-A :: Foxp3
Comp-V450-A :: CD25
CD4 T cells
65.3
Q2
0.25
Q4
7.30
CD8 T cells
27.1
Comp-FITC-A :: CD4
Comp-PerCP-Cy5-5-A :: CD8
CD3 T cells
15.4
Q2
0.16
Q4
81.4
NK cells
3.02
Comp-APC-Cy7-A :: CD3
Comp-PE-A :: NK1-1

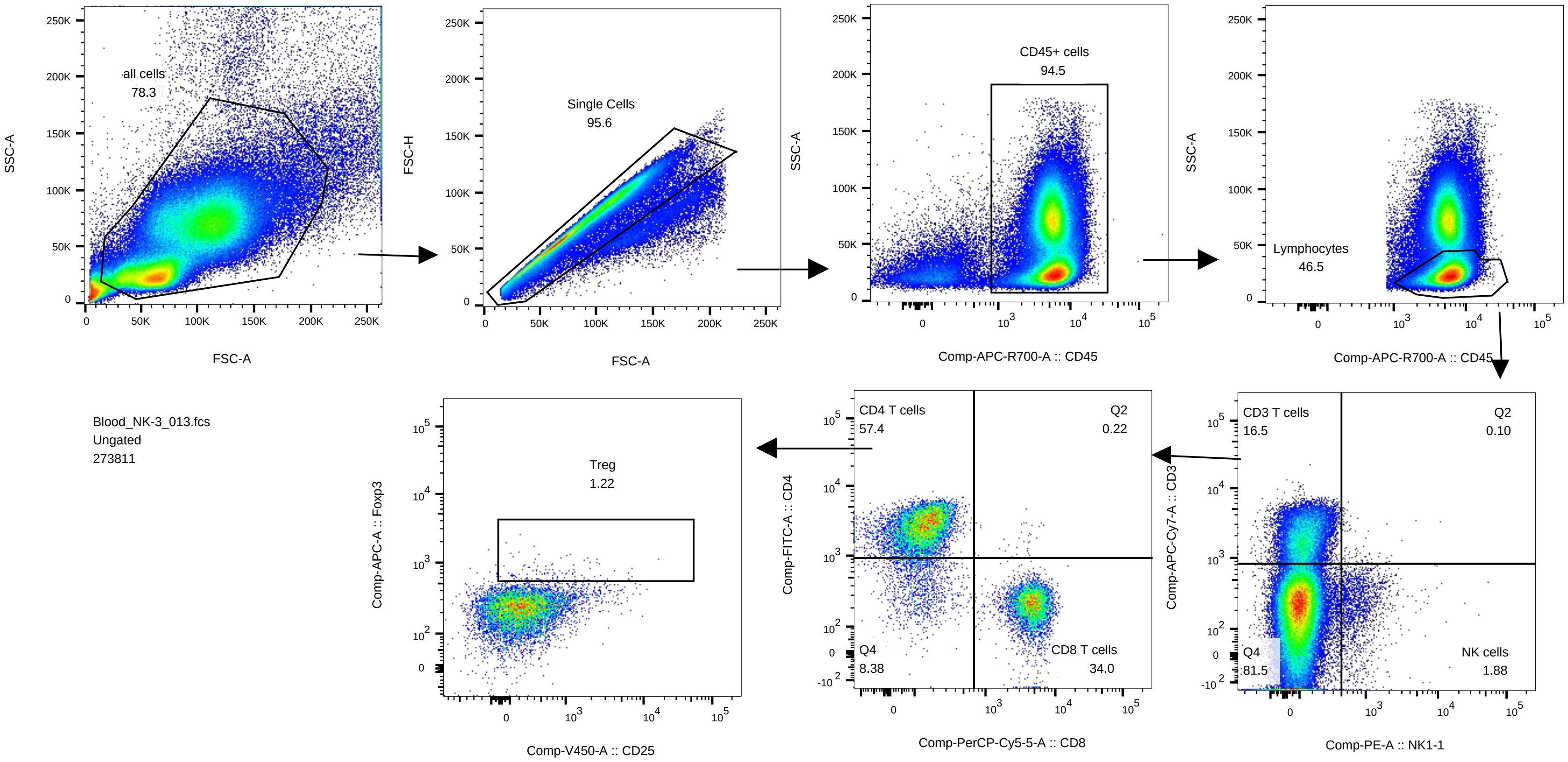
all cells
78.3
SSC-A
FSC-A
Single Cells
95.6
FSC-H
FSC-A
CD45+ cells
94.5
SSC-A
Comp-APC-R700-A :: CD45
Lymphocytes
46.5
SSC-A
Comp-APC-R700-A :: CD45
Blood_NK-3_013.fcs
Ungated
273811
Treg
1.22
Comp-APC-A :: Foxp3
Comp-V450-A :: CD25
CD4 T cells
57.4
Q2
0.22
Q4
8.38
CD8 T cells
34.0
Comp-FITC-A :: CD4
Comp-PerCP-Cy5-5-A :: CD8
CD3 T cells
16.5
Q2
0.10
Q4
81.5
NK cells
1.88
Comp-APC-Cy7-A :: CD3
Comp-PE-A :: NK1-1

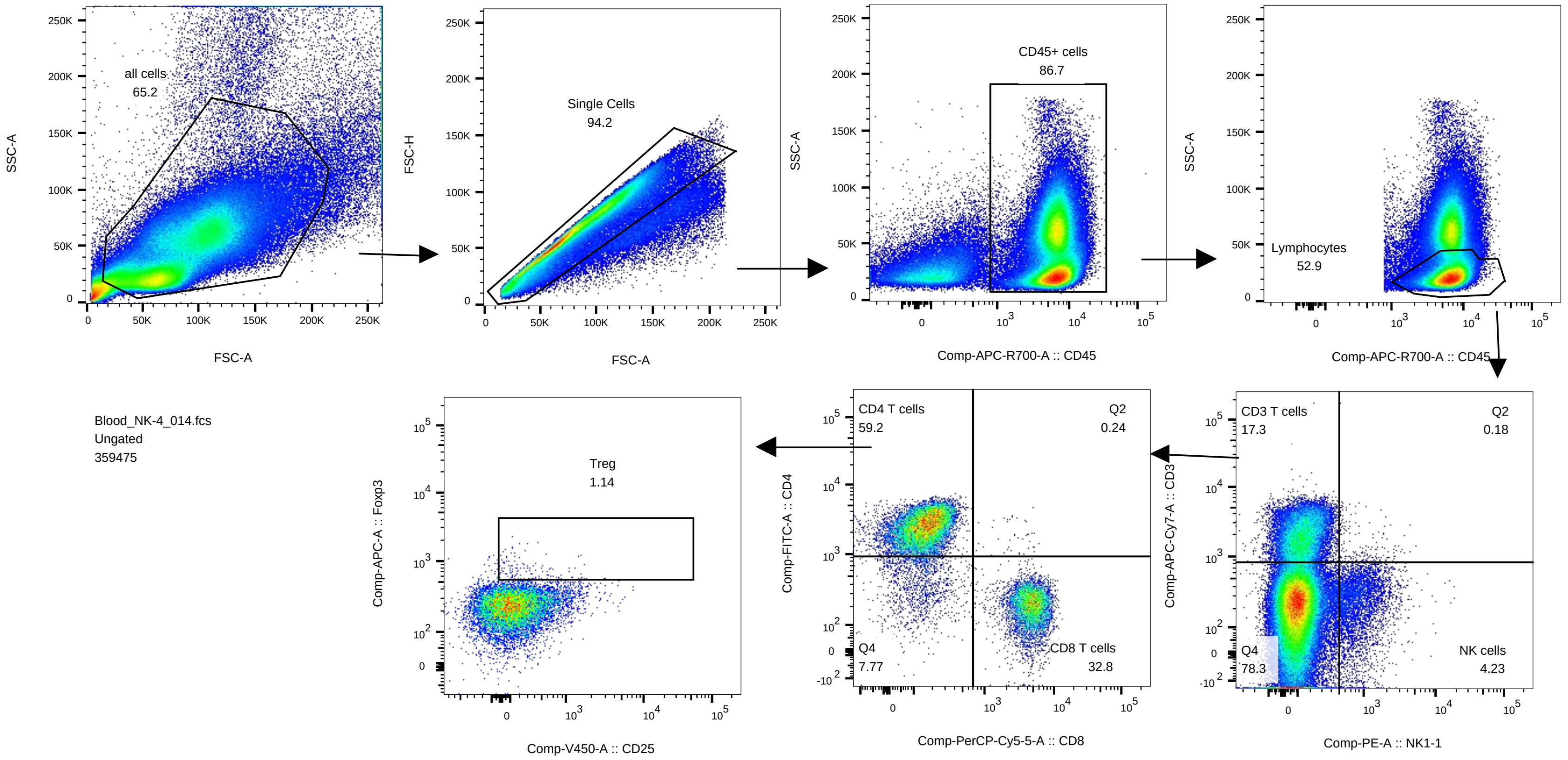

all cells
65.2
SSC-A
FSC-A
Single Cells
94.2
FSC-H
FSC-A
CD45+ cells
86.7
SSC-A
Comp-APC-R700-A :: CD45
Lymphocytes
52.9
SSC-A
Comp-APC-R700-A :: CD45
Blood_NK-4_014.fcs
Ungated
359475
Treg
1.14
Comp-APC-A :: Foxp3
Comp-V450-A :: CD25
CD4 T cells
59.2
Q2
0.24
Q4
7.77
CD8 T cells
32.8
Comp-FITC-A :: CD4
Comp-PerCP-Cy5-5-A :: CD8
CD3 T cells
17.3
Q2
0.18
Q4
78.3
NK cells
4.23
Comp-APC-Cy7-A :: CD3
Comp-PE-A :: NK1-1

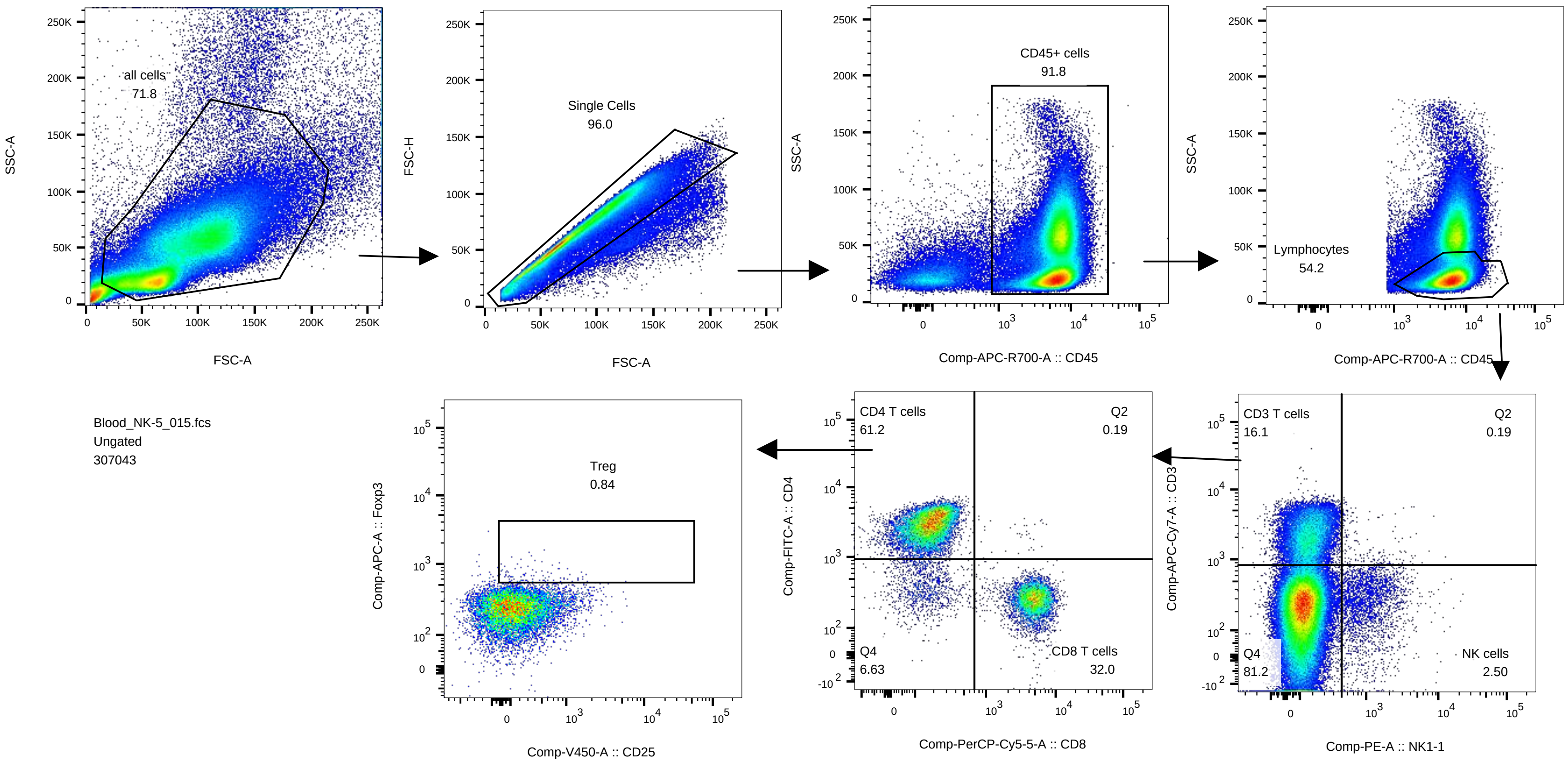

all cells
71.8
SSC-A
FSC-A
Single Cells
96.0
FSC-H
CD45+ cells
91.8
Comp-APC-R700-A :: CD45
Lymphocytes
54.2
CD3 T cells
16.1
Q2
0.19
Q4
81.2
NK cells
2.50
Comp-APC-Cy7-A :: CD3
Comp-PE-A :: NK1-1
CD4 T cells
61.2
Q4
6.63
CD8 T cells
32.0
Comp-FITC-A :: CD4
Comp-PerCP-Cy5-5-A :: CD8
Treg
0.84
Comp-APC-A :: Foxp3
Comp-V450-A :: CD25
Blood_NK-5_015.fcs
Ungated
307043

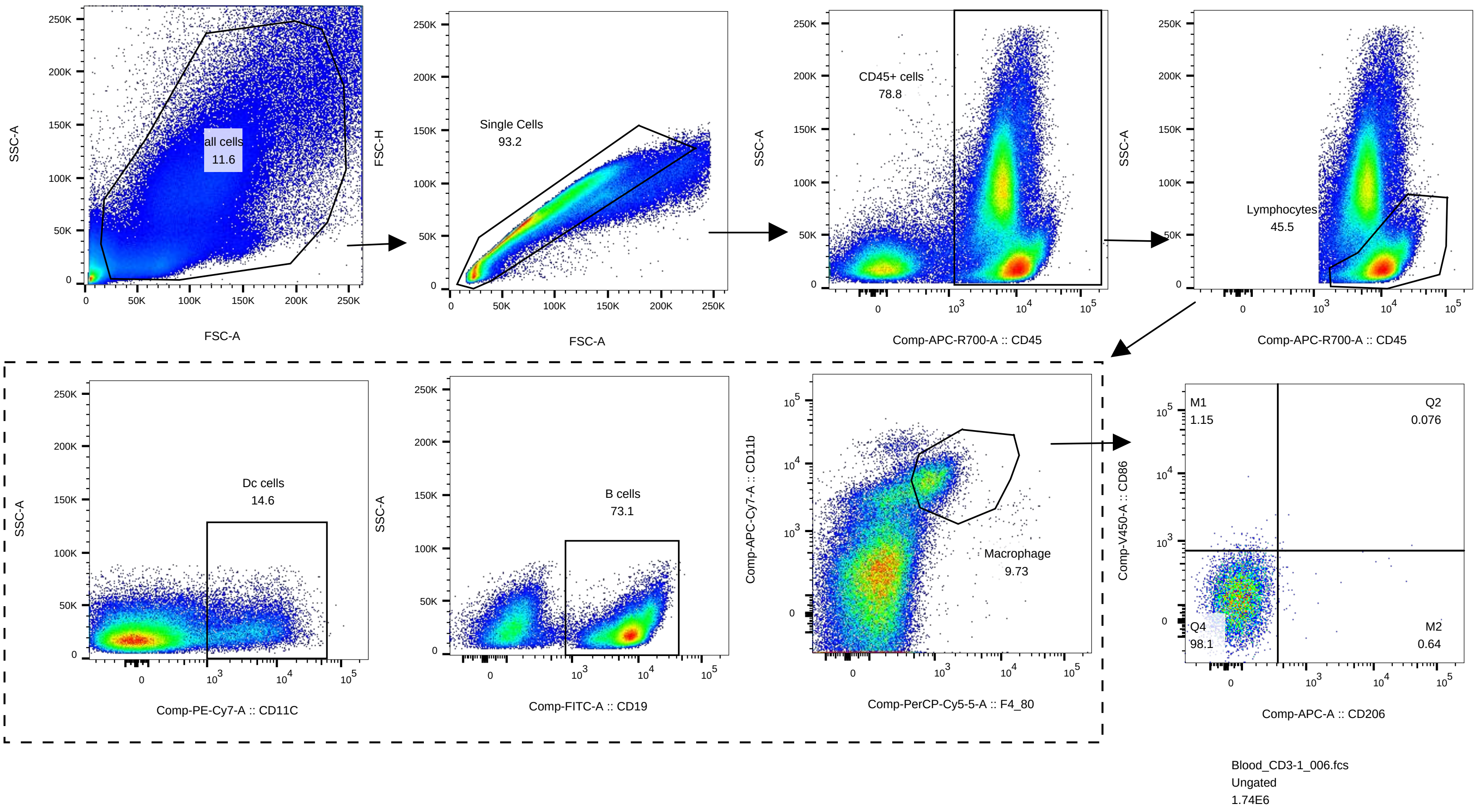
all cells
11.6
FSC-A
SSC-A
Single Cells
93.2
FSC-A
FSC-H
CD45+ cells
78.8
Comp-APC-R700-A :: CD45
SSC-A
Lymphocytes
45.5
Comp-APC-R700-A :: CD45
SSC-A
Dc cells
14.6
Comp-PE-Cy7-A :: CD11C
SSC-A
B cells
73.1
Comp-FITC-A :: CD19
SSC-A
Macrophage
9.73
Comp-PerCP-Cy5-5-A :: F4_80
Comp-APC-Cy7-A :: CD11b
M1
1.15
Q2
0.076
Q4
98.1
M2
0.64
Comp-APC-A :: CD206
Comp-V450-A :: CD86
Blood_CD3-1_006.fcs
Ungated
1.74E6

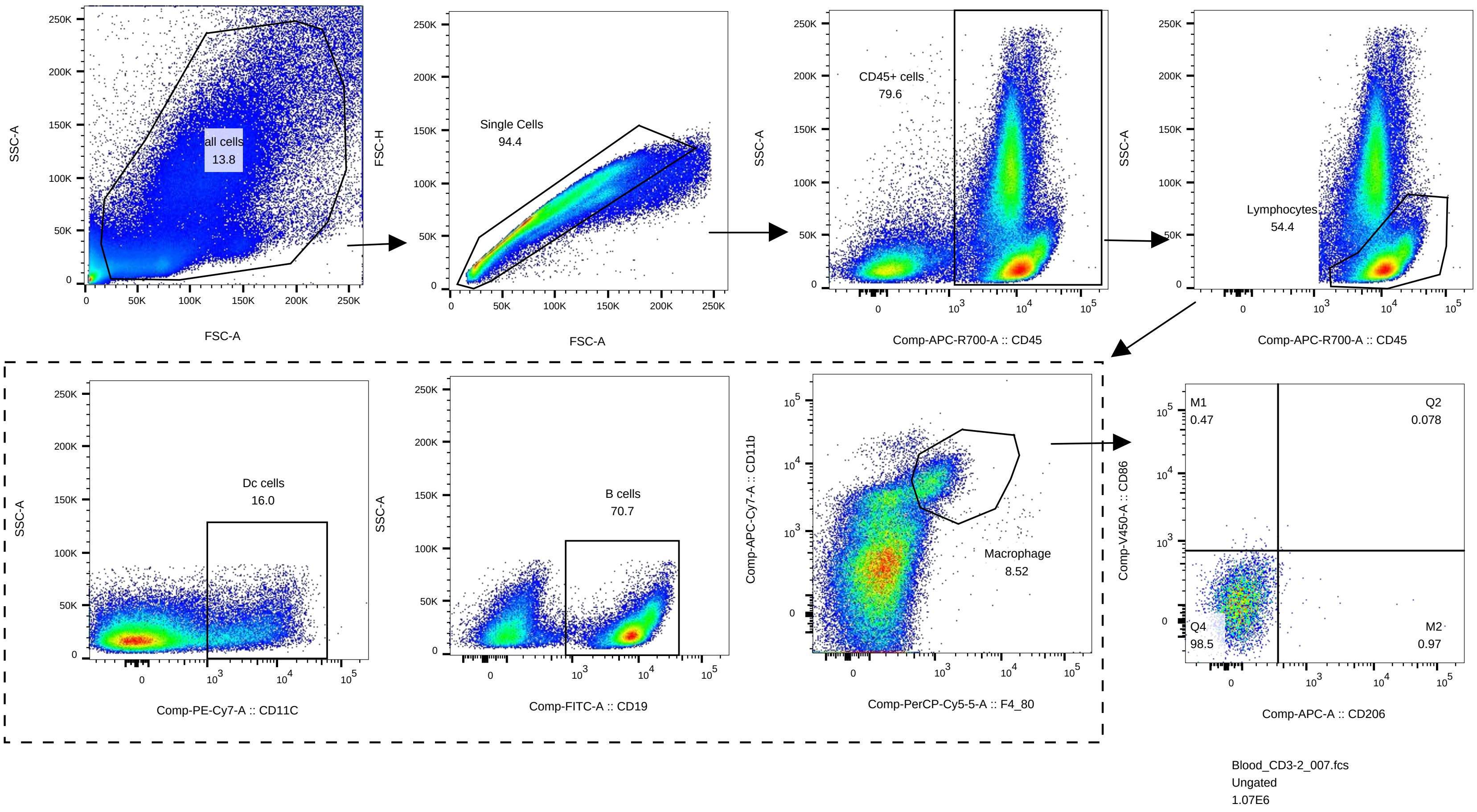
all cells
13.8
SSC-A
FSC-A
Single Cells
94.4
FSC-H
CD45+ cells
79.6
Comp-APC-R700-A :: CD45
Lymphocytes
54.4
Dc cells
16.0
Comp-PE-Cy7-A :: CD11C
B cells
70.7
Comp-FITC-A :: CD19
Macrophage
8.52
Comp-APC-Cy7-A :: CD11b
Comp-PerCP-Cy5-5-A :: F4_80
M1
0.47
Q2
0.078
Q4
98.5
M2
0.97
Comp-V450-A :: CD86
Comp-APC-A :: CD206
Blood_CD3-2_007.fcs
Ungated
1.07E6

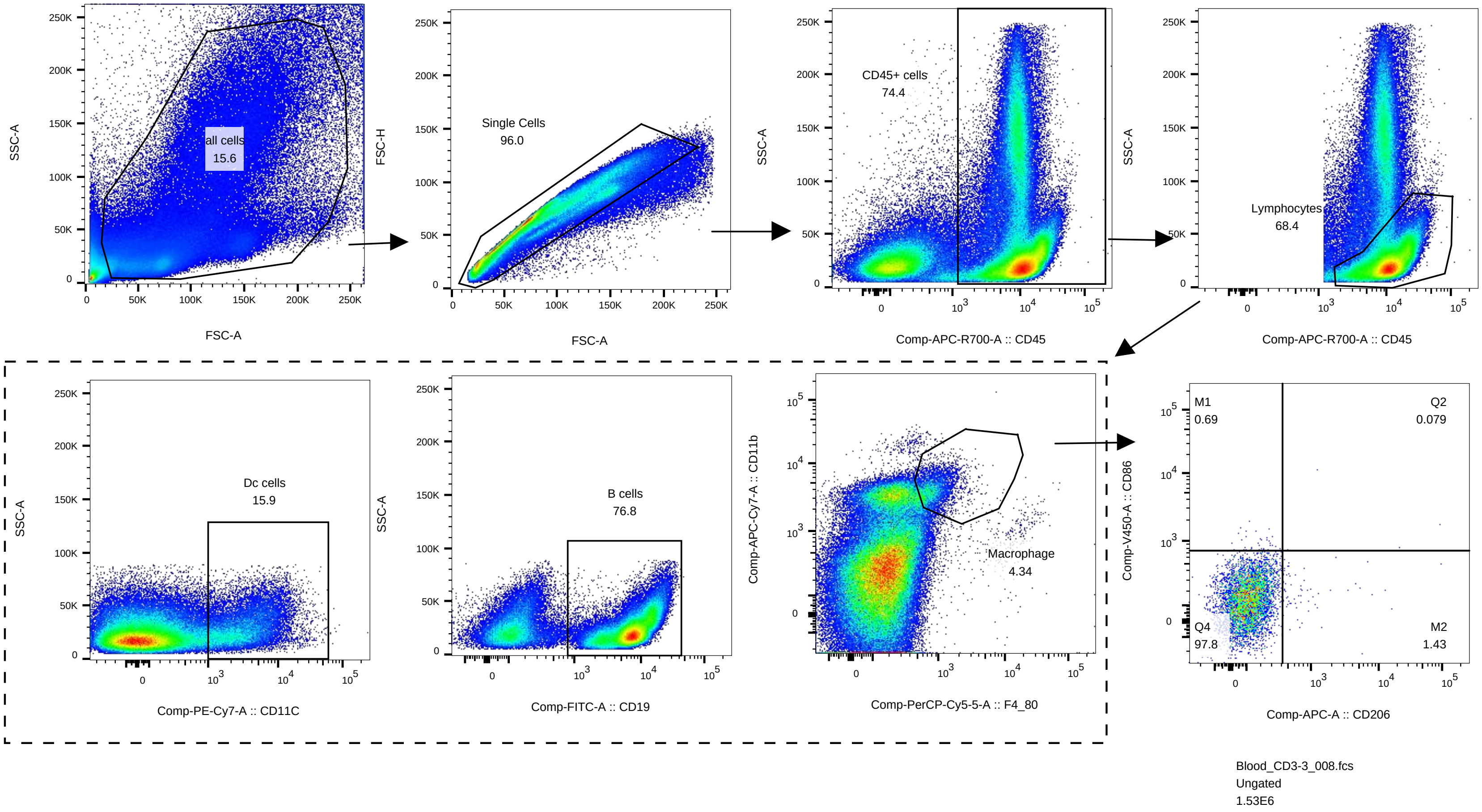
all cells
15.6
SSC-A
FSC-A
Single Cells
96.0
FSC-H
FSC-A
CD45+ cells
74.4
SSC-A
Comp-APC-R700-A :: CD45
Lymphocytes
68.4
SSC-A
Comp-APC-R700-A :: CD45
Dc cells
15.9
SSC-A
Comp-PE-Cy7-A :: CD11C
B cells
76.8
SSC-A
Comp-FITC-A :: CD19
Macrophage
4.34
Comp-APC-Cy7-A :: CD11b
Comp-PerCP-Cy5-5-A :: F4_80
M1
0.69
Q2
0.079
Q4
97.8
M2
1.43
Comp-V450-A :: CD86
Comp-APC-A :: CD206
Blood_CD3-3_008.fcs
Ungated
1.53E6

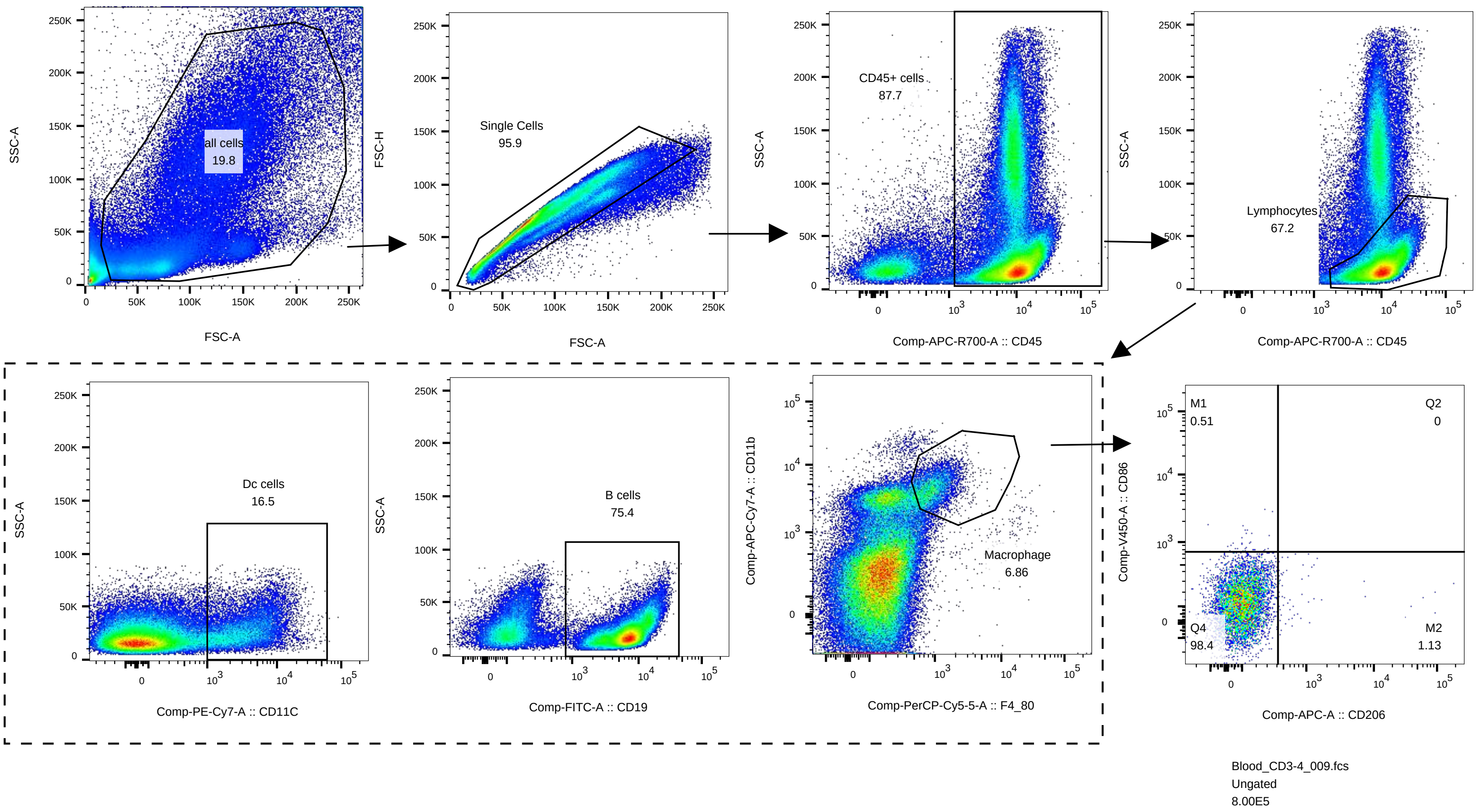
all cells
19.8
SSC-A
FSC-A
Single Cells
95.9
FSC-H
CD45+ cells
87.7
Comp-APC-R700-A :: CD45
Lymphocytes
67.2
Dc cells
16.5
Comp-PE-Cy7-A :: CD11C
B cells
75.4
Comp-FITC-A :: CD19
Macrophage
6.86
Comp-APC-Cy7-A :: CD11b
Comp-PerCP-Cy5-5-A :: F4_80
M1
0.51
Q2
0
Q4
98.4
M2
1.13
Comp-V450-A :: CD86
Comp-APC-A :: CD206
Blood_CD3-4_009.fcs
Ungated
8.00E5

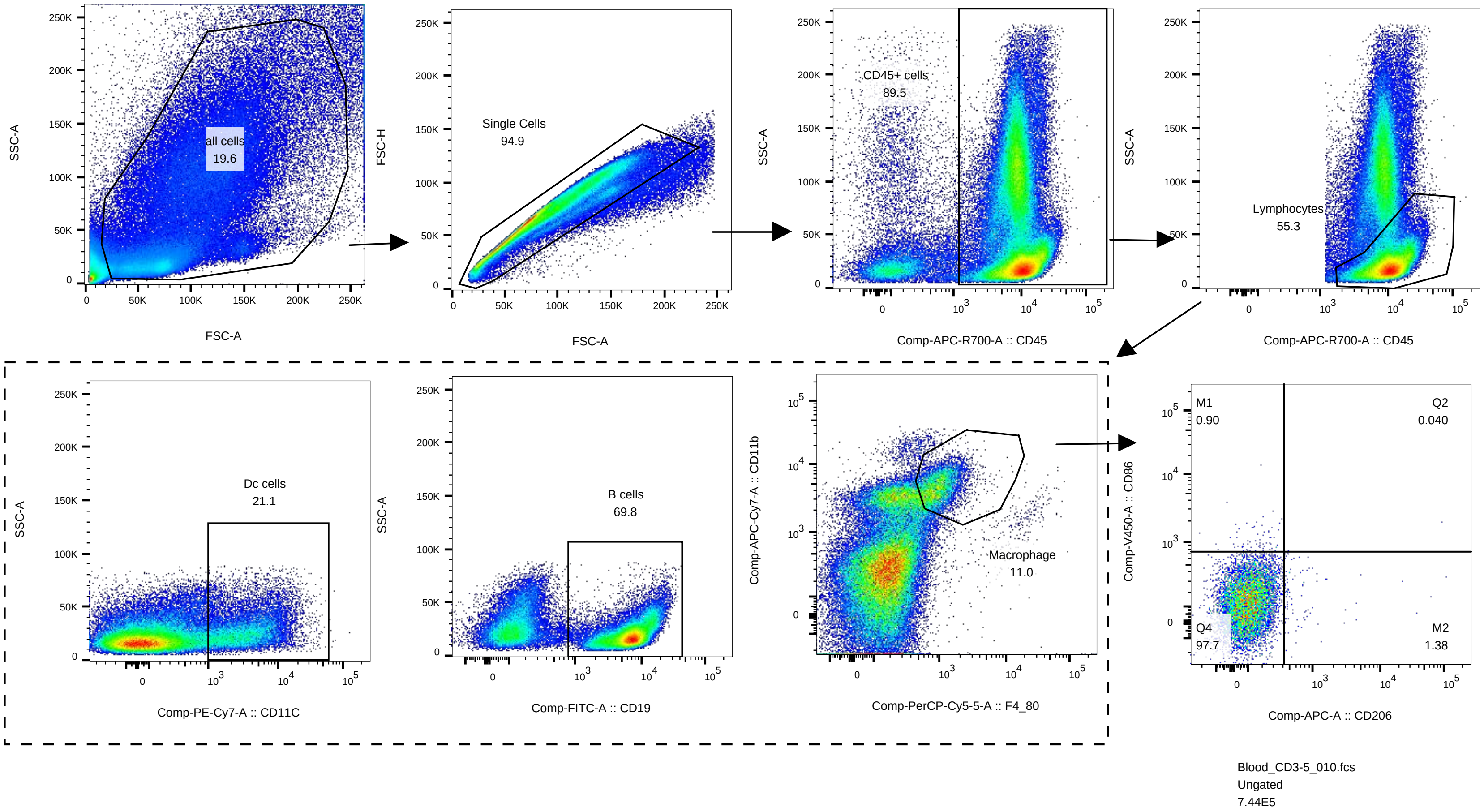
all cells
19.6
SSC-A
FSC-A
Single Cells
94.9
FSC-H
CD45+ cells
89.5
Comp-APC-R700-A :: CD45
Lymphocytes
55.3
Dc cells
21.1
Comp-PE-Cy7-A :: CD11C
B cells
69.8
Comp-FITC-A :: CD19
Macrophage
11.0
Comp-APC-Cy7-A :: CD11b
Comp-PerCP-Cy5-5-A :: F4_80
M1
0.90
Q2
0.040
Q4
97.7
M2
1.38
Comp-V450-A :: CD86
Comp-APC-A :: CD206
Blood_CD3-5_010.fcs
Ungated
7.44E5

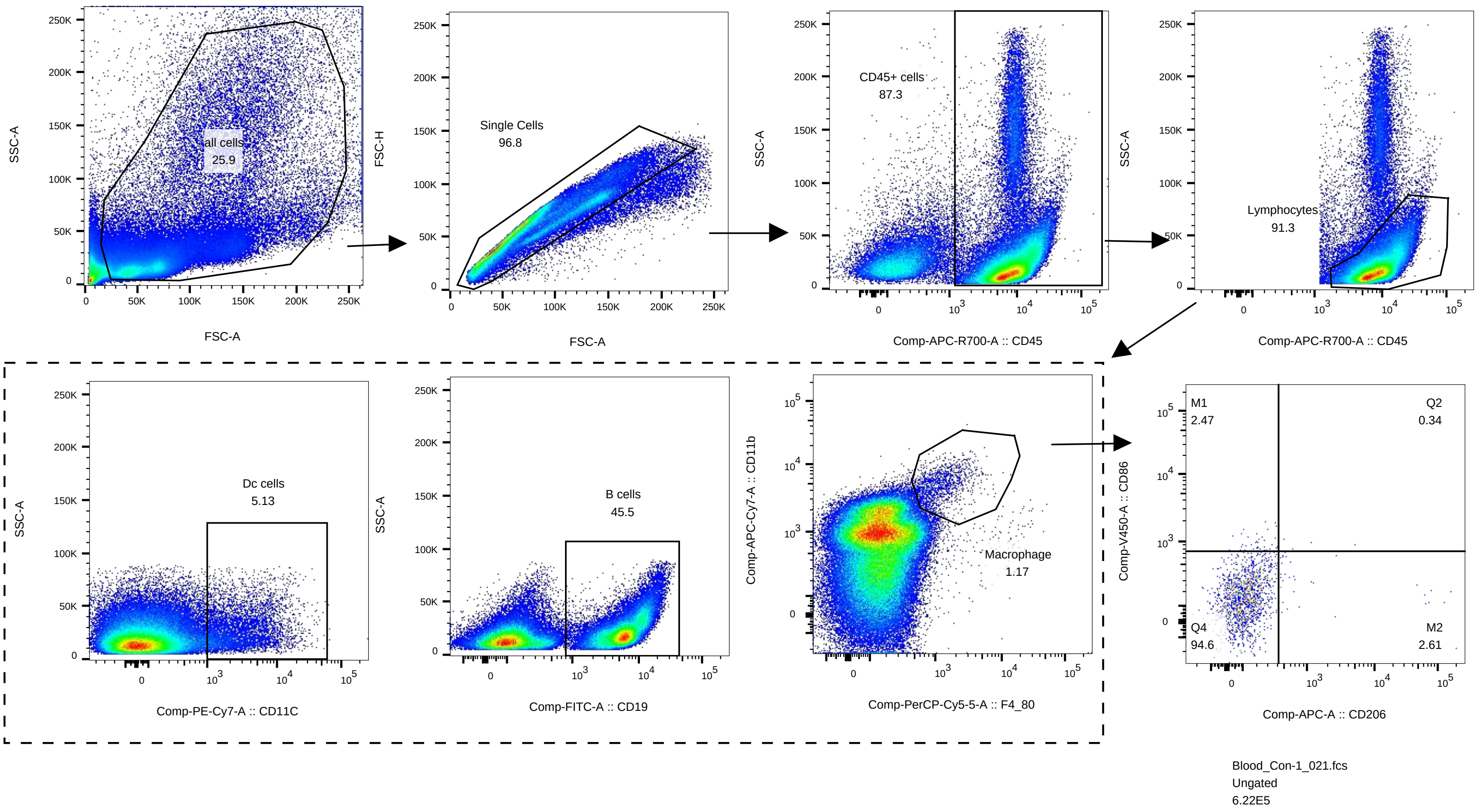
all cells
25.9
SSC-A
FSC-A
Single Cells
96.8
FSC-H
FSC-A
CD45+ cells
87.3
SSC-A
Comp-APC-R700-A :: CD45
Lymphocytes
91.3
SSC-A
Comp-APC-R700-A :: CD45
Dc cells
5.13
SSC-A
Comp-PE-Cy7-A :: CD11C
B cells
45.5
SSC-A
Comp-FITC-A :: CD19
Macrophage
1.17
Comp-APC-Cy7-A :: CD11b
Comp-PerCP-Cy5-5-A :: F4_80
M1
2.47
Q2
0.34
Q4
94.6
M2
2.61
Comp-V450-A :: CD86
Comp-APC-A :: CD206
Blood_Con-1_021.fcs
Ungated
6.22E5

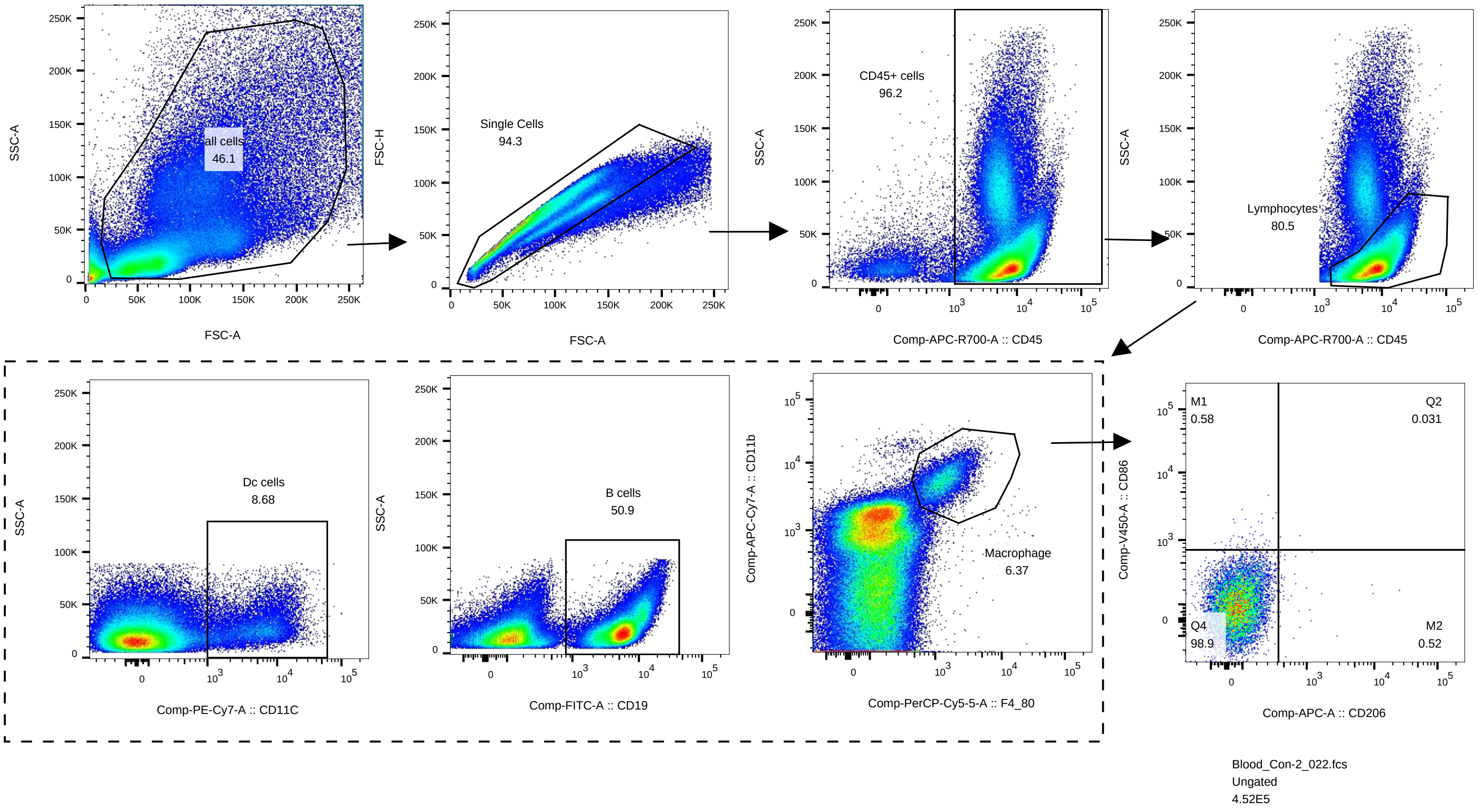
all cells
46.1
SSC-A
FSC-A
Single Cells
94.3
FSC-H
CD45+ cells
96.2
Comp-APC-R700-A :: CD45
Lymphocytes
80.5
Dc cells
8.68
Comp-PE-Cy7-A :: CD11C
B cells
50.9
Comp-FITC-A :: CD19
Macrophage
6.37
Comp-APC-Cy7-A :: CD11b
Comp-PerCP-Cy5-5-A :: F4_80
M1
0.58
Q2
0.031
Q4
98.9
M2
0.52
Comp-V450-A :: CD86
Comp-APC-A :: CD206
Blood_Con-2_022.fcs
Ungated
4.52E5

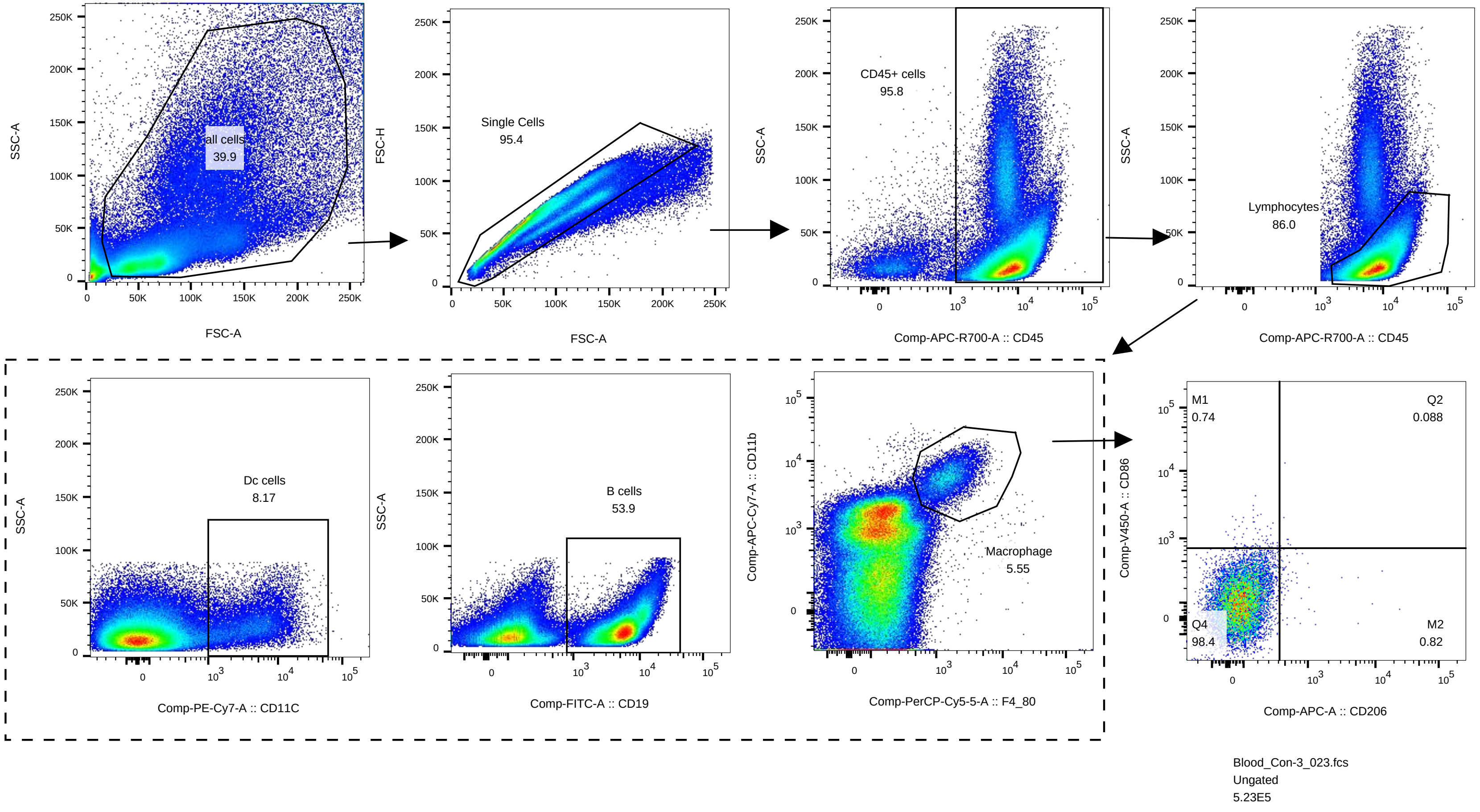

all cells
39.9
SSC-A
FSC-A
Single Cells
95.4
FSC-H
FSC-A
CD45+ cells
95.8
SSC-A
Comp-APC-R700-A :: CD45
Lymphocytes
86.0
SSC-A
Comp-APC-R700-A :: CD45
Dc cells
8.17
SSC-A
Comp-PE-Cy7-A :: CD11C
B cells
53.9
SSC-A
Comp-FITC-A :: CD19
Macrophage
5.55
Comp-APC-Cy7-A :: CD11b
Comp-PerCP-Cy5-5-A :: F4_80
M1
0.74
Q2
0.088
Q4
98.4
M2
0.82
Comp-V450-A :: CD86
Comp-APC-A :: CD206
Blood_Con-3_023.fcs
Ungated
5.23E5

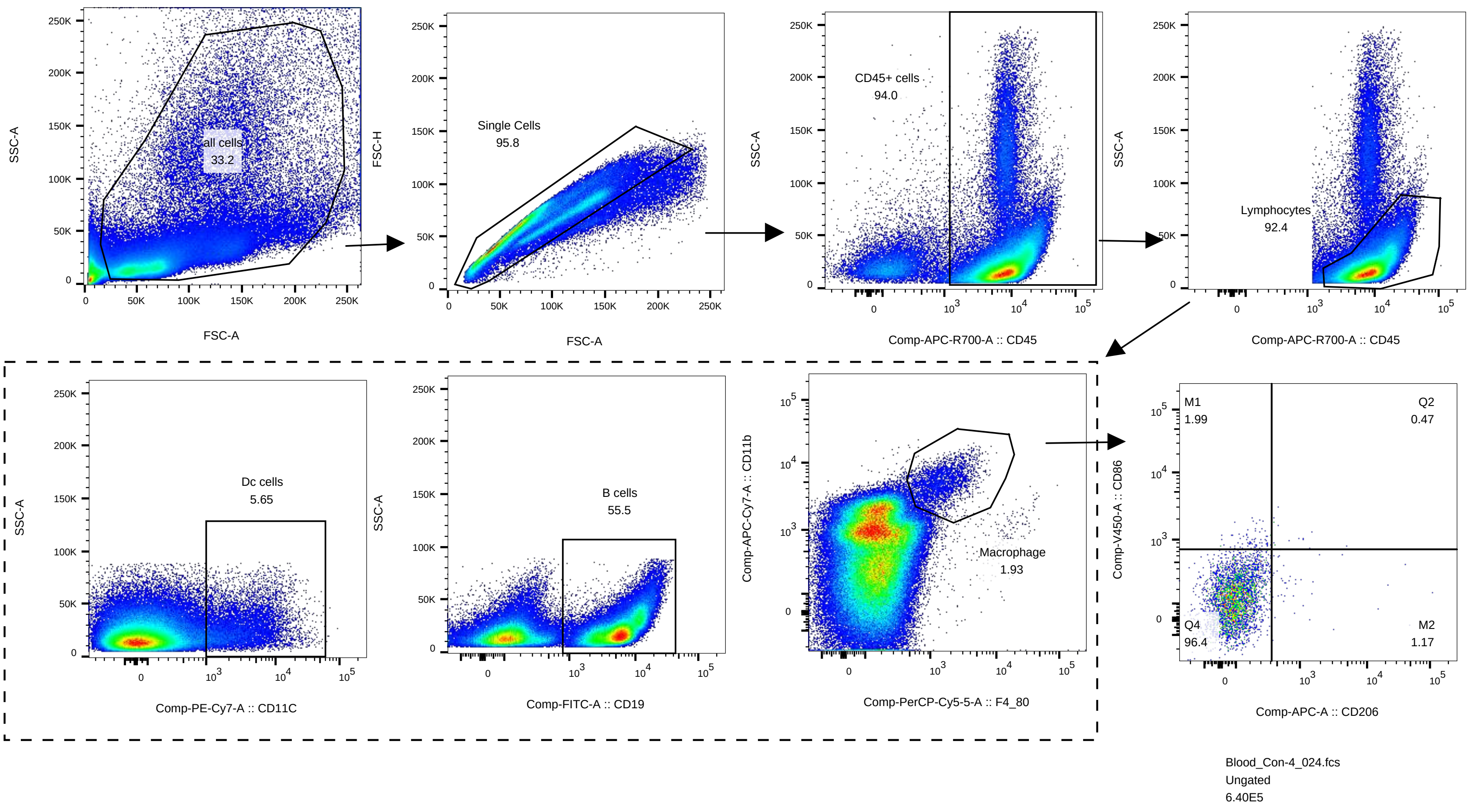
SSC-A
FSC-A
all cells
33.2
FSC-H
Single Cells
95.8
CD45+ cells
94.0
Comp-APC-R700-A :: CD45
Lymphocytes
92.4
Dc cells
5.65
Comp-PE-Cy7-A :: CD11C
B cells
55.5
Comp-FITC-A :: CD19
Comp-APC-Cy7-A :: CD11b
Macrophage
1.93
Comp-PerCP-Cy5-5-A :: F4_80
Comp-V450-A :: CD86
M1
1.99
Q2
0.47
Q4
96.4
M2
1.17
Comp-APC-A :: CD206
Blood_Con-4_024.fcs
Ungated
6.40E5

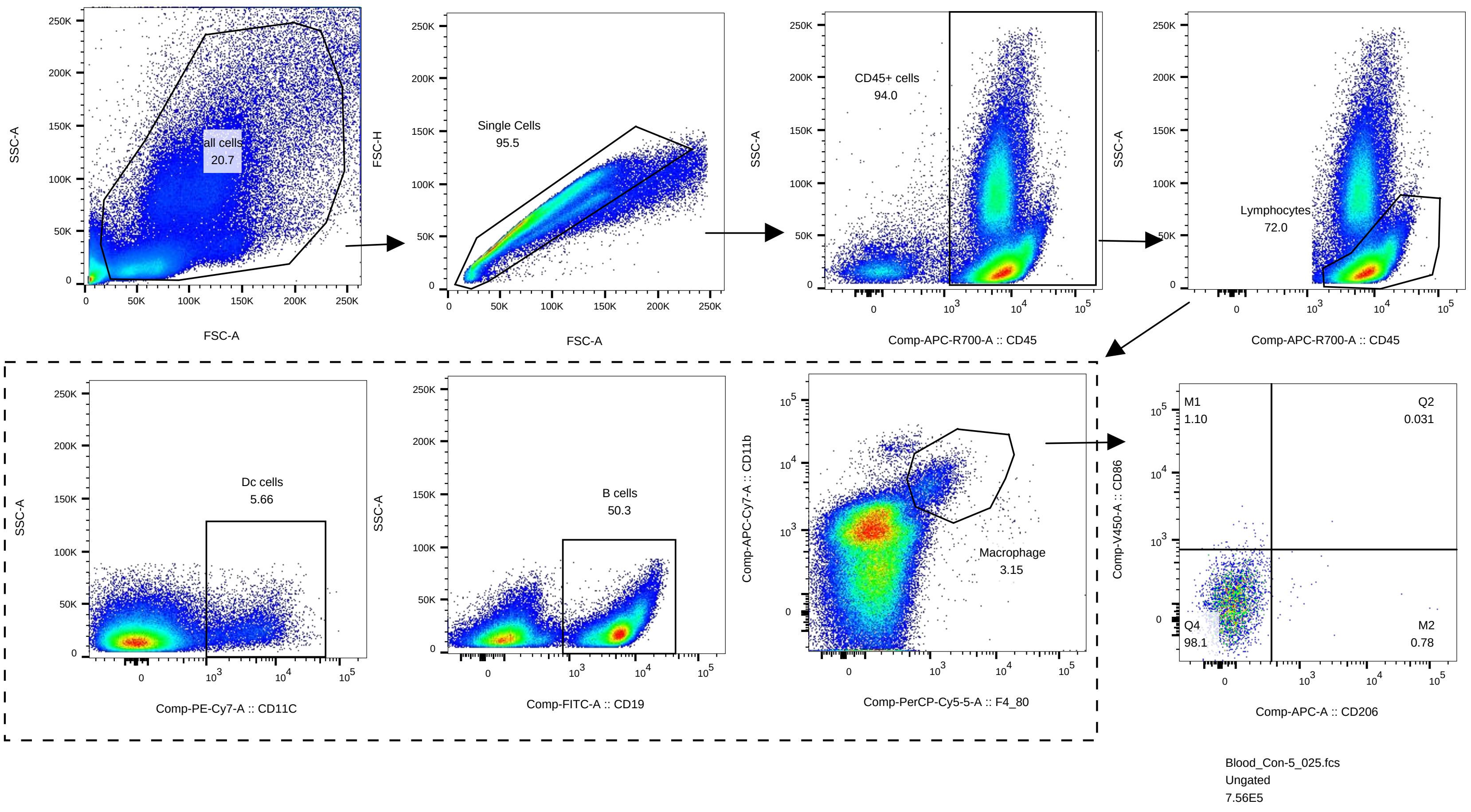
all cells
20.7
SSC-A
FSC-A
Single Cells
95.5
FSC-H
FSC-A
CD45+ cells
94.0
SSC-A
Comp-APC-R700-A :: CD45
Lymphocytes
72.0
SSC-A
Comp-APC-R700-A :: CD45
Dc cells
5.66
SSC-A
Comp-PE-Cy7-A :: CD11C
B cells
50.3
SSC-A
Comp-FITC-A :: CD19
Macrophage
3.15
Comp-APC-Cy7-A :: CD11b
Comp-PerCP-Cy5-5-A :: F4_80
M1
1.10
Q2
0.031
Q4
98.1
M2
0.78
Comp-V450-A :: CD86
Comp-APC-A :: CD206
Blood_Con-5_025.fcs
Ungated
7.56E5

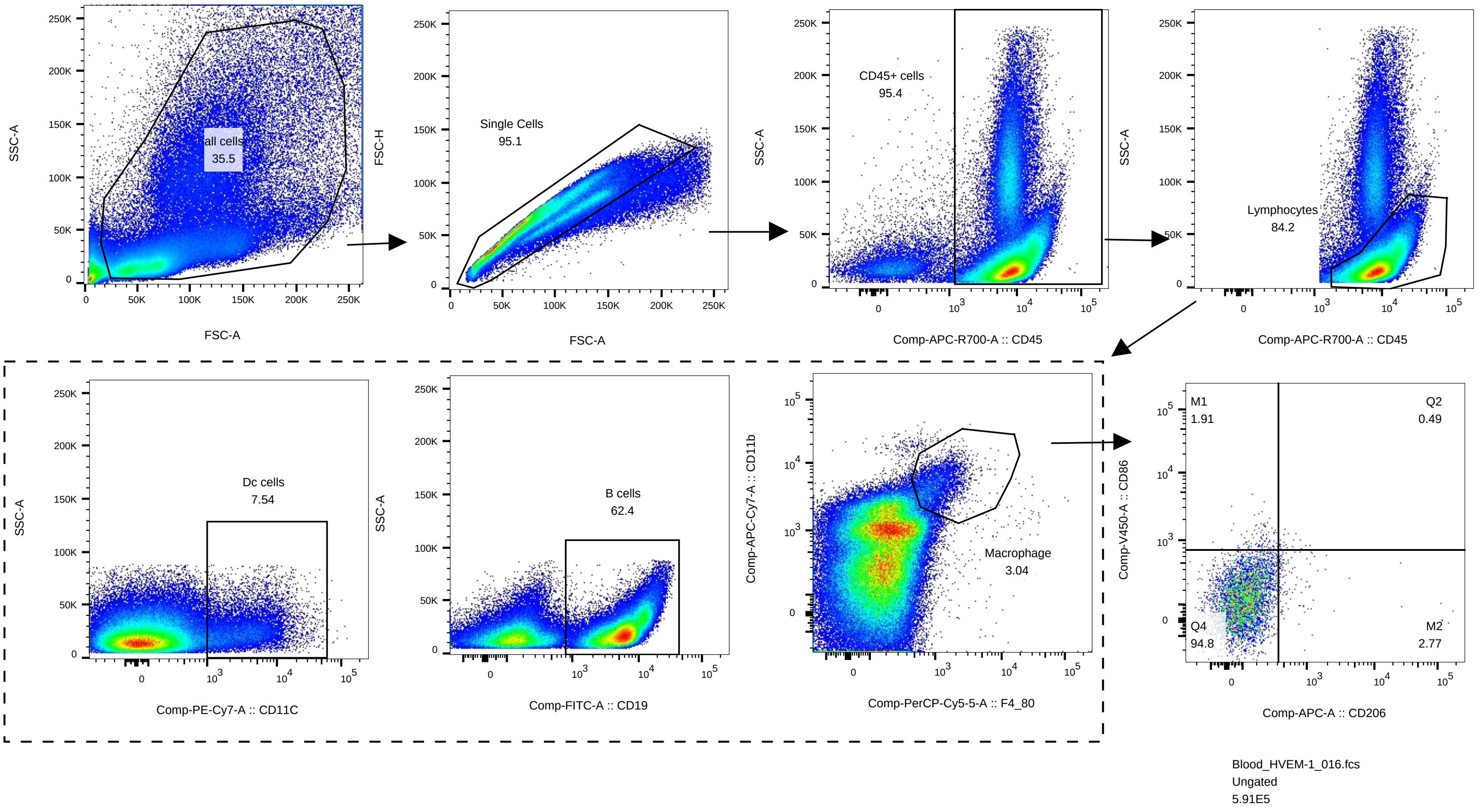
all cells
35.5
Single Cells
95.1
CD45+ cells
95.4
Lymphocytes
84.2
Dc cells
7.54
B cells
62.4
Macrophage
3.04
M1
1.91
Q2
0.49
Q4
94.8
M2
2.77
SSC-A
FSC-A
FSC-H
Comp-APC-R700-A :: CD45
Comp-PE-Cy7-A :: CD11C
Comp-FITC-A :: CD19
Comp-APC-Cy7-A :: CD11b
Comp-PerCP-Cy5-5-A :: F4_80
Comp-V450-A :: CD86
Comp-APC-A :: CD206
Blood_HVEM-1_016.fcs
Ungated
5.91E5

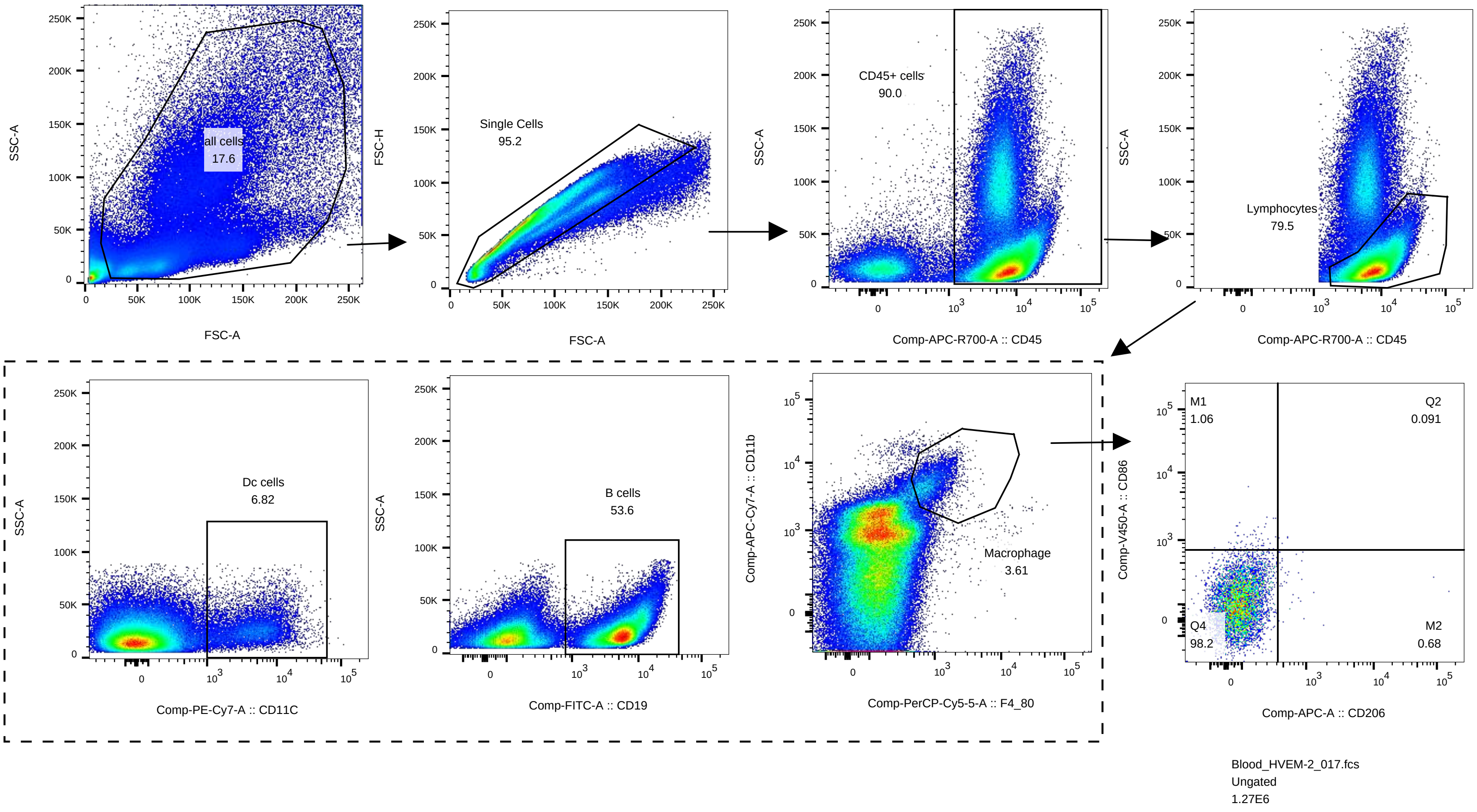
all cells
17.6
Single Cells
95.2
CD45+ cells
90.0
Lymphocytes
79.5
Dc cells
6.82
B cells
53.6
Macrophage
3.61
M1
1.06
Q2
0.091
Q4
98.2
M2
0.68
SSC-A
FSC-A
FSC-H
Comp-APC-R700-A :: CD45
Comp-PE-Cy7-A :: CD11C
Comp-FITC-A :: CD19
Comp-APC-Cy7-A :: CD11b
Comp-PerCP-Cy5-5-A :: F4_80
Comp-V450-A :: CD86
Comp-APC-A :: CD206
Blood_HVEM-2_017.fcs
Ungated
1.27E6

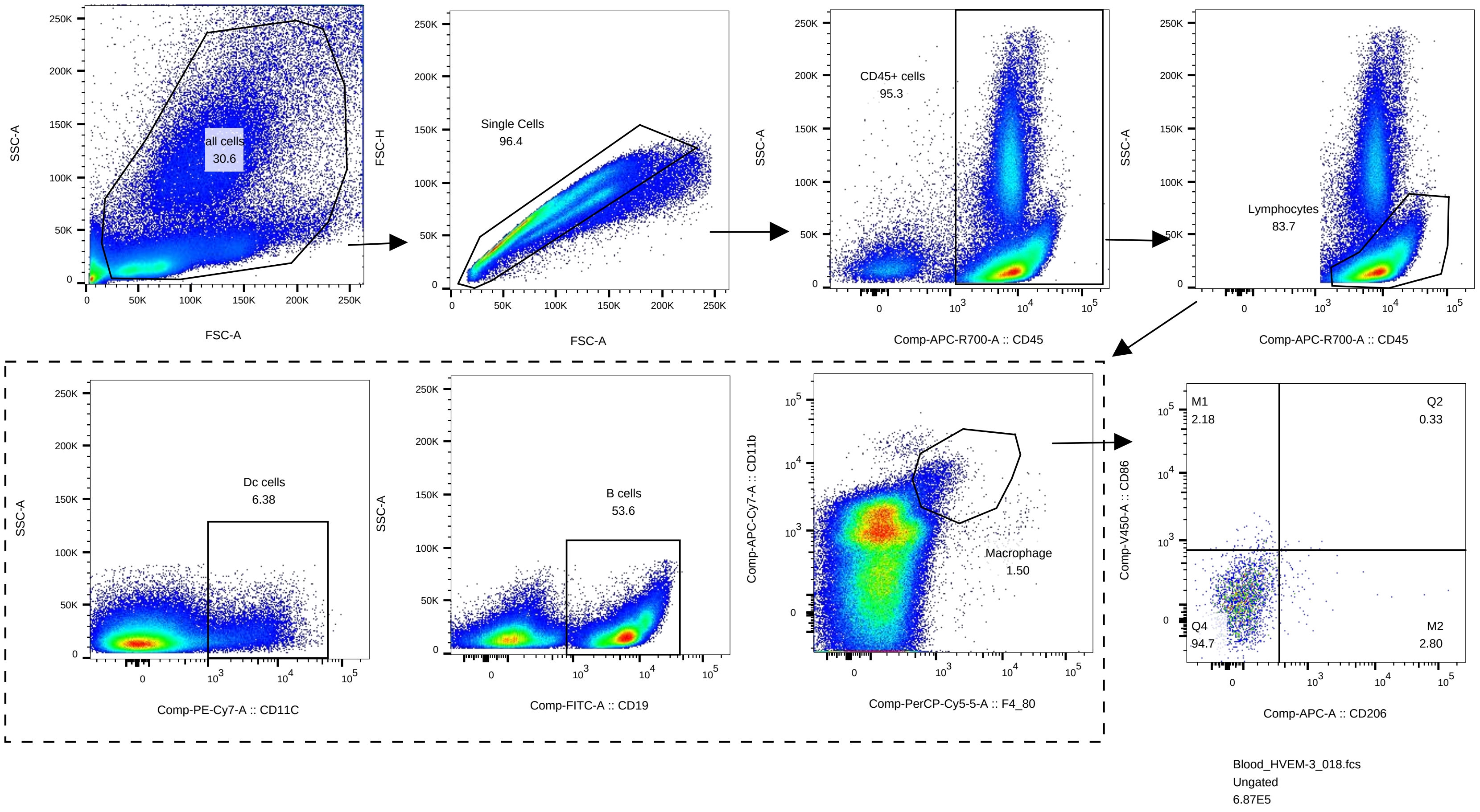

all cells
30.6
Single Cells
96.4
CD45+ cells
95.3
Lymphocytes
83.7
Dc cells
6.38
B cells
53.6
Macrophage
1.50
M1
2.18
Q2
0.33
Q4
94.7
M2
2.80
SSC-A
FSC-A
FSC-H
Comp-APC-R700-A :: CD45
Comp-PE-Cy7-A :: CD11C
Comp-FITC-A :: CD19
Comp-APC-Cy7-A :: CD11b
Comp-PerCP-Cy5-5-A :: F4_80
Comp-V450-A :: CD86
Comp-APC-A :: CD206
Blood_HVEM-3_018.fcs
Ungated
6.87E5

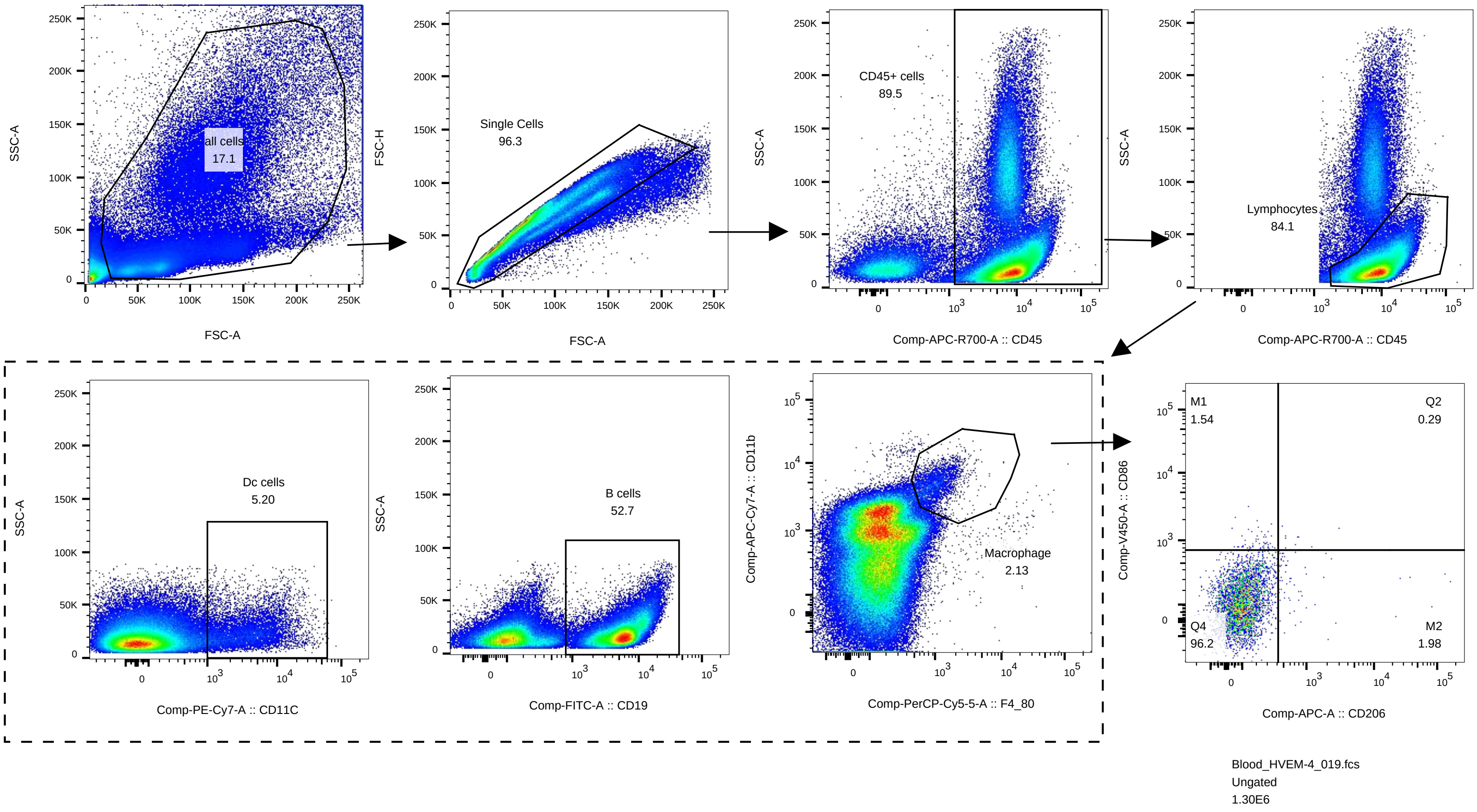
all cells
17.1
SSC-A
FSC-A
Single Cells
96.3
FSC-H
FSC-A
CD45+ cells
89.5
SSC-A
Comp-APC-R700-A :: CD45
Lymphocytes
84.1
SSC-A
Comp-APC-R700-A :: CD45
Dc cells
5.20
SSC-A
Comp-PE-Cy7-A :: CD11C
B cells
52.7
SSC-A
Comp-FITC-A :: CD19
Macrophage
2.13
Comp-APC-Cy7-A :: CD11b
Comp-PerCP-Cy5-5-A :: F4_80
M1
1.54
Q2
0.29
Q4
96.2
M2
1.98
Comp-V450-A :: CD86
Comp-APC-A :: CD206
Blood_HVEM-4_019.fcs
Ungated
1.30E6

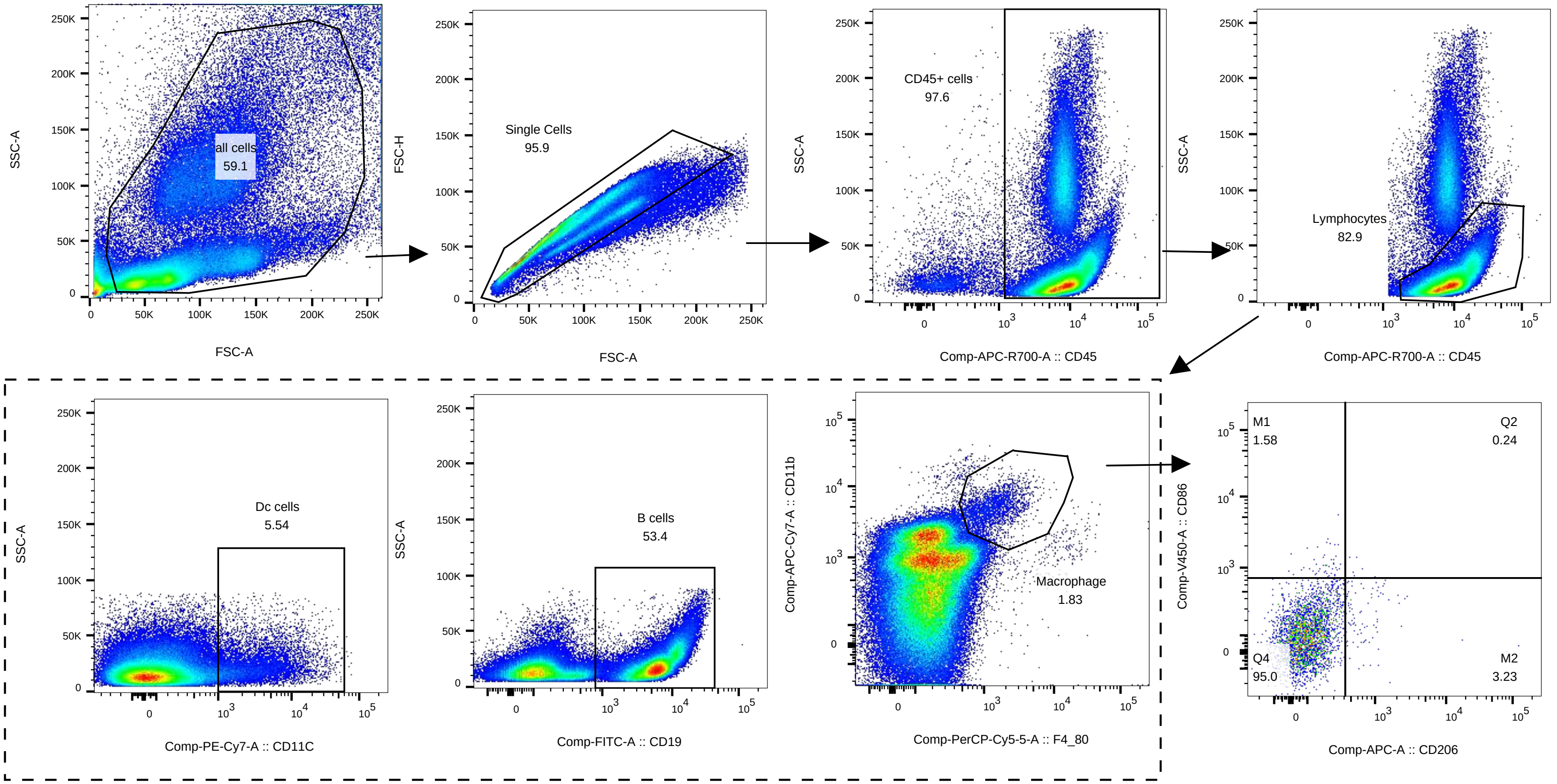

Blood_HVEM-5_020.fcs
Ungated
346815

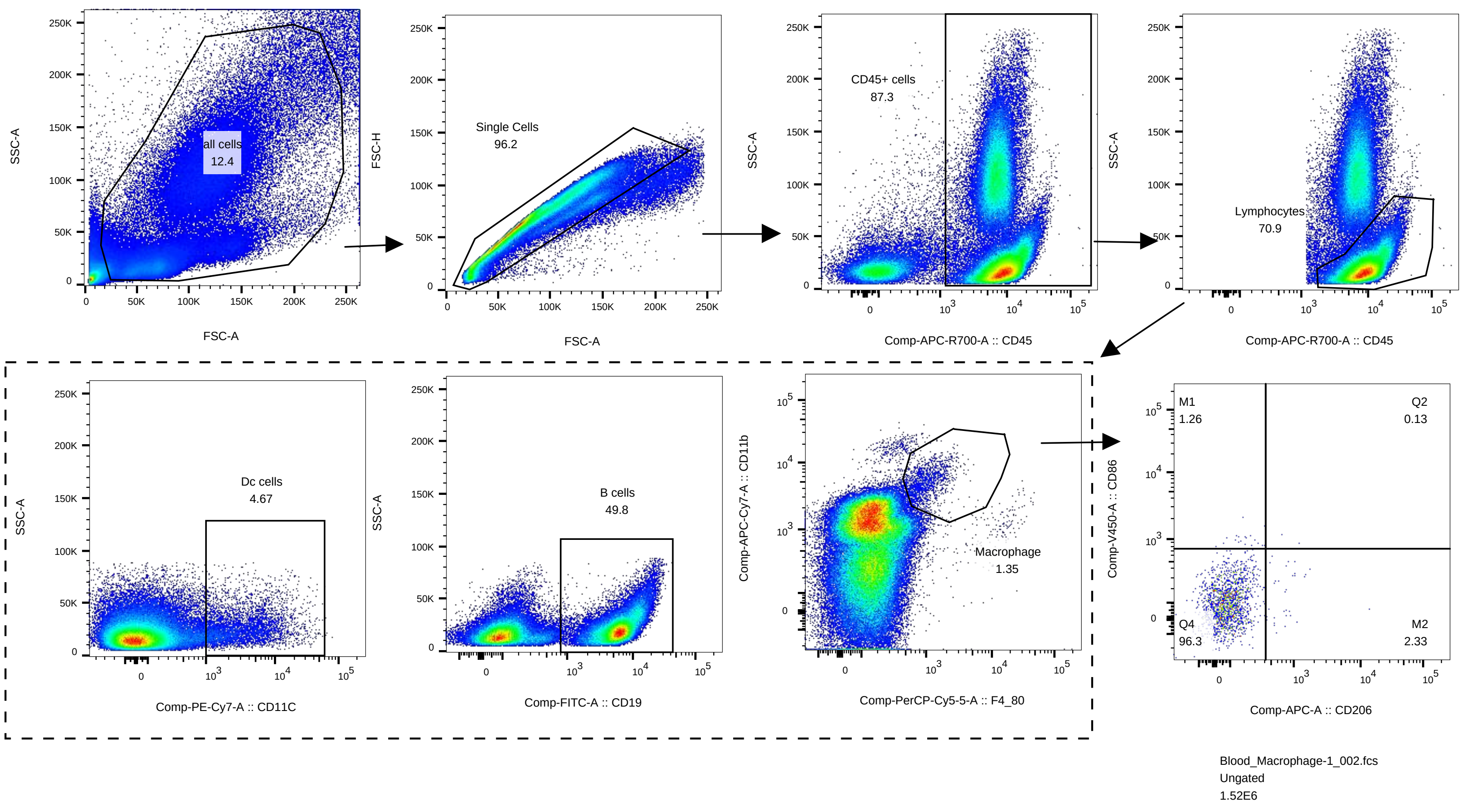
all cells
12.4
SSC-A
FSC-A
Single Cells
96.2
FSC-H
FSC-A
CD45+ cells
87.3
SSC-A
Comp-APC-R700-A :: CD45
Lymphocytes
70.9
SSC-A
Comp-APC-R700-A :: CD45
Dc cells
4.67
SSC-A
Comp-PE-Cy7-A :: CD11C
B cells
49.8
SSC-A
Comp-FITC-A :: CD19
Macrophage
1.35
Comp-APC-Cy7-A :: CD11b
Comp-PerCP-Cy5-5-A :: F4_80
M1
1.26
Q2
0.13
Q4
96.3
M2
2.33
Comp-V450-A :: CD86
Comp-APC-A :: CD206
Blood_Macrophage-1_002.fcs
Ungated
1.52E6

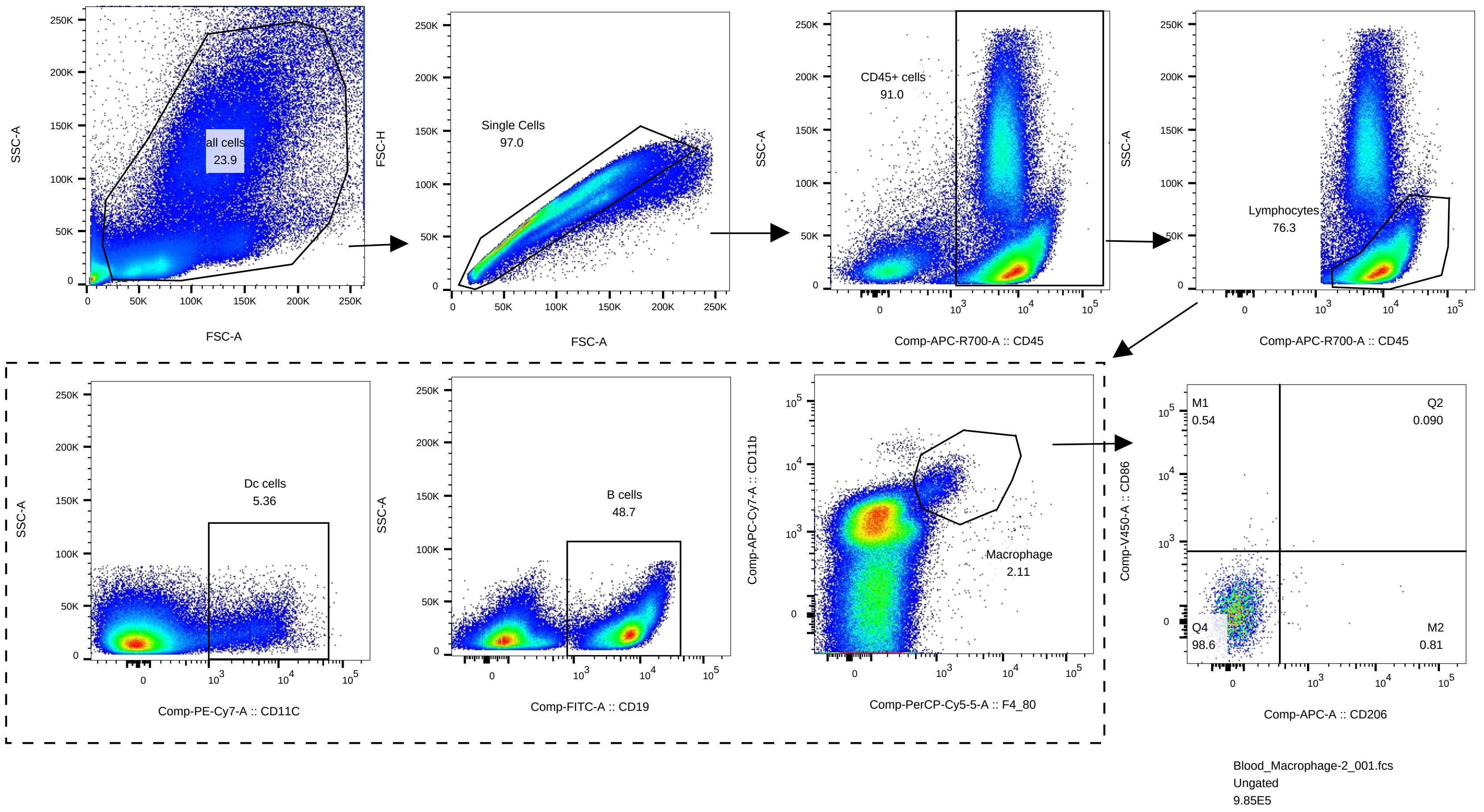

all cells
23.9
SSC-A
FSC-A
Single Cells
97.0
FSC-H
FSC-A
CD45+ cells
91.0
SSC-A
Comp-APC-R700-A :: CD45
Lymphocytes
76.3
SSC-A
Comp-APC-R700-A :: CD45
Dc cells
5.36
SSC-A
Comp-PE-Cy7-A :: CD11C
B cells
48.7
SSC-A
Comp-FITC-A :: CD19
Macrophage
2.11
Comp-APC-Cy7-A :: CD11b
Comp-PerCP-Cy5-5-A :: F4_80
M1
0.54
Q2
0.090
Q4
98.6
M2
0.81
Comp-V450-A :: CD86
Comp-APC-A :: CD206
Blood_Macrophage-2_001.fcs
Ungated
9.85E5

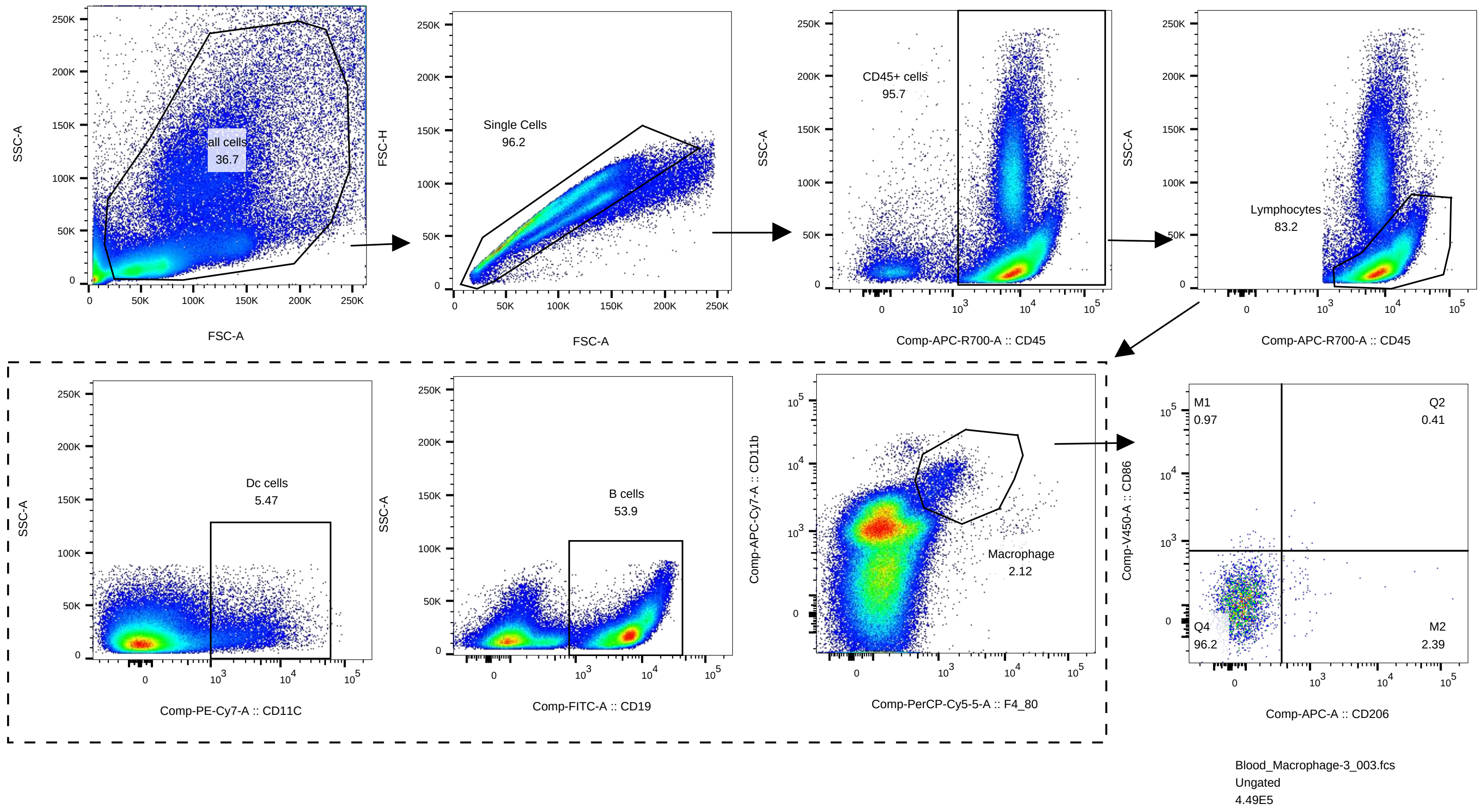
all cells
36.7
Single Cells
96.2
CD45+ cells
95.7
Lymphocytes
83.2
Dc cells
5.47
B cells
53.9
Macrophage
2.12
M1
0.97
Q2
0.41
Q4
96.2
M2
2.39
SSC-A
FSC-A
FSC-H
Comp-APC-R700-A :: CD45
Comp-PE-Cy7-A :: CD11C
Comp-FITC-A :: CD19
Comp-APC-Cy7-A :: CD11b
Comp-PerCP-Cy5-5-A :: F4_80
Comp-V450-A :: CD86
Comp-APC-A :: CD206
Blood_Macrophage-3_003.fcs
Ungated
4.49E5

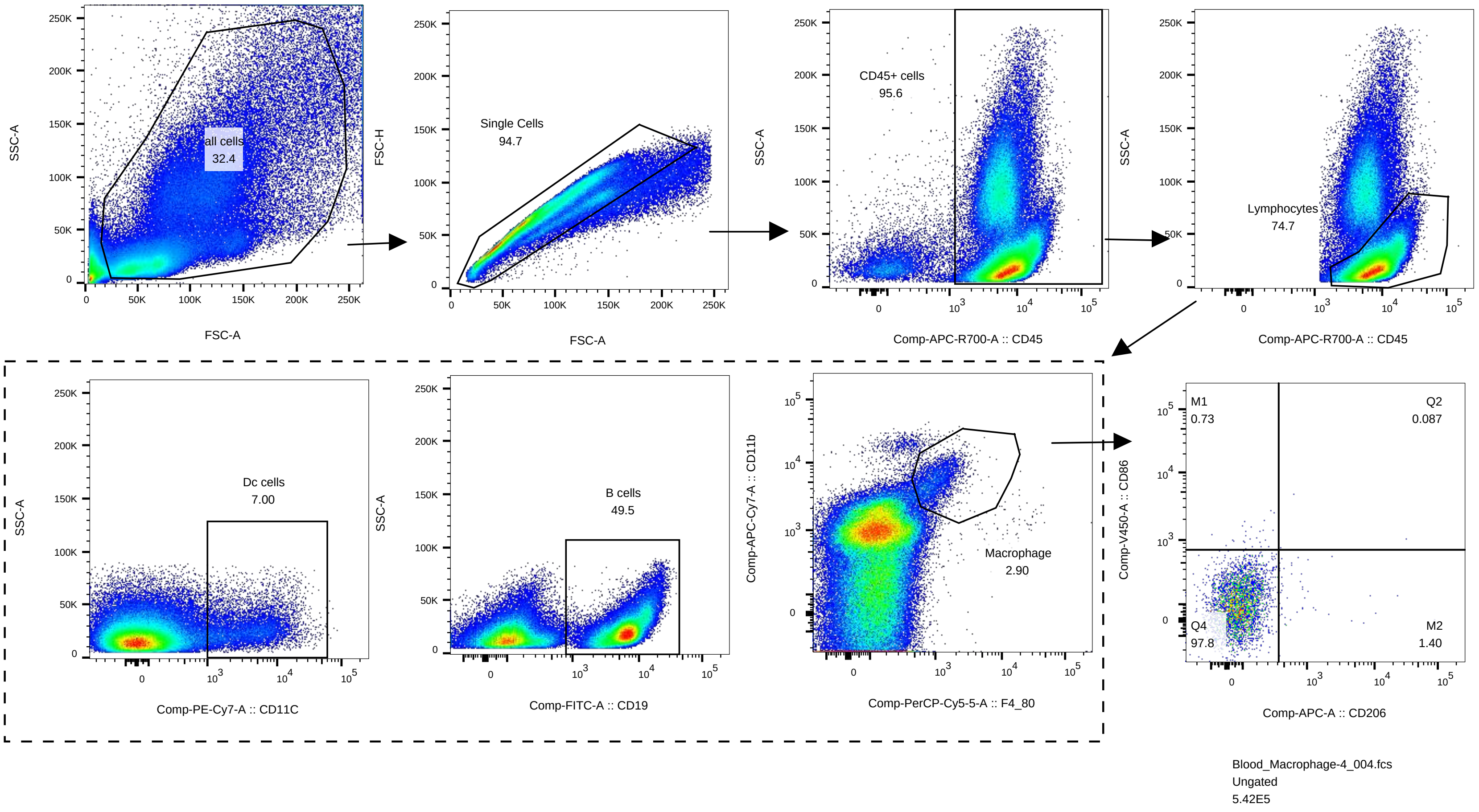
all cells
32.4
SSC-A
FSC-A
Single Cells
94.7
FSC-H
FSC-A
CD45+ cells
95.6
SSC-A
Comp-APC-R700-A :: CD45
Lymphocytes
74.7
SSC-A
Comp-APC-R700-A :: CD45
Dc cells
7.00
SSC-A
Comp-PE-Cy7-A :: CD11C
B cells
49.5
SSC-A
Comp-FITC-A :: CD19
Macrophage
2.90
Comp-APC-Cy7-A :: CD11b
Comp-PerCP-Cy5-5-A :: F4_80
M1
0.73
Q2
0.087
Q4
97.8
M2
1.40
Comp-V450-A :: CD86
Comp-APC-A :: CD206
Blood_Macrophage-4_004.fcs
Ungated
5.42E5

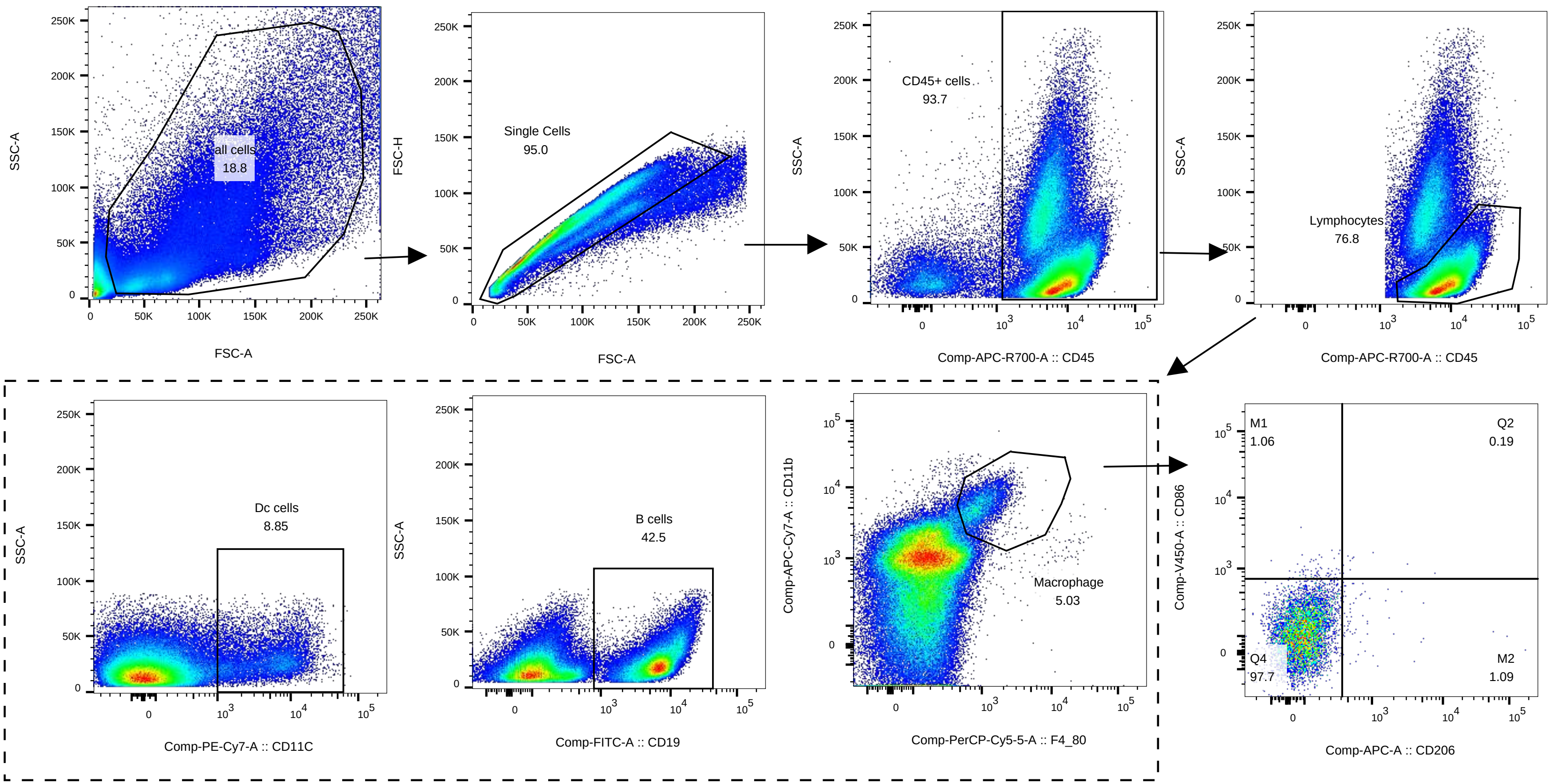

Blood_Macrophage-5_005.fcs
Ungated
9.04E5

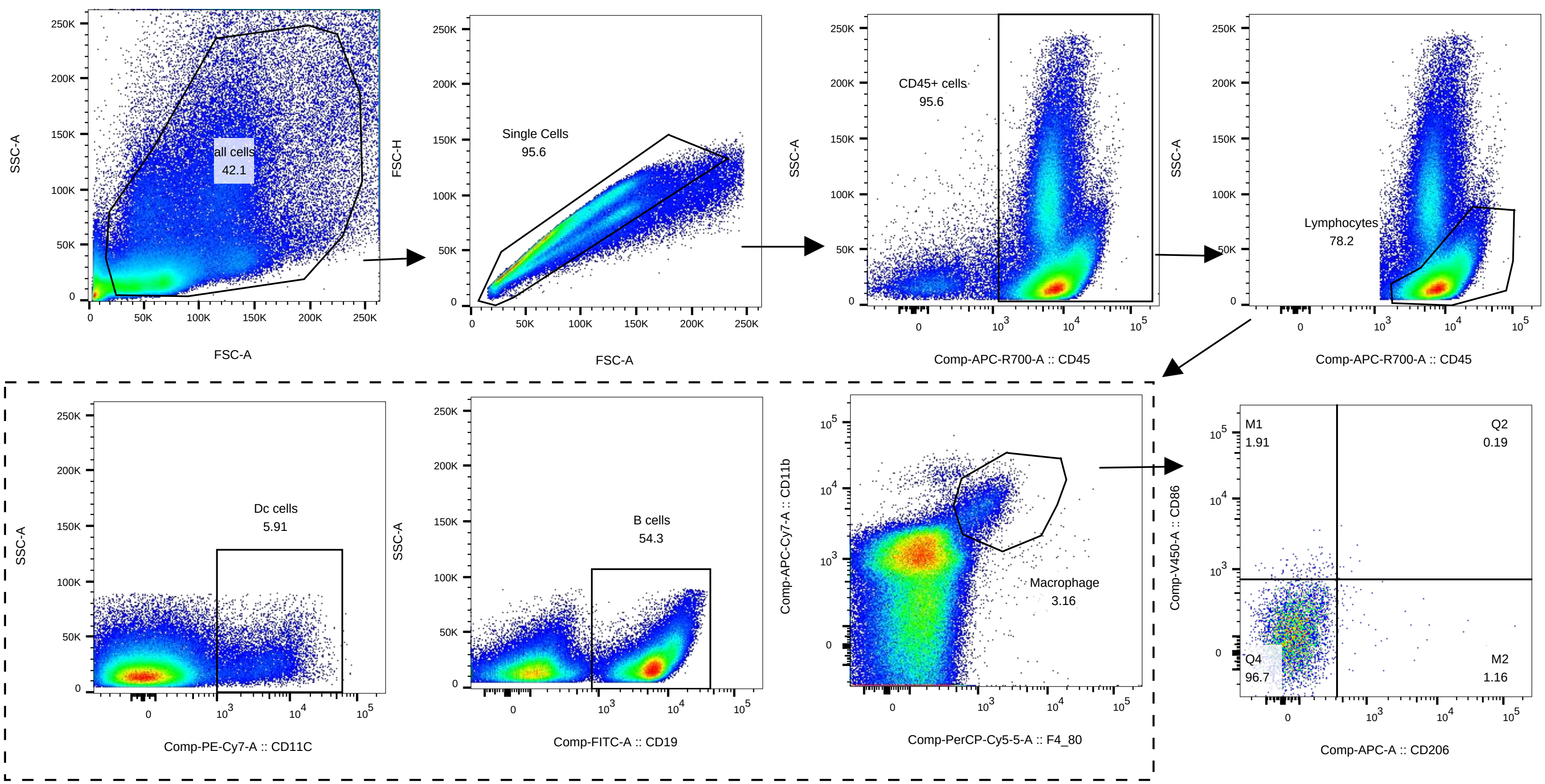

Blood_NK-1_011.fcs
Ungated
4.91E5

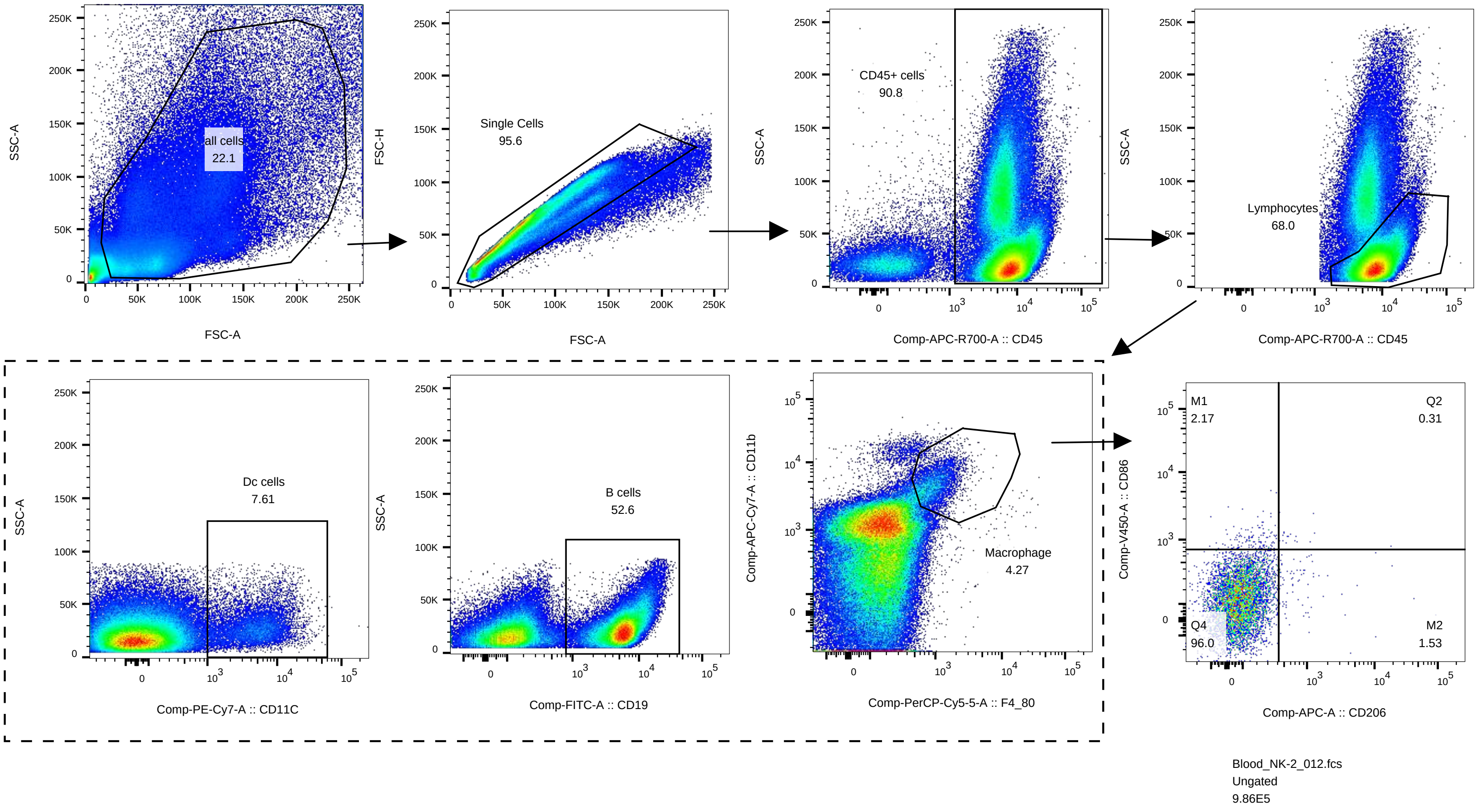
all cells
22.1
SSC-A
FSC-A
Single Cells
95.6
FSC-H
CD45+ cells
90.8
Comp-APC-R700-A :: CD45
Lymphocytes
68.0
Dc cells
7.61
Comp-PE-Cy7-A :: CD11C
B cells
52.6
Comp-FITC-A :: CD19
Macrophage
4.27
Comp-APC-Cy7-A :: CD11b
Comp-PerCP-Cy5-5-A :: F4_80
M1
2.17
Q2
0.31
Q4
96.0
M2
1.53
Comp-V450-A :: CD86
Comp-APC-A :: CD206
Blood_NK-2_012.fcs
Ungated
9.86E5

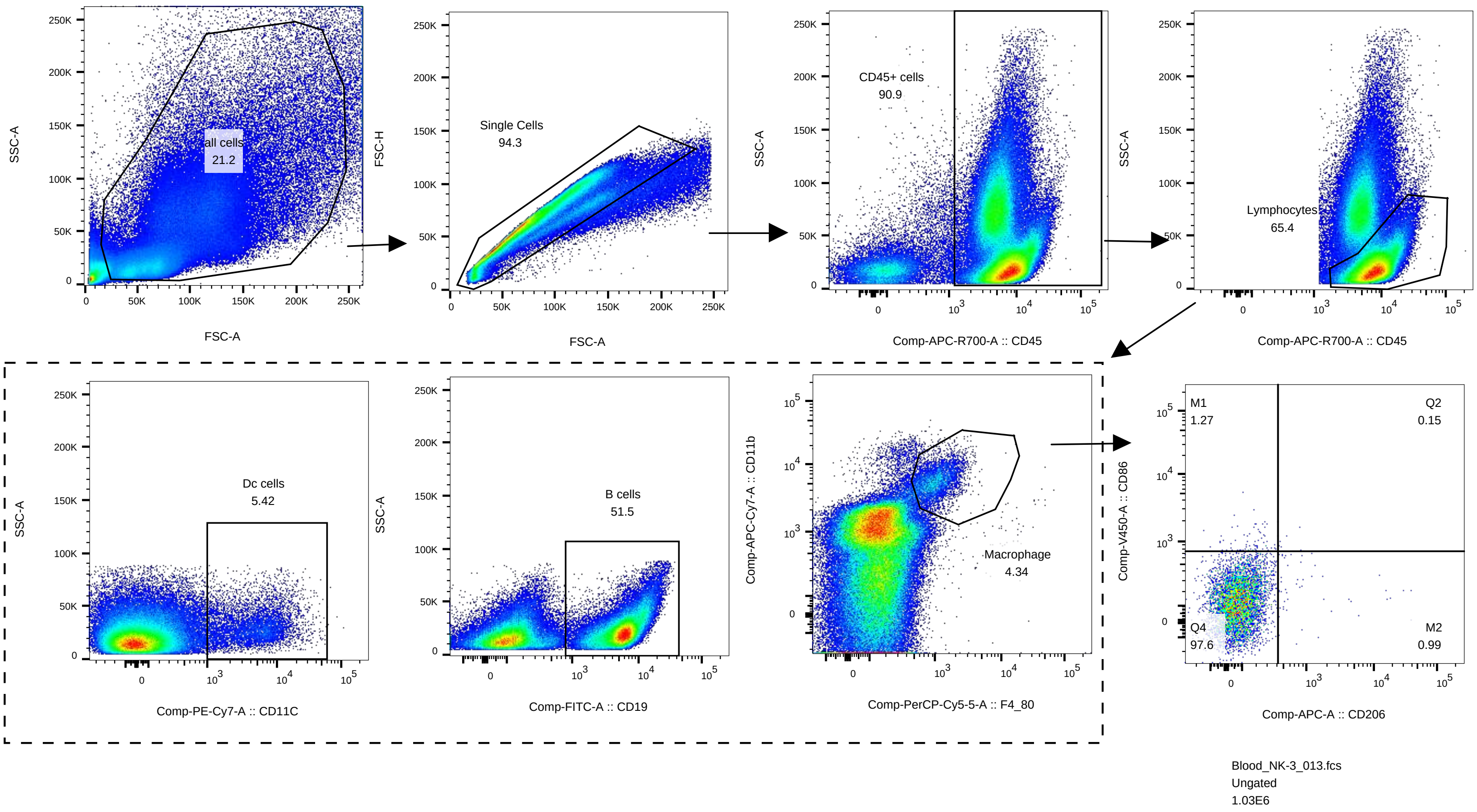

all cells
21.2
SSC-A
FSC-A
Single Cells
94.3
FSC-H
FSC-A
CD45+ cells
90.9
SSC-A
Comp-APC-R700-A :: CD45
Lymphocytes
65.4
SSC-A
Comp-APC-R700-A :: CD45
Dc cells
5.42
SSC-A
Comp-PE-Cy7-A :: CD11C
B cells
51.5
SSC-A
Comp-FITC-A :: CD19
Macrophage
4.34
Comp-APC-Cy7-A :: CD11b
Comp-PerCP-Cy5-5-A :: F4_80
M1
1.27
Q2
0.15
Q4
97.6
M2
0.99
Comp-V450-A :: CD86
Comp-APC-A :: CD206
Blood_NK-3_013.fcs
Ungated
1.03E6

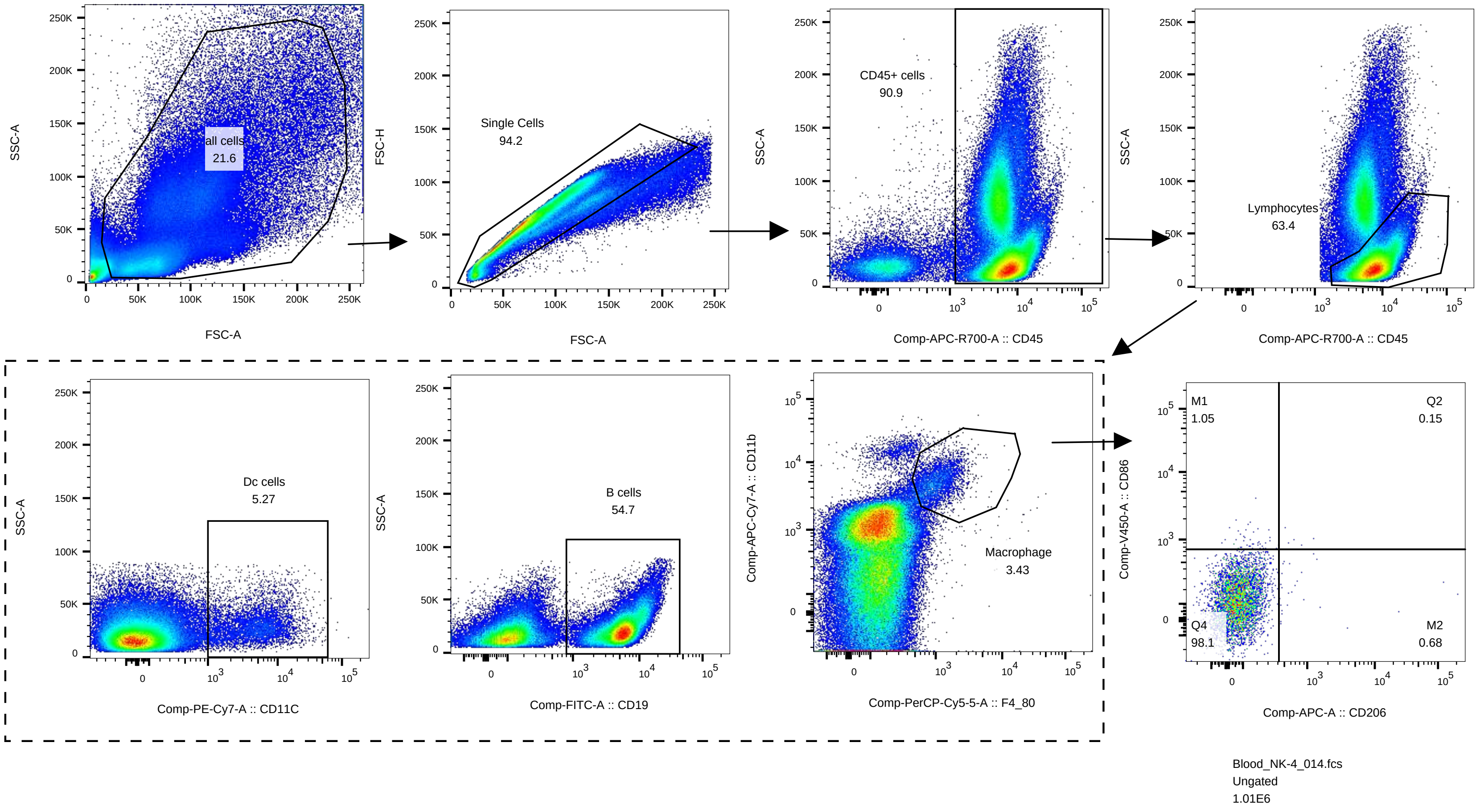

all cells
21.6
Single Cells
94.2
CD45+ cells
90.9
Lymphocytes
63.4
Dc cells
5.27
B cells
54.7
Macrophage
3.43
M1
1.05
Q2
0.15
Q4
98.1
M2
0.68
SSC-A
FSC-A
FSC-H
Comp-APC-R700-A :: CD45
Comp-PE-Cy7-A :: CD11C
Comp-FITC-A :: CD19
Comp-APC-Cy7-A :: CD11b
Comp-PerCP-Cy5-5-A :: F4_80
Comp-V450-A :: CD86
Comp-APC-A :: CD206
Blood_NK-4_014.fcs
Ungated
1.01E6

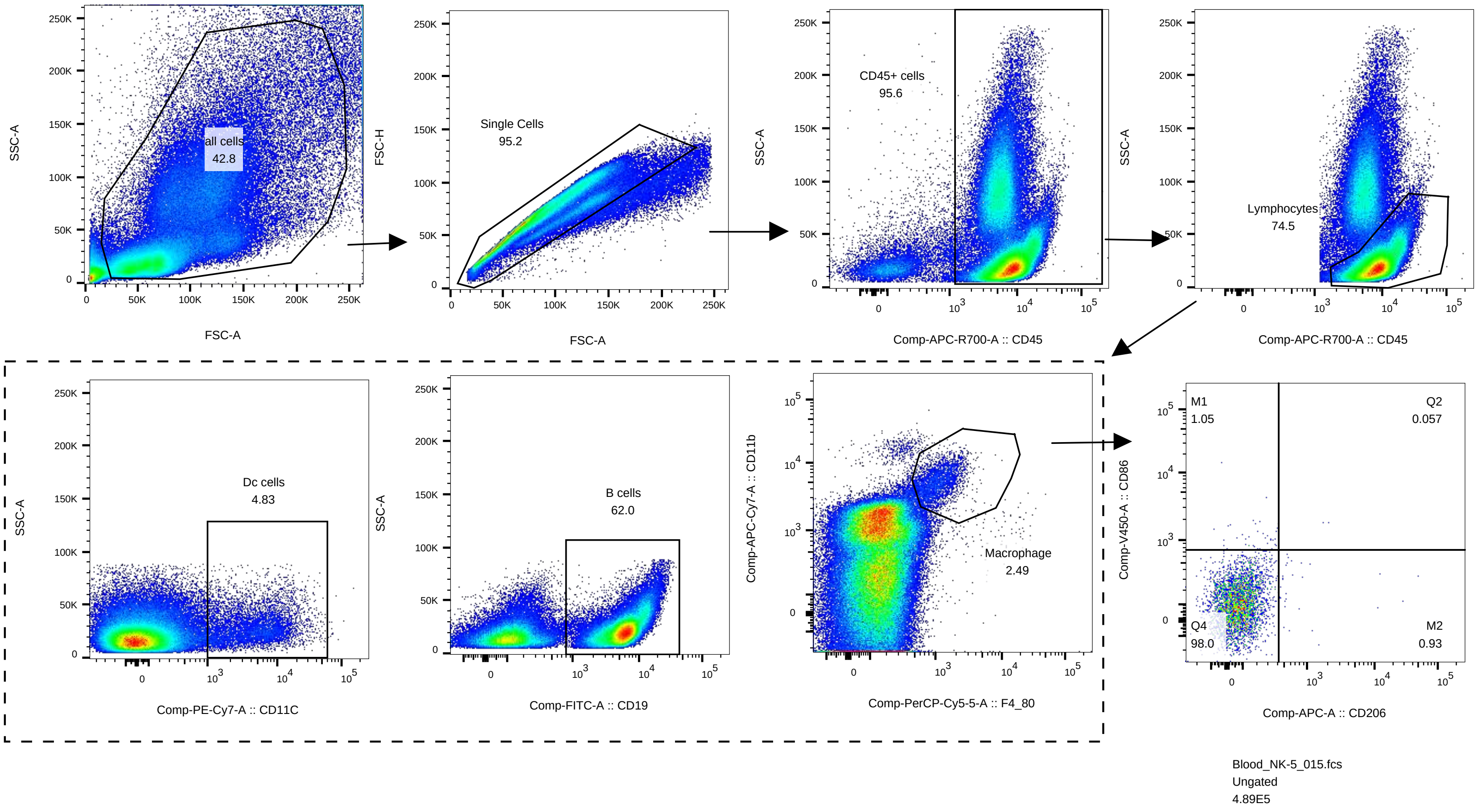
all cells
42.8
SSC-A
FSC-A
Single Cells
95.2
FSC-H
FSC-A
CD45+ cells
95.6
SSC-A
Comp-APC-R700-A :: CD45
Lymphocytes
74.5
SSC-A
Comp-APC-R700-A :: CD45
Dc cells
4.83
SSC-A
Comp-PE-Cy7-A :: CD11C
B cells
62.0
SSC-A
Comp-FITC-A :: CD19
Macrophage
2.49
Comp-APC-Cy7-A :: CD11b
Comp-PerCP-Cy5-5-A :: F4_80
M1
1.05
Q2
0.057
Q4
98.0
M2
0.93
Comp-V450-A :: CD86
Comp-APC-A :: CD206
Blood_NK-5_015.fcs
Ungated
4.89E5
