# Supplementary figures and images for: A novel HVEM-Fc recombinant protein for lung cancer immunotherapy
Source: J Exp Clin Cancer Res. 2025 Feb 20;44:62. doi: 10.1186/s13046-025-03324-8 (PMC11841141; doi:10.1186/s13046-025-03324-8)

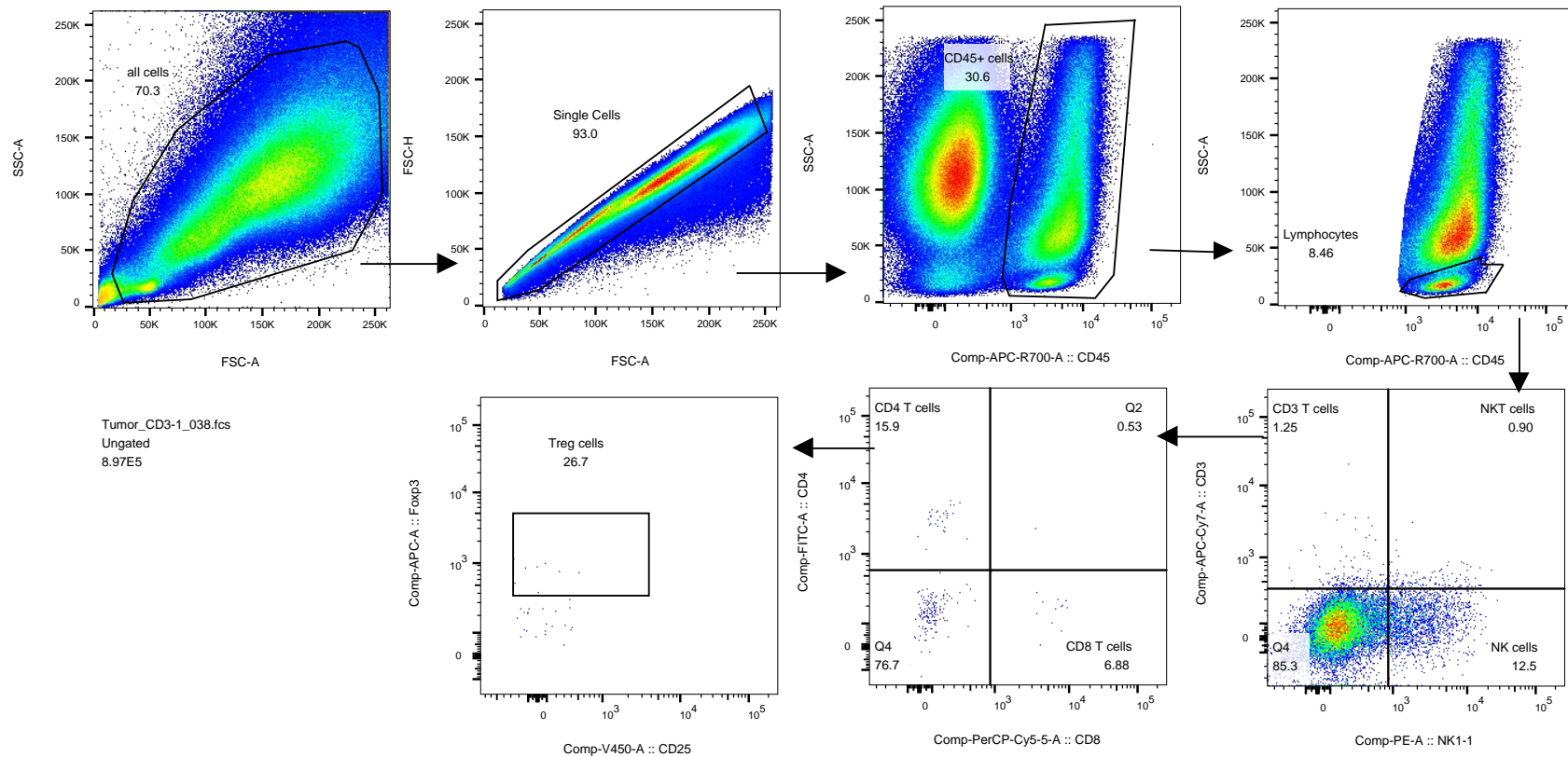

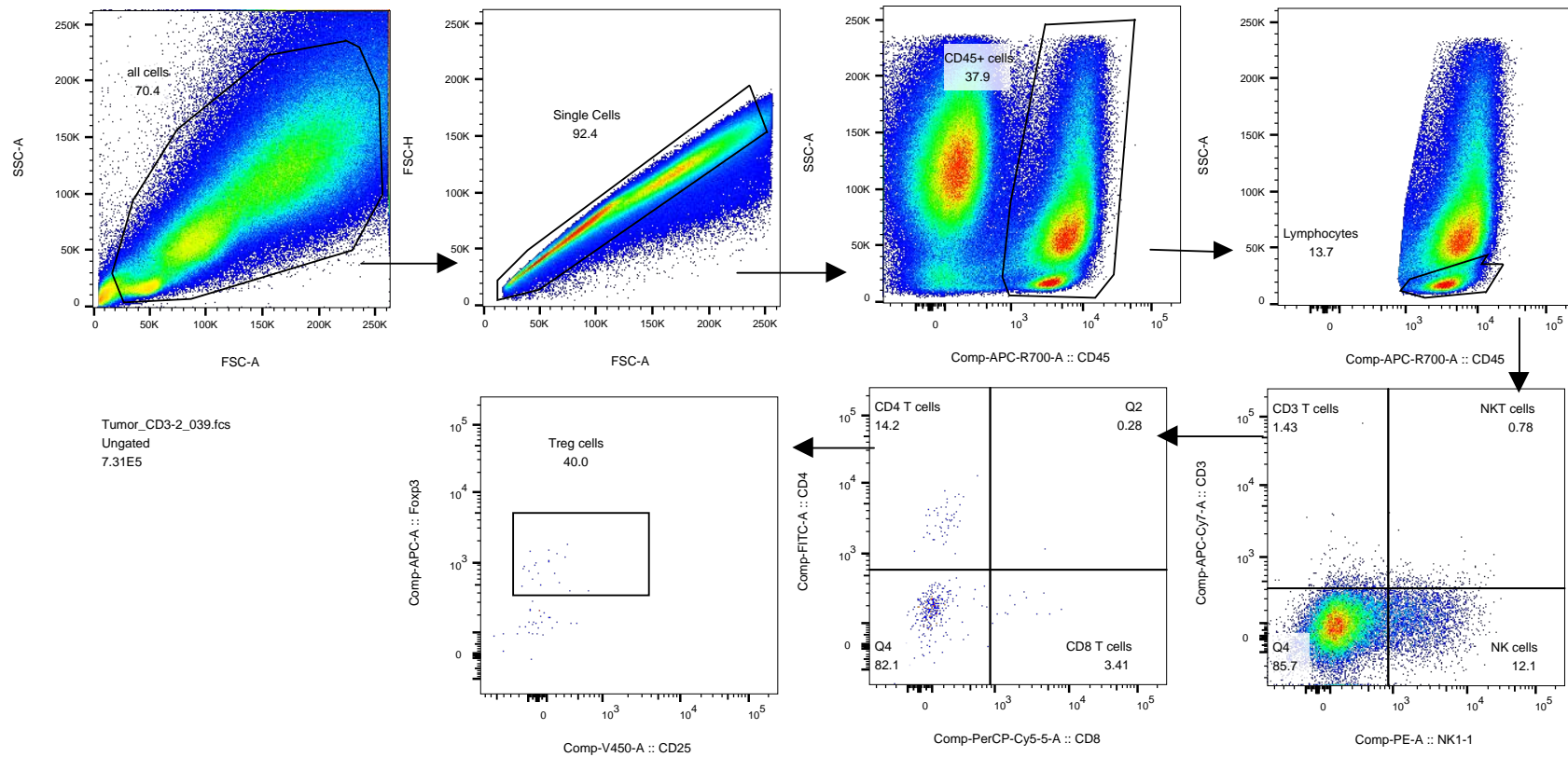

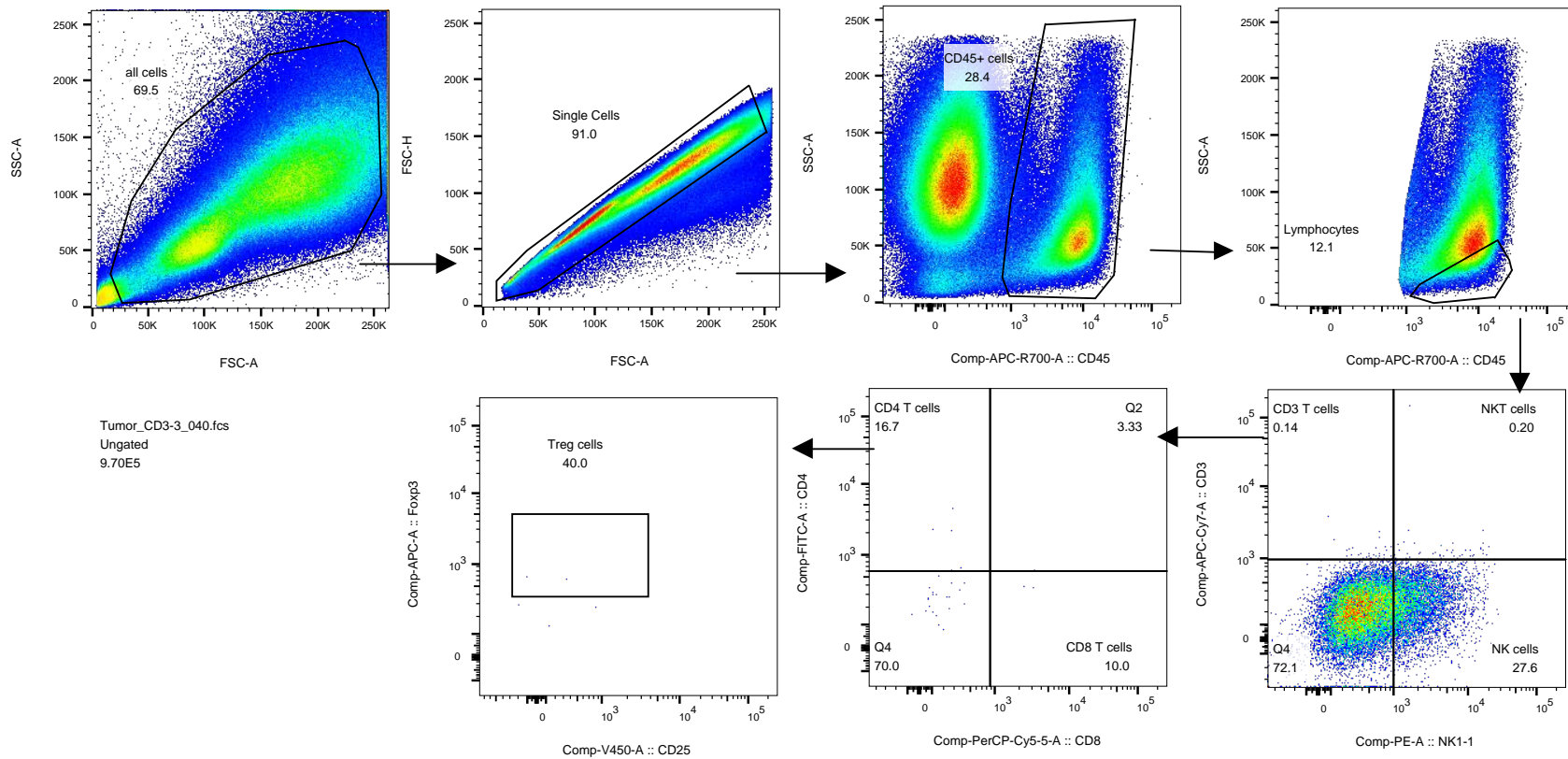

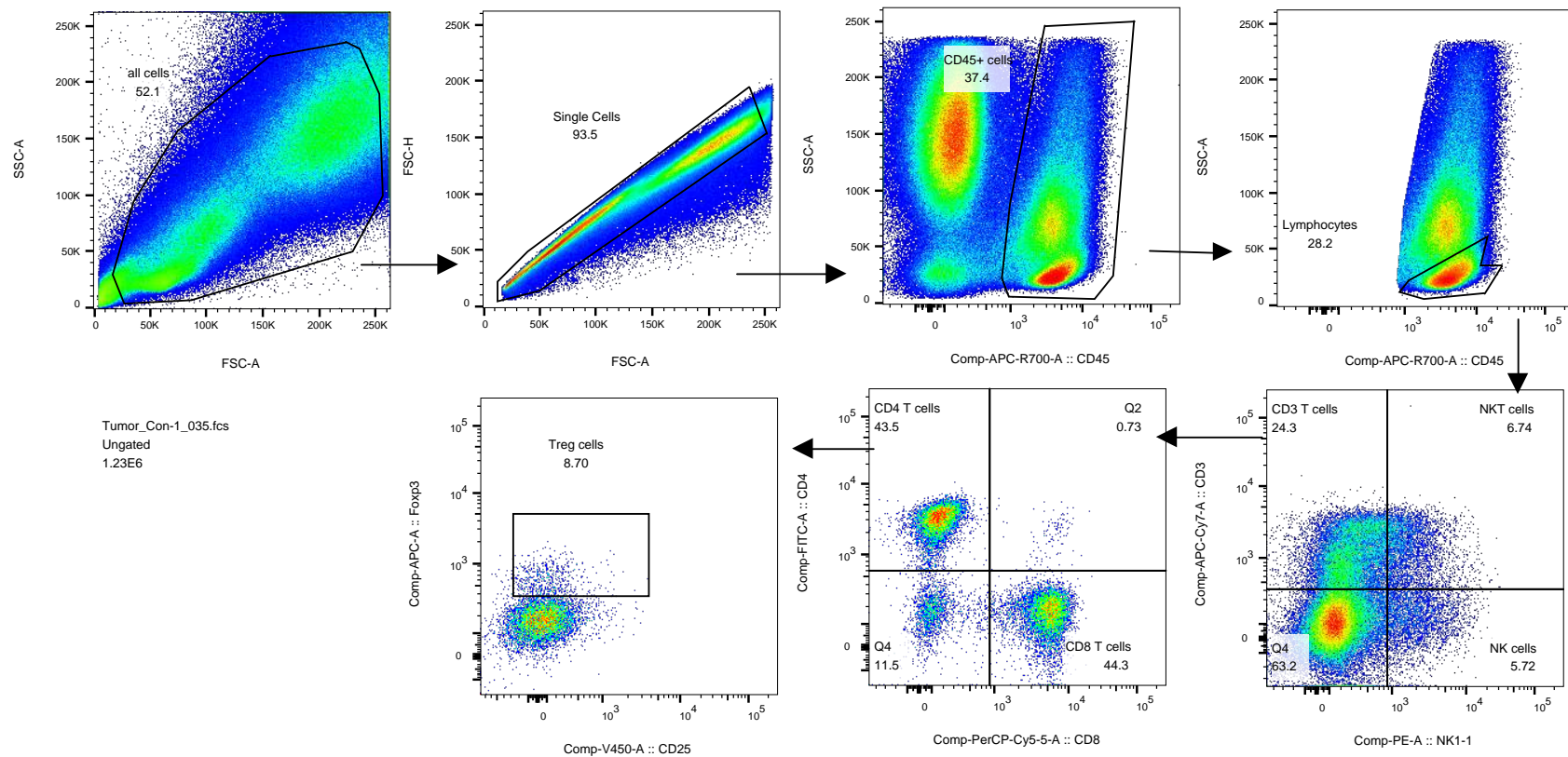

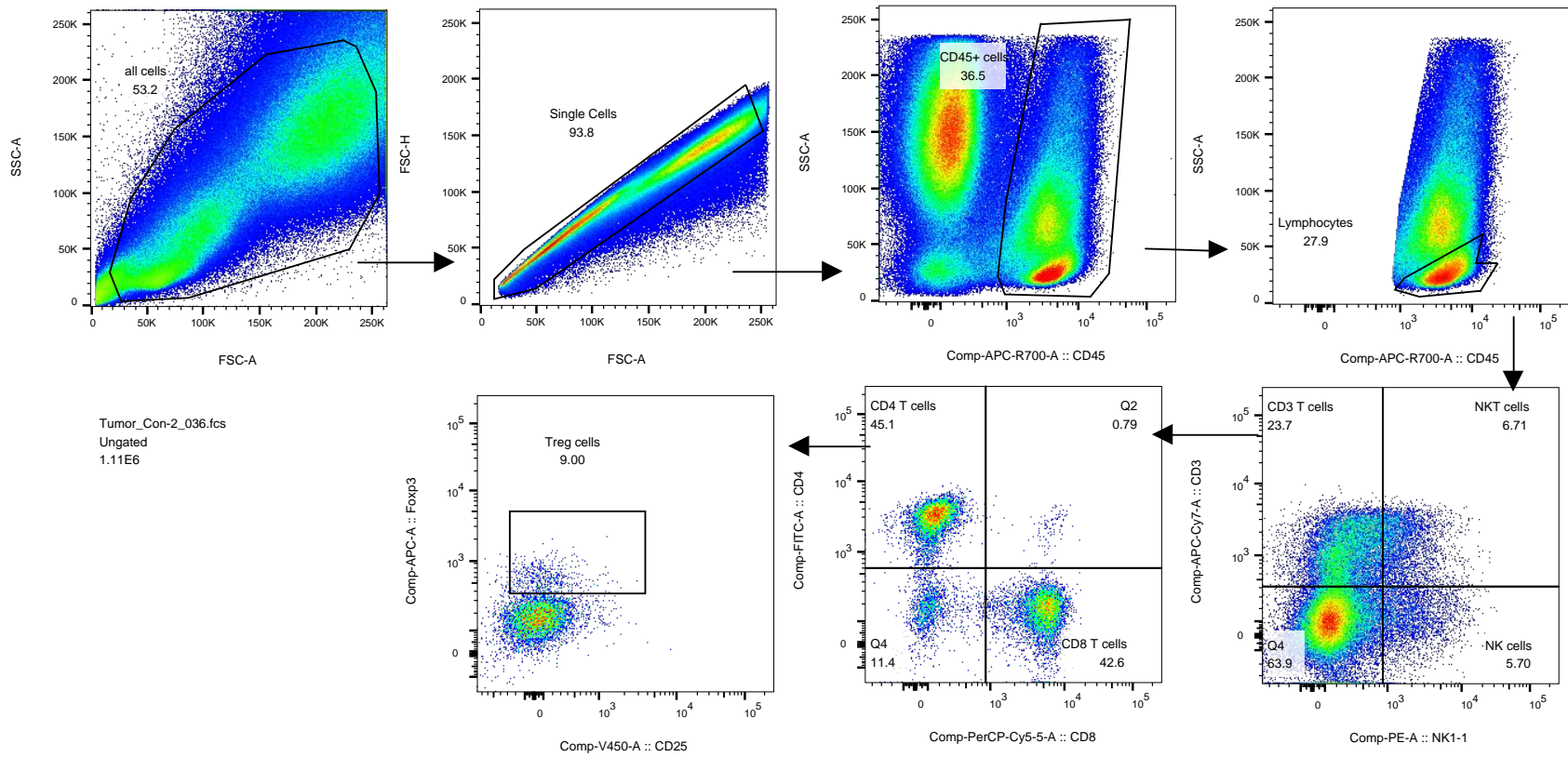

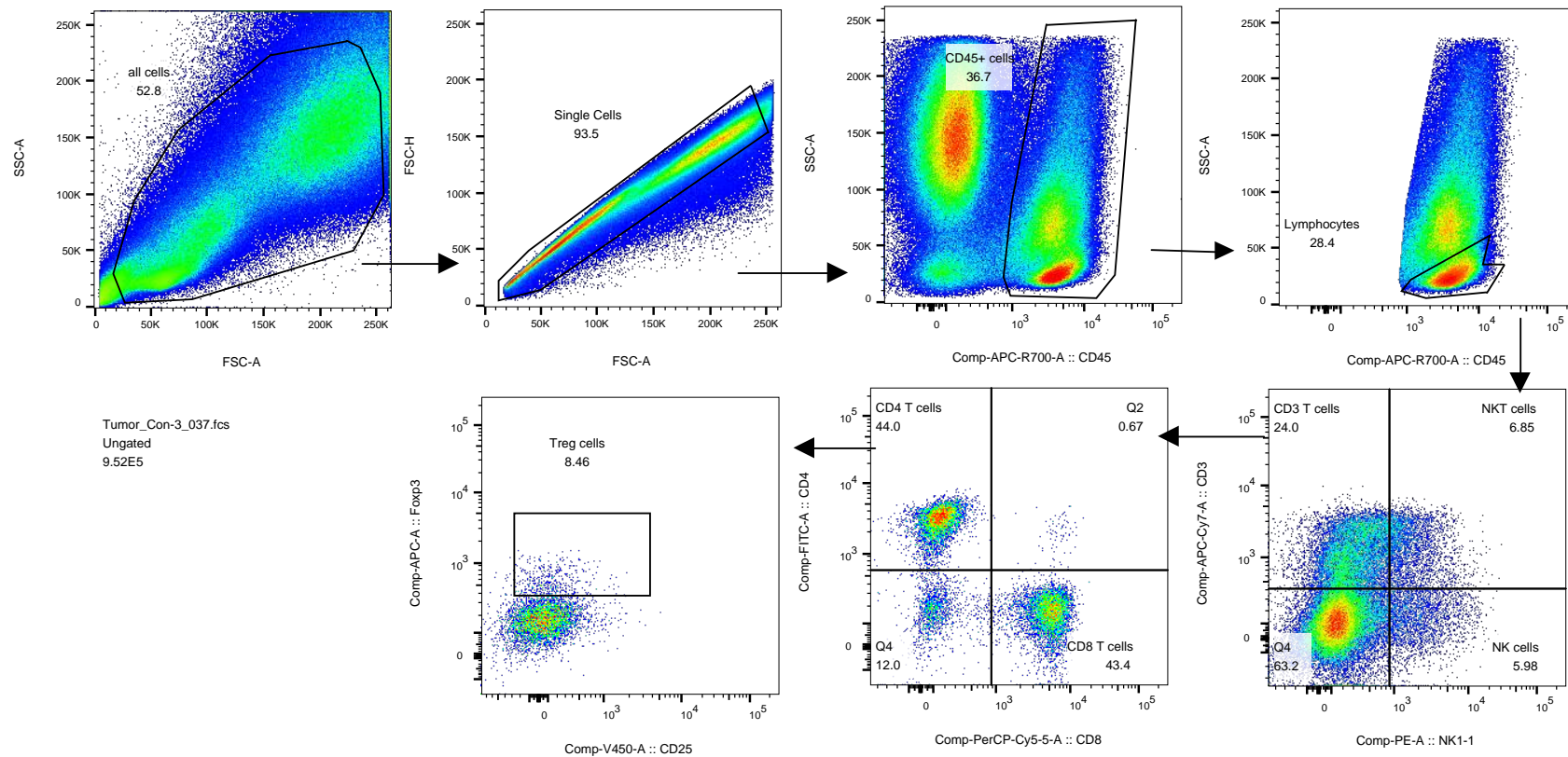

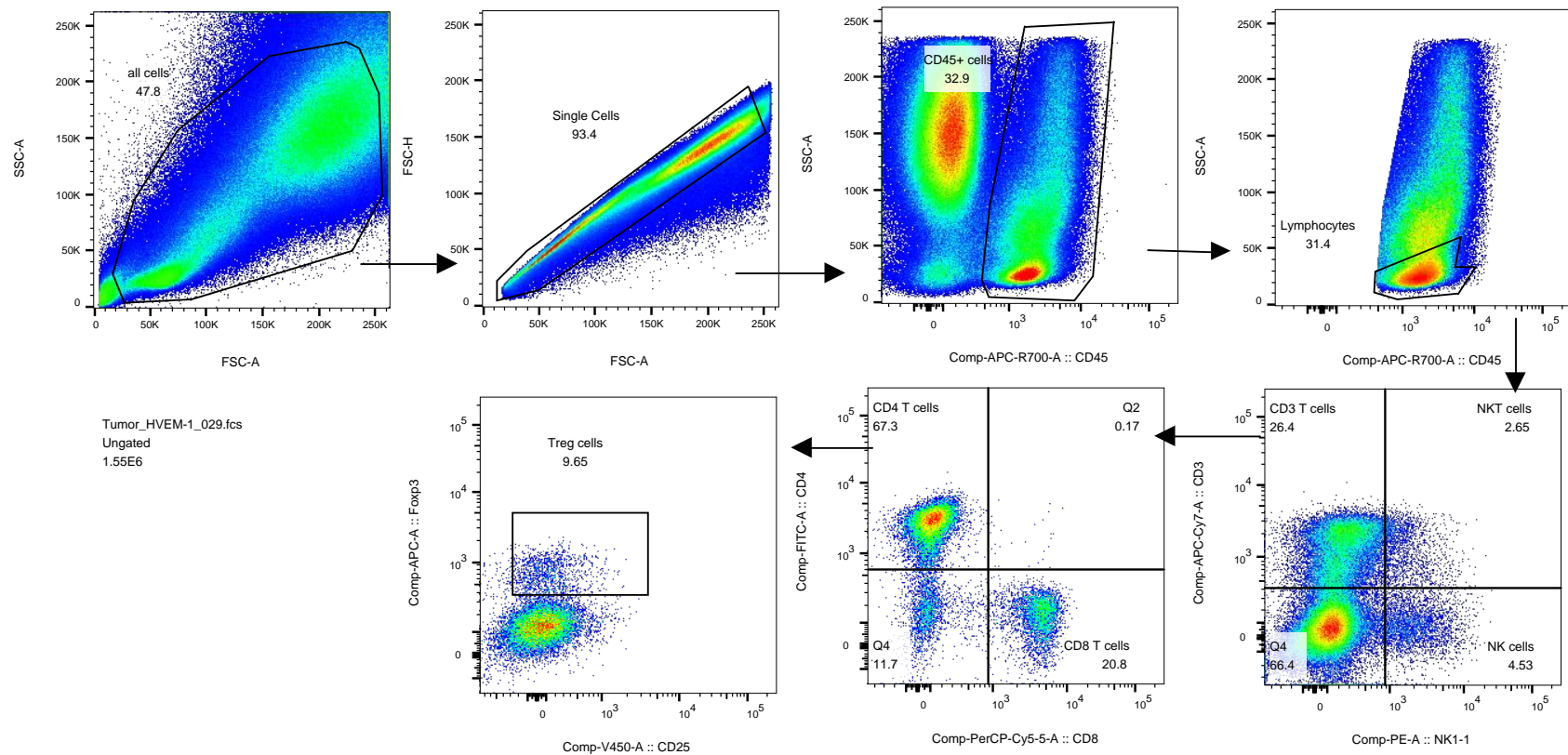

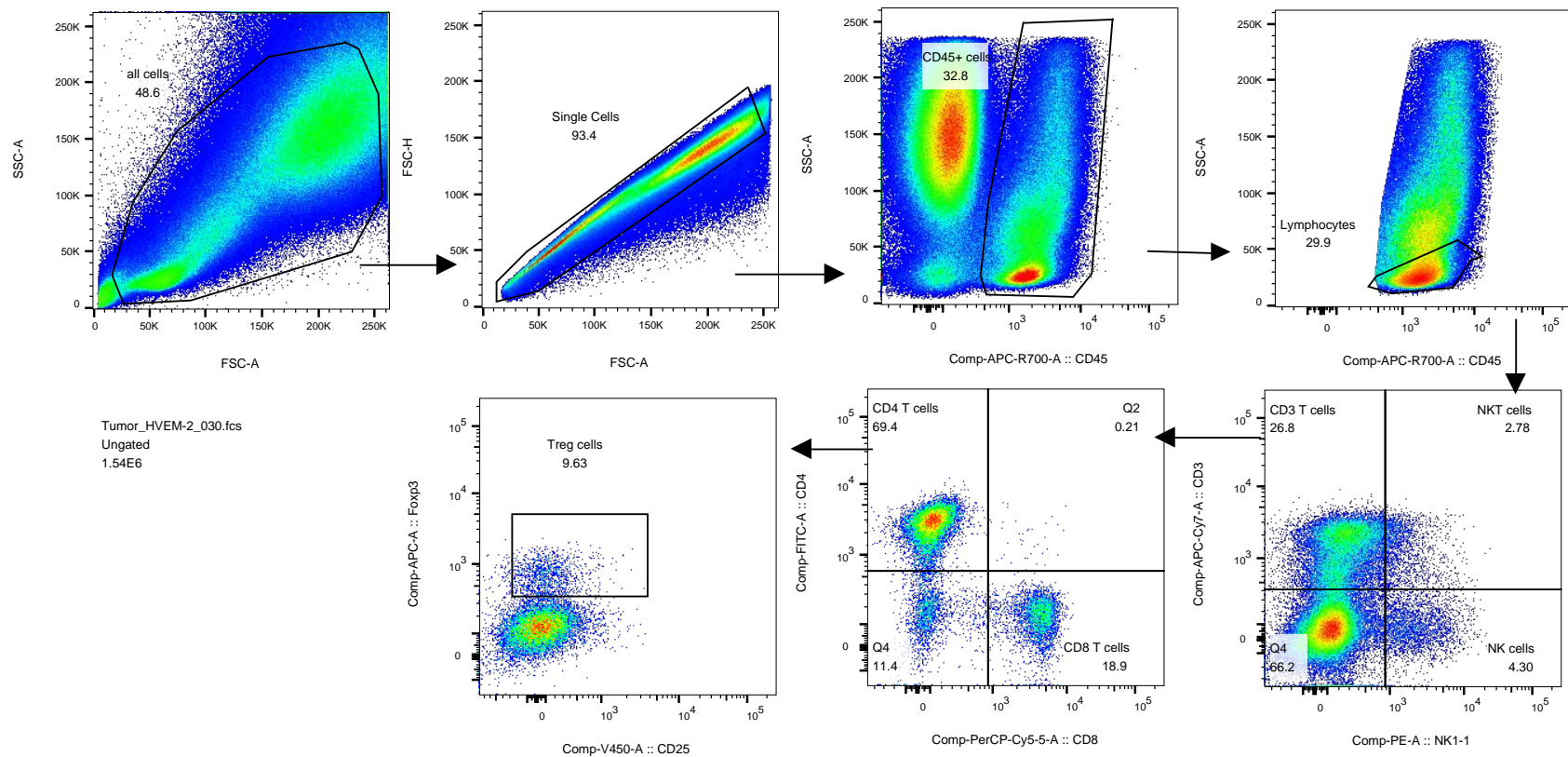

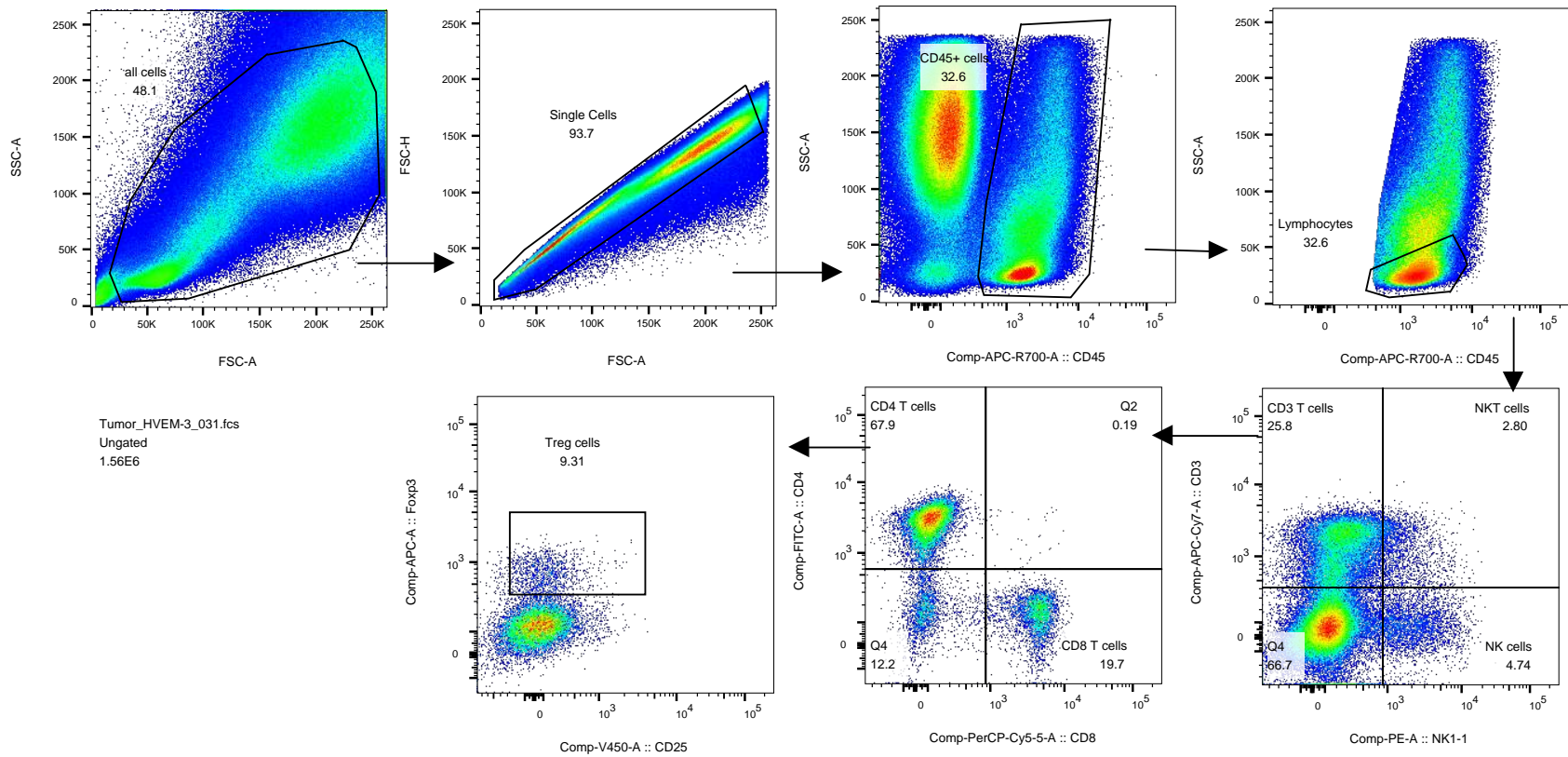

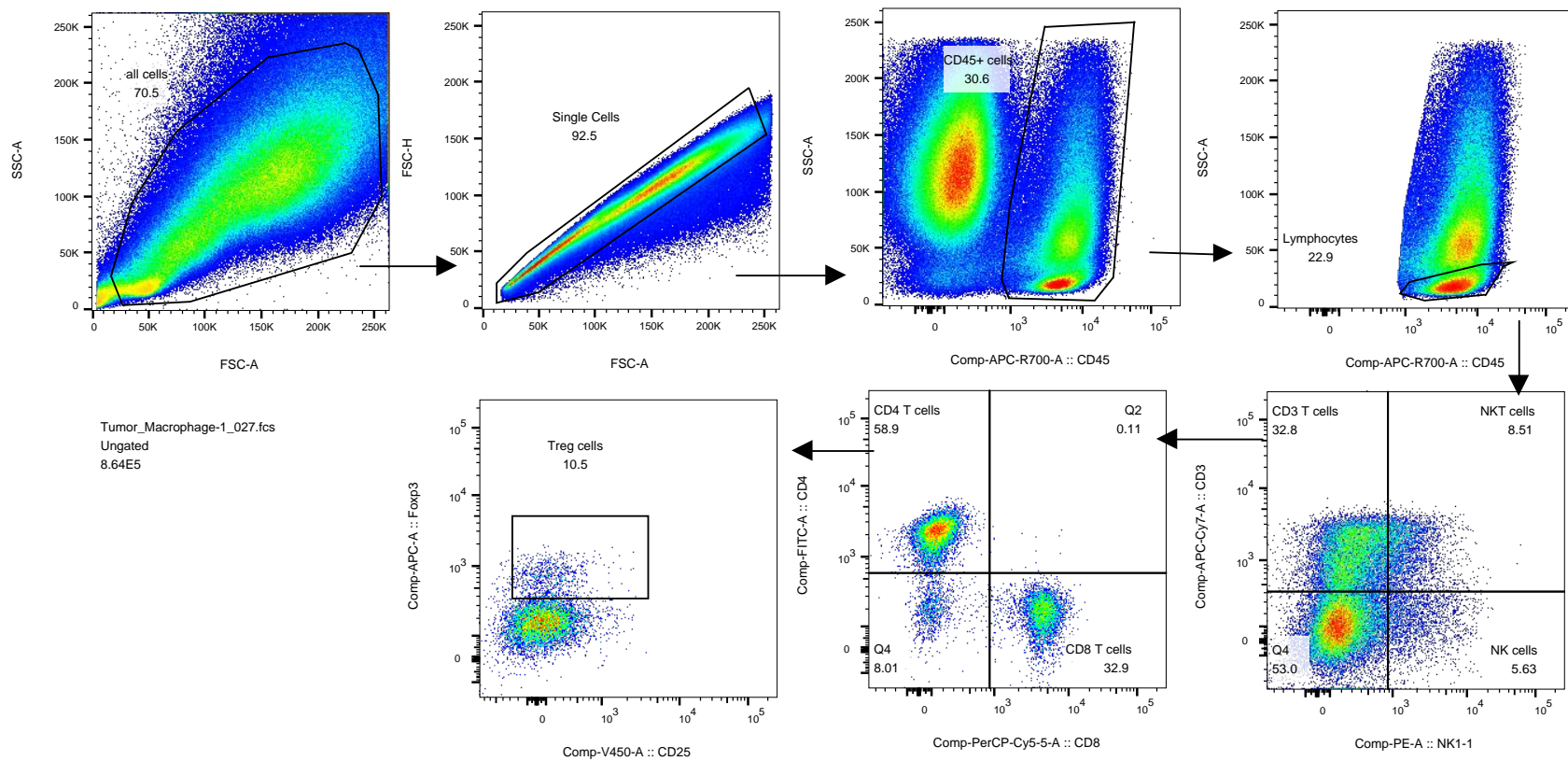

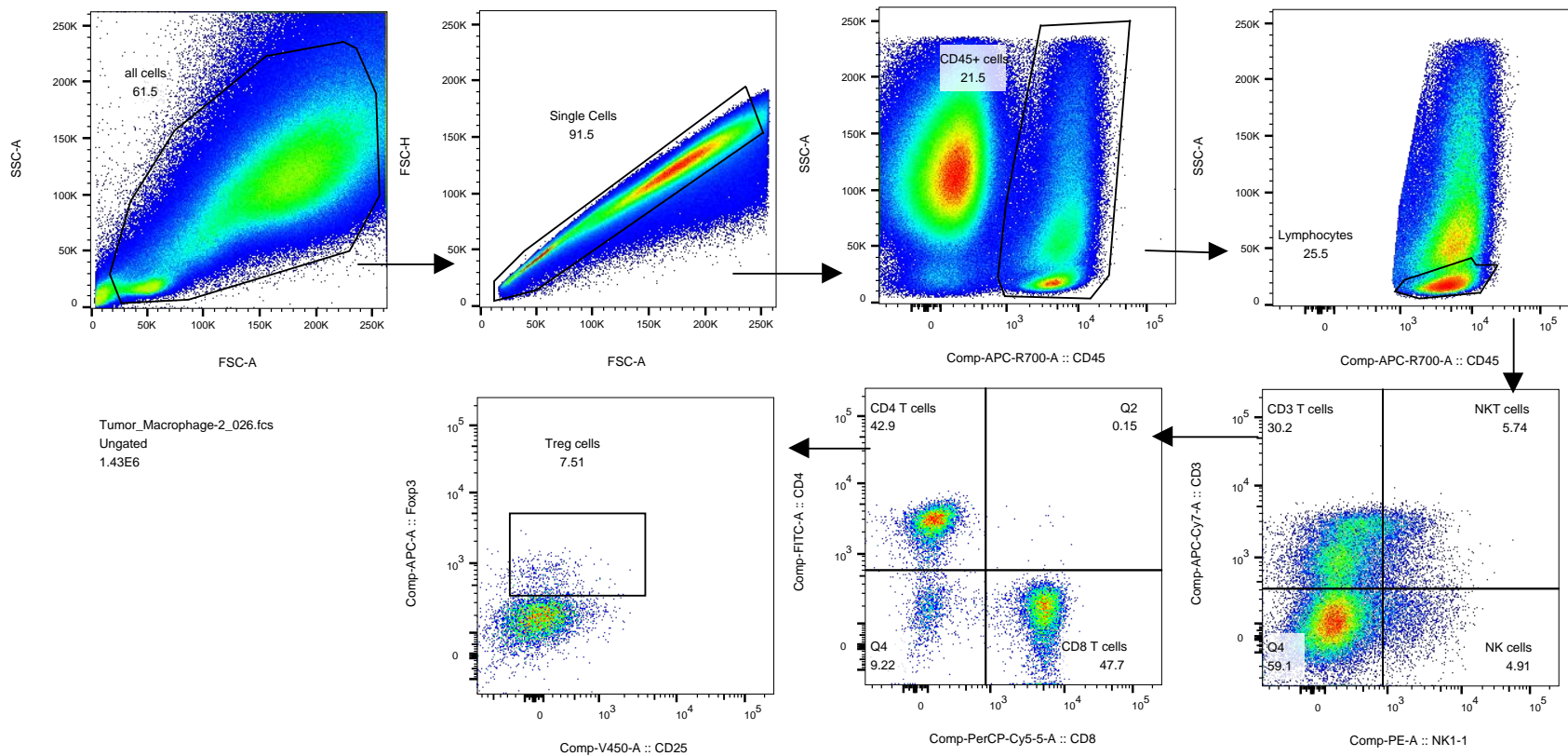

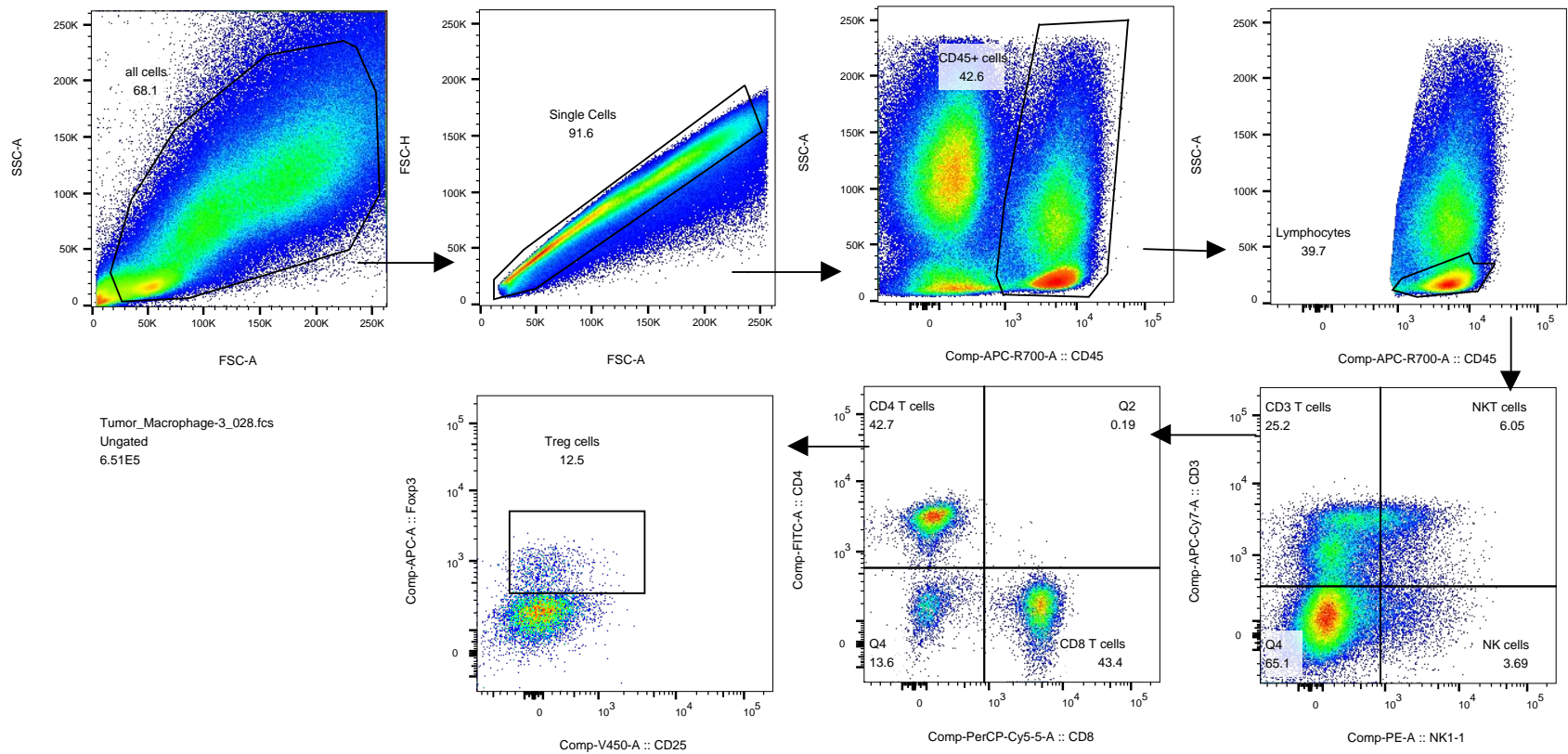

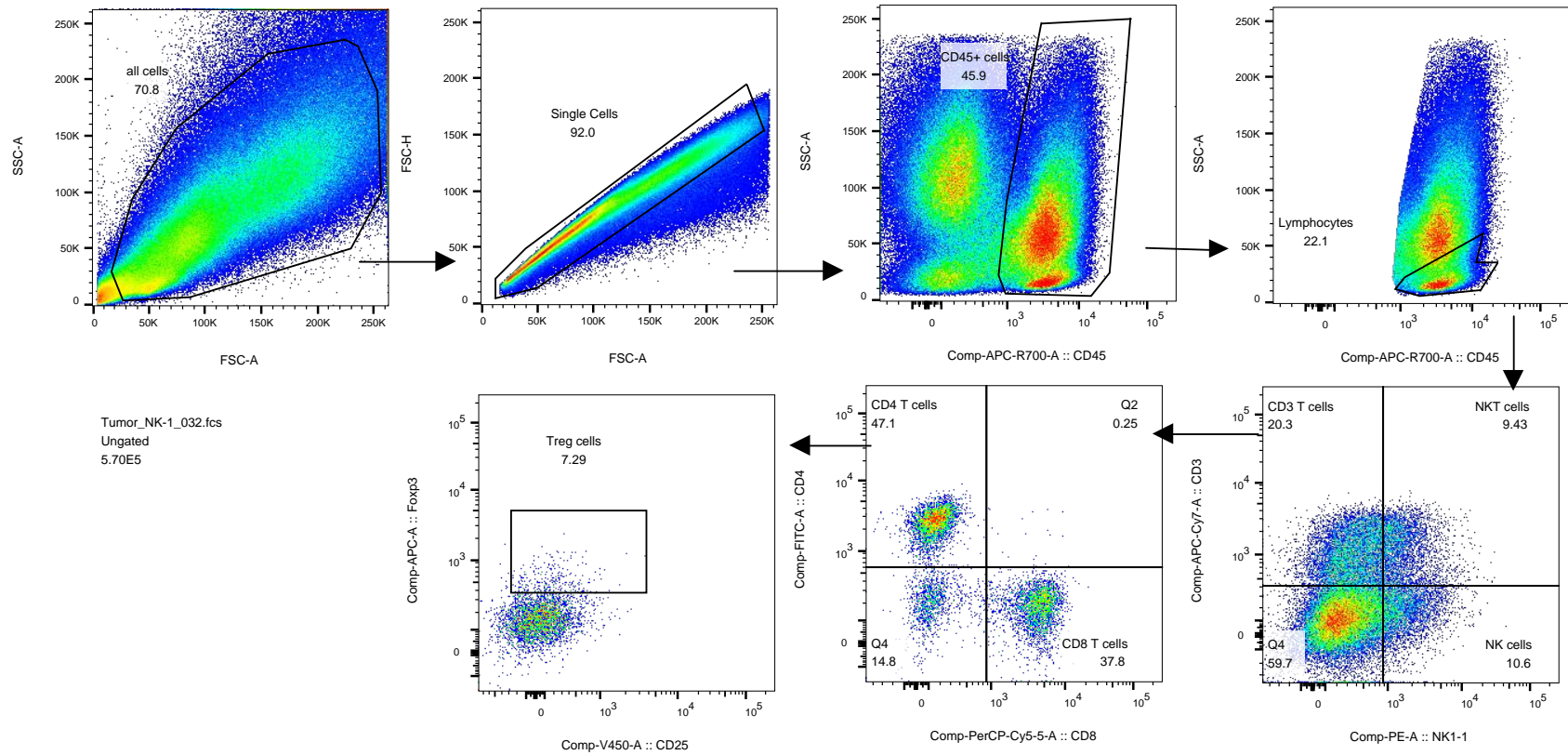

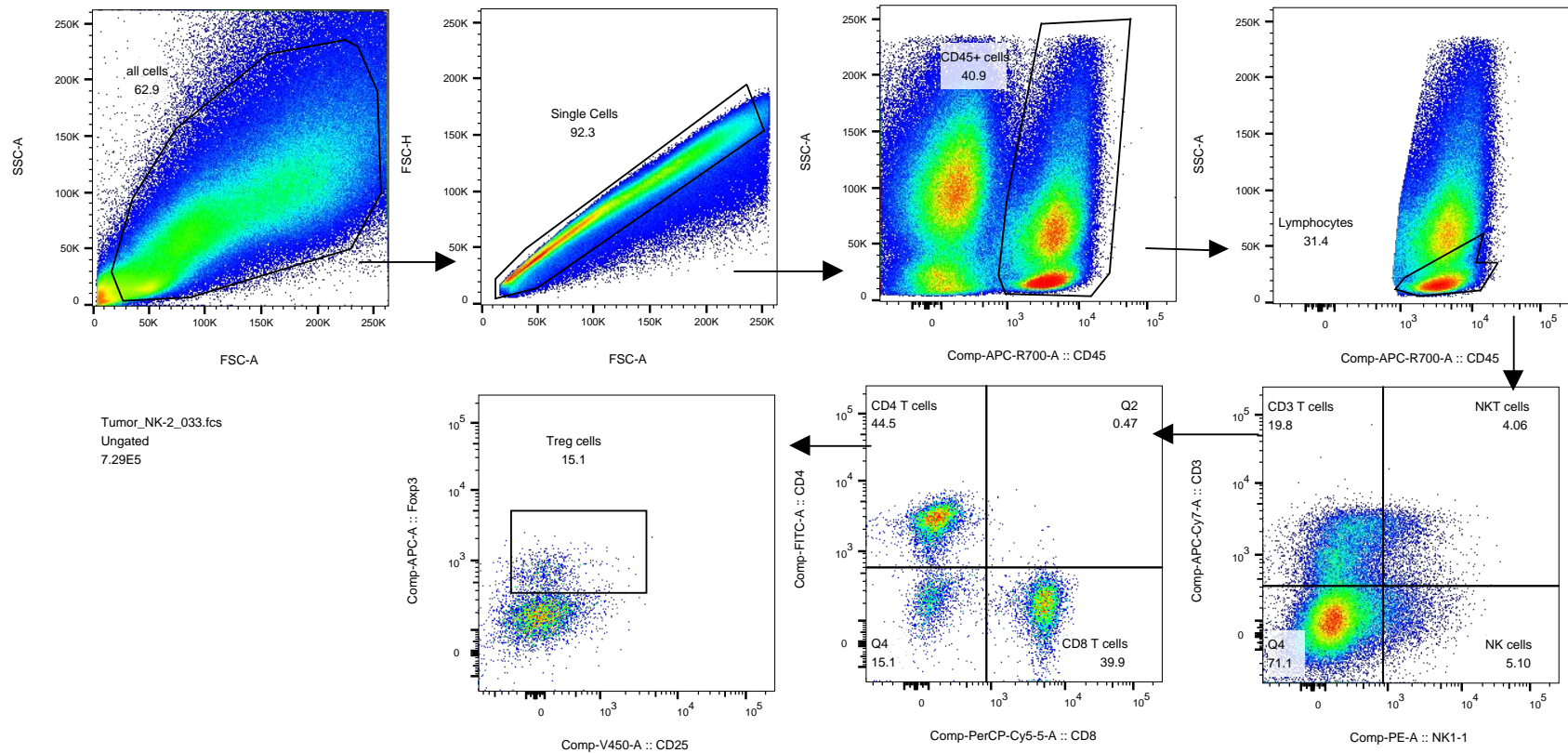

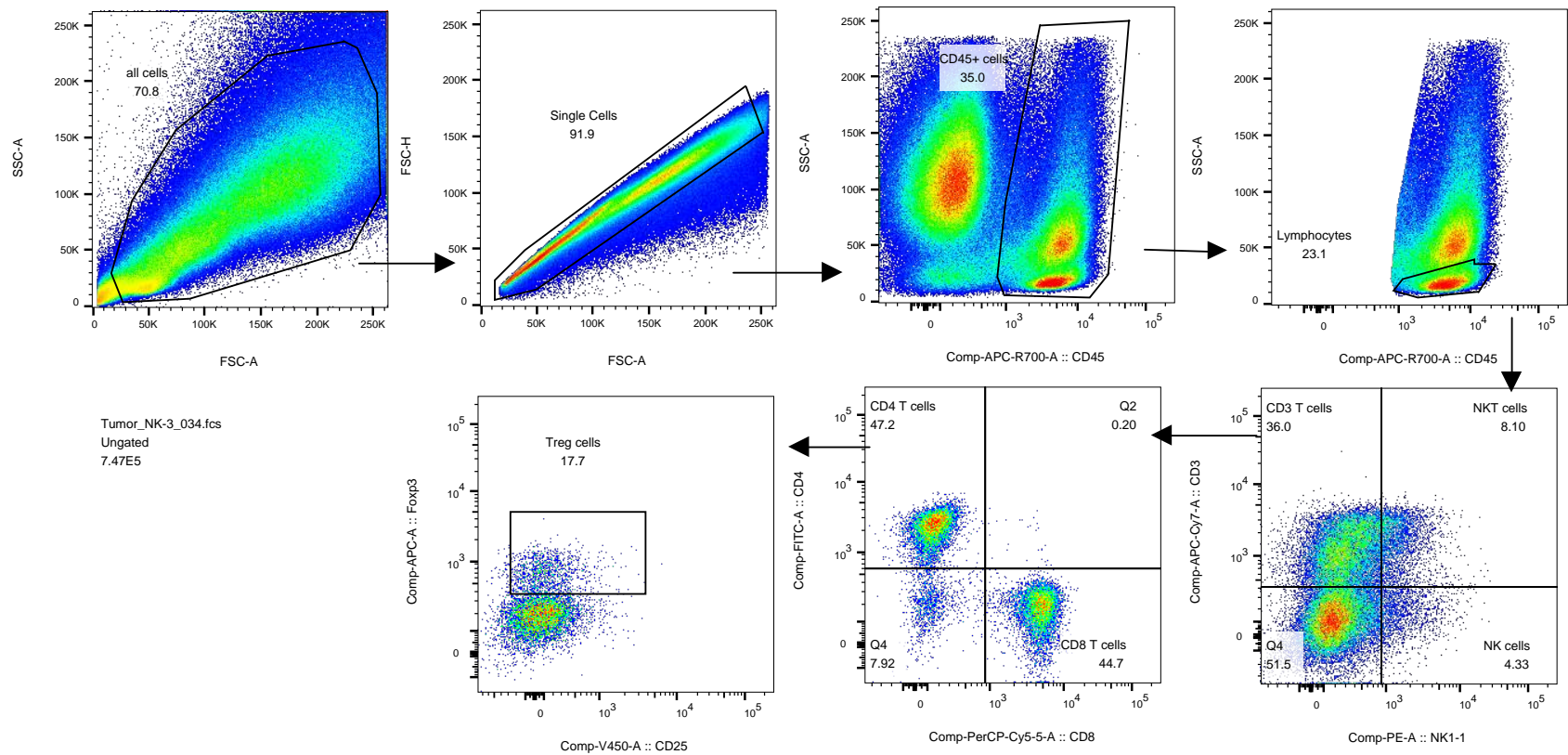

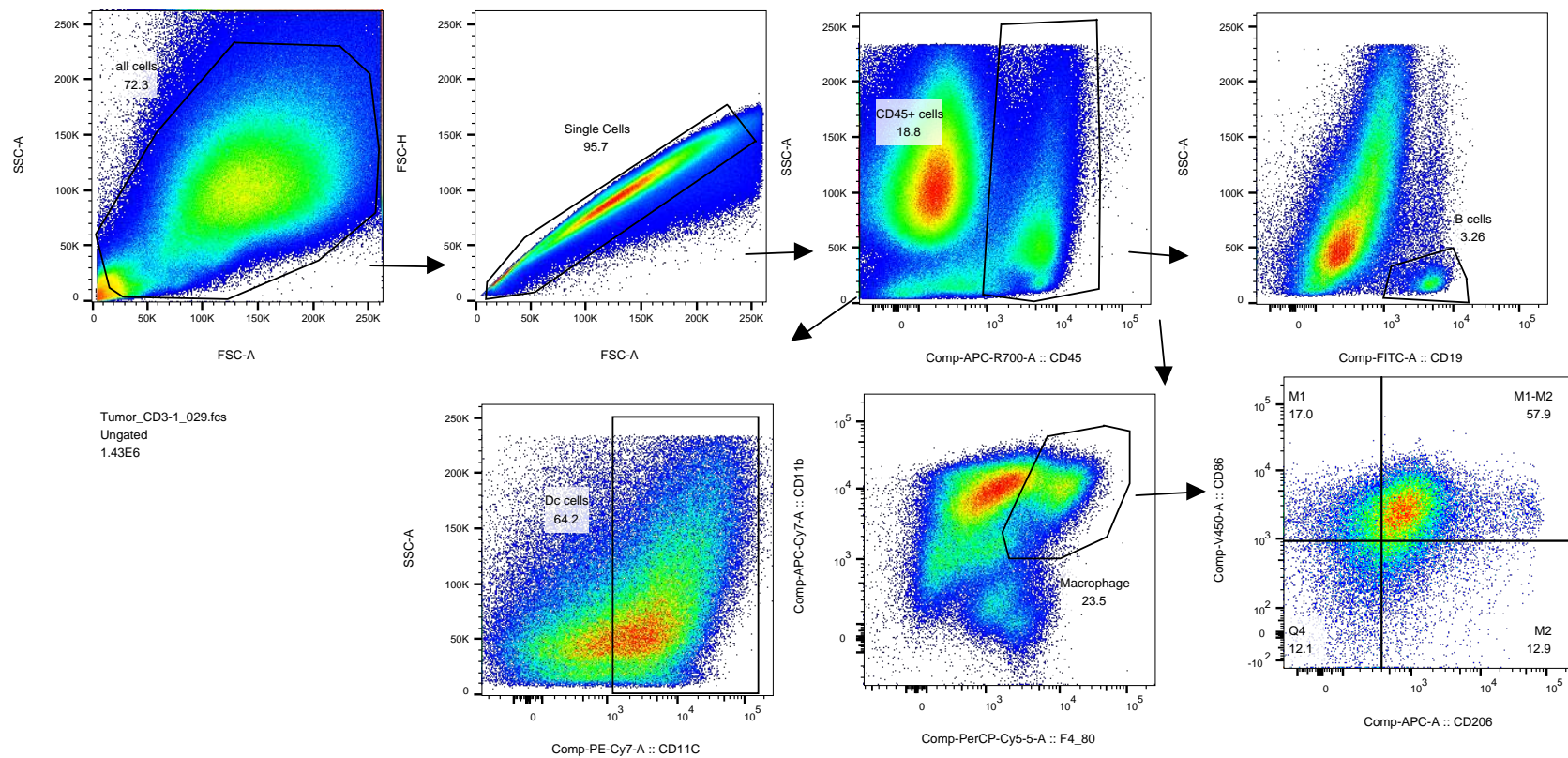

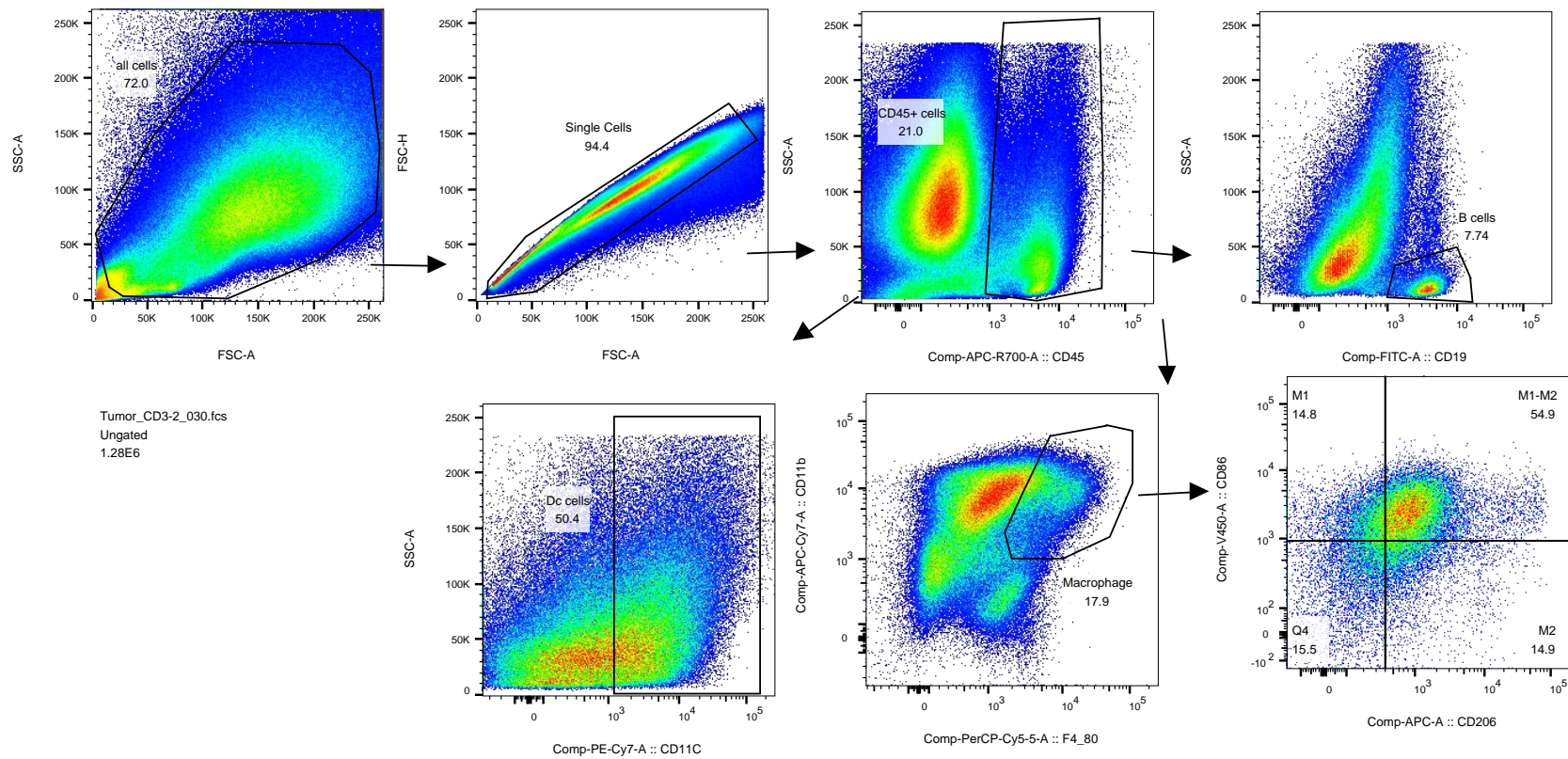

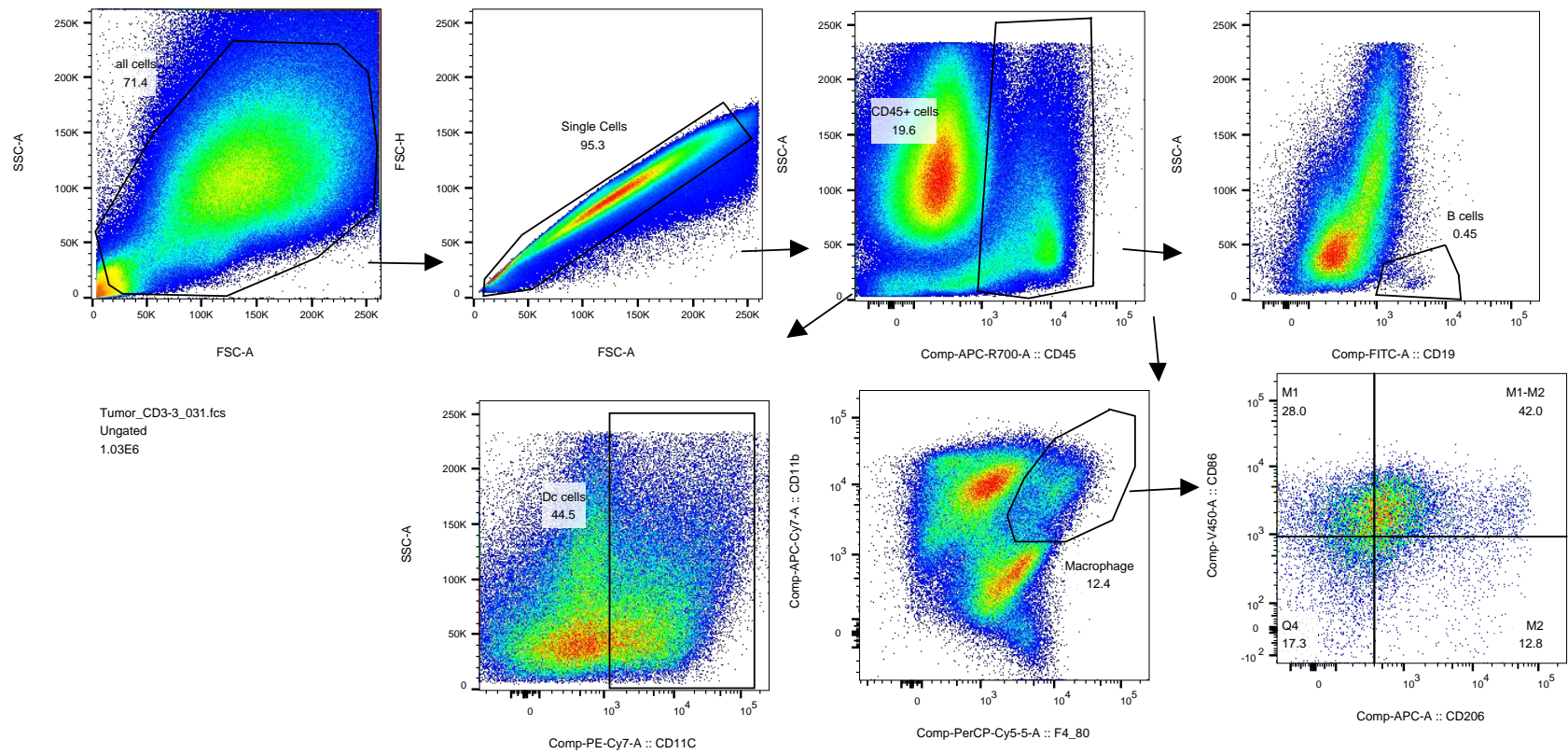

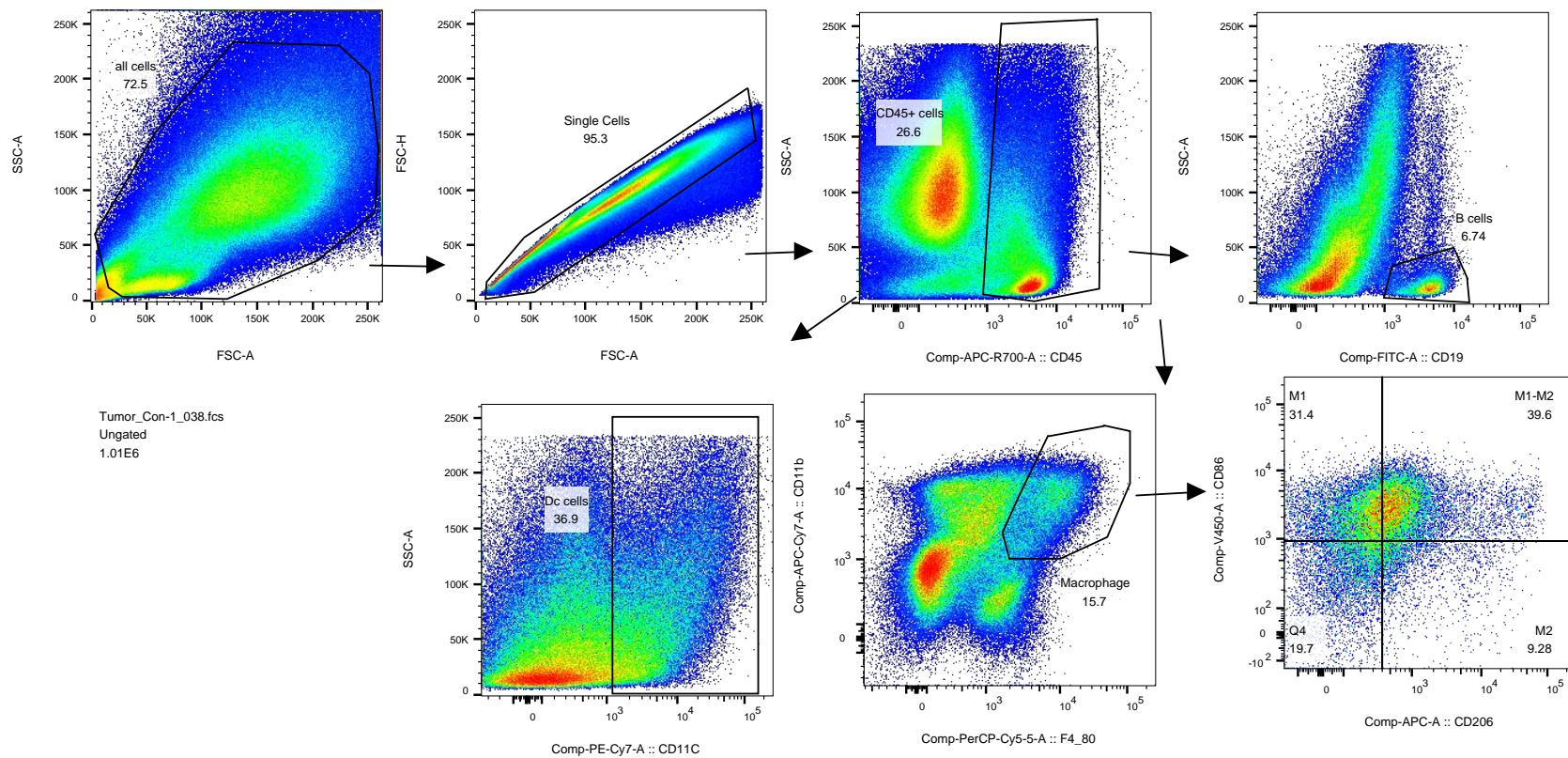

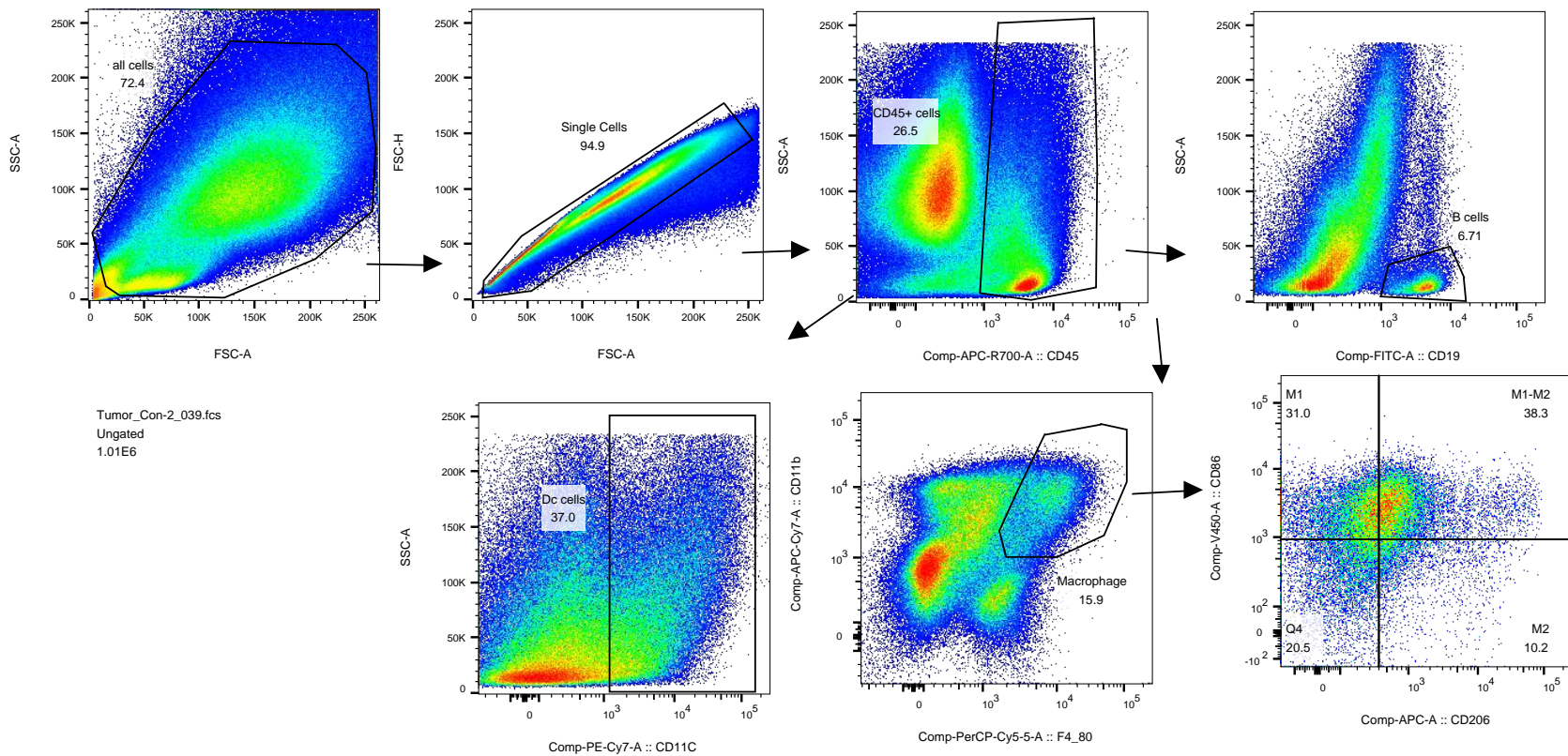

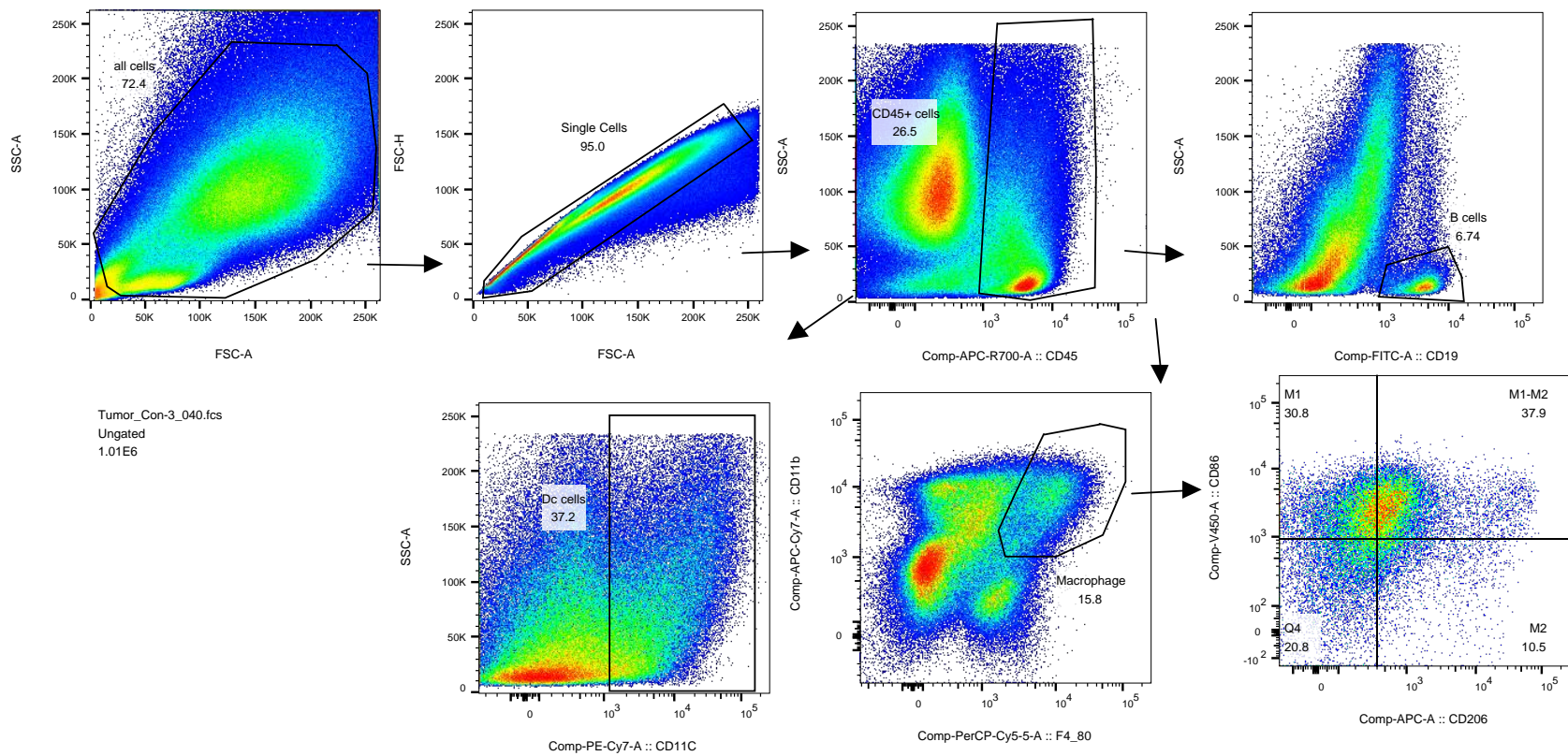

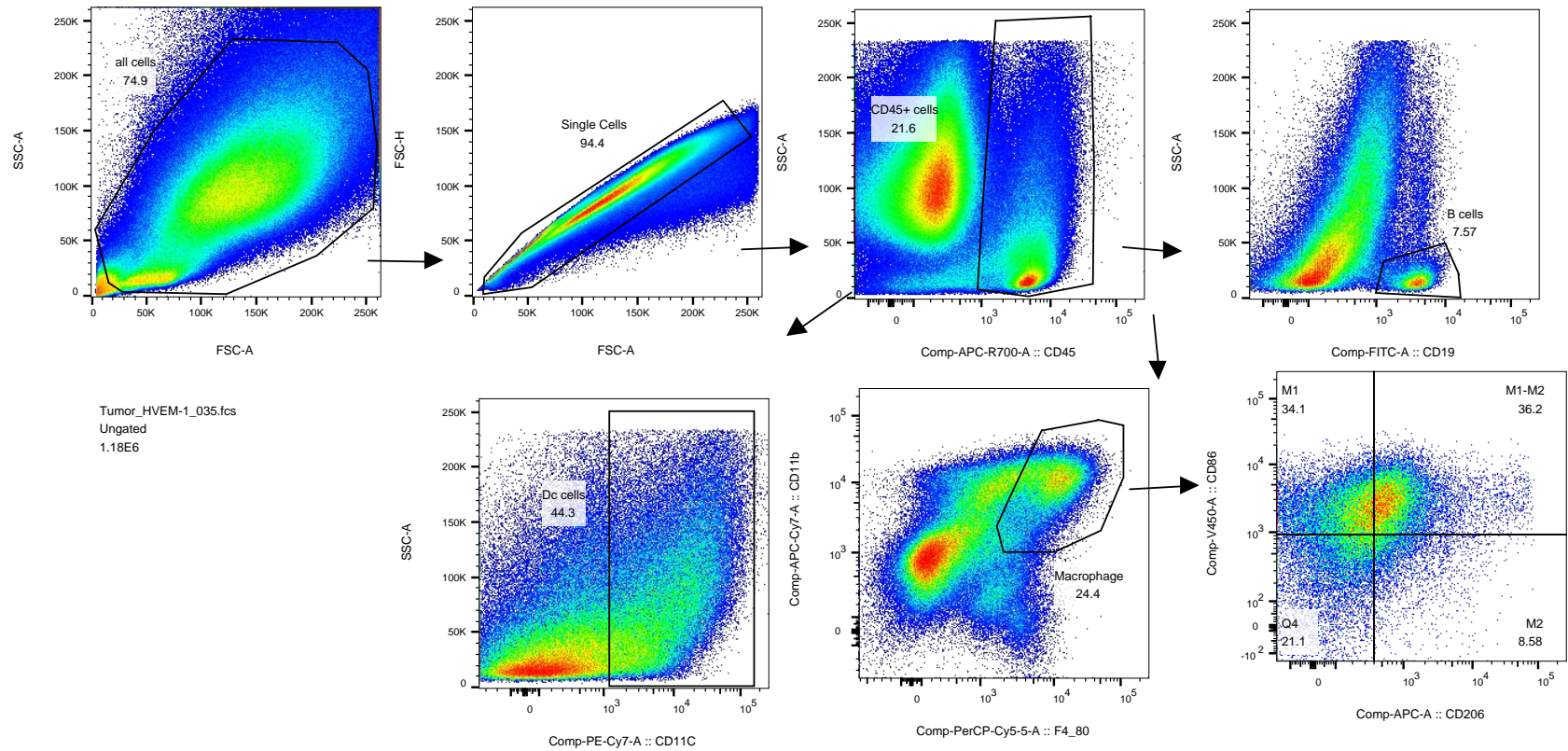

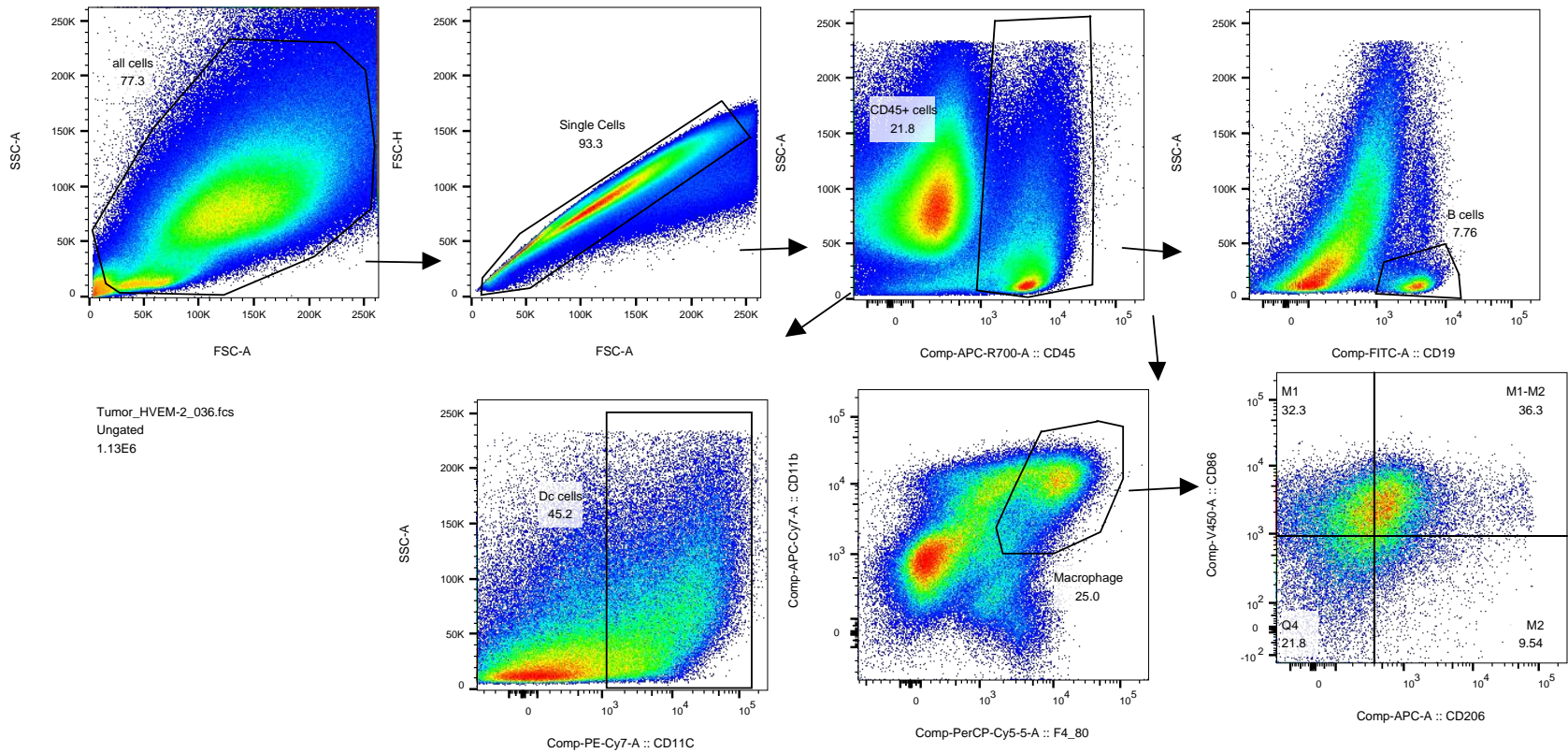

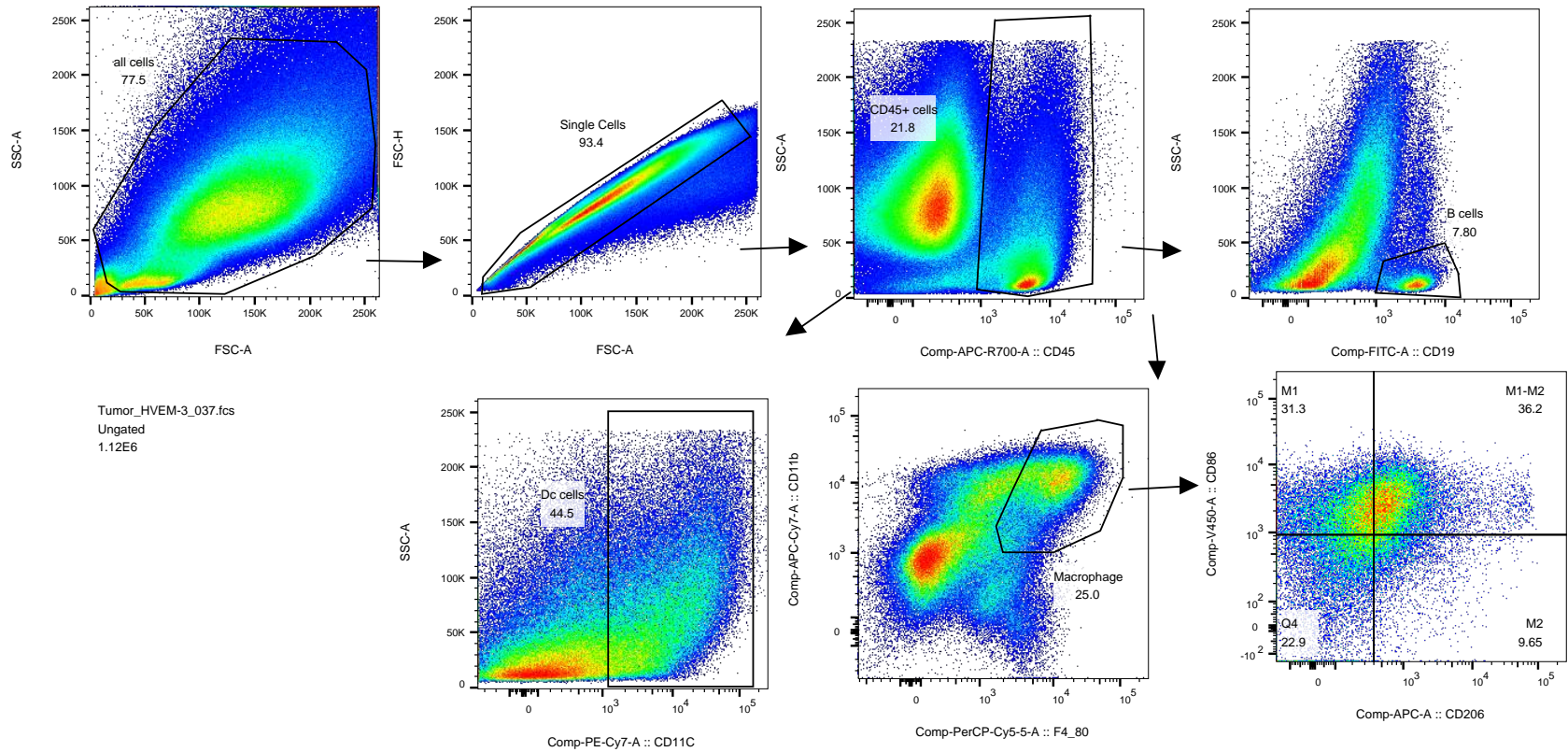

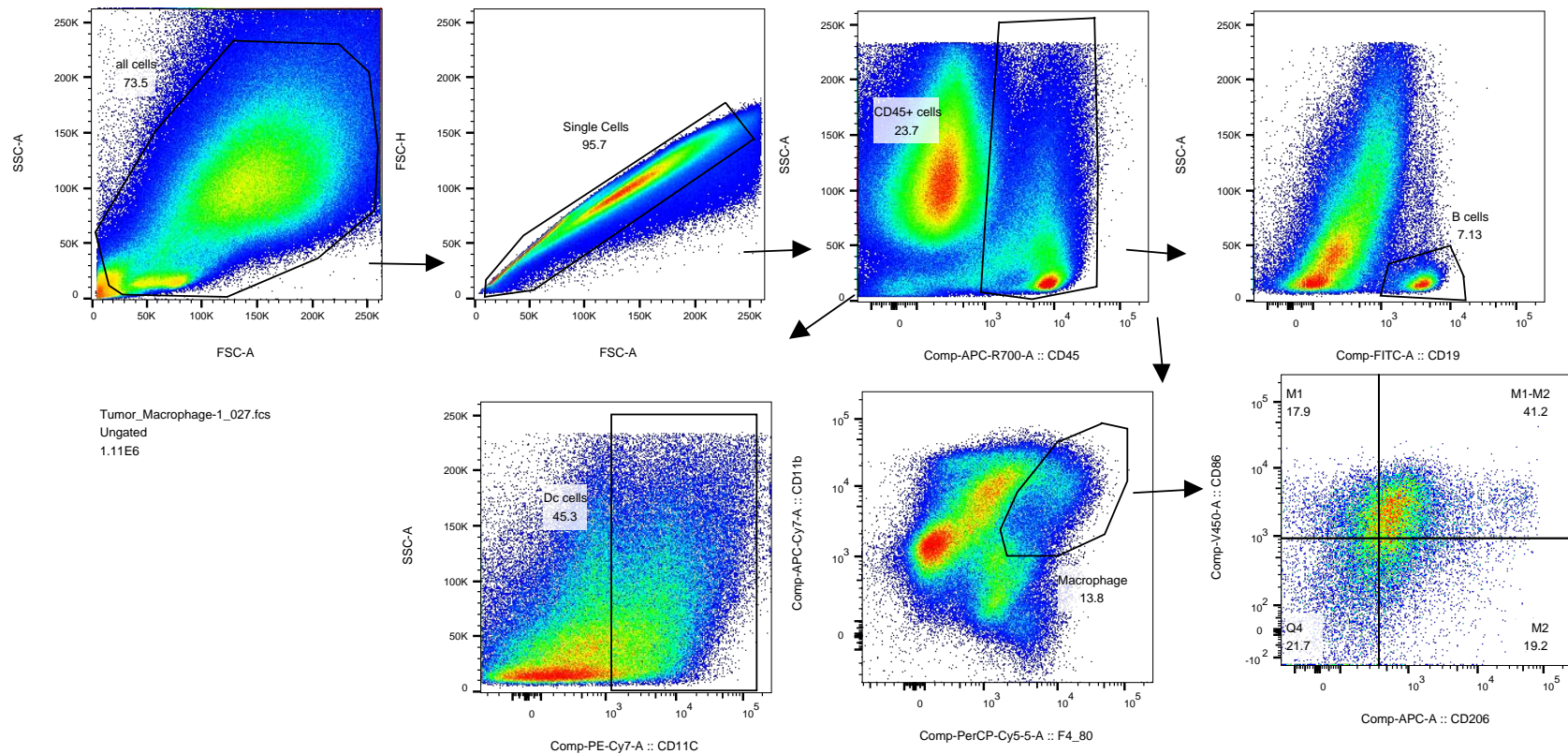

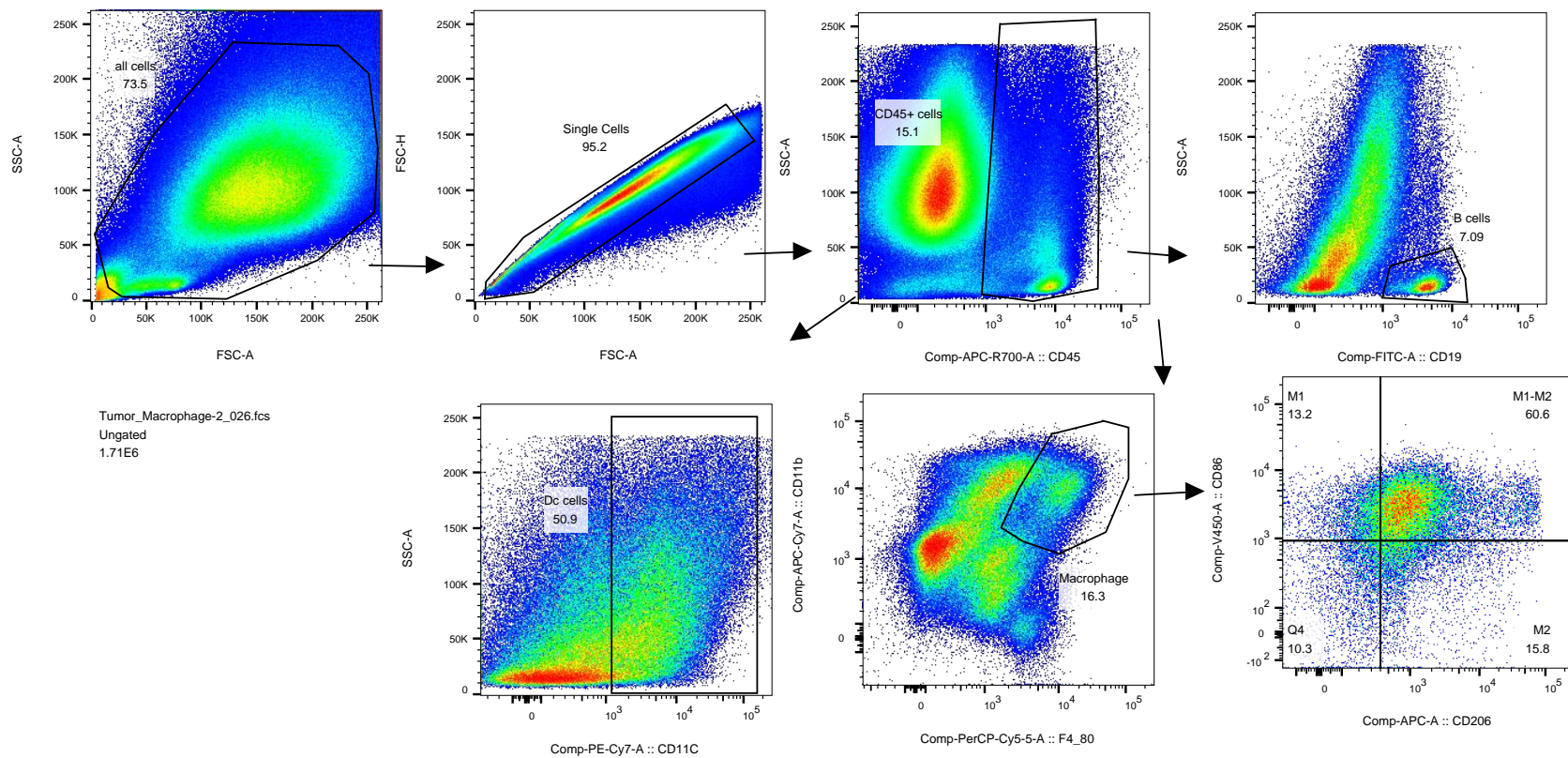

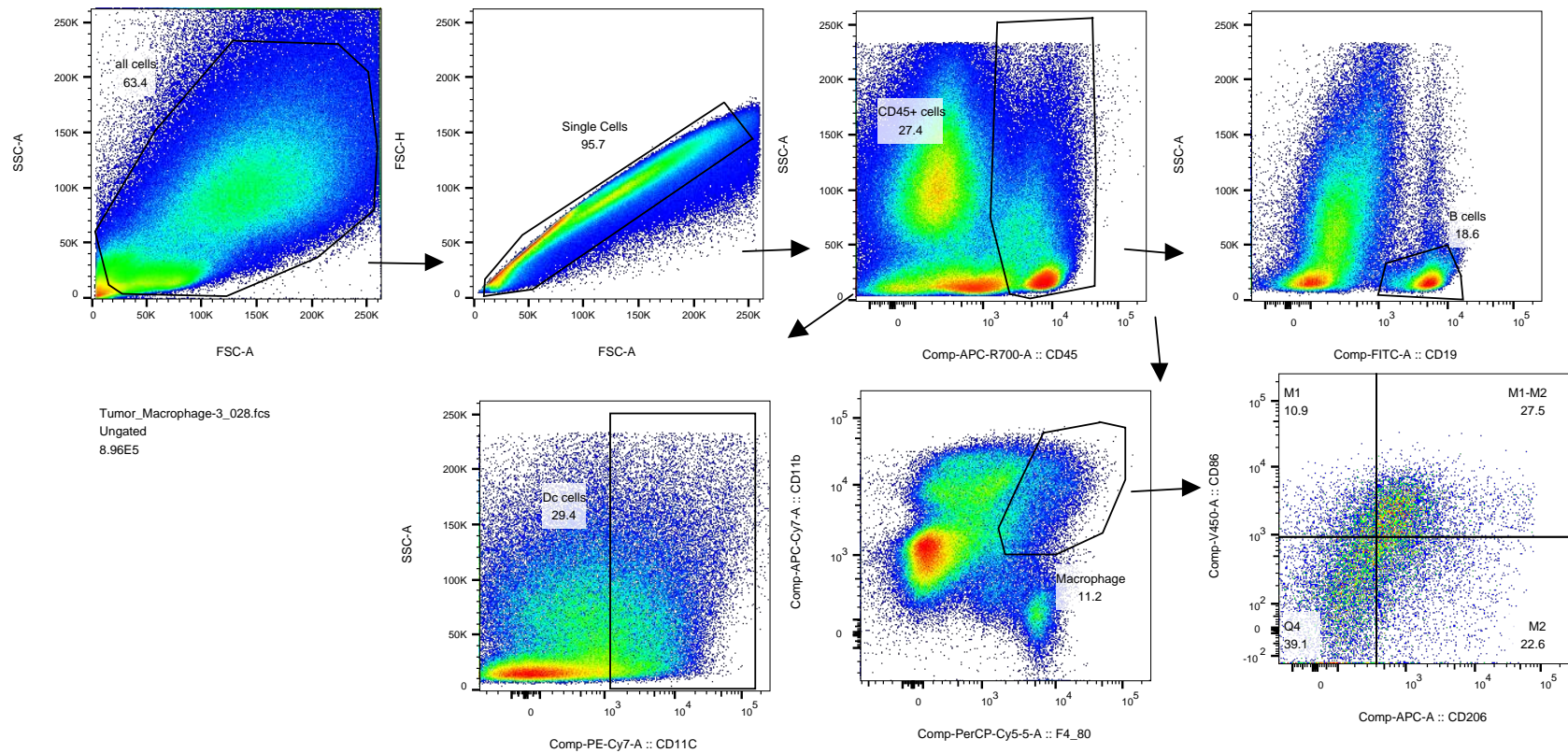

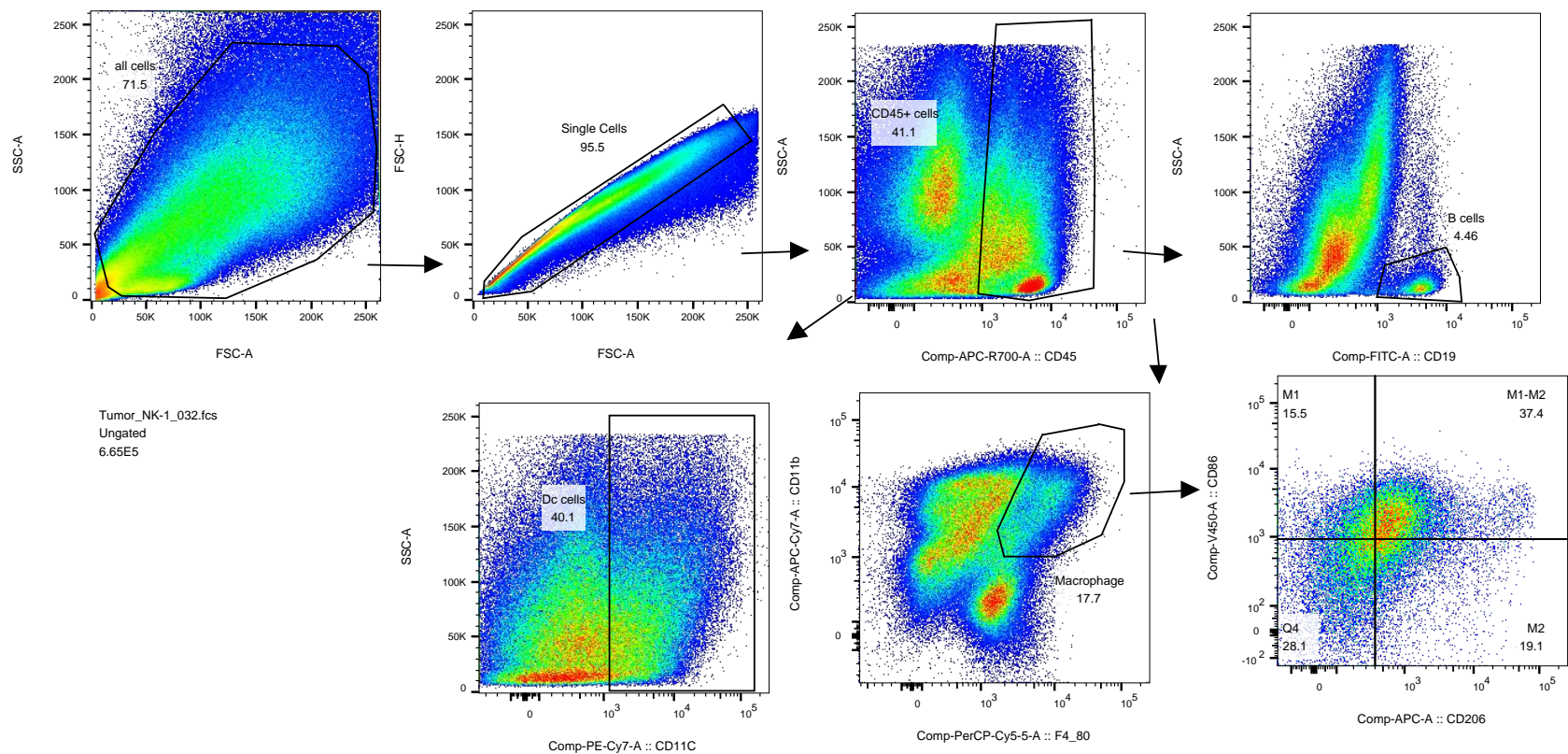

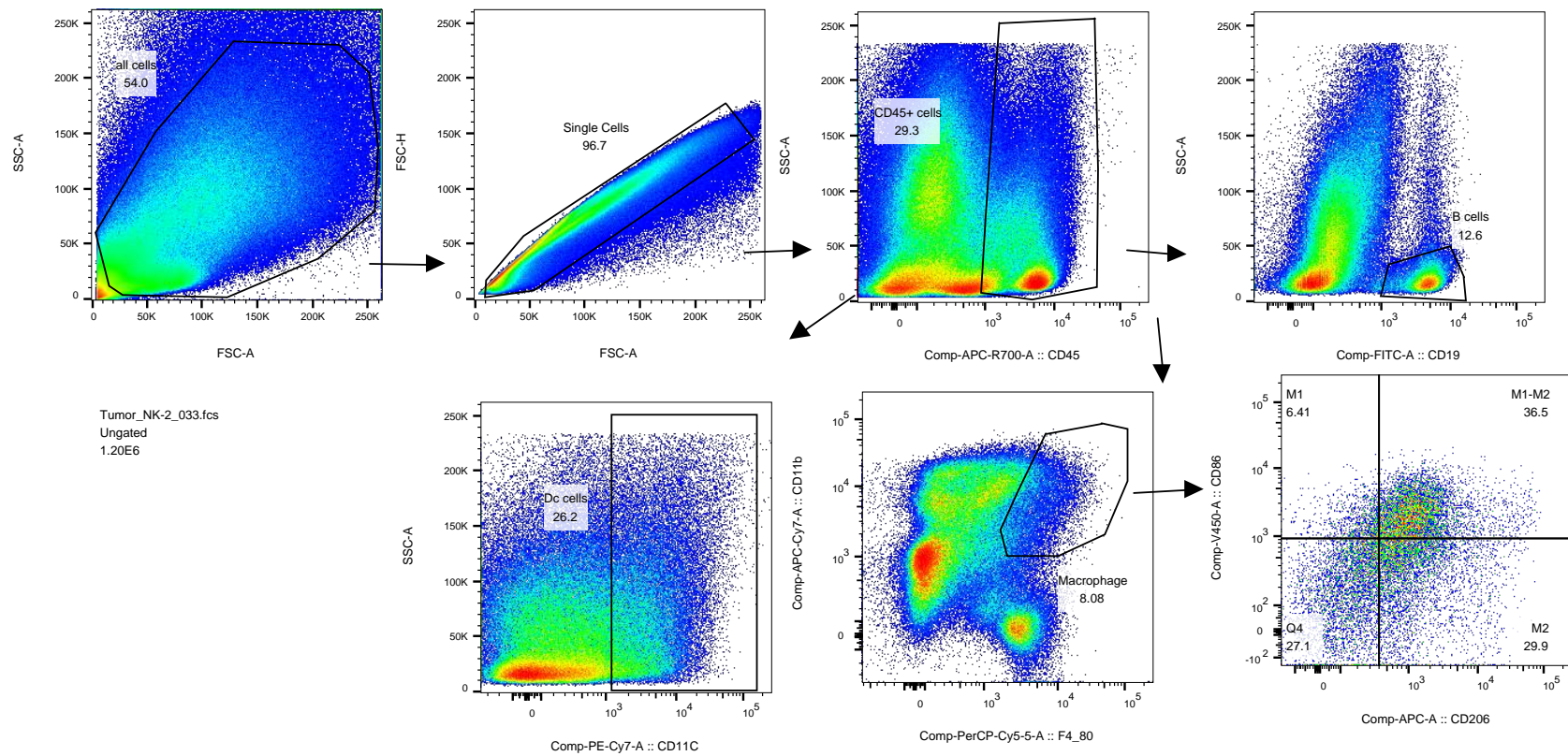

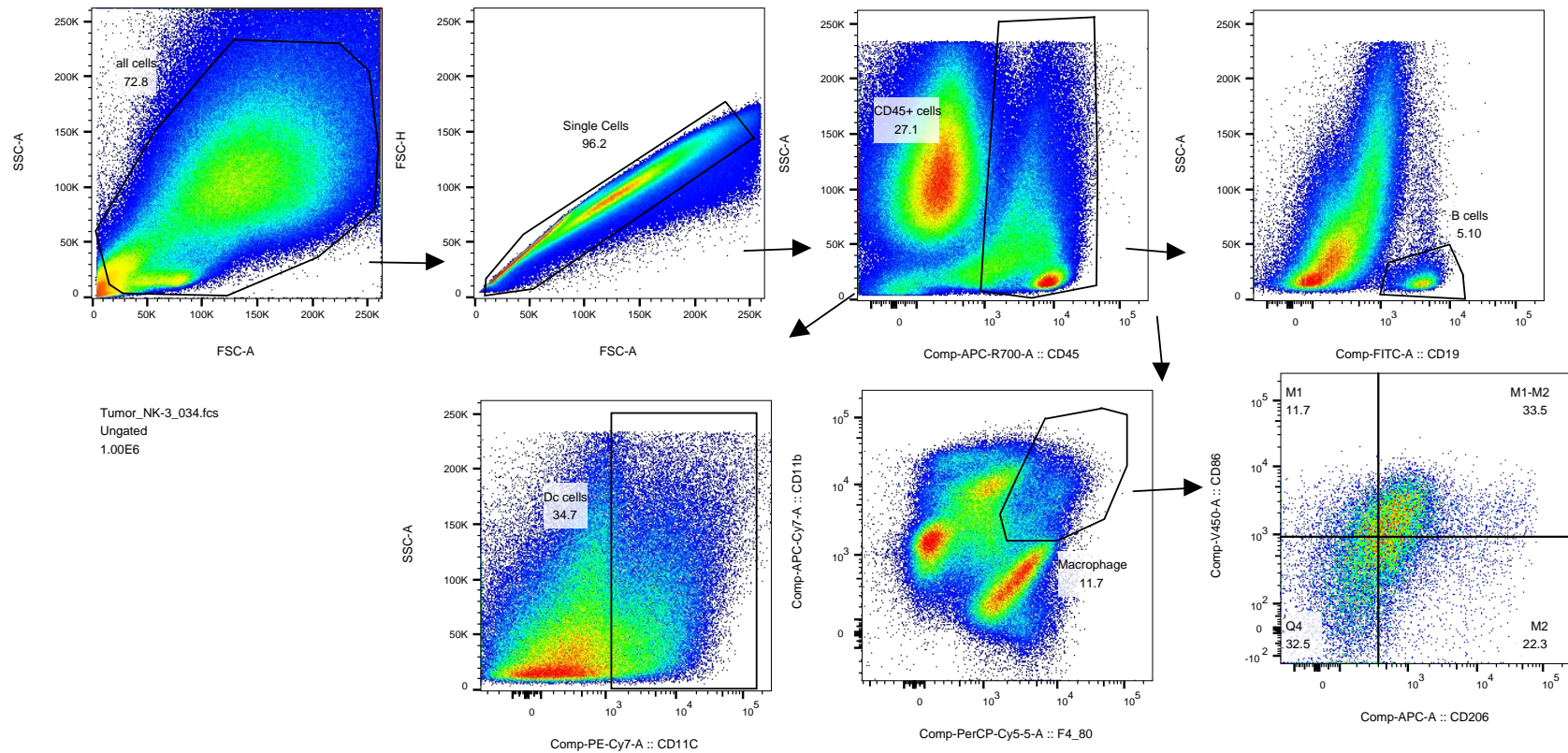

Supplement: Supplementary file 5 — Flow cytometry gating strategies for the identification of immune cell subsets. [file 13046_2025_3324_MOESM5_ESM.zip › Supplement File6.pdf]
