# Supplementary material for: A novel HVEM-Fc recombinant protein for lung cancer immunotherapy
Source: J Exp Clin Cancer Res. 2025 Feb 20;44:62. doi: 10.1186/s13046-025-03324-8 (PMC11841141; doi:10.1186/s13046-025-03324-8)
Supplement: Supplementary file 6 — Table S1 Baseline characteristics of the two patients. [file 13046_2025_3324_MOESM6_ESM.docx]

| Case | Case 1 |  | Case 2 |
| --- | --- | --- | --- |
| Sex | Male |  | Female |
| Age | 81 |  | 49 |
| pTNM | T2N2M0 |  | T2N1M0 |
| cTNM | T2N0M0 |  | T2N0M0 |
| Smoking History | Yes |  | No |
| PD-L1 expression level | 70% |  | 5% |
| EGFR mutation | No |  | Yes |
| ALK rearrangement | No |  | No |
| Family history  Tumor diameter  Pleural invasion  Histology | No  4cm  Yes  Lung squamous cancer |  | Yes  3cm  Yes  Lung adenocarcinoma cancer |

TableS1 Baseline characteristics of the patients
