# Supplementary material for: A novel HVEM-Fc recombinant protein for lung cancer immunotherapy
Source: J Exp Clin Cancer Res. 2025 Feb 20;44:62. doi: 10.1186/s13046-025-03324-8 (PMC11841141; doi:10.1186/s13046-025-03324-8)
Supplement: Supplementary file 7 — Table S2 Proportion of CD4+ and CD8+ T Cells derived from PBMCs. [file 13046_2025_3324_MOESM7_ESM.docx]

| Marker | Case1 |  | Case2 |
| --- | --- | --- | --- |
| CD45+ | 99.78% |  | 98.64% |
| CD3+/CD45+ | 90.83% |  | 91.27% |
| CD4+/CD3+ | 59.81% |  | 61.34% |
| CD8+/CD3+ | 36.31% |  | 33.47% |

TableS2 CD4+ and CD8+ T Cell Proportion deriving from PBMC
